# Advancing PROTAC Characterization: Structural Insights through Adducts and Multimodal Tandem-MS Strategies

**DOI:** 10.1021/jasms.3c00342

**Published:** 2024-01-10

**Authors:** Mohammed Rahman, Bryan Marzullo, Stephen W. Holman, Mark Barrow, Andrew D. Ray, Peter B. O’Connor

**Affiliations:** † Department of Chemistry, University of Warwick, Coventry, CV4 7AL, U.K.; ‡ Department of Physics, University of Warwick, Coventry, CV4 7AL, U.K.; § Chemical Development, Pharmaceutical Technology & Development, Operations, AstraZeneca, Macclesfield, SK10 4TF, U.K.; ∥ New Modalities and Parenteral Development, Pharmaceutical Technology & Development, Operations, AstraZeneca, Macclesfield, SK10 4TF, U.K.

## Abstract

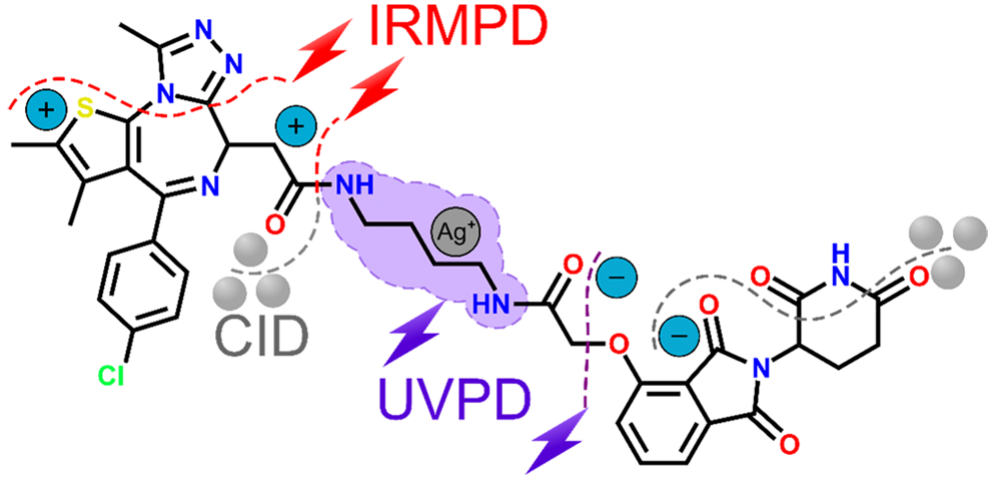

Proteolysis targeting chimeras (PROTACs) are specialized molecules that bind to a target protein and a ubiquitin ligase to facilitate protein degradation. Despite their significance, native PROTACs have not undergone tandem mass spectrometry (MS) analysis. To address this gap, we conducted a pioneering investigation on the fragmentation patterns of two PROTACs in development, dBET1 and VZ185. Employing diverse cations (sodium, lithium, and silver) and multiple tandem-MS techniques, we enhanced their structural characterization. Notably, lithium cations facilitated comprehensive positive-mode coverage for dBET1, while negative polarity mode offered richer insights. Employing de novo structure determination on 2DMS data from degradation studies yielded crucial insights. In the case of VZ185, various charge states were observed, with [M + 2H]^2+^ revealing fewer moieties than [M + H]^+^ due to charge-related factors. Augmenting structural details through silver adducts suggested both charge-directed and charge-remote fragmentation. This comprehensive investigation identifies frequently dissociated bonds across multiple fragmentation techniques, pinpointing optimal approaches for elucidating PROTAC structures. The findings contribute to advancing our understanding of PROTACs, pivotal for their continued development as promising therapeutic agents.

## 1 Introduction

Proteolysis targeting chimeras (PROTACs) are a new form of drug modality that promote protein degradation, as opposed to inhibition of a protein of interest (POI). These molecules contain a motif that binds to a protein, which is connected by a chemical linker to a ligand that binds to an E3 ligase, forming a ternary complex. This directs the E3 ligase to ubiquitination and subsequent degradation of the POI.^1−11^ PROTACs have several advantages over small-molecule pharmaceuticals that solely inhibit protein activity. PROTACs act substoichiometrically, with one PROTAC molecule facilitating the degradation of multiple copies of the protein, thus requiring a lower dose to achieve targeted protein degradation (TPD).^12^ TPD can overcome resistance mechanisms which are observed for other small-molecule inhibitors.^13^ Proteins with multiple functions downregulate drug activity, as more than one site would need to be inhibited, whereas degrading the POI would resolve this issue.^14^ Furthermore, PROTACs can improve cell selectivity to reduce toxicity, as there are more than 600 E3 ubiquitin ligases in the human proteome.^15^

Although there are numerous advantages to PROTACs, there still lies many challenges in their design. For example, the effectiveness of PROTACs depends on the ligands that bind to the POIs (otherwise known as the warhead), E3 ligases, and the length and chemical properties of the linkers connecting the ligands.^13^ Among the plethora of E3 ligases, only a few have been used for PROTAC technology due to the synthetic opportunities to connect the E3 ligand with a linker^16^ where the length and orientation of the linker can influence the selectivity and formation of a ternary complex.^4^ New developments in PROTAC technologies have been elaborated from their original concept proposed by Crews and Deshaies et al.,^5^ such as the formation of trivalent PROTACs for enhanced binding,^17^ RNA–PROTACs,^18^ and antiviral PROTACs;^19^ additionally, structural information on PROTACs is stored in the open-access database, PROTAC-DB.^20^ Moreover, the binding strengths of ligands, spatial orientation, and cell permeability will impact on the efficacy of PROTACs.^21^ The syntheses of PROTACs are often laborious and largely an empirical process;^22^ therefore, analytical techniques which aid with the structural analysis of each product and byproducts are critical. Nuclear magnetic resonance (NMR)^23,24^ and mass spectrometry (MS)^25,26^ are the most commonly used methods for structure characterization. Sensitivity enhancement and widespread implementation favor the use of MS, particularly in cases where the analysis of fragmentation patterns assists in identifying molecular structure.^24,25^

High mass accuracy and mass resolution is essential for unambiguous determination of elemental formulas for precursors and product ions, enabling low assignment errors (less than 1 ppm) and increased confidence in the assignments.^27^ Ultrahigh resolution accurate-mass mass-spectrometry (UHRAMS), such as Fourier transform-ion cyclotron resonance (FT-ICR) combined with tandem MS, has become a prevalent tool for structure elucidation.^28−31^ Collision-induced dissociation (CID),^32^ infrared multiphoton dissociation (IRMPD),^33,34^ ultraviolet photodissociation (UVPD),^35−38^ and electron-based dissociation (ExD)^39−43^ are all compatible dissociation techniques with FT-ICR, with the former three used in this study.^44^ Comparison of the fragmentation spectra with related structures can help with the de novo identification of PROTACs. Ultrahigh resolving power can resolve isotopic fine structure and closely spaced species in *m*/*z* space, which leads to the accurate determination of elemental compositions following appropriate calibration.^29,31,45^

Typically, CID is the most popular method for the deposition of internal energy in ions.^46,47^ Ions of interest are isolated and subsequently undergo a series of collisions with a neutral gas, which leads to the cleavage of the weakest bonds or follows the lowest energy rearrangement pathways, forming product ions.^48^ Thus, CID may limit the extent of structural characterization achieved by focusing cleavage around the weakest bonds. More energetic dissociation techniques such as electron- and photon-based dissociation can access higher energy pathways, resulting in the observation of different cleavage points.^29,49^

Photodissociation relies on the absorption of photons, exciting the molecule to a higher energy state, which promotes bond cleavage. These photons have a wide span of energies, from low-energy IRMPD (10.6 μm, ∼0.12 eV) to UVPD (157–266 nm, 4.66–7.90 eV).^36,49^ IRMPD fragments ions via adiabatic heating with absorption of IR photons, as reported in a landmark paper by Beauchamp and co-workers.^50,51^ The slow heating followed by fast energy redistribution within IR activated ions results in IRMPD, producing product ions similar to those with low collision energy CID.^49,52^ In contrast, UVPD is a high energy, radiative process that allows access to fragmentation pathways with significantly higher activation threshold energies.^36^ UVPD has previously been exploited to identify the cleavages of proteins,^53^ peptides,^54^ lipids,^55^ nucleic acids,^56^ and small organic molecules.^57^ The ability to dissociate bonds directly and form radical intermediates, while producing a larger array of cross-ring cleavages, provides structural information regarding isomerization around rings thus potentially allowing for the complete structural analysis using UVPD.^57^ UVPD generally yields a richer display of product ions compared to CID for small organic molecules.^29,57^ The success of these photon- and radical-induced dissociation techniques for other classes of organic molecules suggests their use for the enhanced structural characterization of PROTACs.

The use of adducts enables access to different charge-sites, thereby allowing for diverse fragmentation pathways.^58^ Adducts can bind to various functional groups depending on the chemical properties of the molecule of interest.^58^ Some notable trends were observed; for example, sodium adducts often bind to functional groups with lone pairs such as oxygen atoms.^59^ Lithium adducts tend to bind to functional groups that are electron-rich, and these are ketones, amines, and π-bonds in unsaturated compounds.^60^ Silver adducts, can bind to a range of functional groups, including carboxylic acids, thiols, halides, and π-bonds.^33^

Two-dimensional mass spectrometry (2DMS)^34,57,61−69^ is a data-independent analysis (DIA) technique used to analyze complex mixtures without the need for prior chromatographic separation or precursor ion isolation. Product ions are correlated to their corresponding precursor ion through 2D-FFT (fast Fourier transform) data processing.^70^ Previous studies have shown 2DMS applied to small-molecule organics such as agrochemicals for their structural characterization in a complex matrix.^57^

**Scheme 1:**
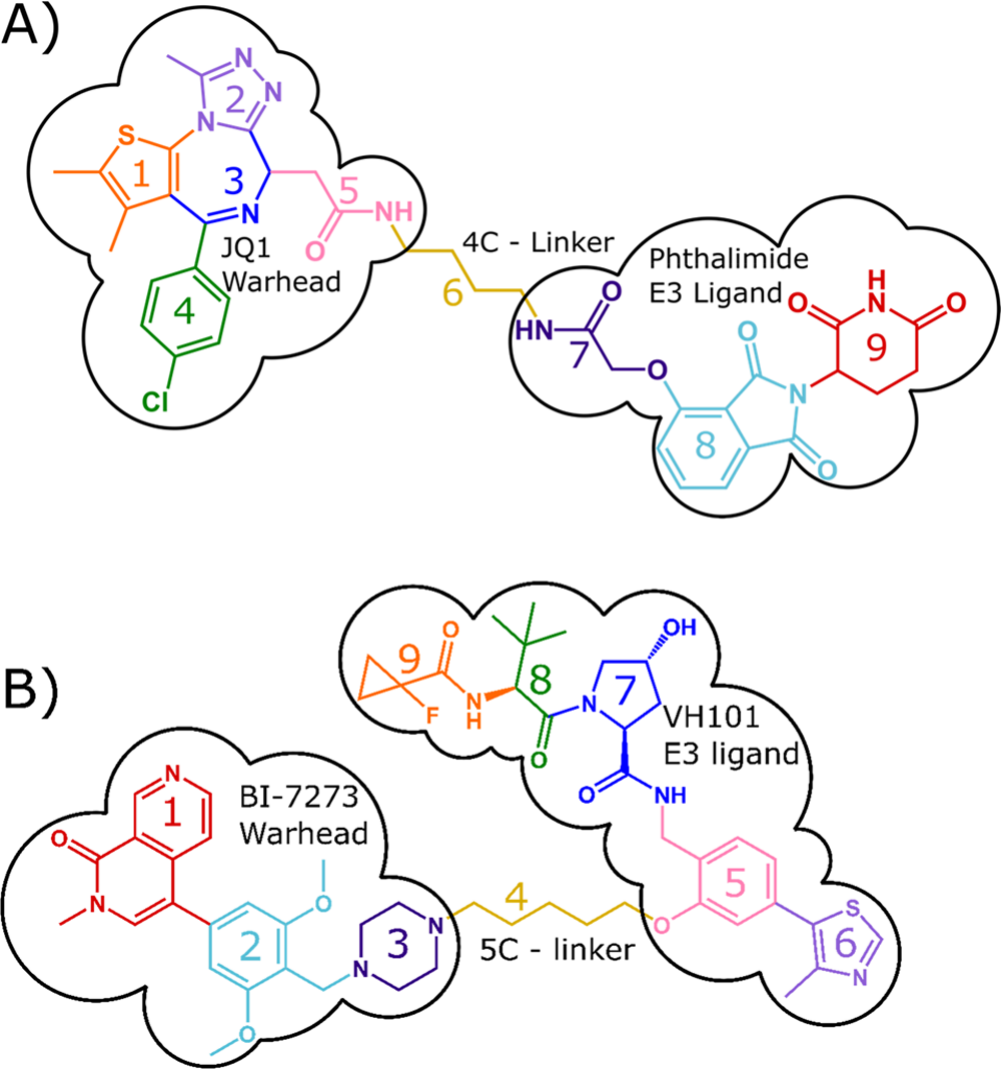
Chemical Structure of (A) dBET1 and (B) VZ185 with Colored Moieties

This study utilizes nanoelectrospray ionization (nESI)-MS coupled with various dissociation techniques to investigate the fragmentation patterns of two PROTACs (dBET1^71^ and VZ185^72^) (Scheme 1) ionized by virtue of a range of cations (H^+^, Na^+^, Li^+^, Ag^+^) and as deprotonated molecules using a 12 T Bruker FT-ICR MS operating at a mass resolution >400 000 full width at half-maximum (fwhm) at *m*/*z* 400. The sub-ppm mass errors coupled with CID MS/MS, IRMPD MS/MS, and UVPD MS/MS allows for confident structural characterization of these molecules. Herein, each fragmentation technique mentioned above, is evaluated for the structural characterization of PROTACs. Moreover, 2DMS has been applied to pharmaceuticals by looking at the possible degradation products formed through hydrolysis and freeze–thaw cycles. Previous MS studies have been performed for the ternary complex by native-MS;^73^ however, in this study we focus solely on the PROTAC and identify the optimum method for complete structure characterization.

## 2 Experimental Section

### Chemicals

Compounds dBET1 (>98%) obtained from Merck (Gillingham, UK) and VZ185 (95%) sourced from Boehringer Ingelheim (Ingelheim, Germany), were dissolved in 50:50 (v/v) acetonitrile (VWR, U.S.A)/water (purified through a Millipore Direct-Q purification system (18.2 Ω; Merck Millipore, MA)). Samples were diluted to a concentration of 1 μM in 50:50 (v/v) acetonitrile/water containing 1% formic acid (Honeywell Fluka, Germany). Adventitious sodium ions from sources such as glass vials formed the sodium species. Lithium and silver adducts were prepared by mixing 1 μM lithium nitrate from Merck (Gillingham, UK) and 1 μM silver nitrate from Fisher Scientific (Loughborough), respectively. dBET1 was freeze–thawed ∼20 times and then hydrolyzed through heating at 90 °C in a hot bath for 168 h.

### Mass Spectrometry

Samples were ionized using a home-built nESI and analyzed with a 12 T Bruker solariX FT-ICR mass spectrometer (Bruker Daltonik, GmbH, Bremen, Germany). A total volume of 10–15 μL was loaded into a glass capillary tip pulled by a Sutter P-97 Flaming/Brown micropipette puller (Sutter Instrument Co., Novato, CA), and the electrical connection was formed using a nichrome wire.^74^ The cationized and deprotonated species were isolated using the quadrupole (isolation window of *m*/*z* 5, *m*/*z* 8, *m*/*z* 10, and *m*/*z* 30 for [M + H]^+^, [M + Li]^+^, [M + Na]^+^, and [M + Ag]^+^, respectively), ensuring the full isotopic envelope was isolated with good precursor intensity.

**Scheme 2:**
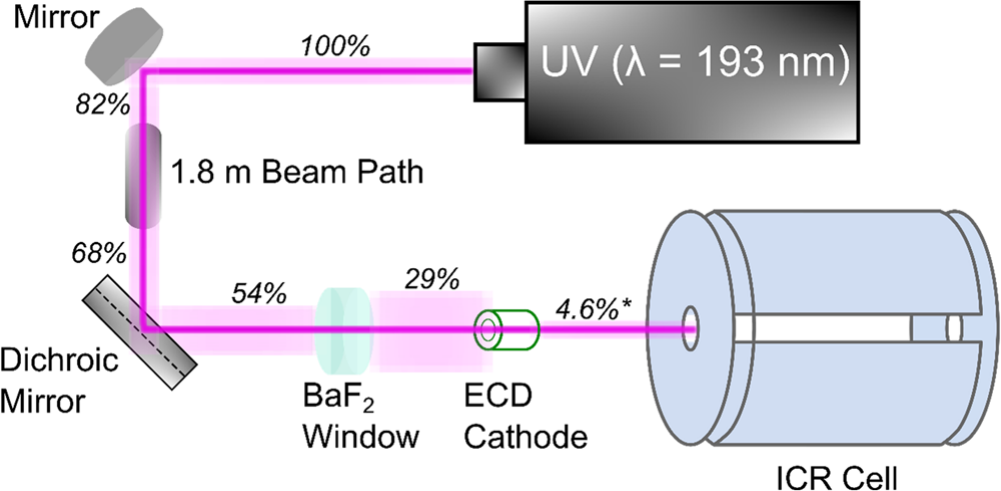
UVPD in Tandem with FT-ICR, Where the Laser Fluence Was Measured Prior to the ECD Cathode (∼3 mm radius) Using a Power Meter and Subsequently Calculated (Marked by an Asterisk) by Accounting for the Beam Divergence over a 3.4 m Distance, before Entering the ICR Cell Laser divergence is indicated by a visible glow.

For CID experiments, the isolated ions were transferred into the collision cell and accumulated for 0.05 to 1.5 s; a compound-optimized collision potential (15–36 V in positive mode and 22 V in negative mode) was applied using argon as the collision gas (∼6.5 × 10^–6^ mbar). The ions were subsequently transferred and detected in the Infinity Cell.^75^ For IRMPD and UVPD experiments, precursor ions were isolated in the quadrupole and subsequently accumulated in the collision cell (0.01 s) before being transferred into the ICR cell. In IRMPD experiments, a 10.6 μm continuous wave 25 W CO_2_ laser (Synrad Inc., Mukilteo, WA) was used at 50–80% of its power output with an irradiation time of 0.25–0.80 s (with ∼25% entering the ICR-cell based on 3.5 mm beam with a divergence of 4 mrad over a flight path of 150 cm). CID and IRMPD experiments were fine-tuned to retain a minimum of 50% of the initial precursor intensity. The UVPD experiment irradiated the trapped ions with 1–3 laser shots of 193 nm (photon = 6.4 eV) from a ArF Excimer laser (ExciStar XS, Coherent) with a pulse energy of 4–5 mJ (measured at the laser exit aperture) and UV shot duration of 7–10 ns. Approximately 4.6% of the 193 nm UVPD laser fluence was found to enter the ICR cell, as described in Scheme 2. A total of 100 scans was accumulated for each fragmentation method and averaged to achieve desirable signal-to-noise ratios (S/N). All data were acquired with a 4 M (2^22^, 32-bit), 1.12 s transient, average resolving power >400 000 (fwhm) at *m*/*z* 400. All spectra were processed and analyzed using Bruker DataAnalysis 5.0 software (Bruker, Bremen, Germany). The 2DMS data was processed by SPIKE^70^ and analyzed using an in-house LabView based program, T2D. All the spectra were analyzed with a similar workflow to the method described by Marzullo et al.^29^

## 3 Results and Discussion

### 3.1 Introduction to Dumbbell Plots

**Scheme 3:**
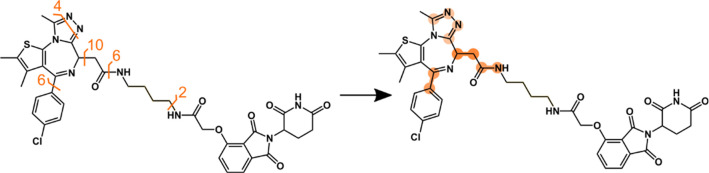
Dumbbell Plots Effectively Emphasize the Most Frequent Cleavage Sites. In These Plots, the Numbers Indicate the Frequency of Cleavages, While the Color Intensity on the Right Corresponds to the Prominence of These Cleavages Relative to Others It’s important to note that only the top three cleavage events are highlighted to avoid congestion.

The intricate fragmentation patterns of the studied PROTACs often lead to congested and complex spectra. To address this, a dumbbell plot is introduced, highlighting bonds with the highest number of cleavages. The intensity of color saturation in the plot signifies the extent of fragmentation, making the interpretation clearer. An illustrative instance of this plot can be found in Scheme 3.

### 3.2 Structural Features of dBET1 and VZ185

dBET1 is a PROTAC composed of a phthalimide moiety attached to a glutarimide ring, which is a ligand known to bind to E3 ubiquitin ligase cereblon^1,76^ linked to a selective bromodomain and extraterminal (BET) protein inhibitor, JQ1 (see Scheme 1),^77^ which is formed by the chemical substitutions of the carboxyl group on JQ1 and the aryl ring of thalidomide.^71^ VZ185 (994.48 g mol^–1^) is larger than dBET1 (784.22 g mol^–1^) and consists of a different chemical composition, namely a BI-7273 warhead and VH101 E3 ligand (see Scheme 1, Table 1). dBET1 is observed in both polarities albeit with a single charge state, while VZ185 does not exhibit a deprotonated form due to its chemistry, instead showing multiple charge states. A summary for each of the bond cleavages for the moieties of dBET1 and VZ185 is described in Scheme 1 and Table 1. Cleavage assignments are color coded based on the moieties in Scheme 1 and grouped together by their adducts and deprotonated form. The molecule is characterized in the cationized and deprotonated forms using CID, IRMPD, and UVPD, where the peaks assigned to the molecule are marked by blue, red, and purple circles, respectively.

**Table 1:** List of Moieties for PROTACs dBET1 and VZ185 That Correspond to the Labels in Scheme 1

moiety	dBET1	VZ185
1	dimethylthiophene	2-methyl-2,7-naphthyridin-1(2*H*)-one
2	3-methyl-1,2,4-triazole	2,6-dimethoxytoluene
3	*N*-methylethanimine	piperazine
4	chlorobenzene	pentane
5	acetylamide	2-methylphenol
6	butane	4-methylthiazole
7	glycolamide	1-formyl-4-hydroxyprolinamide
8	phthalimide	3,3-dimethylbutanal
9	glutarimide	1-fluorocyclopropanecarboxamide

### 3.3 Structural Characterization of dBET1 and VZ185 with a Hydrogen Adduct

**Figure 1:**
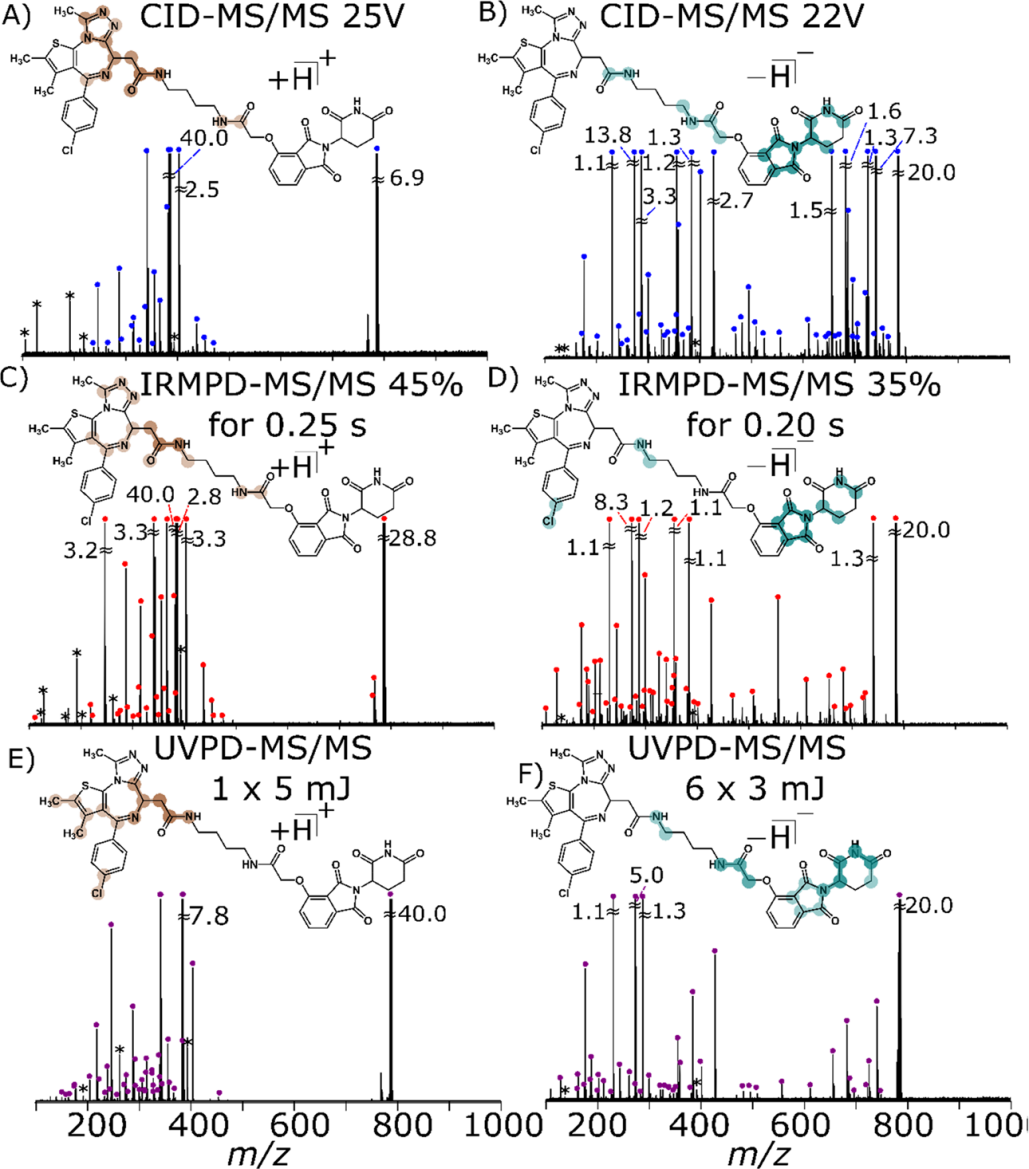
Fragmentation of dBET1 using CID-MS/MS at (A) [M + H]^+^ (25 V) and (B) [M – H]^−^ (22 V); UVPD-MS/MS at (C)) [M + H]^+^ (45% for 0.25 s) and (D) [M – H]^−^ (35% for 0.2 s); and UVPD-MS/MS at (E)) [M + H]^+^ (one shot of 5 mJ) and (F) [M – H]^−^ (six shots of 3 mJ). Dumbbell plots were overlaid, representing the most prominent cleavages as described in Supporting Information Figure S16.

Typically, the prevalent form of fragmentation for these molecules depends on whether they are protonated or deprotonated, which is often influenced by the solvent used. For dBET1, considering its ability to exist in both polarities, the impact of polarity variation across different fragmentation methods is illustrated in Figure 1.

Different moieties can be probed depending on the polarity of the analyte ion; cations display fragmentation of the acetylamide and triazole moieties, while anions produced cleavages around the phthalimide and glutarimide rings (Table 1, Figure 1). The [M – H]^−^ shows fragmentation to be concentrated around the E3 ligase (Scheme 1), demonstrated by the shift in dumbbells in Figure 1 A to B, C to D, and E to F, which implies a potential deprotonation site assuming a charge-direct fragmentation model. The change of charge sites can be observed as the most intense fragment ion shifts from the acetylamide group in the protonated form to the glutarimide moiety in the deprotonated form (Supporting Information Figure S17). Full characterization of the deprotonated dBET1 and its isomers was achieved with CID as the fragmentation technique, which can be seen in Figure 1B and Supporting Information Figure S5 and Table S13. Moreover, CID and IRMPD both display extensive cross-ring cleavages around the chlorobenzene moiety, which can help identify isomers of the JQ1 warhead ligand (see Table 1, Scheme 1, and Supporting Information Figures S5 and S10, also see Supporting Information Tables S13 and S14).

A key difference between positive and negative mode is the extensive fragmentation observed for CID (see Supporting Information Figures S1 and S5). For example, CID demonstrates extensive fragmentation across the aromatic rings, which could be aided by the formation of stable product ions formed by dissociation of the deprotonated molecule, with similar fragmentation patterns seen using IRMPD-MS/MS. Cross-ring cleavages are atypical of CID;^29,78^ however, sub-ppm assignment can characterize the extensive aromatic ring cleavages in negative mode, which could be attributed to the charge-migration fragmentation, seen with loss of a CO,^79^ or retro Diels–Alder reaction.^80^ However, cleavages across rings decrease when using UVPD, which suggest that these ring cleavages occur through vibrational pathways, albeit all moieties can still be identified in negative mode UVPD (see Supporting Information Figure S15 and Table S15). Complete structural characterization was achieved in the anionic form, even in the presence of acid, as detailed in section 3.6.

**Figure 2:**
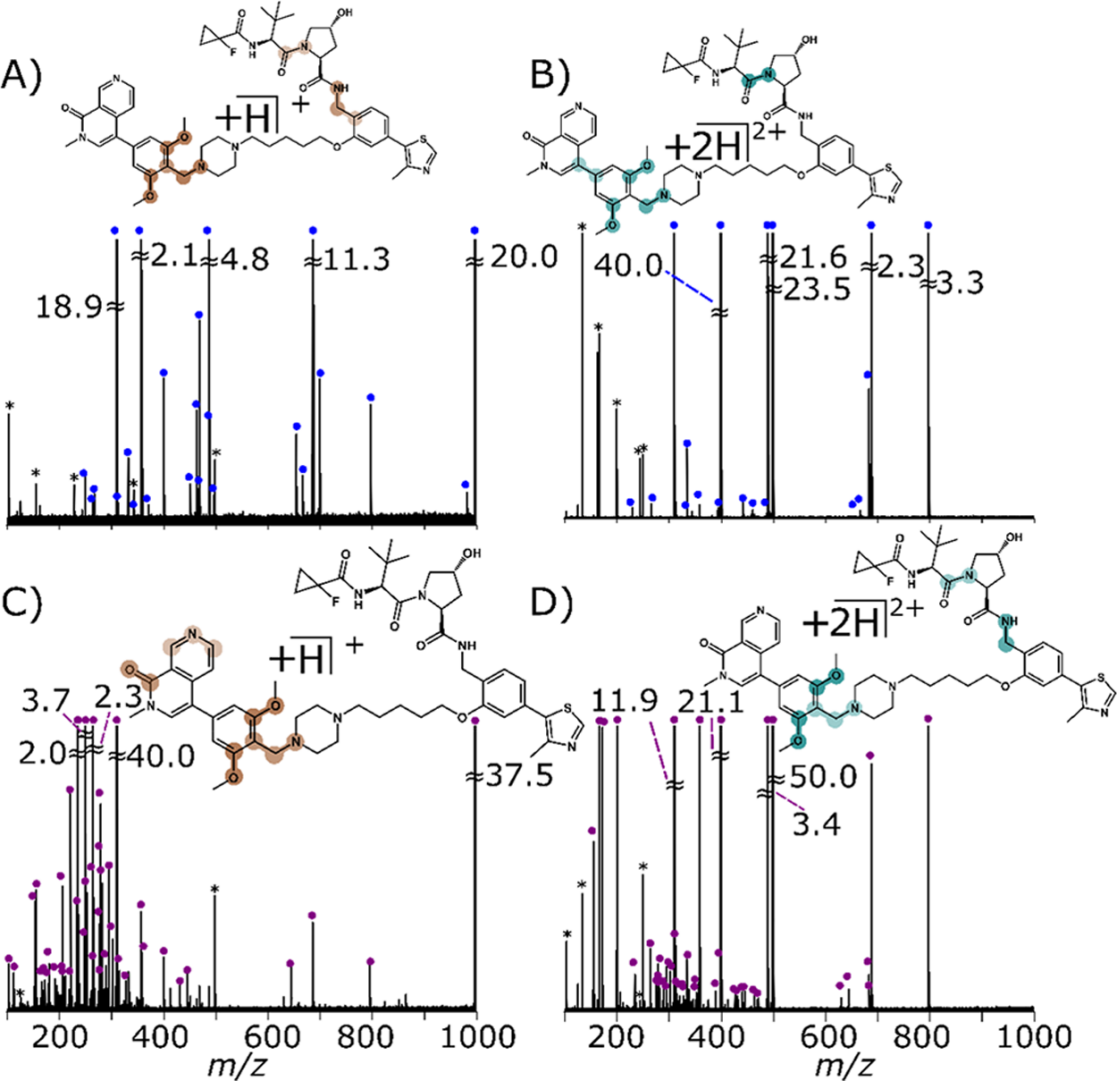
Fragmentation of VZ185 by CID-MS/MS of (A) [M + H]^+^ at 34.5 V and (B) [M + 2H]^2+^ at 9.2 V, and UVPD-MS/MS of (C) [M + H]^+^ with five shots of 3.3 mJ and (D) [M + 2H]^2+^ with one shot of 2.5 mJ. Dumbbell plots were overlaid, representing the most abundant cleavages as described in Supporting Information Figure S38.

In situations where negative polarity is not applicable, it becomes necessary to explore positive polarity. This scenario is exemplified with VZ185, where the investigation of its protonated form is depicted in Figure 2.

Previously, for biomolecules, sequence coverage in CID was found to be dependent on the precursor ion charge state, with higher charged species displaying more fragmentation than lower charge state.^81^ PROTACs displayed similar fragmentation patterns across both charge states, with the higher charge state requiring lower collision energies and UVPD photon energies (three shots of 5 mJ for [M + H]^+^ and one shot of 3 mJ for [M + 2H]^2+^), consistent with the mobile proton model described by Summerfield and Gaskell,^82^ which suggests that the higher charge states weaken the bonds without adding any new structural information.

When contrasted with CID-MS/MS (Figure 2A [M + H]^+^, Figure 2B [M + 2H]^2+^, and Supporting Information Figures S20 and S24) alone, the UVPD-MS/MS spectra (Figure 2C [M + H]^+^, Figure 2D [M + 2H]^2+^, and Supporting Information Figures S29 and S33) unveiled a 2-fold increase in structural moieties, offering a higher level of structural information. The comparison of structural characterization is demonstrated in Supporting Information Figure S35 through the number of peaks, cleavages, and the corresponding moiety coverage for both charge states using CID and UVPD. Lower cleavage coverage when using CID alone could be attributed to the stable fragment ions formed when bonds are cleaved by buildup of internal energy. Low-energy fragmentation occurs by virtue of charge-directed fragmentation, where the yield and diversity depend on the location of the proton on the backbone.^82^

A similar fragmentation pattern was observed when using CID across both charge-states, which changed substantially for UVPD, where the [M + H]^+^ significantly increased in the number of peaks and moiety coverage. One key difference is the enhanced fragmentation focused on the naphthalene and methoxytoluene moieties (Table 1, Scheme 1) for [M + H]^+^. The difference in fragmentation pattern coverage of the [M + 2H]^2+^ could be the due different conformations, and this can be measured by ion mobility (such as trapped ion mobility spectrometry, Supporting Information Figure S37, which showed CCS values of 366.7 Å^2^ and 315.2 Å^2^ for [M + 2H]^2+^ and [M + H]^+^, respectively). Alternatively, the elevation of bond cleavages is likely due to a greater proton mobility,^82^ where the higher charge state would have more localized protons due to columbic repulsions, resulting in fewer cleavages as shown in section 3.7. In contrast, CID had the lowest moiety coverage for both charge states (see section 3.7).

By solely analyzing the protonated adducts of the two PROTACs, it becomes evident that the level of fragmentation is inadequate for comprehensive structural characterization. Consequently, there arises a necessity to augment this by employing alternative adducts.

### 3.4 Collision-Induced Dissociation (CID) of dBET1 by Use of Different Adducts

**Figure 3:**
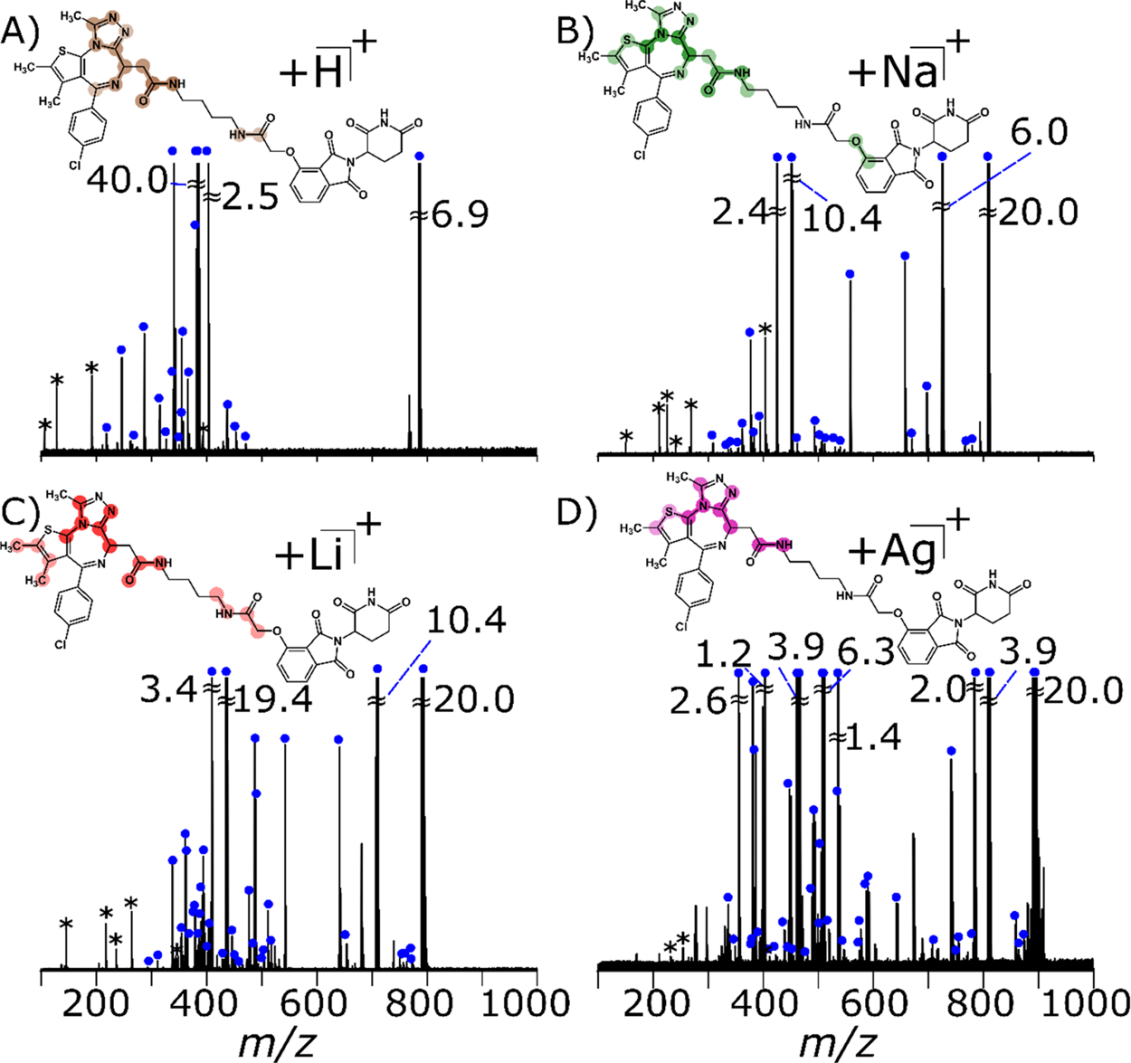
Fragmentation of dBET1 using CID-MS/MS (with dumbbells denoting the most frequent cleavages, ranked by the intensity of the color, see Supporting Information Figure S16) of (A) [M + H]^+^ at 25 V (reproduced from Figure 1), (B) [M + Na]^+^ at 36 V, (C) [M + Li]^+^ at 36 V, and (D) [M + Ag]^+^ at 35.5 V, with the most frequent cleavages highlighted. Assigned peaks are noted with blue circles and noise/harmonics by asterisks.

Using CID, fragmentation of dBET1 was performed on the [M + H]^+^ (25 V), [M + Na]^+^ (36 V), [M + Li]^+^ (36 V), and [M + Ag]^+^ (35.5 V), see Figure 3. More specifically, a high intensity product ion was observed across the amide bond (within the acetylamide group, Table 1) adjacent to the JQ1 ligand^71^ for all adducts in positive mode, denoted by a C1 cleavage (H^+^: −0.243 ppm, Na^+^: 0.177 ppm, Li^+^: 0.179 ppm, and Ag^+^: 0.041 ppm). For the protonated species, the C1 cleavage was the base peak, which can subsequently fragment providing the peaks observed in Figure 3A (Supporting Information Table S1). Intensity of the C1 fragment could be due to the conjugation with the ethylamine chlorobenzene moiety (Table 1). Certain adducts provided more structural information about dBET1 with the order being Li^+^ > Ag^+^ > Na^+^ > H^+^.

In Figure 3, parts of the molecule covered by a dumbbell illustrate the bulk of the cleavages, where the weighting of the color refers to the highest number of cleavage(s). For example, in each quadrant, there is an intense dumbbell around the amide group, which indicates that several fragments are centered around that bond. The position of the dumbbell changes for each cation, thereby suggesting that the location of cleavages is dependent on the choice of cation, which could be due to cleavages caused by different charge sites or charge-remote fragmentation.^79,83^ Comparison of the [M + H]^+^ MS/MS spectrum (Figure 3A) against the [M + Na]^+^ MS/MS spectrum (Figure 3B) displayed additional bond dissociations within the glycolamide and thiophene moiety (Table 1). An example is cleavage C9 (see Supporting Information Figure S2 and Table S7) (Na^+^: 0.006 ppm) and C10 (H^+^: −0.243 ppm, Na^+^: 0.177 ppm), which targets either side of the oxygen atom bonding to the phthalimide moiety (Table 1, Scheme 1). However, additional cleavages were seen within the dimethylthiophene moiety around the sulfur atom, C29 (also see Supporting Information Figure S2 and Table S7) (Na^+^: −0.280), which results in bond dissociation across two bonds within the ring.

Moreover, enhanced structural characterization was observed for the [M + Li]^+^ (see Figure 3C and Supporting Information Table S4). In particular, there was a greater dispersity of dumbbells across the molecule, which suggests that the lithium charge sites are more distributed across the molecule by targeting more bonds. Cleavages were mainly focused on the triazole, acetylamide, glycolamide, and thiophene moieties (Table 1). In this case, more frequent methyl losses were observed, especially around the thiophene group. Full backbone cleavage of the butane group was observed for the lithium precursor ion, see Supporting Information Figure S3.

Finally, the use of a silver adduct allowed for the characterization of the dimethylthiophene substructure, especially around the double bond, which was not seen in the [M + H]^+^ spectrum (see Supporting Information Figure S4 and Table S10). However, the [M + Ag]^+^ spectrum does not aid in the structural characterization as much as other adducts, such as [M + Na]^+^ or [M + Li]^+^. However, more peaks were observed in the [M + Ag]^+^ spectrum than in the [M + Na]^+^ spectrum, which can be attributed to internal fragments that do not produce any new structural information, and the increased number of peaks complicates the mass spectra. However, limited structural information was available about the E3 ligase using CID in the cationized form, which is likely due to the charge sites.^83^

### 3.5 Infrared Multiphoton Dissociation (IRMPD) of dBET1 and Its Adducts

**Figure 4:**
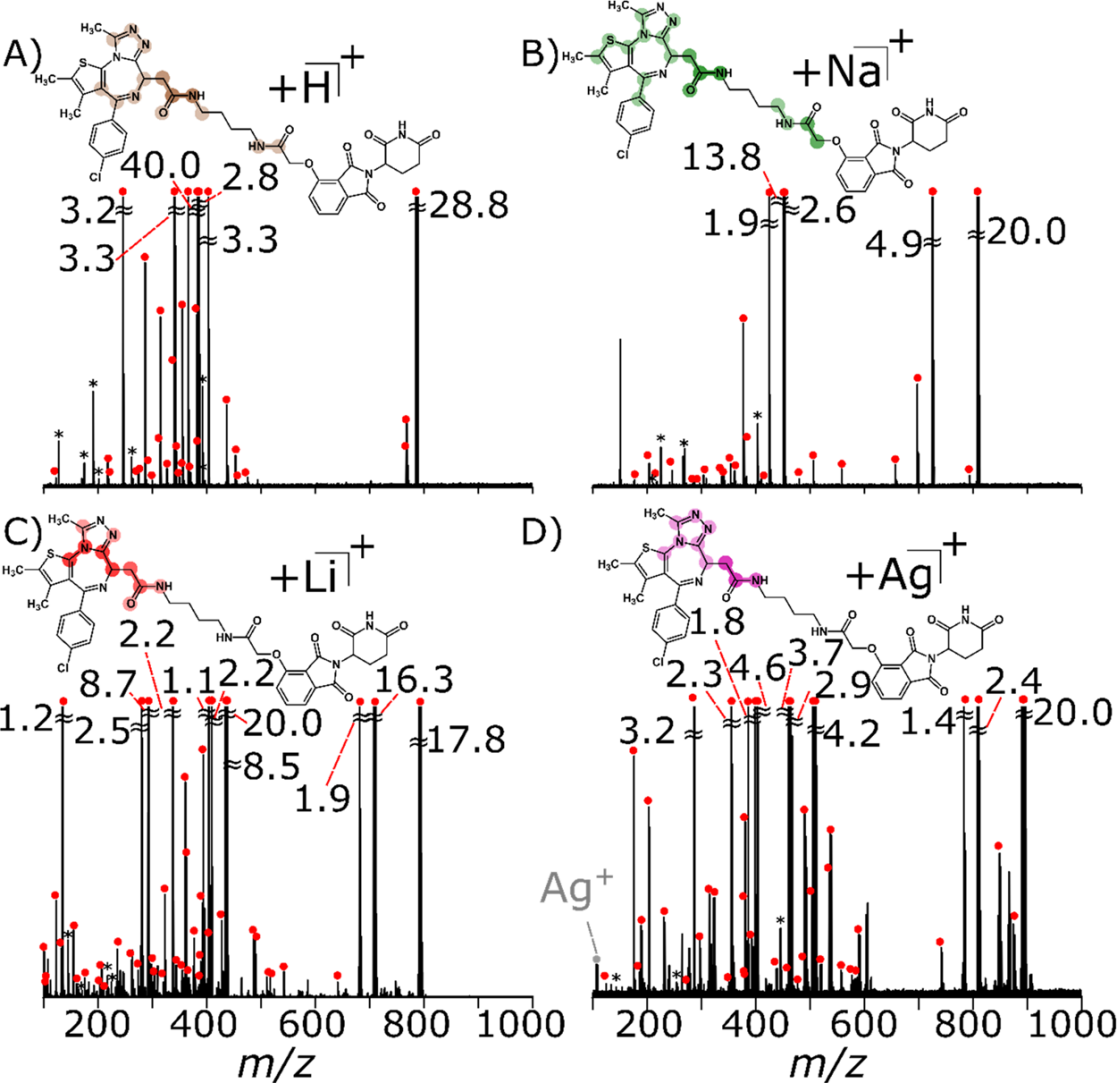
Fragmentation of dBET1 using IRMPD-MS/MS of (A) [M + H]^+^ at 45% for 0.25 s (reproduced from Figure 1), (B) [M + Na]^+^ at 57.5% for 1.0 s, (C) [M + Li]^+^ at 70% for 1.0 s, and (D) [M + Ag]^+^ at 80% for 1.0 s, with the most frequent cleavage points highlighted (Supporting Information Figure S16). Assigned peaks are noted with red circles and noise/harmonics by asterisks.

IRMPD of the protonated species displayed similar structural information to that obtained by CID (Figure 4A, Supporting Information Table S2), albeit with increased complexity due to the secondary fragments occurring through IR activation.^76^ The number of dumbbells increased from Figure 3A to Figure 4A, which suggests that IRMPD helps to further elucidate the structure of the [M + H]^+^. However, one notable difference is the cleavage of the chlorine from the chlorobenzene, which occurs in CID, but was not observed with IRMPD, see Supporting Information Table S2. In general, IRMPD provided a greater moiety coverage ∼40% compared to CID, as well as a new cleavage in between the phthalimide and glutarimide moieties (Table 1), denoted by C99, see Supporting Information Figure S6.

Different ionic forms of the molecule fragmented with adiabatic heating of IRMPD gives rise to complementarity bond cleavages, which becomes prominent for the [M + Na]^+^ species. The bond cleavages are now focused around the chlorobenzene moiety (Table 1) as shown by a new dumbbell in Figure 4B. Additional cross-ring cleavages were observed around the phthalimide and glutarimide moieties such as C90 and C97′ (see Supporting Information Figure S7 and Table S8), which were not observed with [M + H]^+^ ion or solely [M + Na]^+^ species by using CID.

IRMPD was shown to favor cleavages around the triazole and acetylamide moieties for the [M + Li]^+^ and [M + Ag]^+^ species, which can be seen by the more intense dumbbells in Figure 4C,D. Further, there were an abundance of peaks, albeit in low intensity, which enabled characterization of all nine moieties for the [M + Li]^+^ species (Supporting Information Figure S7 and Table S5). Most importantly, both lithium and silver adducts now demonstrate cleavages within the phthalimide conjugate (Table 1), of the E3 ligase, which was not previously seen with CID or with [M + H]^+^, as shown in Supporting Information Figures S8 and S9 (Supporting Information Tables S5 and S11). Although both IRMPD and CID lead to cleavages via the lowest energy rearrangement pathways, this significant difference between the two techniques is untypical and is suggestive that the adiabatic heating coupled to cations that bind to different parts of the molecule leads to complementary fragmentation.

### 3.6 Ultraviolet Photodissociation (UVPD) of dBET1 and Its Adducts

**Figure 5:**
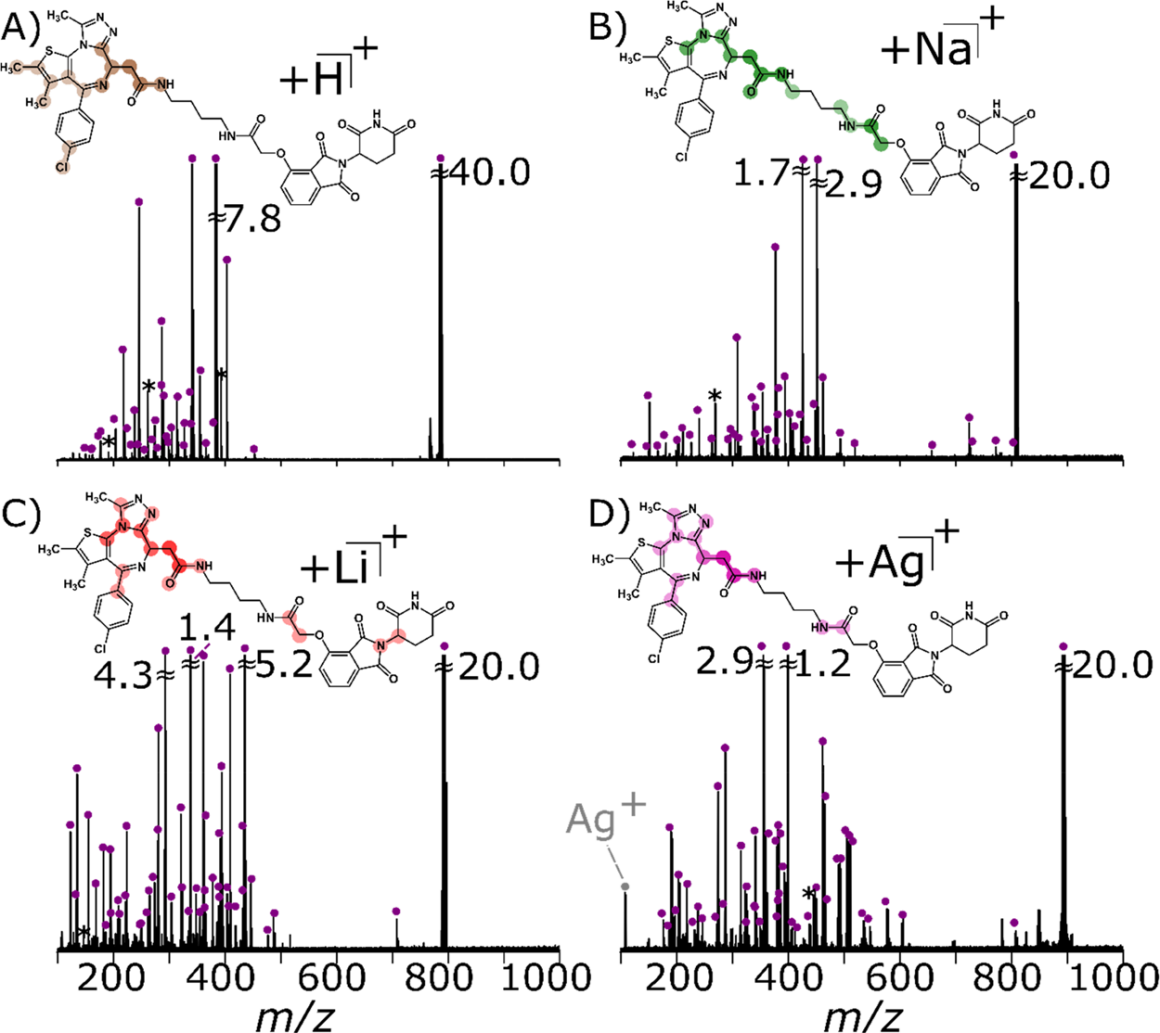
Fragmentation of dBET1 using UVPD-MS/MS (λ = 193 nm) of (A) [M + H]^+^at one shot of 5 mJ (reproduced from Figure 1), (B) [M + Na]^+^ at eight shots of 3.6 mJ, (C) [M + Li]^+^ at five shots of 3.4 mJ, and (D) [M + Ag]^+^ at three shots of 3.5 mJ, with the most frequently observed cleavages highlighted (Supporting Information Figure S16). Assigned peaks are noted with purple circles and noise/harmonics by asterisks.

Similar to IRMPD, UVPD causes dissociation via absorption of a photon by an IR-active or UV-active functional group. However, UVPD allows access to higher energy fragmentation through different pathways such as electronic excited state and radical states, which cannot be achieved by CID or IRMPD. Prominent differences in fragmentation patterns for the [M + H]^+^ can be observed (Supporting Information Figure S11 and Table S3), especially with the enhanced backbone cleavage around the chlorobenzene moiety (Table 1), as shown by the new dumbbells in Figure 5A. In addition, UVPD-MS/MS of [M + Na]^+^ significantly increased cleavage (Supporting Information Figure S12 and Table S9) around the linker, which allowed for full characterization, previously not seen for IRMPD or CID, marked by heavily weighted dumbbells around the acetylamide and glycolamide moieties (Table 1), see Figure 5B, illustrating that the cleavages that occur most frequently.

The UVPD-MS/MS [M + Li]^+^ displayed substantial increase in the number of peaks, albeit with low ion intensity. The cleavages observed were more dispersed across the molecule as shown by the dumbbell plot in in Figure 5C. Additionally, there was an increase in cleavages around the phthalimide moiety (Table 1), which was difficult to fragment for the protonated molecule. Thus, the lithium adducts allowed for characterization of new bonds, previously not seen with the protonated form, and allowed for characterization of all nine moieties (see Table 1, Supporting Information Figure S13 and Table S6).

UVPD-MS/MS of [M + Ag]^+^ species enhanced dissociation around the glycolamide and chlorobenzene moieties, see Figure 5D. Furthermore, full characterization of the four-carbon linker, phthalidomide conjugate, and the JQ1 ligand (Table 1) was achieved (Supporting Information Figure S14 and Table S12). Prominent fragmentation across the thiophene moiety (Table 1) was observed, suggesting that the silver charge site may be in the vicinity to the sulfur- and/or nitrogen-containing rings assuming charge-direct fragmentation.

### 3.7 Overview of dBET1

**Figure 6:**
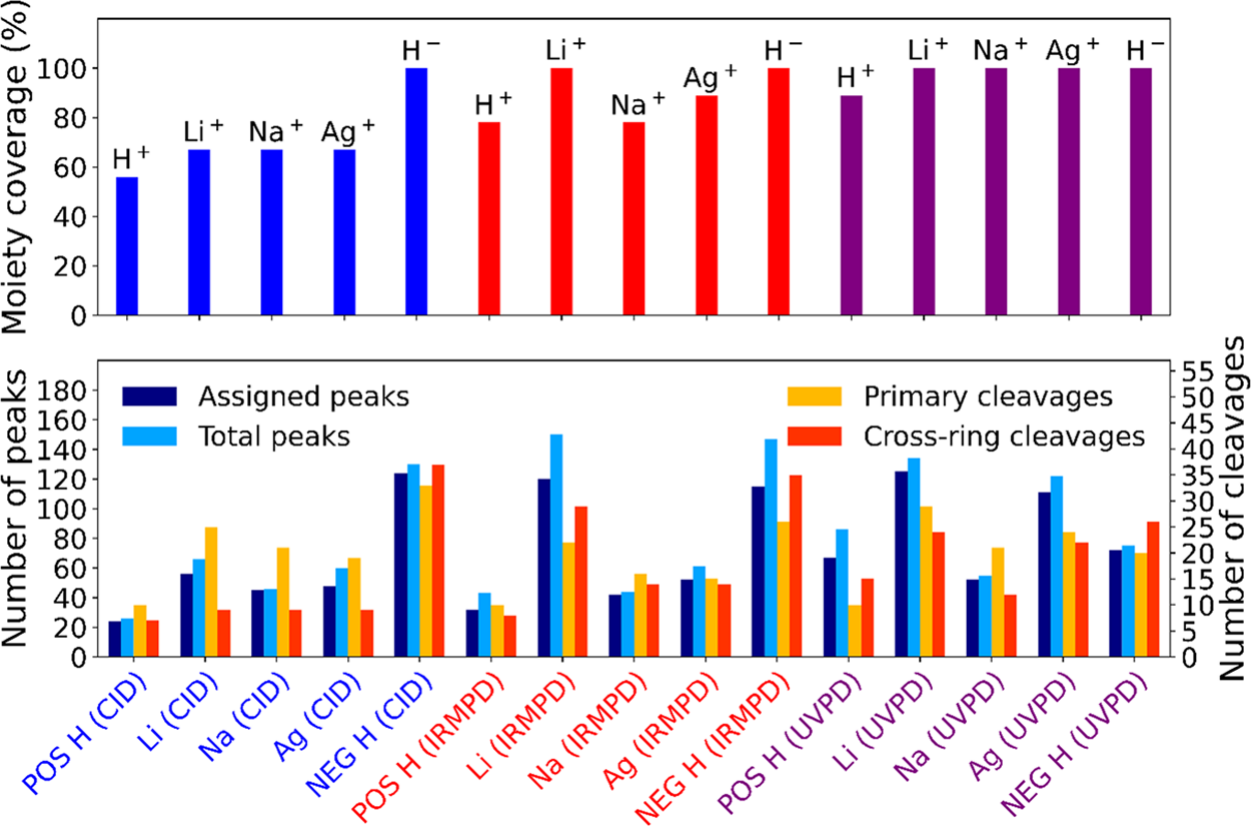
Comparison of each fragmentation technique used to dissociate dBET1, CID-MS/MS (blue), IRMPD-MS/MS (red), and UVPD-MS/MS (purple) against the adducts (H^+^, Li^+^, Na^+^, Ag^+^) and deprotonated molecule (H^–^), with the total number of peak clusters (including isotope patterns) on the left and primary/cross-ring cleavages on the right.

The comparison of the different fragmentation techniques (which include CID (blue), IRMPD (red), and UVPD (purple)), adducts, and polarities used to structurally characterize dBET1 is displayed in Figure 6. The total number of peaks is deisotoped by virtue of grouping related isotopes into one cluster. The number of assigned cleavages refers to the peaks which can be correlated to the structure, permitting structural characterization; otherwise, peaks with elemental formula only (see Supporting Information Tables S1–S15) are likely a result of gas-phase rearrangements and secondary cleavages where structural information on the precursor ion is limited if the rearrangement pathway is unknown.^79^ To help elucidate structures further, primary cleavages can be used, which are similar to protein and peptide fragmentation nomenclature, i.e., a/b/c and the complementary pair x/y/z. Here, they are referred to as C1/C2/C3 and as C1′/C2′/C3′ for the complementary ion pair as shown in the cleavage diagrams in Supporting Information Figures S1–S15. Primary cleavages allow for straightforward structure characterization and avoid complexities that arise due to internal fragmentation. However, cross-ring cleavages, which are produced when two bonds in a ring are broken and can lead to loss of aromaticity of aromatic rings, help to characterize ring substituent locations and thus isomers.

In positive-ion mode, the accepted fragmentation pathway for the protonated species is described by the mobile-proton model, where bond cleavages are driven by migration of a proton weakening adjacent bonds.^84^ However, limited structural information is achieved for the protonated form, as described by the smaller moiety coverage in Figure 6, where the moiety coverage refers to the number of moieties in Table 1 (Scheme 1). UVPD was shown to provide the most structural information, defined as the highest moiety coverage, which can be seen across all adducts but is especially prominent for the lithiated molecule. Lithium was previously shown to have an high affinity to keto groups as well as hydroxy groups and olefinic double bonds in steroids.^60^ Utilizing techniques such as UVPD and IRMPD with adducts such as lithium can allow for complementary fragmentation via a charge-site fragmentation, as the keto-group/double bond, which has a strong affinity to lithium, is also IR and UV active. Lithiated species contain the largest number of peaks and assignable cleavages in positive mode as shown in Figure 6; thus, a deep structural characterization of dBET1 and of similar PROTACs in the cationized form can be achieved by the lithium-bound precursor.

Each lithium and silver adduct displays a distinct isotope pattern, characterized by A – 1 for lithium and A + 2 for silver as displayed in Supporting Information Figure S19, which can help identify product ions with the adduct attached, and although the additional peaks can complicate the mass spectra, ultrahigh resolving power (>400 000 at *m*/*z* 400) can resolve closely placed peaks of similar *m*/*z* values. Silver adducts have a high affinity to π-bonds,^85,86^ and this can be seen by cleavage centered around the triazole group (see Figure 3). However, the dispersity of cleavages across the molecule was limited compared to other adducts, which suggest the lack of migration of the silver charge. IRMPD and CID spectra of [M + Ag]^+^ (Figure 3), have limited cleavage across the aromatic rings, which is especially prominent for the chlorobenzene moiety, which suggests the strong silver interaction to the π-bonds of nitrogen atoms shown in Supporting Information Figure S16. One key feature of this interaction allows for the characterization of the 4C-linker, through cleavage of the nitrogen atoms on either side of the chain (see Supporting Information Figure S18 at *m*/*z* 195.0045973), which is commonly referred to as an internal fragment.

Internal fragments can be difficult to characterize, particularly in certain cases where repeating units or sequences are present, such as polymers,^61^ peptides, and proteins.^87,88^ However, characterization of internal fragments, which occurs by subsequent bond cleavage of the product ion resulting in secondary and MS^n^ fragments, for small molecules has been widely used to increase the obtainable structural information on the molecule.^29^ High mass-accuracy allows for the characterization of internal fragments favored for sub-ppm assignment errors; see Supporting Information Tables S1–S15, which shows cleavage assignment of a typical mass accuracy of approximately ±0.1 ppm. Figure 6 shows that at least 60% of the total peaks is due to internal fragments. Usually, PROTACs have a major bond cleavage, such as the cleavage across the acetylamide moiety C1 (see section 3.2), which can sequentially fragment to give internal fragments such as C1C25 (also see Supporting Information Tables S1–S15), which subsequently breaks the bond within the ethylamine moiety (Table 1). Furthermore, cross-ring cleavages are fundamental, as they help in the elucidation of positional isomers around the rings.^29^ For example, extensive cleavage was observed across the chlorobenzene moiety in negative IRMPD (see Supporting Information Figure S10), which identifies the bonding of the substituent to the ethylamine moiety and that the chlorine is in the para-position. Therefore, internal fragments are important for PROTAC characterization and can be characterized with high mass-accuracy mass spectrometers.

Enhanced structural characterization was observed in the deprotonated form for dBET1, Figure 6, which could be driven by stable product ion formation.^89^ A negative charge site was found by studying the fragmentation patterns and observing which bonds cleaved the most while retaining the charge, and this was likely located within the phthalimide and glutarimide moiety (Table 1). Although the charge site most likely occurred within the E3 ligase, fragmentation was seen around the four-carbon linker and JQ1 warhead (see Scheme 1), which is typical of a charge-migration fragmentation.^79^ Further evidence to support the charge-migration fragmentation can be seen in Supporting Information Figure S17, where the most intense cleavage is the primary fragment C9′ (Supporting Information Figure S15), which is not a deprotonation site but can form internal fragments (C95C9′, error: −0.016 ppm, see Supporting Information Figure S5 and Table S13) that also do not have a deprotonation site. Finally, it can be assumed that the cationized forms of the dBET1 followed a charge-retention fragmentation, as product ions were immersed within potential charge sites, whereas the deprotonated form displayed a charge-migration fragmentation, which allowed for the characterization of other moieties not accessed in the cationized form.

### 3.8 Overview of VZ185

Structural characterization of VZ185 depends on using different adducts and fragmentation techniques (see Supporting Information Figures S20–S33 and Tables S17–S30), which follows a general trend of Ag^+^ > H^+^ > Na^+^ > Li^+^ by use of CID (as measured by assigned cleavages) and changed to Ag^+^ > Li^+^ > Na^+^ > H^+^ for both IRMPD and UVPD. An overall comparison of each technique and cation/deprotonated form is shown in Supporting Information Figure S34 for VZ185, similar to the comparison of dBET1 in Figure 6. Furthermore, there was a common cleavage across the methoxy group (C6) for most adducts (excluding Na^+^ CID, Li^+^ CID, see Supporting Information Figure S38), which became prominent in UVPD, where this functional group fragmented the most frequently (Supporting Information Figures S29–S33) and could be due to the resonance stabilization of the aromatic ring. Cross-ring cleavages were observed within the piperazine moiety (Table 1), and the abundance of these may be due to the presence of nitrogen atoms in the ring for a charge-site fragmentation. Fragmentation of [M + Ag]^+^ by UVPD (see Supporting Information Figure S32) demonstrates enhanced cleavage of the piperazine group, in addition to the targeted cleavage of the amide bonds across the PROTAC, further supporting the attachment of the silver adduct to nitrogen π-bonds.^33^ The use of UVPD can create radical product ion species, and this can lead to rearrangements, such as the McLafferty rearrangement (see Supporting Information Figure S36), which results in the bond cleavage of the *tert*-butyl substituent (C38). Moreover, the stabilization of radical product ions can explain the enhanced cleavages across the amide bond between the butanal and hyrdroxyprolinamide (see Table 1 and Supporting Information Figures S29–S31).

Location of the proton can be measured by the most abundant peaks, as they represent the most stable product ions, which were measured to be adjacent to the piperazine and hyrdroxyprolinamide moieties (Scheme 1, Table 1), described by C1/C1′ and C36 in Supporting Information Figure S39 for the [M + H]^+^ and [M + 2H]^2+^, respectively. Thus, the singly charged species displayed elevated moiety coverage, as there are more sites for accessible sites for proton migration adjacent to the piperazine moiety (Scheme 1, Table 1). Interestingly, only UVPD displayed cleavages outside the typical charge sites observed in CID and IRMPD (Supporting Information Figure S39), which could be due to the higher energy of UVPD, allowing for the dissociation of more bonds.

### 3.9 Two-Dimensional Mass Spectrometry (2DMS) of dBET1

**Figure 7:**
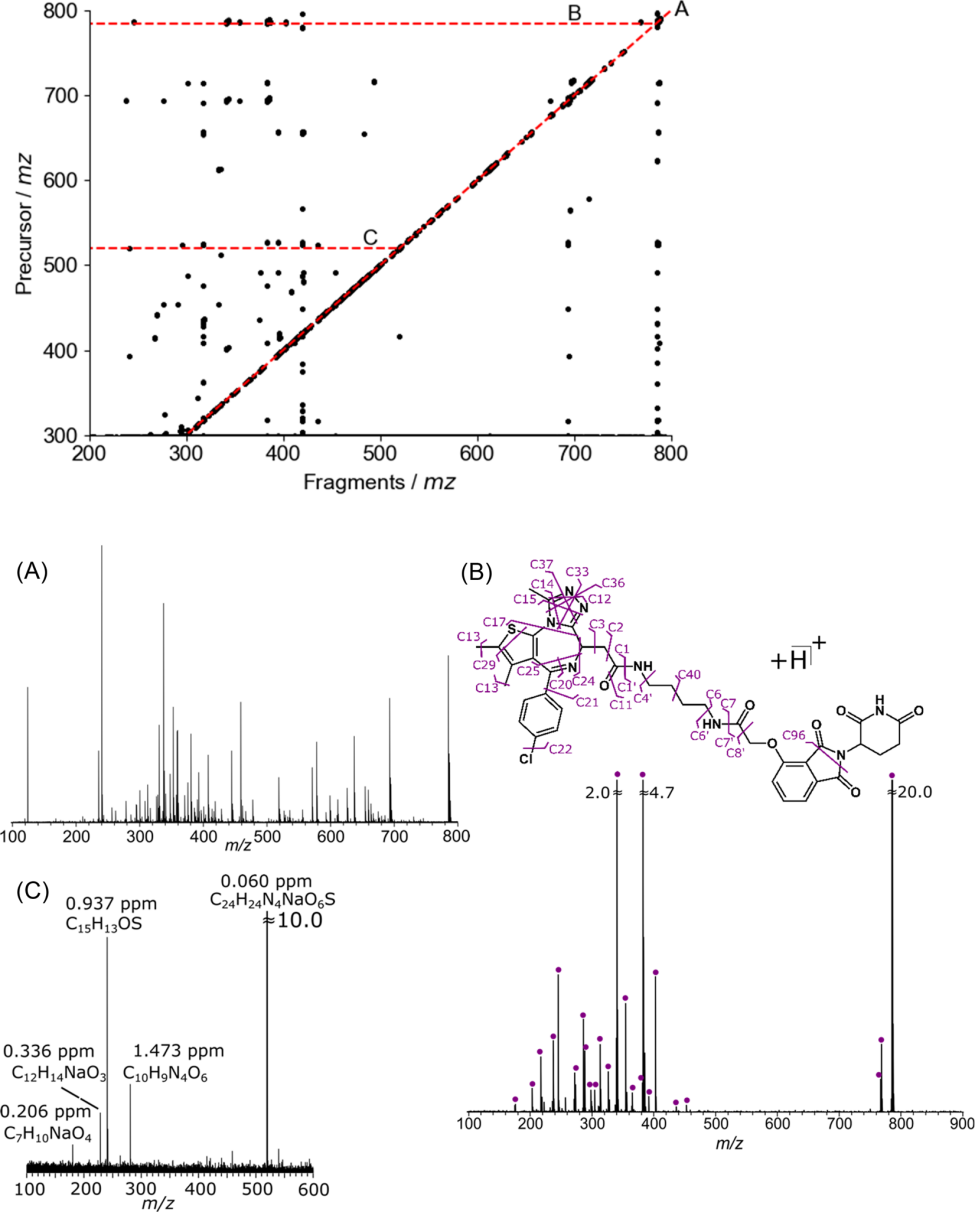
2DMS of PROTAC dBET1, which has been hydrolyzed by heating in a hot bath (at 90 °C) for 168 h, with 2 M data points (*x*-axis) by 2048 (*y*-axis). (A) Autocorrelation line is similar to the standard 1D mass spectrum. (B) Horizontal lines show fragments at the protonated precursor ion of interest [M + H]^+^ with the cleavage assignment. (C) An impurity can be extracted at *m*/*z* 519.130845, see Supporting Information Table S16 and Table 2.

Tandem-MS studies helped deduce UVPD as the most efficient method for structural characterization of dBET1. To fully aid with the characterization of the molecule, an attempt to elucidate the structure of several unknown impurities was made using 2DMS. 2DMS (see section 1) is a DIA technique that can correlate fragments to their respective precursor based on their modulation frequency.^57^ dBET1 was hydrolyzed (see section 2), whereby the products formed a complex mixture of unknowns, and was subsequently characterized using UVPD-2DMS, which can be seen in Figure 7. Precursor ion scan, see Figure 7a, displays the cationized forms of the impurities, similar to a MS scan of the compound. In 2DMS, these precursors are sequentially fragmented, and their respective fragments can be extracted from a horizontal line, known as a product-ion scan, which illustrates similar structural information to the tandem-1D-UVPD spectrum in Figure 5A. Differences between 1D (Figure 5A) and 2DMS (Figure 7B) for the structural characterization of dBET1 can be attributed to the enhanced signal-to-noise obtained in tandem MS, which allows for the detection of smaller peaks lost in the baseline of a 2D spectrum. However, the power of 2DMS remains in the structural characterization of several, or many, precursors simultaneously.

Additional peaks observed in the autocorrelation line can be correlated to dBET1 as degradation products due to the structural similarity, see Table 2. Here, de novo structure determination was performed on a sample of unknowns based on the precursor mass and their corresponding product ions. Most of the structures proposed were concerted around the JQ1 warhead and were confirmed by their tandem-MS patterns. A [M + Na]^+^ precursor was proposed due to the sodium visible in tandem-MS (horizontal line, mass difference of 23 *m*/*z*) in Figure 7. Furthermore, several other peaks were observed, which could be combinations from side-reactions during hydrolysis with the solvent and storage conditions, leading to several unknown impurities, see Supporting Information Table S16. 2DMS has allowed the detection and fragmentation of such impurities where the structure remains unknown due to insufficient fragments in the horizontal line.

**Table 2:**
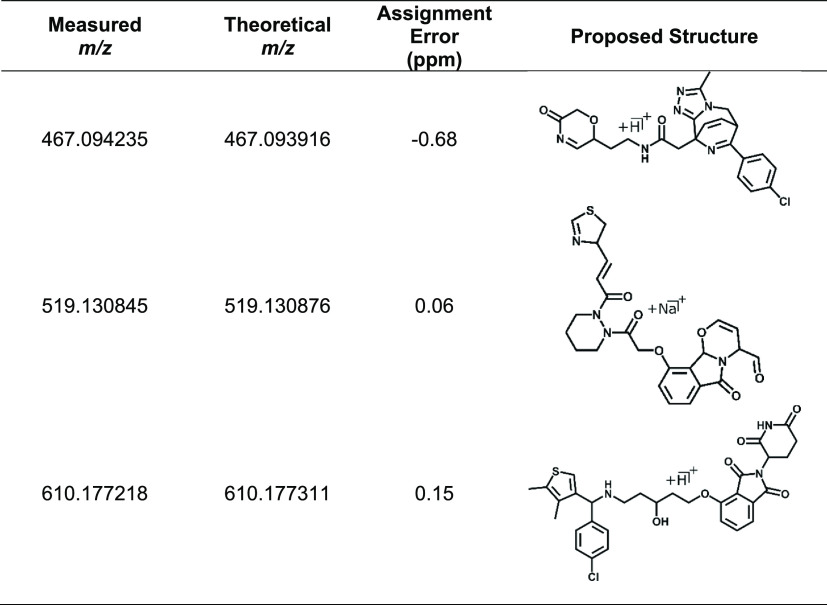
Possible Structures of the Hydrolyzed Compounds^a^

a A full list is shown in Supporting Information Table S16.

Structures assigned by 2DMS can be confirmed by other analytical techniques such as NMR, IR, or UV, after they have been purified by HPLC or a batch sample-extraction process. In 2DMS, they can be analyzed via direct infusion, and the compound’s elemental formula can be assigned to sub-ppm errors (Table 2, Supporting Information Table S16). Most notably, the compound at *m*/*z* 610.177218 shows the loss of a triazole and butane moiety with the degradation reaction, producing an alcohol group in place of the amide within the glycolamide moiety.

## 4 Conclusion and Future Work

PROTACs are versatile molecules that can be ionized into multiple charge states and both polarities. In all cases, internal fragments are common throughout most of the spectra, but the elemental composition of these internal fragments can be characterized by high-mass accuracy instruments. Fragmentation techniques such as CID, IRMPD, and UVPD were found to target different bonds, where UVPD provided the most structural information. Furthermore, using adducts such as sodium, lithium, and silver allowed access to different charge bonding points, and therefore alternative fragmentation pathways can be observed assuming a charge-site fragmentation. Lithium was found to bind strongly to the π-bonds of ketones, whereas silver displayed strong bonding to the nitrogen π-bonds and aromatic rings. Thus, synergic bond cleavage resulted by using techniques such as IRMPD and UVPD, as these π-bonds are IR and UV active. Lithium and silver adducts provided the largest amount of structural information for the smaller molecule (dBET1)^11^ and the larger molecule (VZ185) PROTACs, respectively.

Negative mode ionization was found to produce the most structural information for the case of dBET1 which could be due to the large number of aromatic rings and keto groups which can stabilize the negative charge. Furthermore, PROTACs with multiple charge states, as seen with VZ185, were found to exhibit different conformations depending on their charge state (CCS values of 366.7 Å^2^ and 315.2 Å^2^ for [M + 2H]^2+^ and [M + H]^+^, respectively) and subsequently lower moiety coverage for CID and UVPD than the singly charged ion. Alternative studies can be performed to determine how the conformation of PROTACs is affected by their charge state and the use of adducts. In addition, variations of product ions across different conformers that have been separated by ion mobility can also be studied. 2DMS has shown the ability to dissociate PROTACs, without the need for isolation, leading to a correlated fragment-to-precursor spectrum without any spectral contamination from nearby precursors. In addition, accurate mass measurement allows the elemental formula of impurities to be obtained, and with the complementary fragmentation, the structure of impurities can be identified. De novo structure determination of a sample of unknowns was successfully performed with the help of 2DMS, elucidating structures similar to dBET1. This concept allows for degradation studies to be performed in order to identify PROTACs and their related degradants within one single 2DMS experiment. This work can be improved by exploring the structures produced in other polarities, such as negative mode, or confirming the proposed structures using other analytical techniques, such as chromatography coupled to MS,^90,91^ IR, and NMR.

## Supporting Information Available

MS assignments and fragmentation spectra with cleavage diagrams for both dbet1 and VZ185, with accompanying MS tables (PDF)
