# Supplementary material for: Advancing PROTAC Characterization: Structural Insights through Adducts and Multimodal Tandem-MS Strategies
Source: J Am Soc Mass Spectrom. 2024 Jan 10;35(2):285–99. doi: 10.1021/jasms.3c00342 (PMC10853971; doi:10.1021/jasms.3c00342)
Supplement: Supplementary file 1 — js3c00342_si_001.pdf [file js3c00342_si_001.pdf]

Advancing PROTAC Characterisation: Structural Insights through Adducts and Multimodal Tandem-MS Strategies Supplementary Information

Mohammed Rahman[1,2], Bryan. Marzullo[1,3], Stephen. W. Holman[4], Mark Barrow[1], Andrew. D. Ray[5] and Peter. B. O'Connor[1*]

[1]Department of Chemistry, University of Warwick, Coventry, CV4 7AL, UK

[2]Department of Physics, University of Warwick, Coventry, CV4 7AL, UK

[3]Present address: Metabolite Tracer Analysis Core, University of Birmingham, Birmingham, B15 2TT, UK

[4]Chemical Development, Pharmaceutical Technology & Development, Operations, AstraZeneca, Macclesfield, SK10 4TF, UK

[5]New Modalities and Parenteral Development, Pharmaceutical Technology & Development, Operations, AstraZeneca, Macclesfield, SK10 4TF, UK

*Corresponding authors

Email:

p.oconnor@warwick.ac.uk

The supporting information provides cleavage diagrams, with addional mass spectra for VZ185. MS for the extracted 2DMS rows are presented here as well. Mass tables with cleavage assignment can be found towards the end of the supporting information.

Table of Contents

S1 Structural characterisation of dBET1 by Collision-Induced Dissociation 7
- Figure S1 Cleavage diagram of dBET1 [M+H]+ using CID at 25 V 7
- Figure S2 Cleavage diagram of dBET1 $[M+Na]^+$ using CID at 36 V 7
- Figure S3 Cleavage diagram of dBET1 $[M+Li]^+$ using CID at 36 V.......................7
- Figure S4 Cleavage diagram of dBET1 $[M+Ag]^+$ using CID at 35.5 V 8
- Figure S5 Cleavage diagram of dBET1 $[M–H]^–$ using CID at 22 V 8

S2 Structural characterisation of dBET1 by Infrared Multiphoton Dissociation.........9
- Figure S6 Cleavage diagram of dBET1 $[M+H]^+$ using IRMPD (45% for 0.25 s) ...9
- Figure S7 Cleavage diagram of dBET1 $[M+Na]^+$ using IRMPD (57.5% for 1.0 s) 9
- Figure S8 Cleavage diagram of dBET1 $[M+Li]^+$ using IRMPD (70.0% for 1.0 s) ..9
- Figure S9 Cleavage diagram of dBET1 $[M+Ag]^+$ using IRMPD (80.0% for 1.0 s) 10
- Figure S10 Cleavage diagram of dBET1 $[M–H]^–$ using IRMPD (35% for 0.2 s)..10

S3 Structural characterisation of dBET1 by Ultraviolet Photo-Dissociation 11
- Figure S11 Cleavage diagram of dBET1 $[M+H]^+$ using UVPD (1 shot of 5 mJ)..11

Figure S12 Cleavage diagram of dBET1 [M+Na]$^+$ using UVPD (8 shots of 3.6 mJ) 11
Figure S13 Cleavage diagram of dBET1 [M+Li]$^+$ using UVPD (5 shots of 3.4 mJ) 11
Figure S14 Cleavage diagram of dBET1 [M+Ag]$^+$ using UVPD (3 shots of 3.5 mJ) 12
Figure S15 Cleavage diagram of dBET1 [M–H]$^-$ using UVPD (6 shots of 3 mJ) 12
S4 Summary of cleavages for dBET1 13
Figure S16A Frequency of bond cleavages for dBET1 using CID-MS/MS, IRMPD-MS/MS, and UVPD-MS/MS for each cation and deprotonate form, cleavages are colour coded based on the moieties in Scheme 1 (see main paper). 13
Figure S16B Most intense (relative intensity > 5%) product ions for dBET1 using CID-MS/MS, IRMPD-MS/MS, and UVPD-MS/MS for each cation and deprotonate form, cleavages are colour coded based on the moieties in Scheme 1 (see main paper). 14
S5 Cleavages of dBET1 [M+Ag]$^+$ molecular ion 16
Figure S17 Cleavages observed for dBET1 [M+Ag]$^+$ where the silver adduct was found to still be attached 16
Supplementary Figure S18 Measured (top) and simulated (bottom) isotope pattern of a dBET1 cleavage at C1' ($C_{19}H_{22}N_4O_6Li$), lithium analogue (left); and C2 ($C_{18}H_{17}ClN_4SAg$), silver analogue (right). 17
S6 Structural characterisation of VZ185 by Collision Induced Dissociation 18
Figure S19 Cleavage diagram and dumbbell plot displaying the most common dissociation locations for VZ185 [M+H]$^+$ using CID at 34.5 V 18
Figure S20 Cleavage diagram, dumbbell plot displaying the most common dissociation points, and product ion spectrum for VZ185 [M+Na]$^+$ using CID at 39 V. 19
Figure S21 Cleavage diagram and dumbbell plot displaying the most common dissociation locations for VZ185 [M+Li]$^+$ using CID at 35.5 V. 20
Figure S22 Cleavage diagram and dumbbell plot displaying the most common dissociation locations for VZ185 [M+Ag]$^+$ using CID at 37 V. 21
Figure S23 Cleavage diagram and dumbbell plot displaying the most common dissociation locations for VZ185 [M+2H]$^{2+}$ using CID at 9.2 V. 22
S7 Structural characterisation of VZ185 by Infrared Multiphoton Dissociation 23
Figure S24 Cleavage diagram and dumbbell plot displaying the most common dissociation locations for VZ185 [M+H]$^+$ using IRMPD (35% for 0.25 s). 23
Figure S25 Cleavage diagram and dumbbell plot displaying the most common dissociation locations for VZ185 [M+Na]$^+$ using IRMPD (35% for 0.85 s). 24
Figure S26 Cleavage diagram and dumbbell plot displaying the most common dissociation locations for VZ185 [M+Li]$^+$ using IRMPD (50.0% for 0.85 s). 25
Figure S27 Cleavage diagram and dumbbell plot displaying the most common dissociation locations for VZ185 [M+H]$^+$ using IRMPD (40.0% for 0.85 s). 26
S8 Structural characterisation of VZ185 by Ultraviolet Photo-Dissociation 27

Figure S28 Cleavage diagram and dumbbell plot displaying the most common dissociation locations for VZ185 $[M+H]^+$ using UVPD (5 shots of 3.3 mJ)..........27

Figure S29 Cleavage diagram and dumbbell plot displaying the most common dissociation locations for VZ185 $[M+Na]^+$ using UVPD (5 shots of 3.2 mJ)........28

Figure S30 Cleavage diagram and dumbbell plot displaying the most common dissociation locations for VZ185 $[M+Li]^+$ using UVPD (5 shots of 3 mJ). 29

Figure S31 Cleavage diagram and dumbbell plot displaying the most common dissociation locations for VZ185 $[M+Ag]^+$ using UVPD (5 shots of 3.3 mJ)........30

Figure S32 Cleavage diagram and dumbbell plot displaying the most common dissociation locations for VZ185 $[M+2H]^{2+}$ using UVPD (1 shot of 2.5 mJ). 31

S9 Comparison of VZ185 and its adducts and charge states 32

Figure S33A Comparison of each fragmentation technique, CID (blue), IRMPD (red), and UVPD (purple) against the adducts (H^+, Li^+, Na^+, Ag^+), with the total number of peak clusters (including isotope patterns) on left and direct/cross-ring cleavages on right. 32

Figure S33B Comparison of each fragmentation technique, CID (blue) and UVPD (purple) against the charge states of VZ185 with the total number of peak clusters (including isotope patterns) on left and direct/cross-ring cleavages on right. 33

S10 Rearrangements of VZ185 34

Figure S34 McLafferty Rearrangement of VZ185, which leads to the bond cleavage of tert-butyl substituent (C38)..34

S11 Trapped Ion Mobility mobilograms of VZ185...35

Figure S35 Trapped Ion Mobility (TIMS) spectrum of PROTAC VZ185, displaying the two different mobilities of $[M+H]^+$ (*m/z* 995.485937, extracted mobilogram in blue, CCS: 315.2 Å^2) and $[M+2H]^{2+}$ (*m/z* 498.246607, extracted mobilogram in purple, CCS: 366.7 Å^2)..35

S12 Summary of cleavages for VZ185 36

Figure S36 Frequency of bond cleavages for VZ185 using CID-MS/MS, IRMPD-MS/MS, and UVPD-MS/MS for each cation and deprotonate form, cleavages are colour coded based on the moieties in Scheme 1 (see main paper). 36

Supplementary Figure 37 Relative abundance of ions produced by fragmentation of VZ185 using CID-MS/MS, IRMPD-MS/MS, and UVPD-MS/MS for each cation, displaying fragments with relative intensities > 5%, colors for the cleavages are based on the moieties described in Scheme 1 and Table 1. 37

Supplementary Tables of dBET1 38

Table S1 showing peak list, signal-to-noise ratio (S/N), elemental composition, and assignment with mass errors (ppm) of the $[M+H]^+$ dBET1 compound by CID MS/MS, calibration points are marked by an asterisk (✱). 38

Table S2 showing peak list, signal-to-noise ratio (S/N), elemental composition, and assignment with mass errors (ppm) of the $[M+H]^+$ dBET1 compound by IRMPD MS/MS, calibration points are marked by an asterisk (✱)......................39

Table S3 showing peak list, signal-to-noise ratio (S/N), elemental composition, and assignment with mass errors (ppm) of the $[M+H]^+$ dBET1 compound by UVPD MS/MS, calibration points are marked by an asterisk (✱). 40

Table S4 showing peak list, signal-to-noise ratio (S/N), elemental composition, and assignment with mass errors (ppm) of the $[M+Li]^+$ dBET1 compound by CID MS/MS, calibration points are marked by an asterisk (✱).................................42
Table S5 showing peak list, signal-to-noise ratio (S/N), elemental composition, and assignment with mass errors (ppm) of the $[M+Li]^+$ dBET1 compound by IRMPD MS/MS, calibration points are marked by an asterisk (✱)......................44
Table S6 showing peak list, signal-to-noise ratio (S/N), elemental composition, and assignment with mass errors (ppm) of the $[M+Li]^+$ dBET1 compound by UVPD MS/MS, calibration points are marked by an asterisk (✱).................................47
Table S7 showing peak list, signal-to-noise ratio (S/N), elemental composition, and assignment with mass errors (ppm) of the $[M+Na]^+$ dBET1 compound by CID MS/MS, calibration points are marked by an asterisk (✱).................................50
Table S8 showing peak list, signal-to-noise ratio (S/N), elemental composition, and assignment with mass errors (ppm) of the $[M+Na]^+$ dBET1 compound by IRMPD MS/MS, calibration points are marked by an asterisk (✱)......................51
Table S9 showing peak list, signal-to-noise ratio (S/N), elemental composition, and assignment with mass errors (ppm) of the $[M+Na]^+$ dBET1 compound by UVPD MS/MS, calibration points are marked by an asterisk (✱). 52
Table S10 showing peak list, signal-to-noise ratio (S/N), elemental composition, and assignment with mass errors (ppm) of the $[M+Ag]^+$ dBET1 compound by CID MS/MS, calibration points are marked by an asterisk (✱).................................54
Table S11 showing peak list, signal-to-noise ratio (S/N), elemental composition, and assignment with mass errors (ppm) of the $[M+Ag]^+$ dBET1 compound by IRMPD MS/MS, calibration points are marked by an asterisk (✱)......................56
Table S12 showing peak list, signal-to-noise ratio (S/N), elemental composition, and assignment with mass errors (ppm) of the $[M+Ag]^+$ dBET1 compound by UVPD MS/MS, calibration points are marked by an asterisk (✱). 58
Table S13 showing peak list, signal-to-noise ratio (S/N), elemental composition, and assignment with mass errors (ppm) of the $[M-H]^-$ dBET1 compound by CID MS/MS, calibration points are marked by an asterisk (✱).................................63
Table S14 showing peak list, signal-to-noise ratio (S/N), elemental composition, and assignment with mass errors (ppm) of the $[M-H]^-$ dBET1 compound by IRMPD MS/MS, calibration points are marked by an asterisk (✱).................................66
Table S15 showing peak list, signal-to-noise ratio (S/N), elemental composition, and assignment with mass errors (ppm) of the $[M-H]^-$ dBET1 compound by UVPD MS/MS, calibration points are marked by an asterisk (✱).................................69
Supplementary tables for 2DMS of dBET1...71
Table S16 showing peak list, signal-to-noise ratio (S/N), and possible elemental composition with mass errors (ppm) of the hydrolysed dBET1 compound by 2DMS in tandem with UVPD, calibrated by the precursor at 785.226723 *m/z*. 71
Supplementary Tables of VZ185...73
Table S17 showing peak list, signal-to-noise ratio (S/N), elemental composition, and assignment with mass errors (ppm) of the $[M+H]^+$ VZ185 compound by CID MS/MS, calibration points are marked by an asterisk (✱).................................73

Table S18 showing peak list, signal-to-noise ratio (S/N), elemental composition, and assignment with mass errors (ppm) of the $[M+H]^+$ VZ185 compound by IRMPD MS/MS, calibration points are marked by an asterisk (✱)......................74

Table S19 showing peak list, signal-to-noise ratio (S/N), elemental composition, and assignment with mass errors (ppm) of the $[M+H]^+$ VZ185 compound by UVPD MS/MS, calibration points are marked by an asterisk (✱)..................................76

Table S20 showing peak list, signal-to-noise ratio (S/N), elemental composition, and assignment with mass errors (ppm) of the $[M+Li]^+$ VZ185 compound by CID MS/MS, calibration points are marked by an asterisk (✱)..................................80

Table S21 showing peak list, signal-to-noise ratio (S/N), elemental composition, and assignment with mass errors (ppm) of the $[M+Li]^+$ VZ185 compound by IRMPD MS/MS, calibration points are marked by an asterisk (✱)......................81

Table S22 showing peak list, signal-to-noise ratio (S/N), elemental composition, and assignment with mass errors (ppm) of the $[M+Li]^+$ VZ185 compound by UVPD MS/MS, calibration points are marked by an asterisk (✱)..................................83

Table S23 showing peak list, signal-to-noise ratio (S/N), elemental composition, and assignment with mass errors (ppm) of the $[M+Na]^+$ VZ185 compound by CID MS/MS, calibration points are marked by an asterisk (✱)..................................86

Table S24 showing peak list, signal-to-noise ratio (S/N), elemental composition, and assignment with mass errors (ppm) of the $[M+Na]^+$ VZ185 compound by IRMPD MS/MS, calibration points are marked by an asterisk (✱)......................87

Table S25 showing peak list, signal-to-noise ratio (S/N), elemental composition, and assignment with mass errors (ppm) of the $[M+Na]^+$ VZ185 compound by UVPD MS/MS, calibration points are marked by an asterisk (✱). 89

Table S26 showing peak list, signal-to-noise ratio (S/N), elemental composition, and assignment with mass errors (ppm) of the $[M+Ag]^+$ VZ185 compound by CID MS/MS, calibration points are marked by an asterisk (✱)..................................93

Table S27 showing peak list, signal-to-noise ratio (S/N), elemental composition, and assignment with mass errors (ppm) of the $[M+Ag]^+$ VZ185 compound by IRMPD MS/MS, calibration points are marked by an asterisk (✱)......................95

Table S28 showing peak list, signal-to-noise ratio (S/N), elemental composition, and assignment with mass errors (ppm) of the $[M+Ag]^+$ VZ185 compound by UVPD MS/MS, calibration points are marked by an asterisk (✱). 98

Table S29 showing peak list, signal-to-noise ratio (S/N), elemental composition, and assignment with mass errors (ppm) of the $[M-H]^-$ VZ185 compound by CID MS/MS, calibration points are marked by an asterisk (✱)................................104

Table S30 showing peak list, signal-to-noise ratio (S/N), elemental composition, and assignment with mass errors (ppm) of the $[M-H]^-$ VZ185 compound by UVPD MS/MS, calibration points are marked by an asterisk (✱)................................105

S1 Structural characterisation of dBET1 by Collision-Induced Dissociation

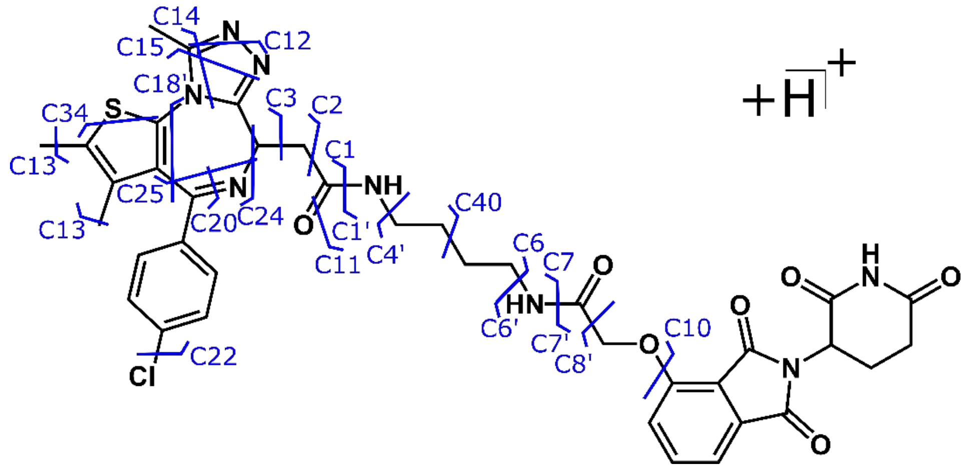

Figure S1 Cleavage diagram of dBET1 [M+H]+ using CID at 25 V

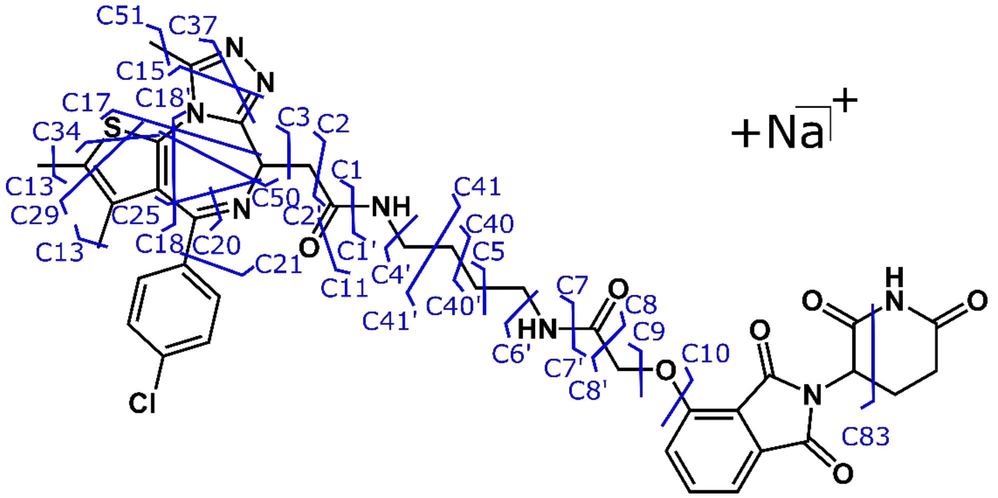

Figure S2 Cleavage diagram of dBET1 [M+Na]+ using CID at 36 V

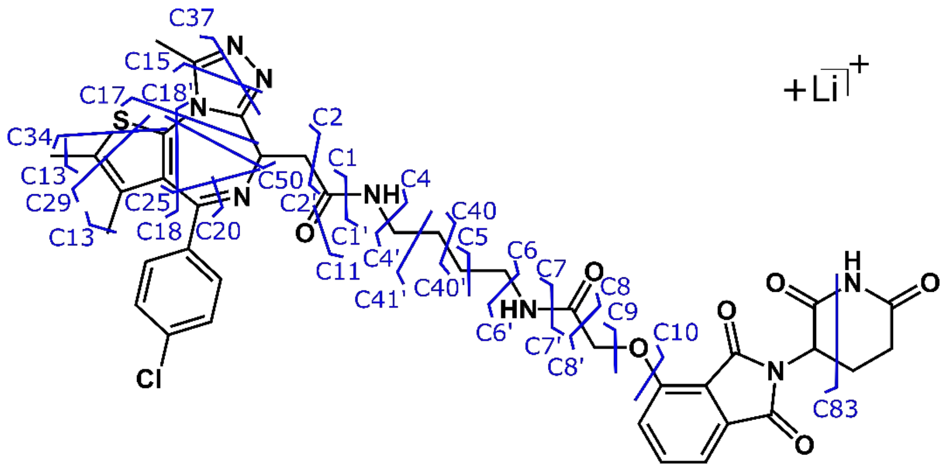

Figure S3 Cleavage diagram of dBET1 [M+Li]+ using CID at 36 V

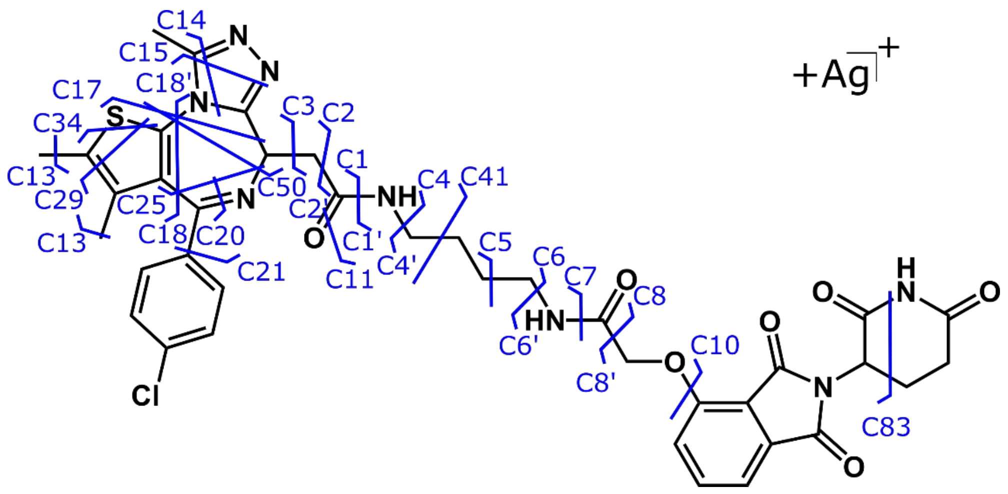

Figure S4 Cleavage diagram of dBET1 [M+Ag]⁺ using CID at 35.5 V

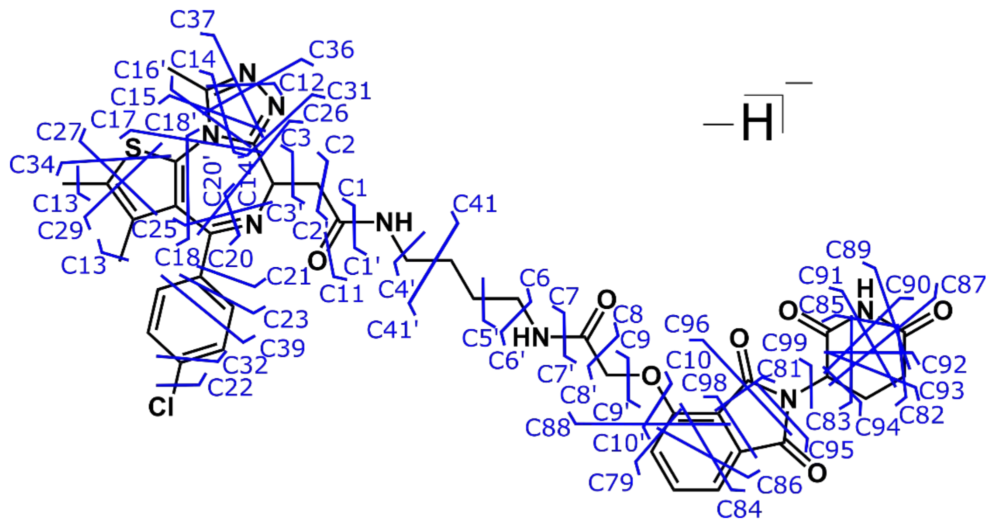

Figure S5 Cleavage diagram of dBET1 [M–H]⁻ using CID at 22 V

S2 Structural characterisation of dBET1 by Infrared Multiphoton Dissociation

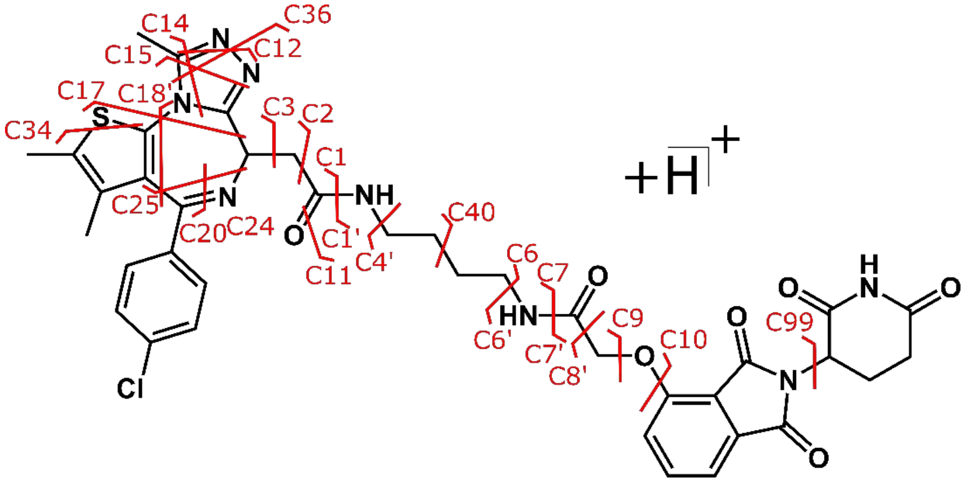

Figure S6 Cleavage diagram of dBET1 [M+H]+ using IRMPD (45% for 0.25 s)

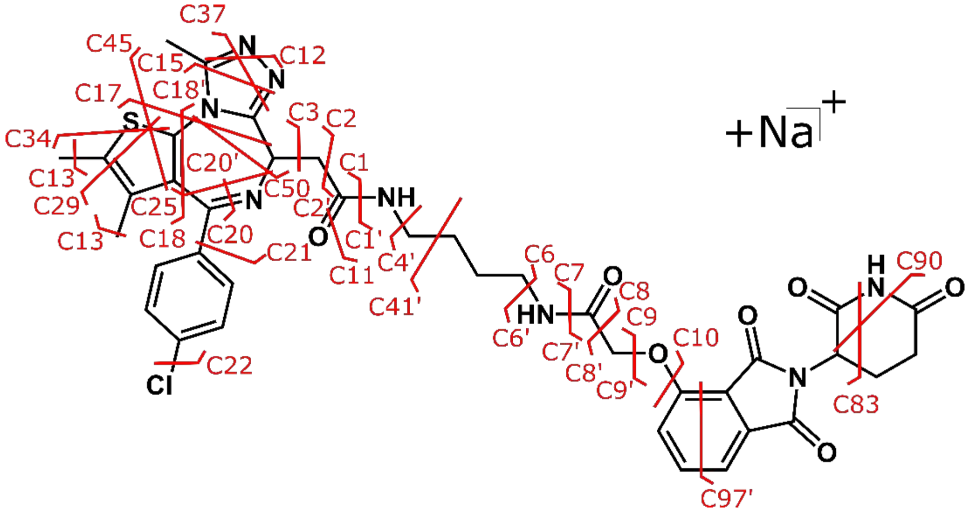

Figure S7 Cleavage diagram of dBET1 [M+Na]+ using IRMPD (57.5% for 1.0 s)

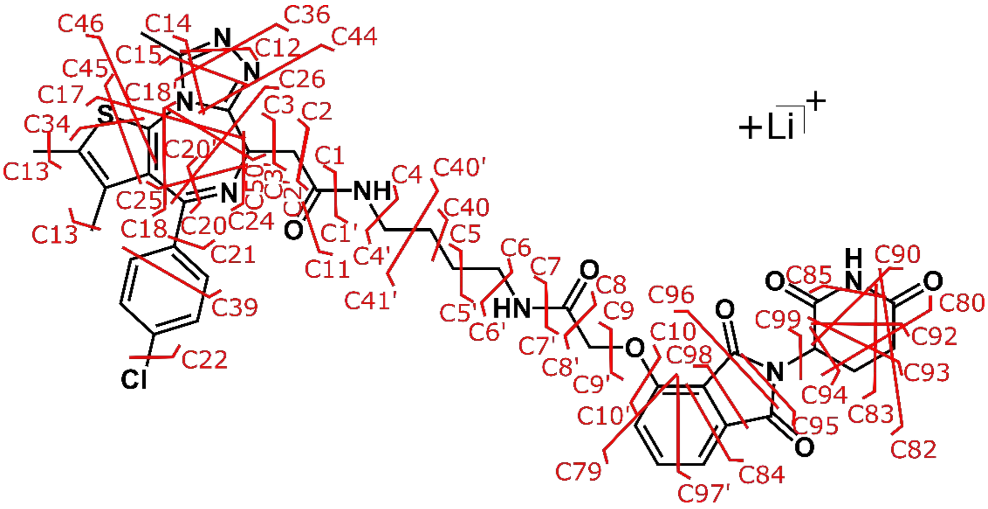

Figure S8 Cleavage diagram of dBET1 [M+Li]⁺ using IRMPD (70.0% for 1.0 s)

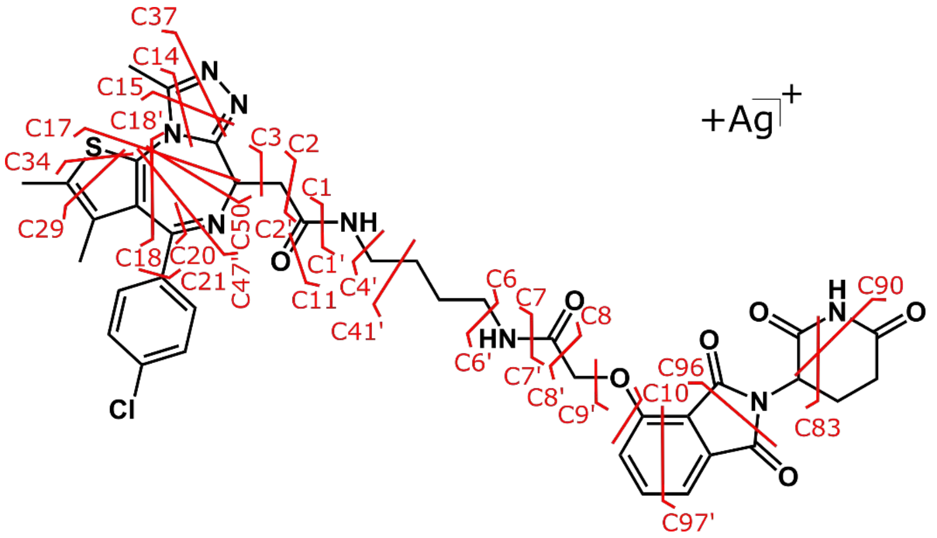

Figure S9 Cleavage diagram of dBET1 [M+Ag]⁺ using IRMPD (80.0% for 1.0 s)

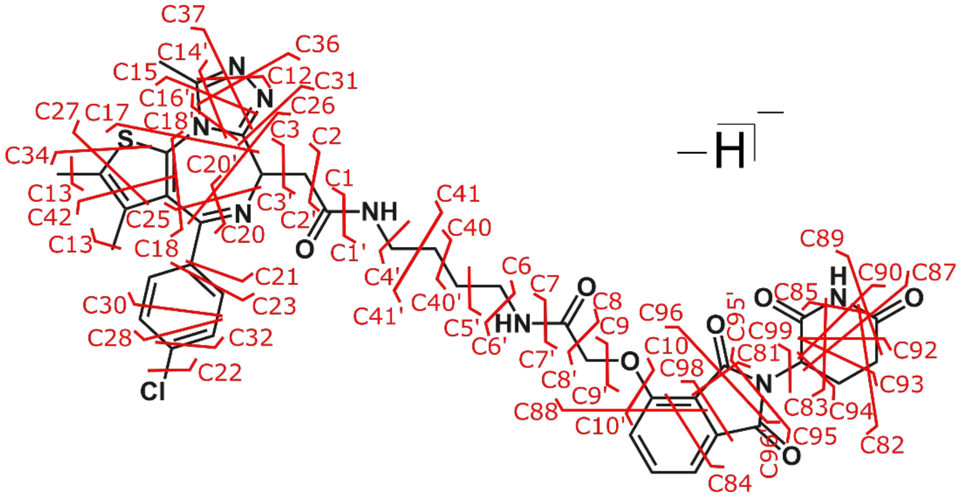

Figure S10 Cleavage diagram of dBET1 [M–H]⁻ using IRMPD (35% for 0.2 s)

S3 Structural characterisation of dBET1 by Ultraviolet Photo-Dissociation

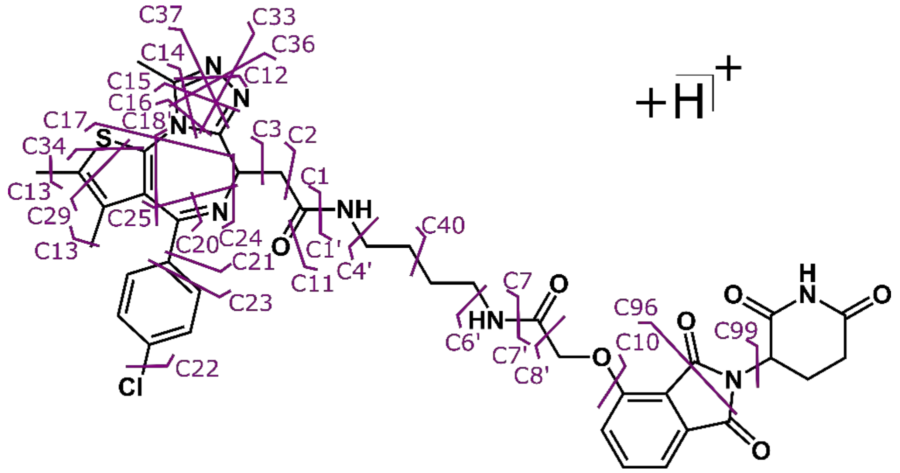

Figure S11 Cleavage diagram of dBET1 [M+H]$^+$ using UVPD (1 shot of 5 mJ)

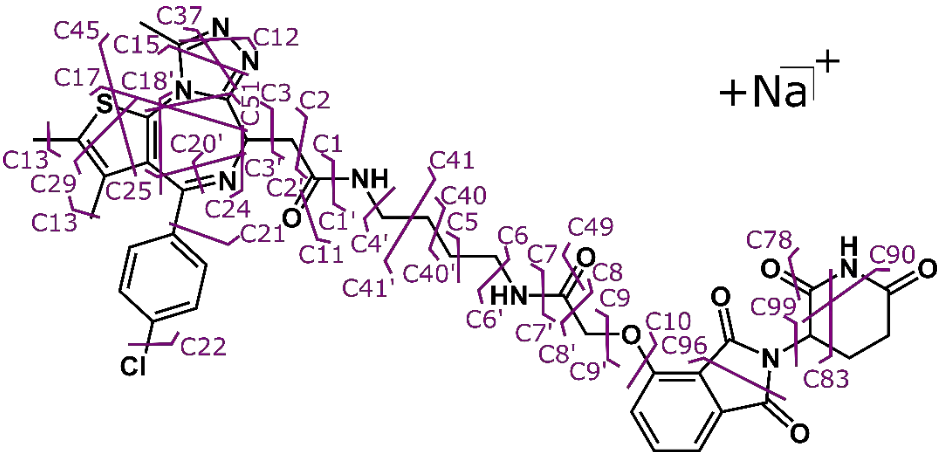

Figure S12 Cleavage diagram of dBET1 [M+Na]$^+$ using UVPD (8 shots of 3.6 mJ)

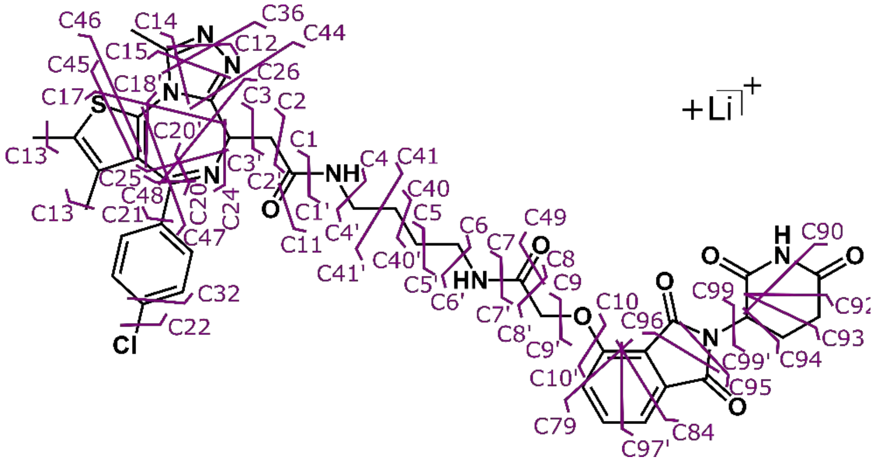

Figure S13 Cleavage diagram of dBET1 [M+Li]+ using UVPD (5 shots of 3.4 mJ)

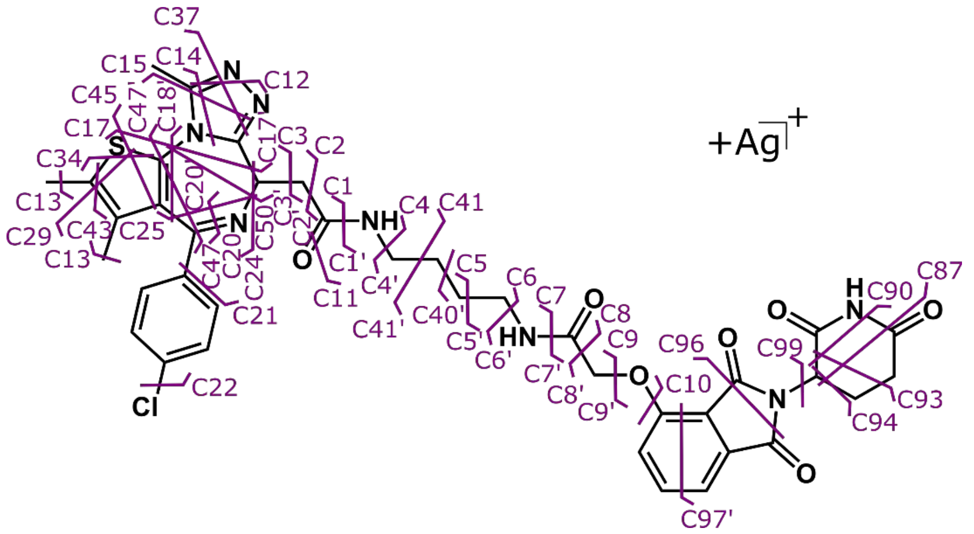

Figure S14 Cleavage diagram of dBET1 [M+Ag]+ using UVPD (3 shots of 3.5 mJ)

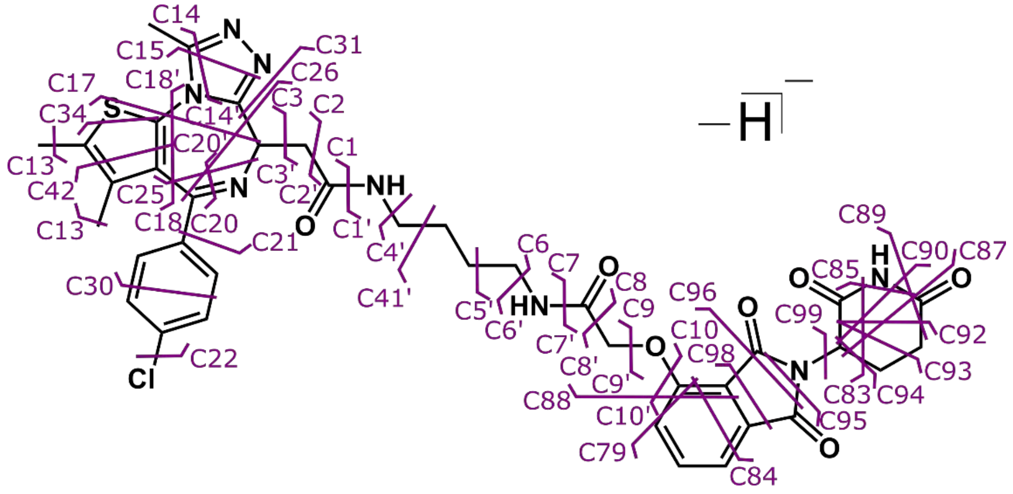

Figure S15 Cleavage diagram of dBET1 [M–H]− using UVPD (6 shots of 3 mJ)

S4 Summary of cleavages for dBET1

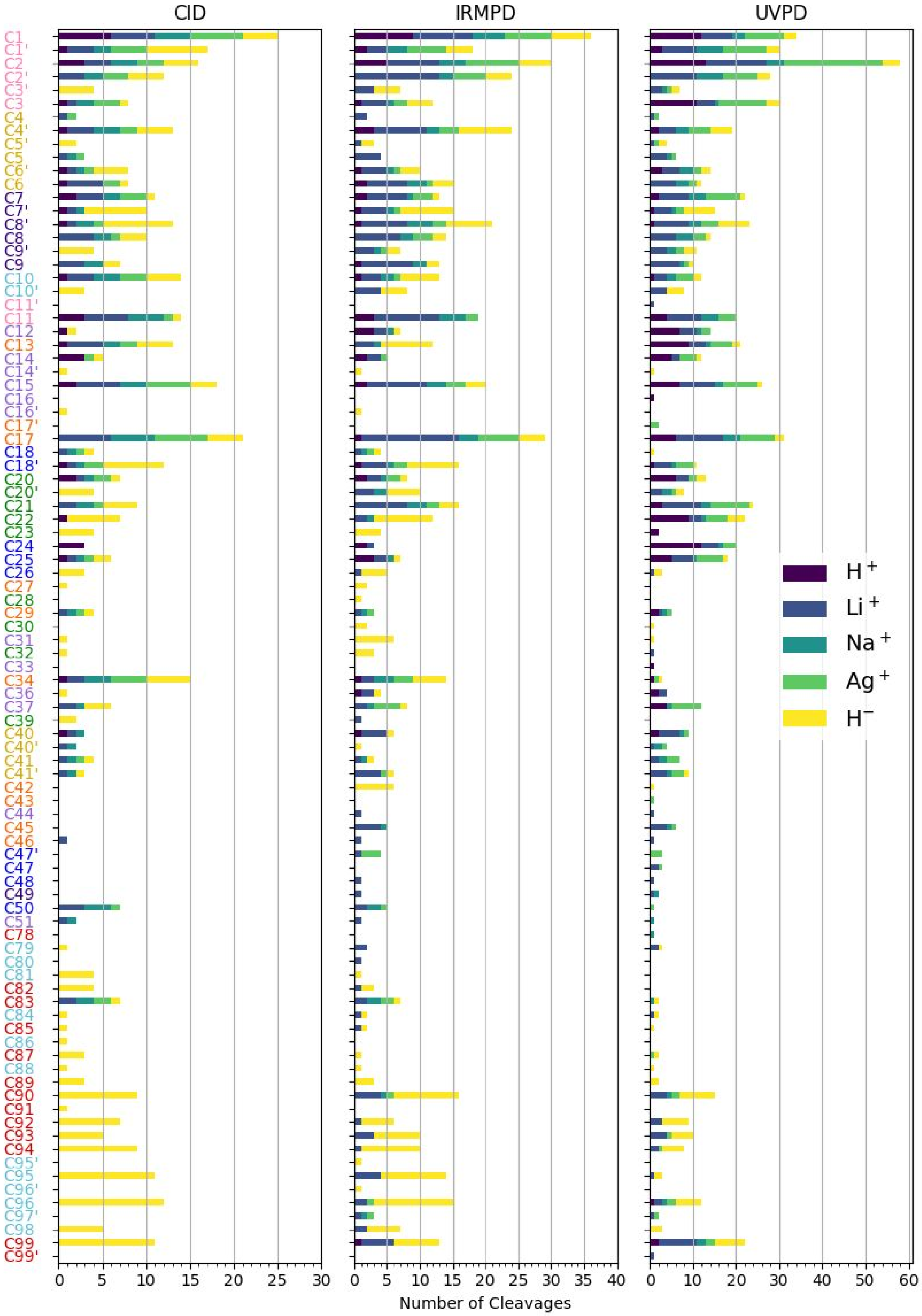

Figure S16 Frequency of bond cleavages for dBET1 using CID-MS/MS, IRMPD-MS/MS, and UVPD-MS/MS for each cation and deprotonate form, cleavages are colour coded based on the moieties in Scheme 1 (see main paper).

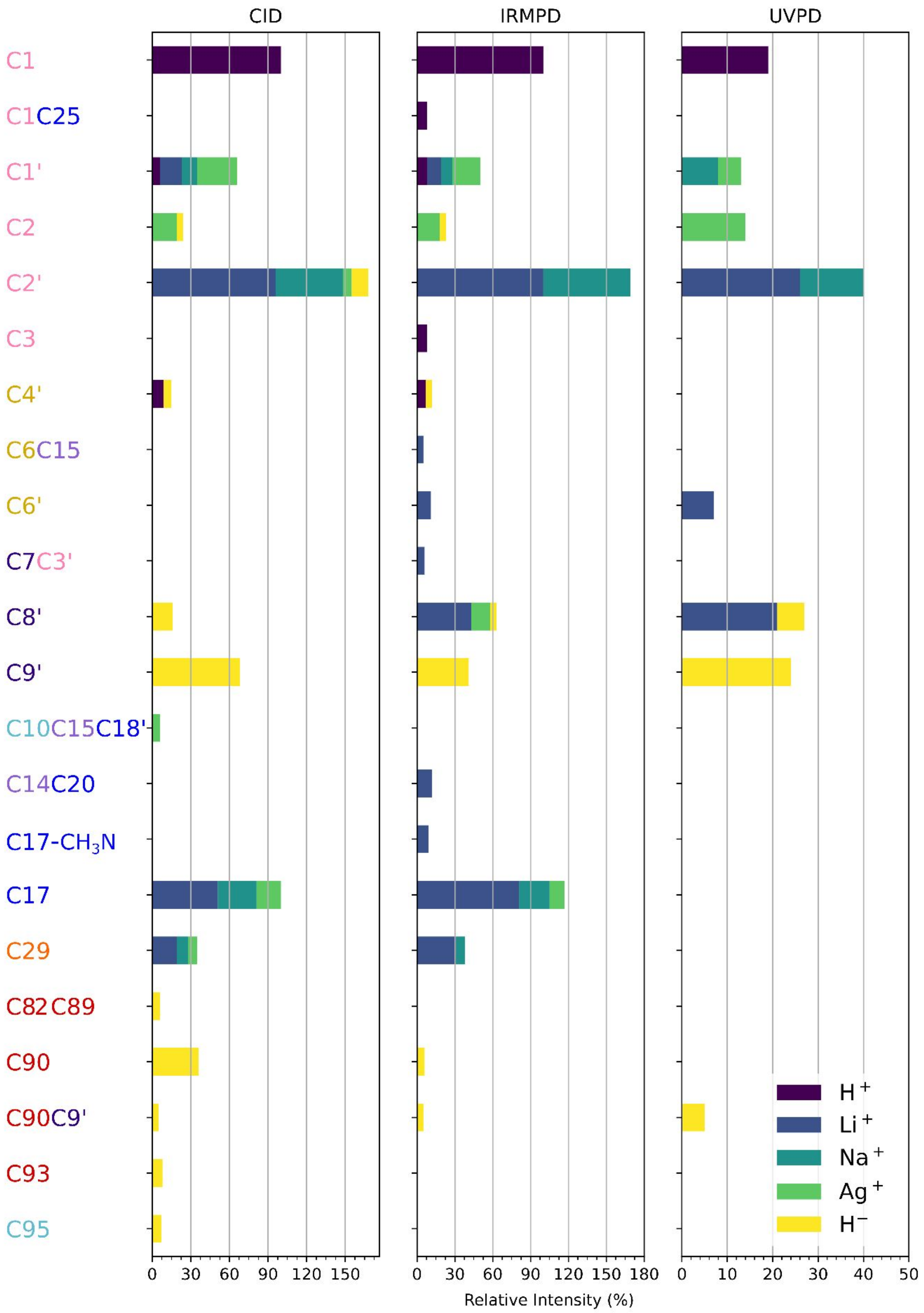

Figure S17 Most intense (relative intensity > 5%) product ions for dBET1 using CID-MS/MS, IRMPD-MS/MS, and UVPD-MS/MS for each cation and deprotonate form, cleavages are colour coded based on the moieties in Scheme 1 (see main paper).

S5 Cleavages of dBET1 [M+Ag]$^+$ molecular ion

m/z 230.9682226

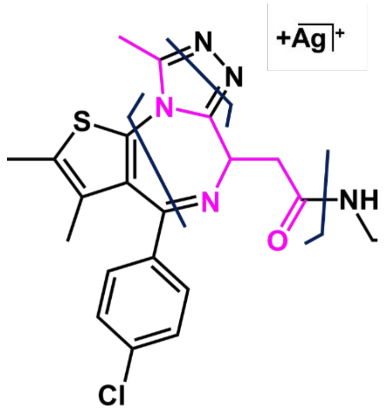

m/z 185.9467523

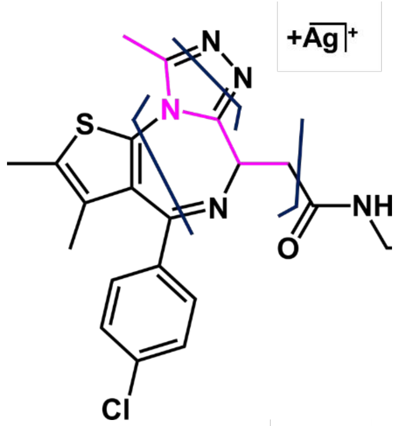

m/z 147.9310889

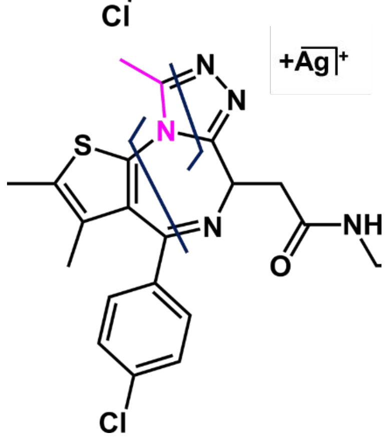

m/z 195.0045973

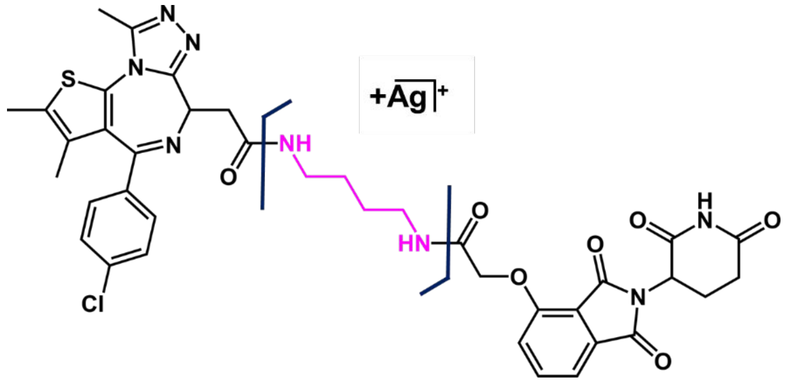

Figure S18 Cleavages observed for dBET1 [M+Ag]$^+$ where the silver adduct was found to still be attached

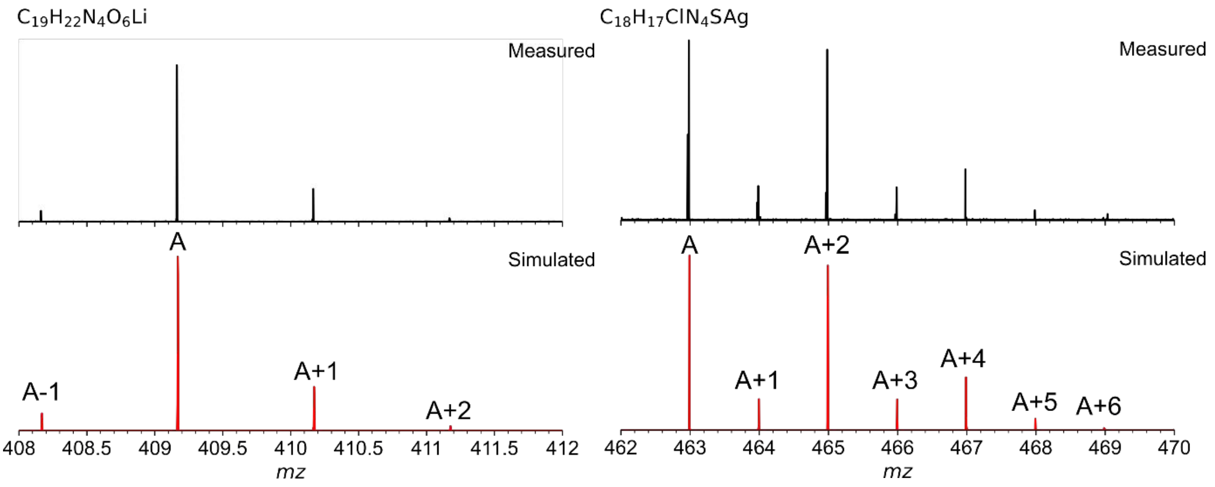

Supplementary Figure S19 Measured (top) and simulated (bottom) isotope pattern of a dBET1 cleavage at C1' ($C_{19}H_{22}N_4O_6Li$), lithium analogue (left); and C2 ($C_{18}H_{17}ClN_4SAg$), silver analogue (right).

S6 Structural characterisation of VZ185 by Collision Induced Dissociation

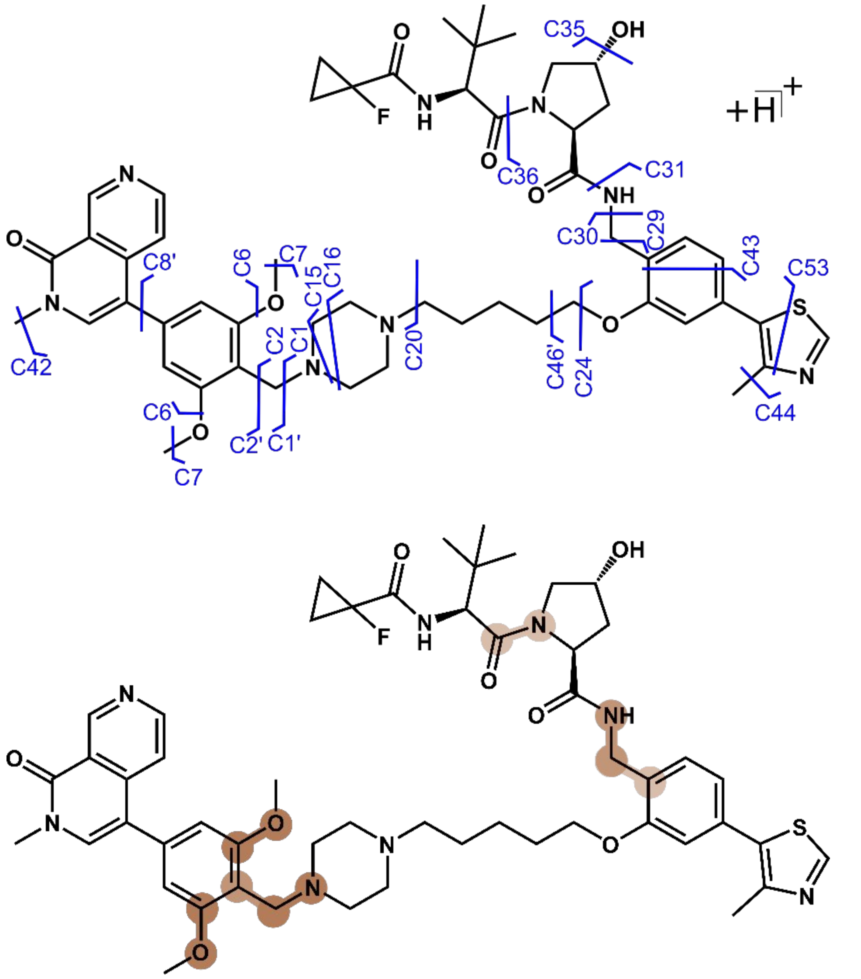

Figure S20 Cleavage diagram and dumbbell plot displaying the most common dissociation locations for VZ185 [M+H]+ using CID at 34.5 V

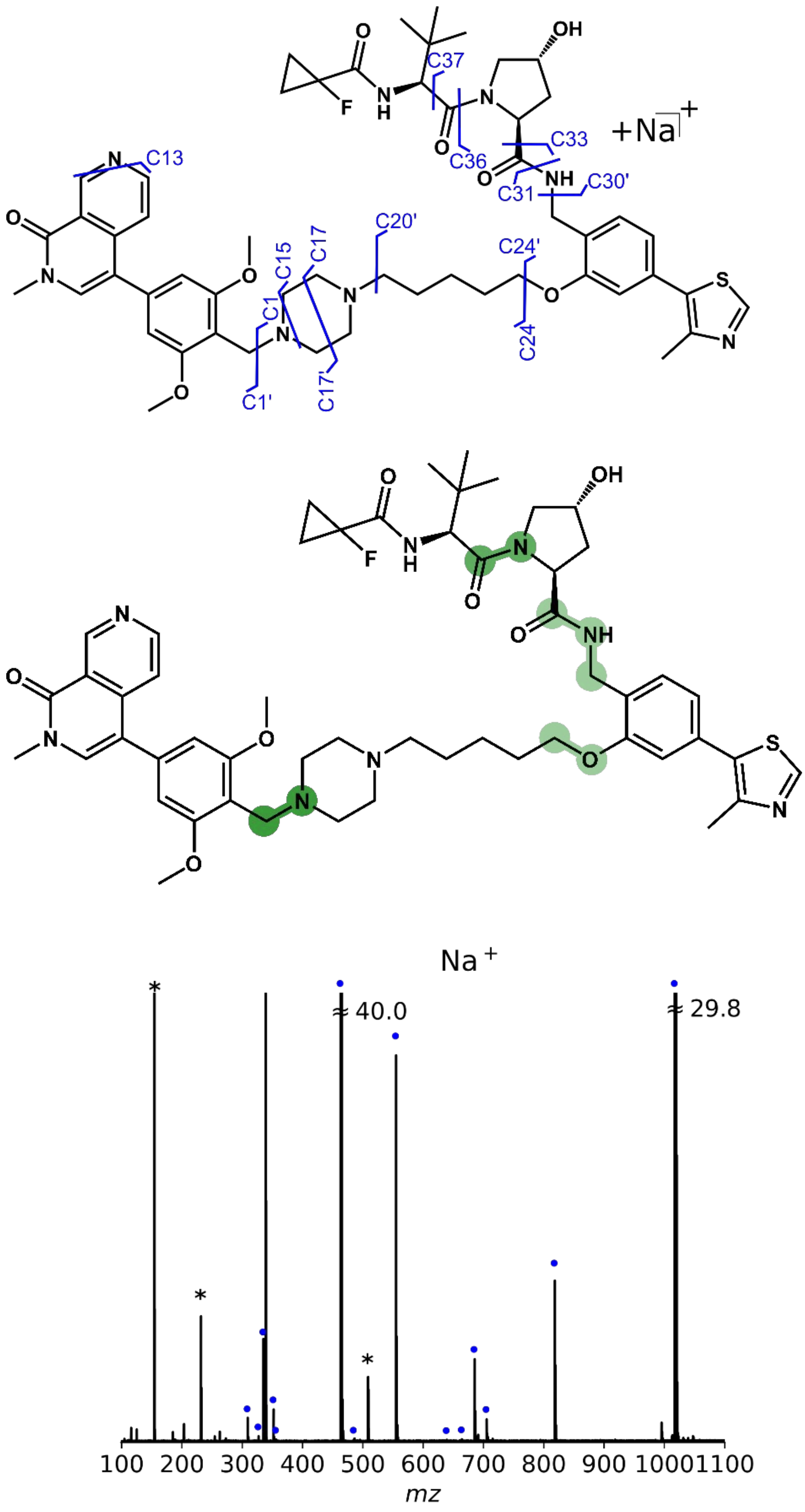

Figure S21 Cleavage diagram, dumbbell plot displaying the most common dissociation points, and product ion spectrum for VZ185 $[M+Na]^+$ using CID at 39 V.

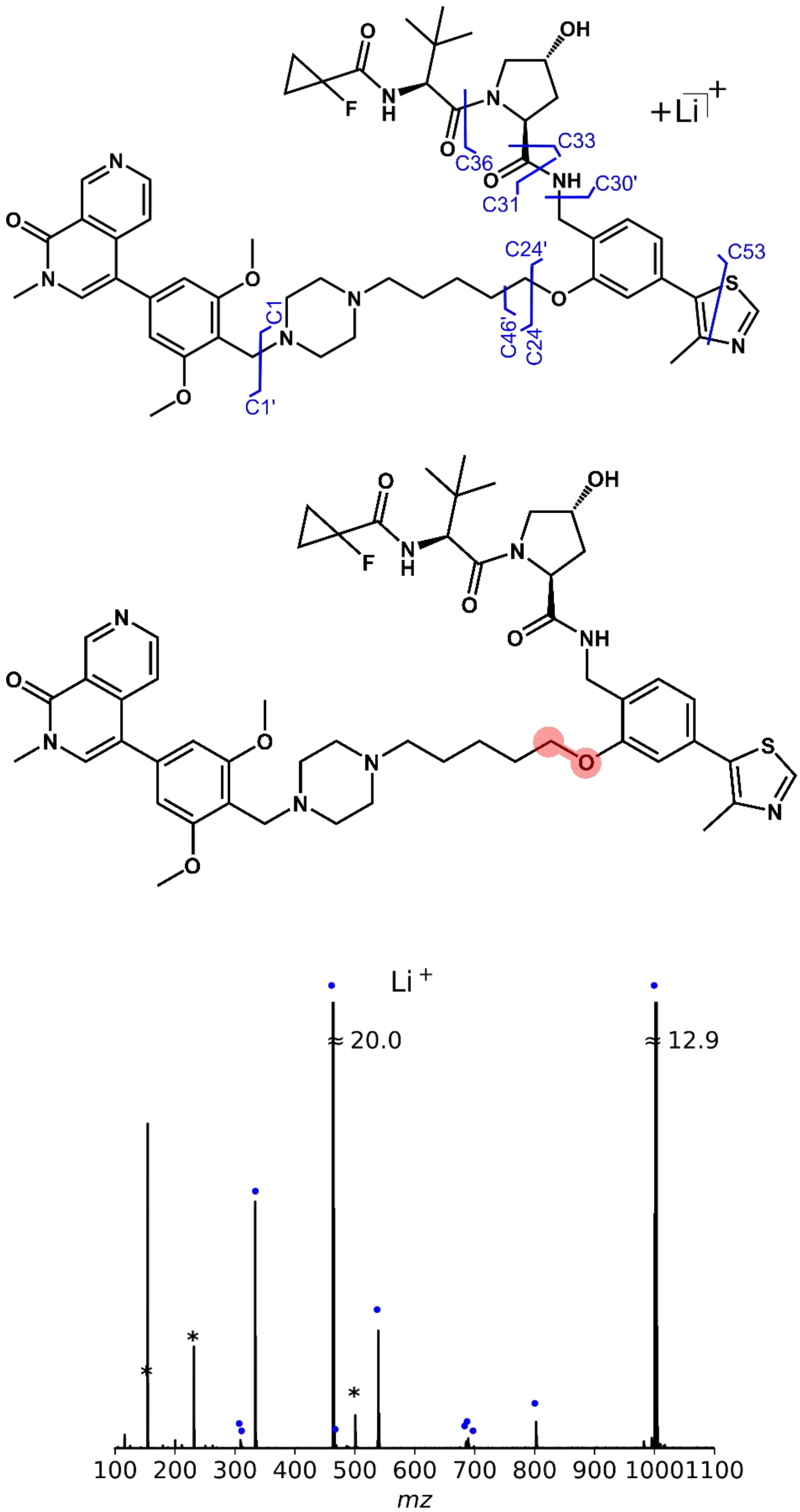

Figure S22 Cleavage diagram and dumbbell plot displaying the most common dissociation locations for VZ185 [M+Li]+ using CID at 35.5 V.

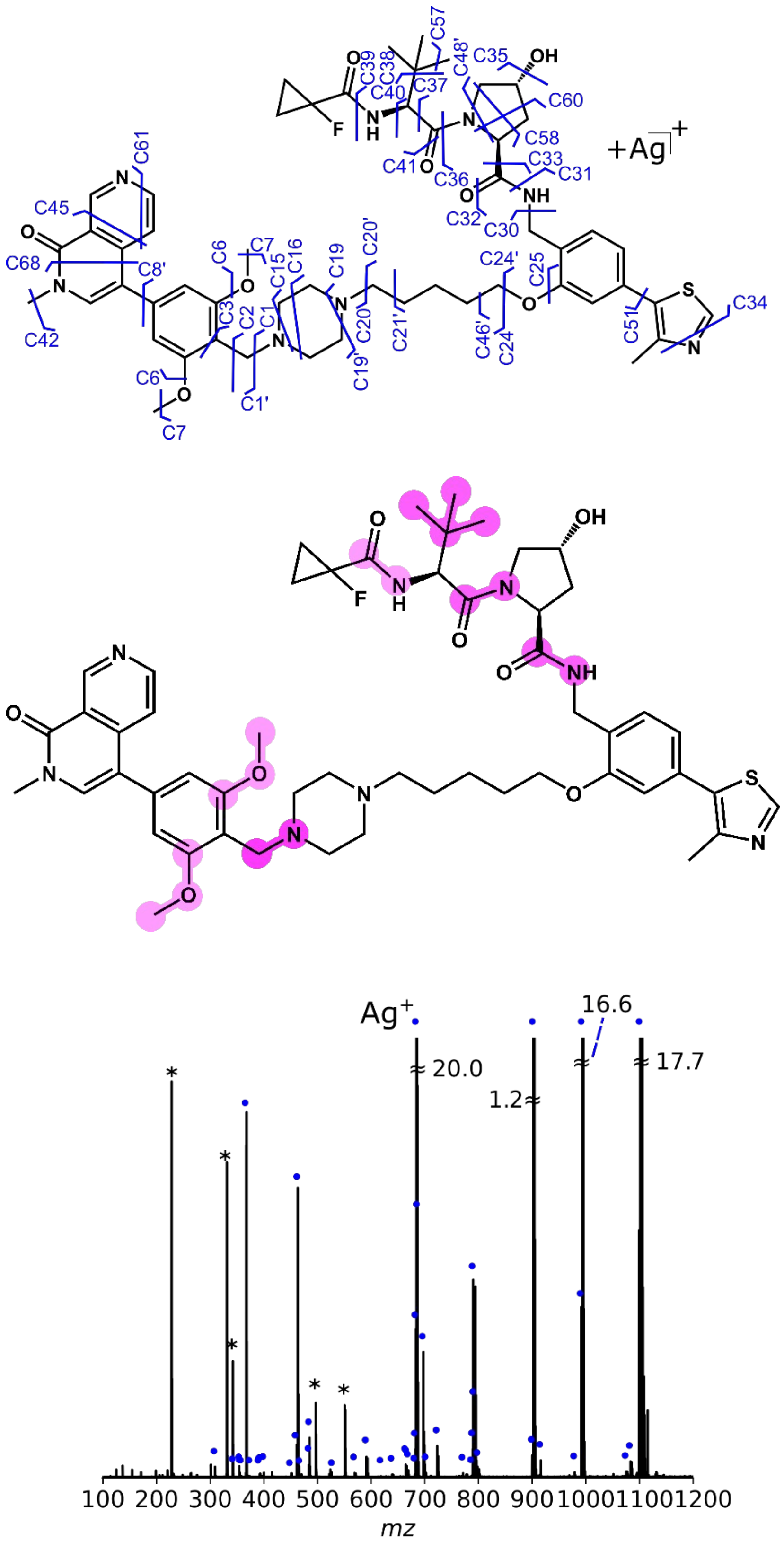

Figure S23 Cleavage diagram and dumbbell plot displaying the most common dissociation locations for VZ185 [M+Ag]+ using CID at 37 V.

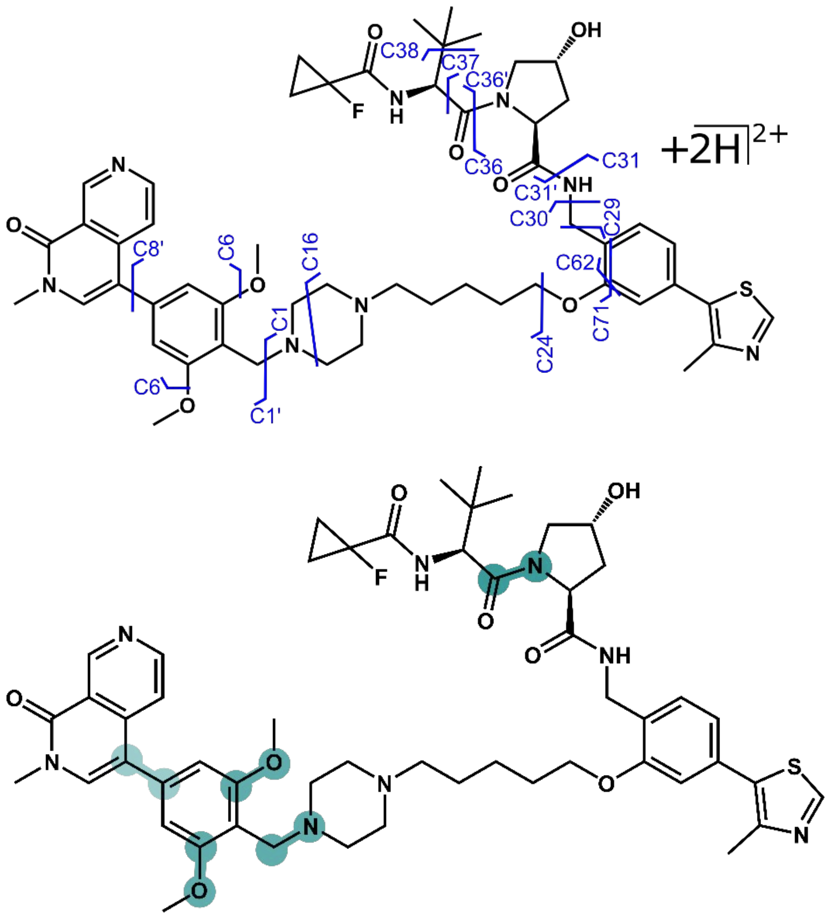

Figure S24 Cleavage diagram and dumbbell plot displaying the most common dissociation locations for VZ185 $[M+2H]^{2+}$ using CID at 9.2 V.

S7 Structural characterisation of VZ185 by Infrared Multiphoton Dissociation

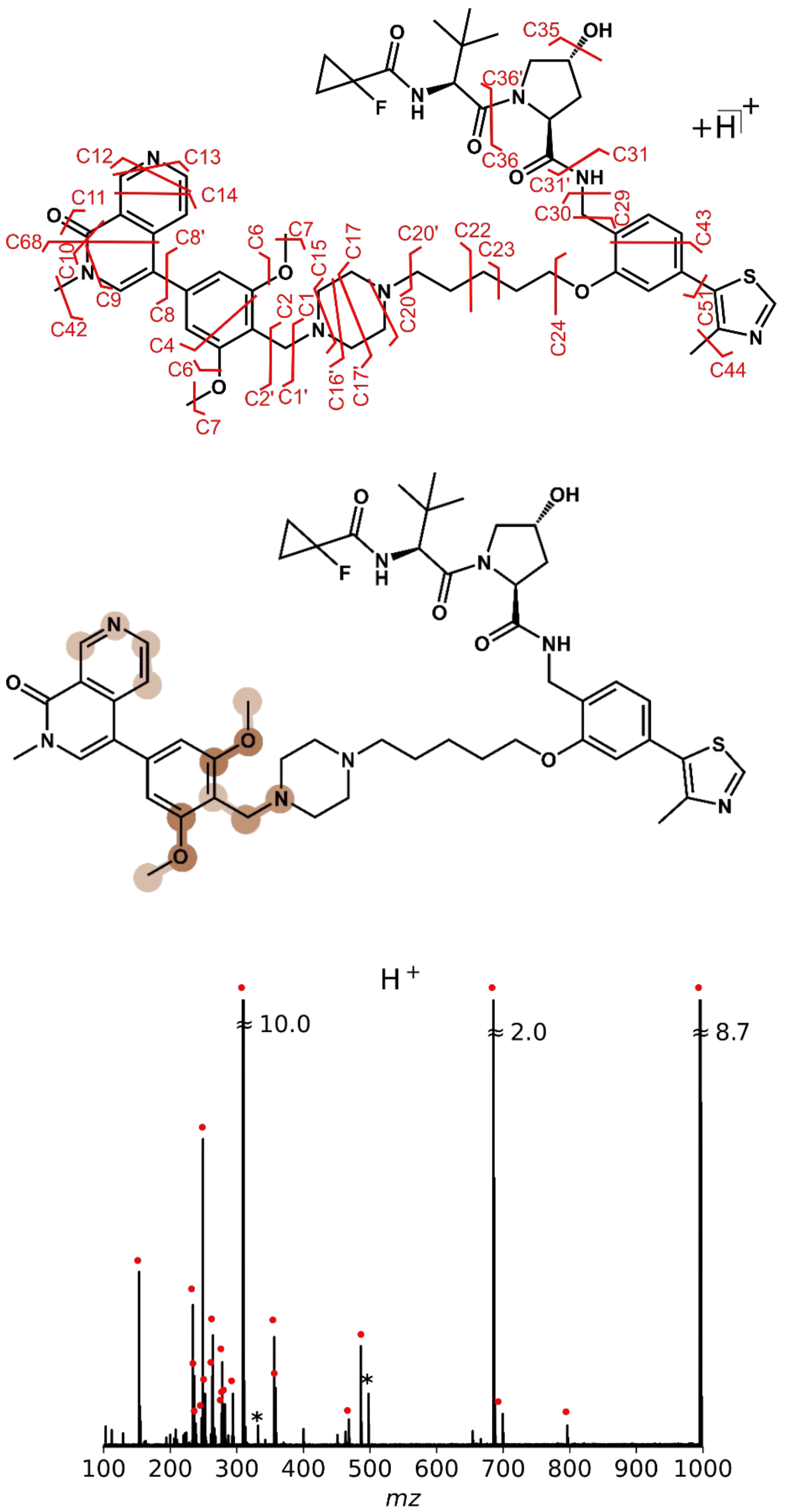

Figure S25 Cleavage diagram and dumbbell plot displaying the most common dissociation locations for VZ185 [M+H]⁺ using IRMPD (35% for 0.25 s).

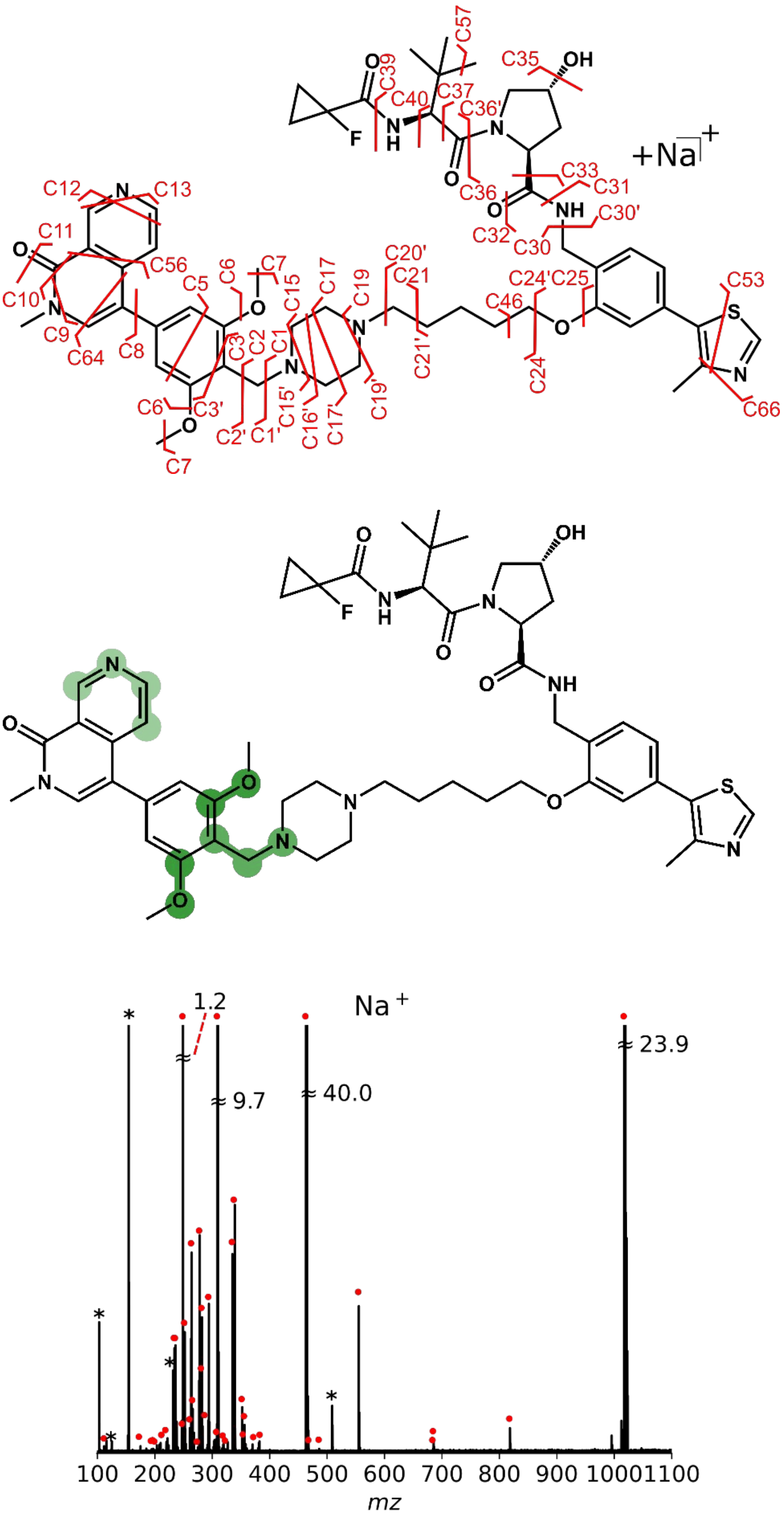

Figure S26 Cleavage diagram and dumbbell plot displaying the most common dissociation locations for VZ185 [M+Na]⁺ using IRMPD (35% for 0.85 s).

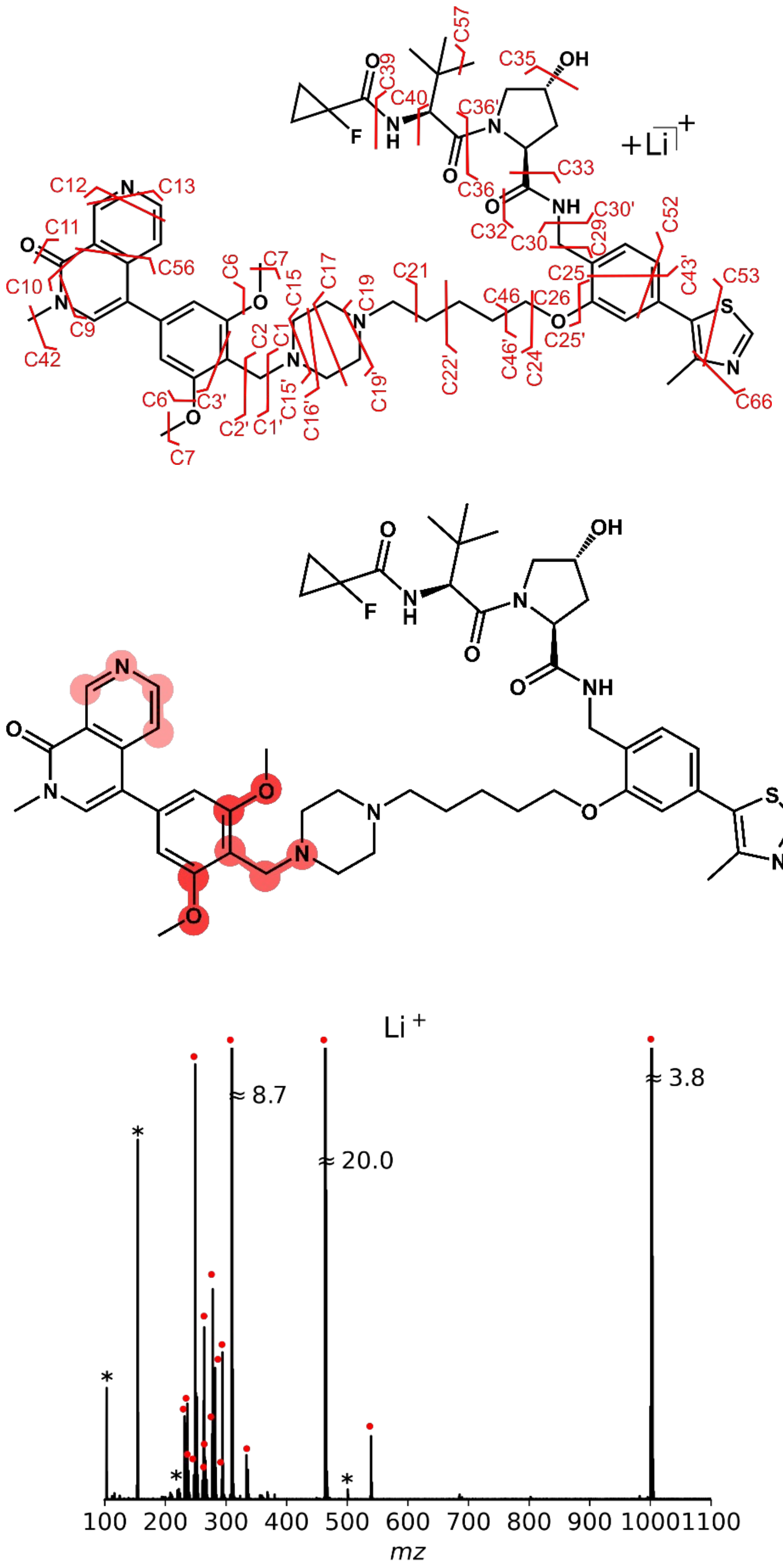

Figure S27 Cleavage diagram and dumbbell plot displaying the most common dissociation locations for VZ185 [M+Li]+ using IRMPD (50.0% for 0.85 s).

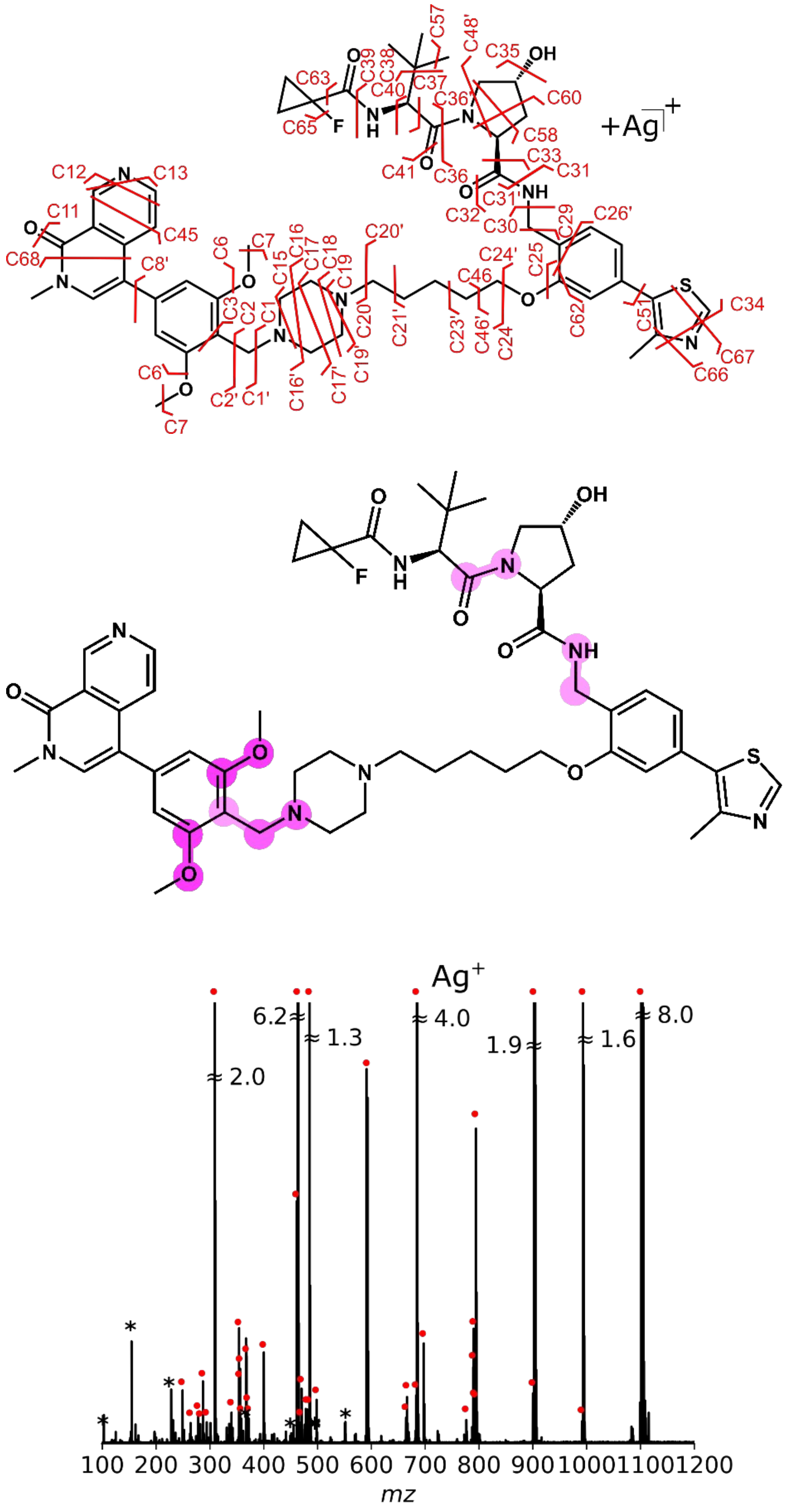

Figure S28 Cleavage diagram and dumbbell plot displaying the most common dissociation locations for VZ185 [M+H]+ using IRMPD (40.0% for 0.85 s).

S8 Structural characterisation of VZ185 by Ultraviolet Photo-Dissociation

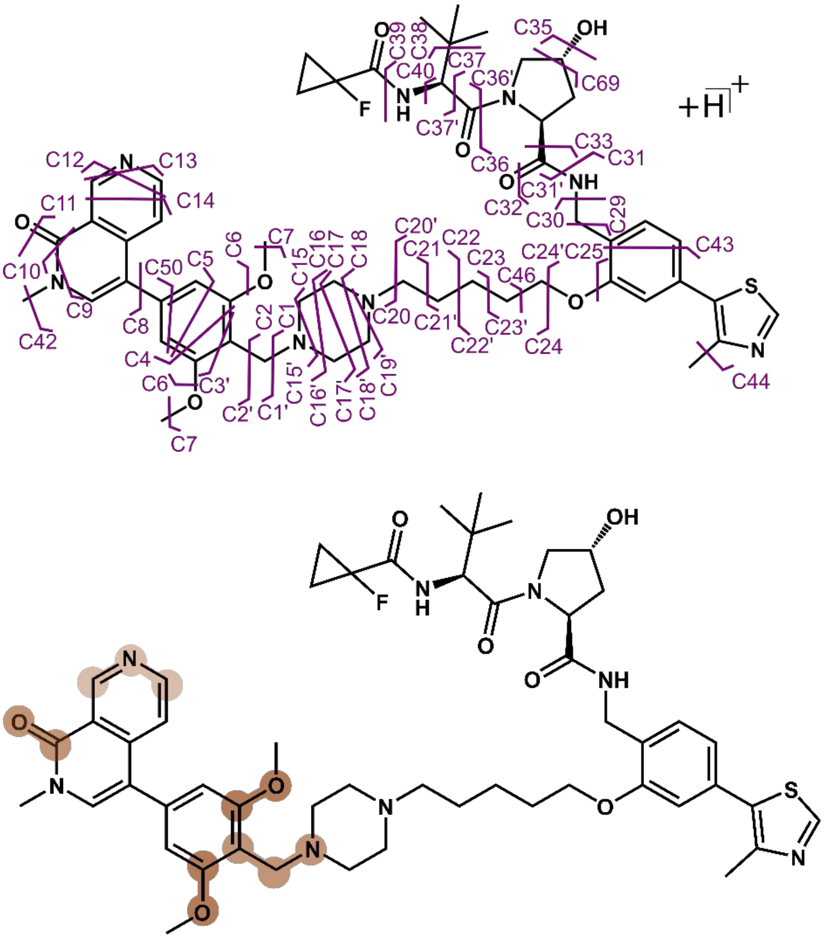

Figure S29 Cleavage diagram and dumbbell plot displaying the most common dissociation locations for VZ185 [M+H]$^+$ using UVPD (5 shots of 3.3 mJ)

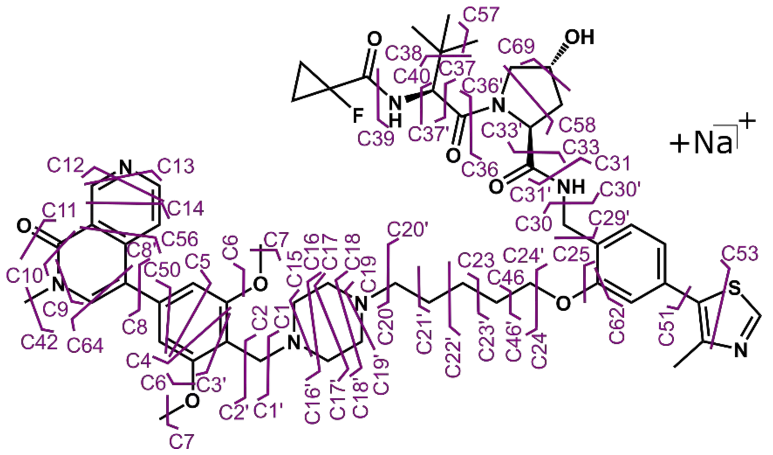

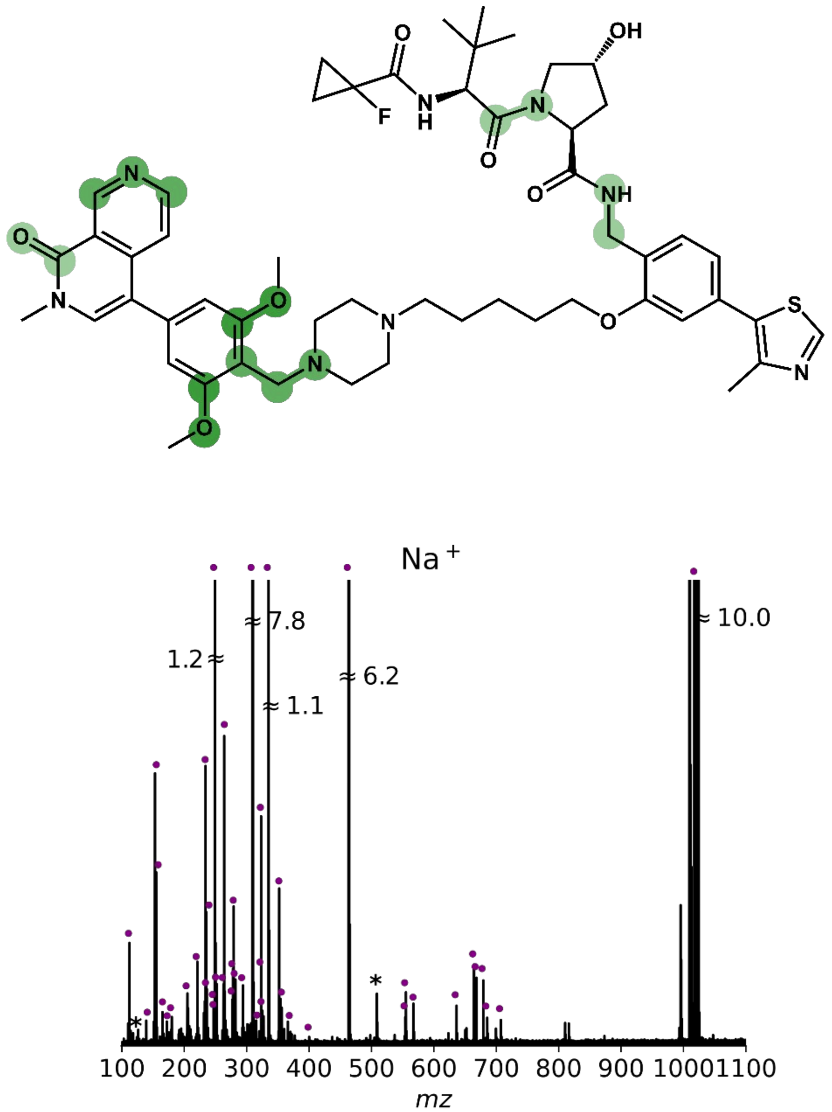

Figure S30 Cleavage diagram and dumbbell plot displaying the most common dissociation locations for VZ185 [M+Na]+ using UVPD (5 shots of 3.2 mJ).

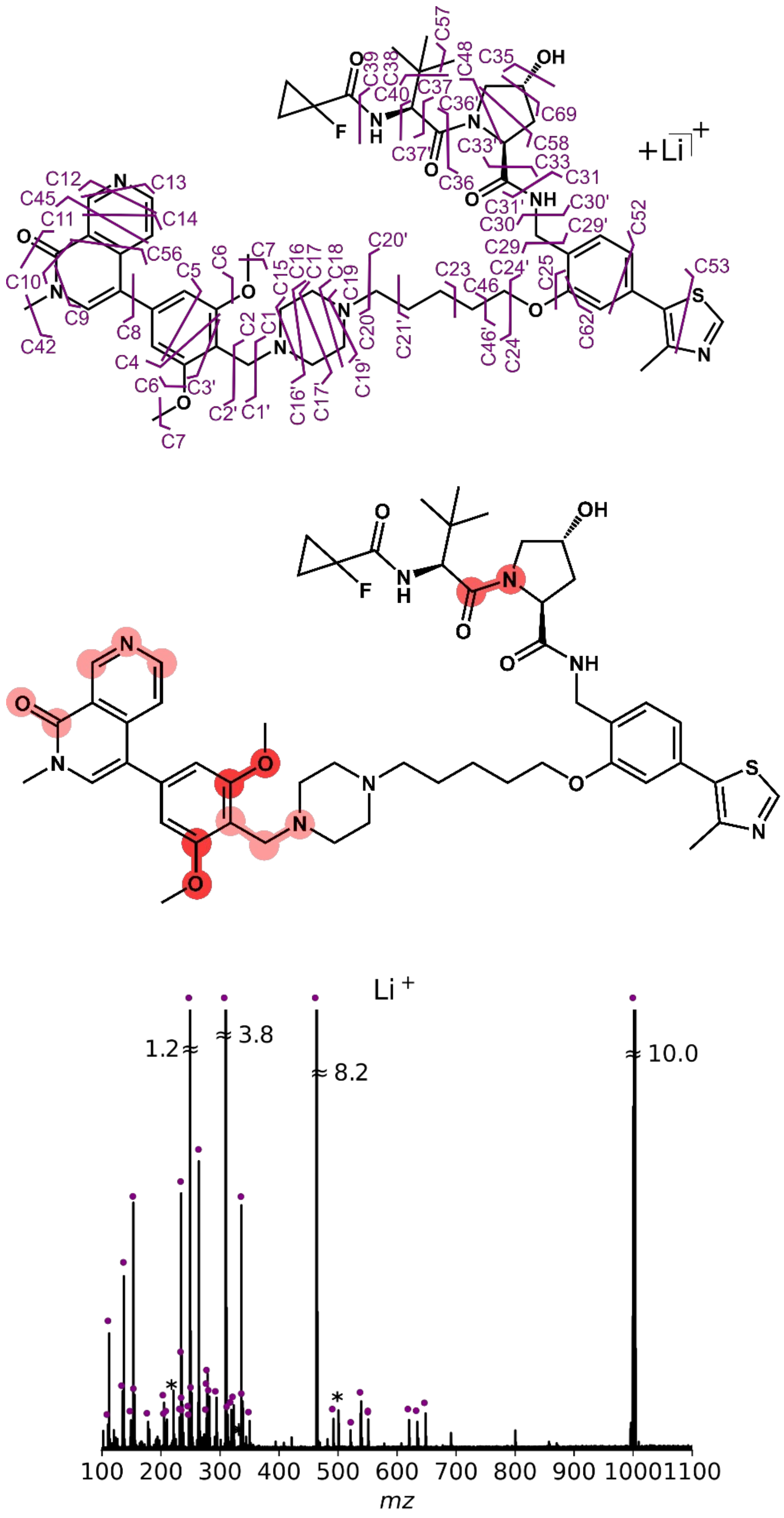

Figure S31 Cleavage diagram and dumbbell plot displaying the most common dissociation locations for VZ185 [M+Li]⁺ using UVPD (5 shots of 3 mJ).

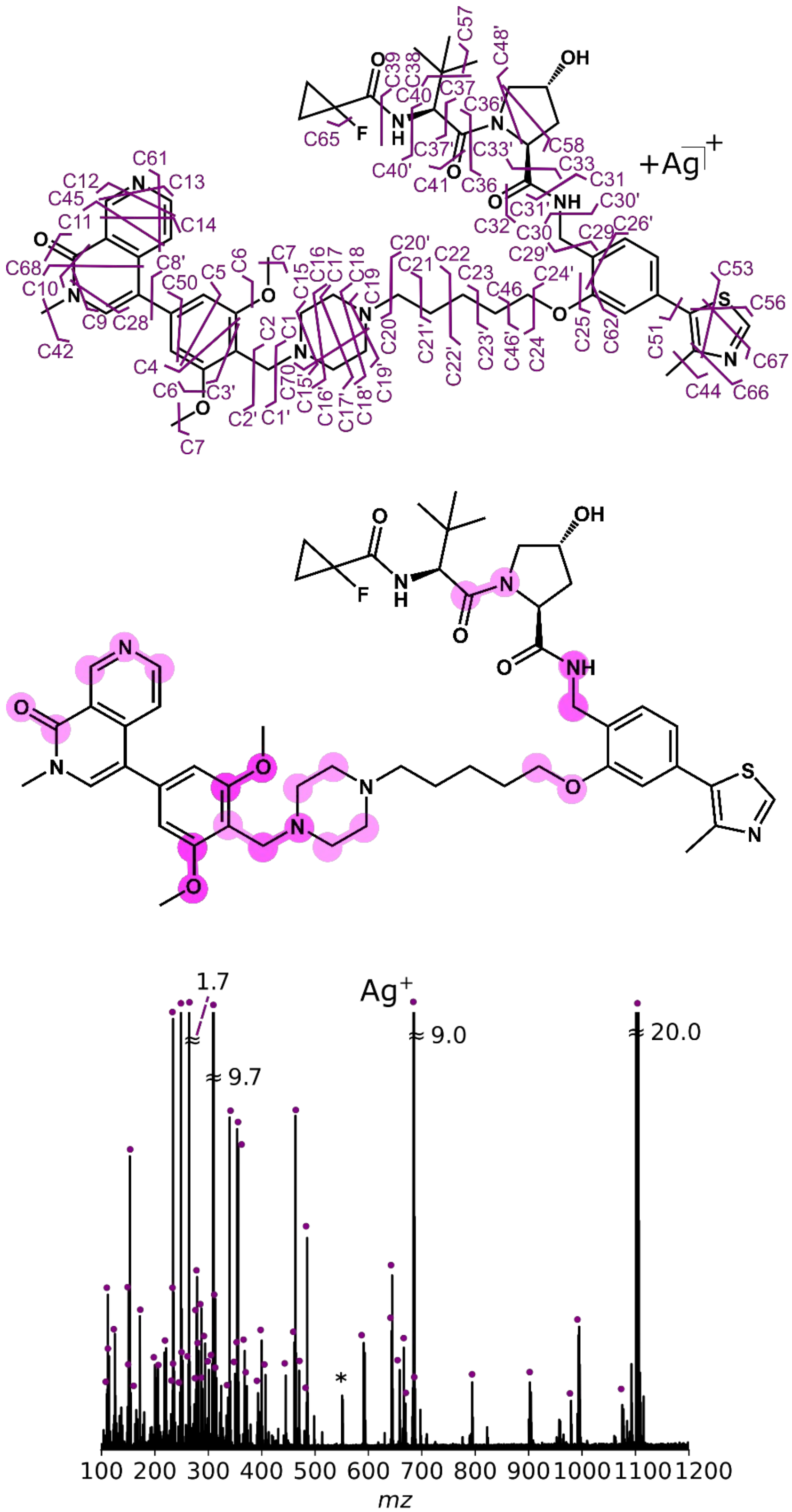

Figure S32 Cleavage diagram and dumbbell plot displaying the most common dissociation locations for VZ185 $[M+Ag]^+$ using UVPD (5 shots of 3.3 mJ).

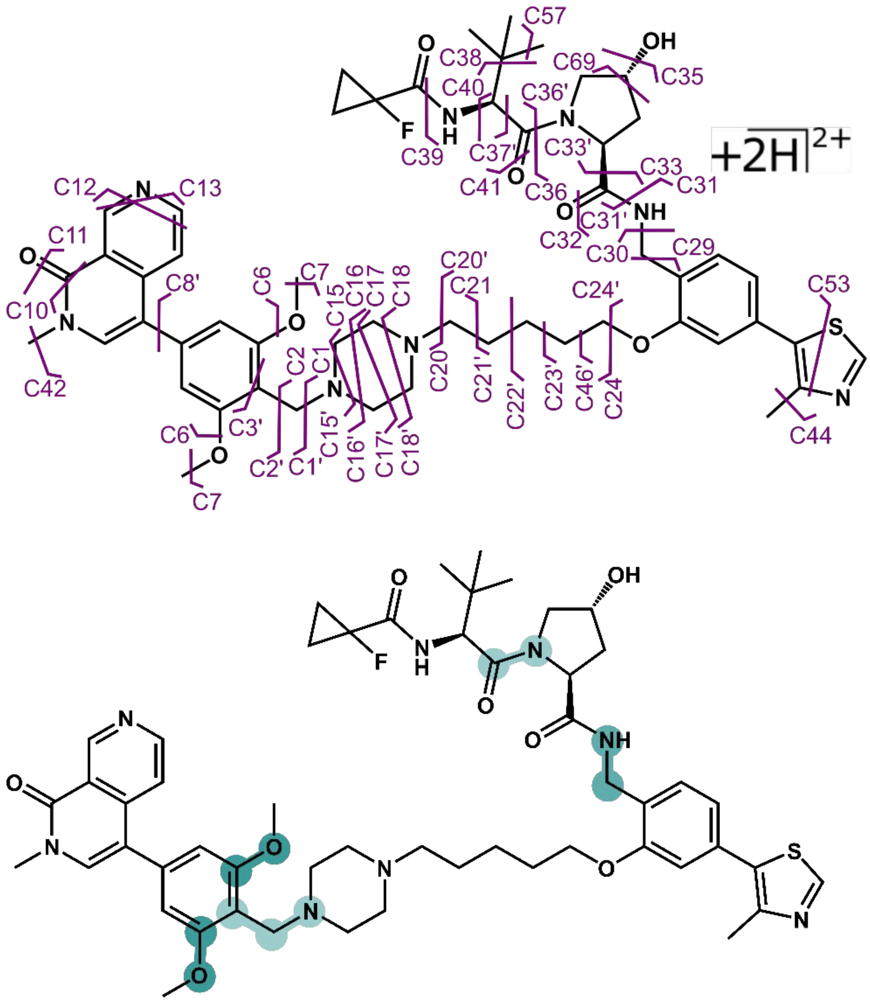

Figure S33 Cleavage diagram and dumbbell plot displaying the most common dissociation locations for VZ185 [M+2H]$^{2+}$ using UVPD (1 shot of 2.5 mJ).

S9 Comparison of VZ185 and its adducts and charge states

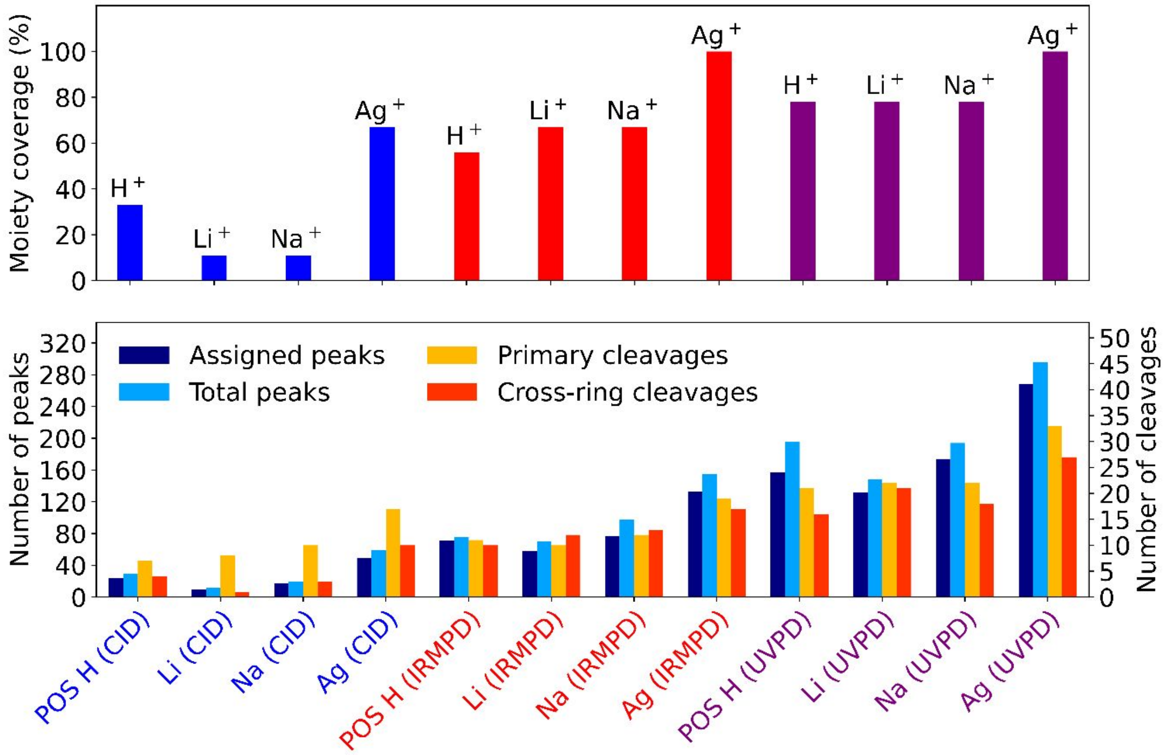

Figure S34 Comparison of each fragmentation technique, CID (blue), IRMPD (red), and UVPD (purple) against the adducts (H^+, Li^+, Na^+, Ag^+), with the total number of peak clusters (including isotope patterns) on left and direct/cross-ring cleavages on right.

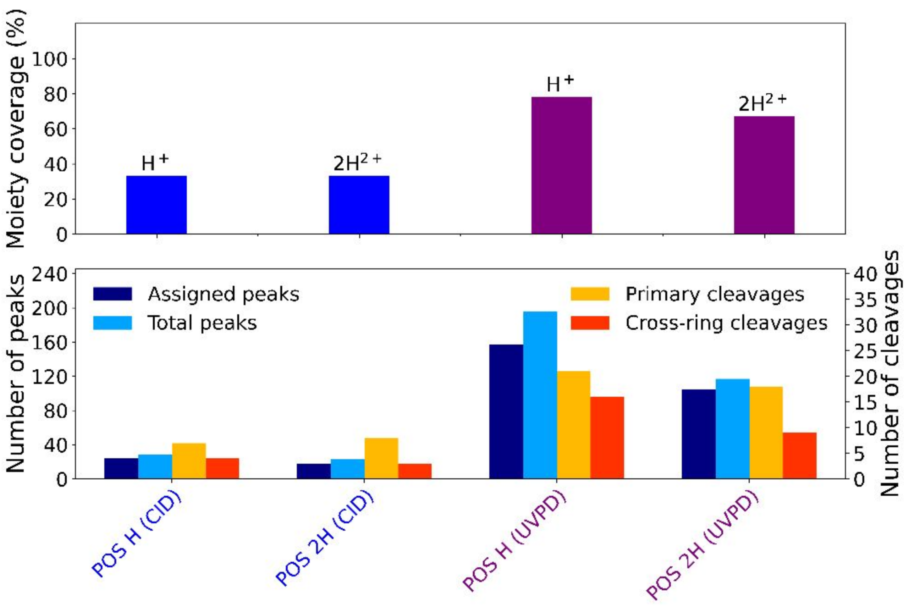

Figure S35 Comparison of each fragmentation technique, CID (blue) and UVPD (purple) against the charge states of VZ185 with the total number of peak clusters (including isotope patterns) on left and direct/cross-ring cleavages on right.

S10 Rearrangements of VZ185

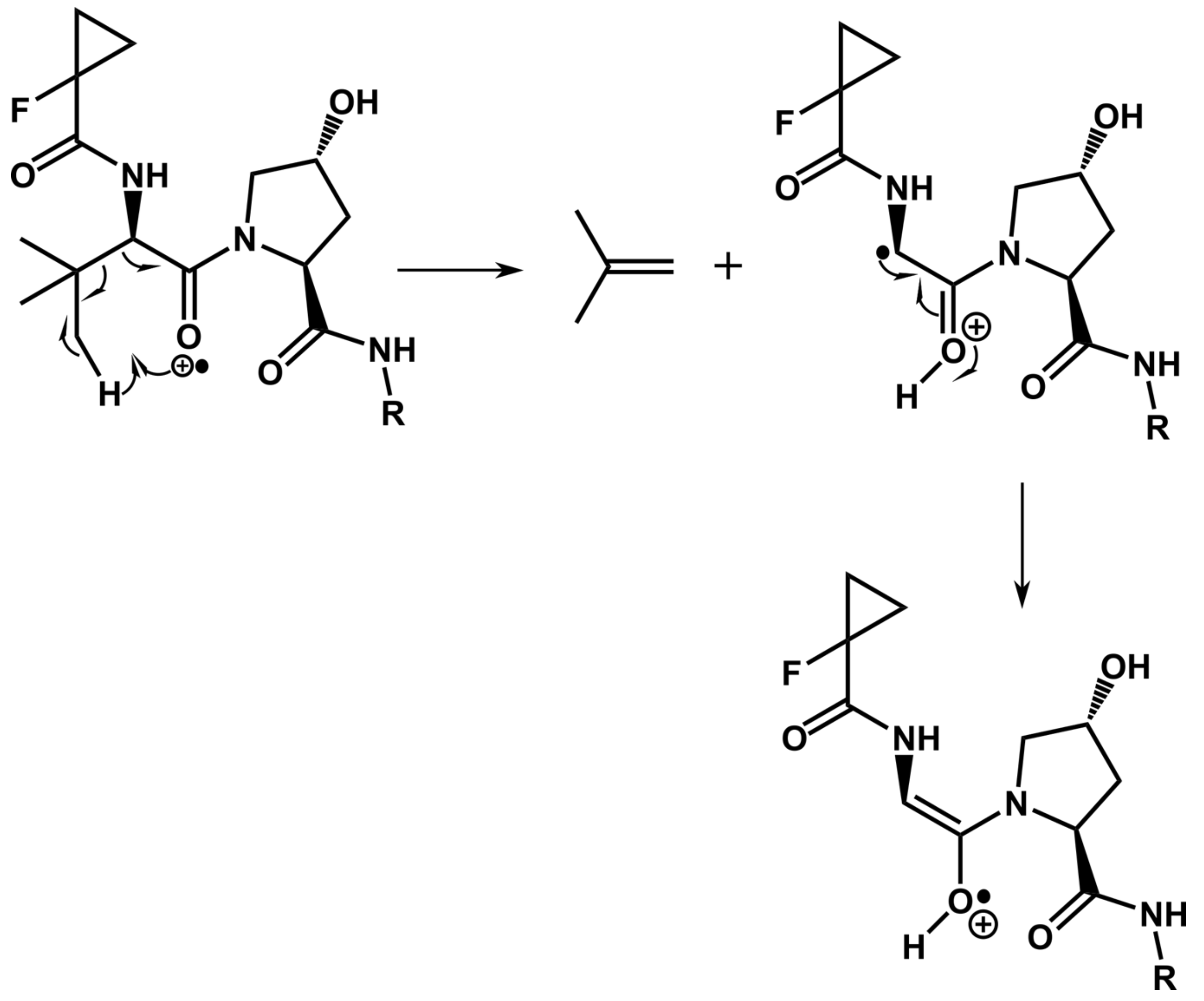

Figure S36 McLafferty Rearrangement of VZ185, which leads to the bond cleavage of tert-butyl substituent (C38).

S11 Trapped Ion Mobility mobilograms of VZ185

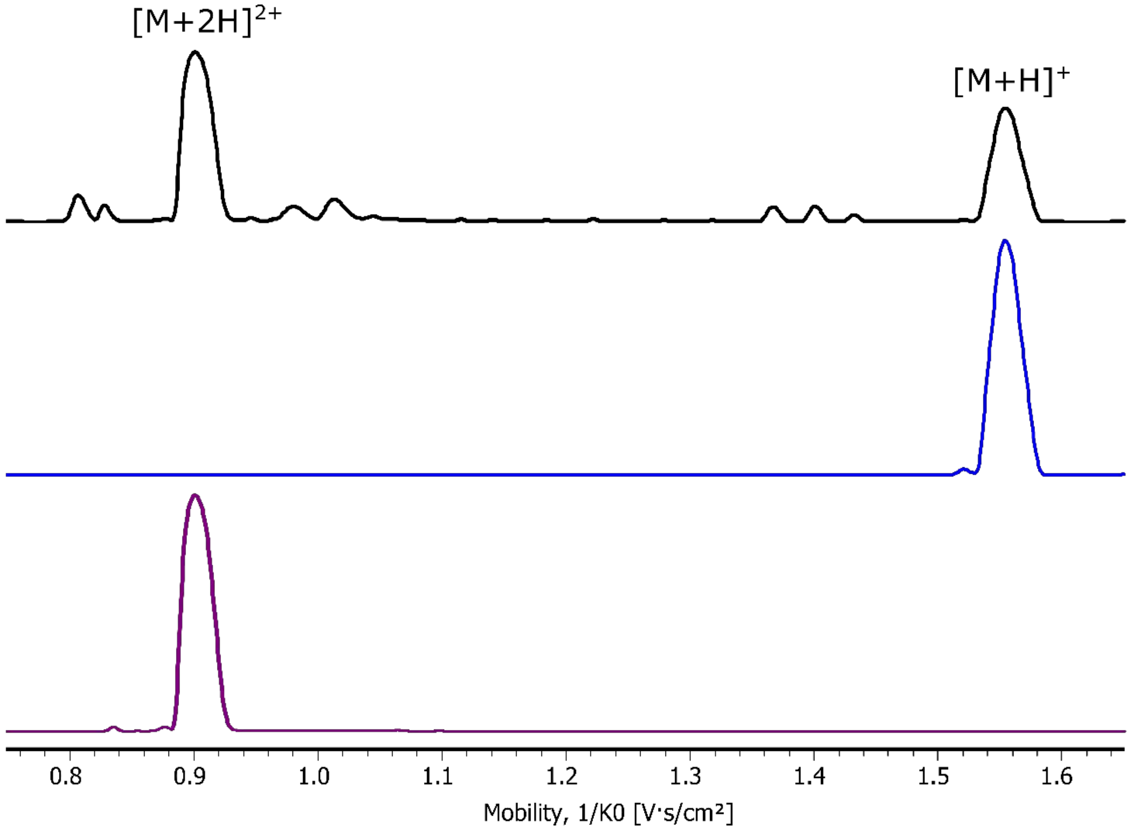

Figure S37 Trapped Ion Mobility (TIMS) spectrum of PROTAC VZ185, displaying the two different mobilities of $[M+H]^+$ (*m/z* 995.485937, extracted mobilogram in blue, CCS: 315.2 Å^2) and $[M+2H]^{2+}$ (*m/z* 498.246607, extracted mobilogram in purple, CCS: 366.7 Å^2).

S12 Summary of cleavages for VZ185

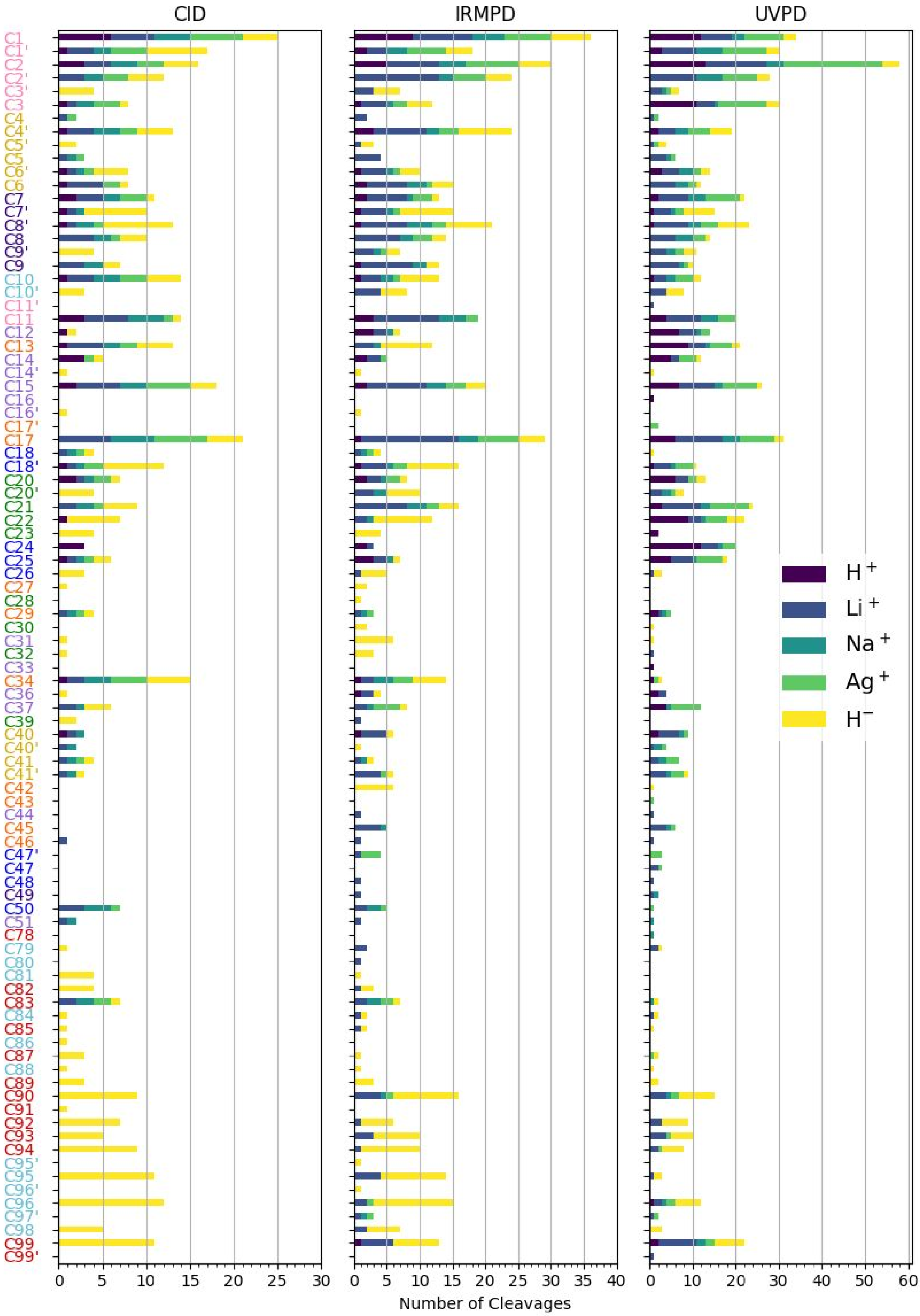

Figure S38 Frequency of bond cleavages for VZ185 using CID-MS/MS, IRMPD-MS/MS, and UVPD-MS/MS for each cation and deprotonate form, cleavages are colour coded based on the moieties in Scheme 1 (see main paper).

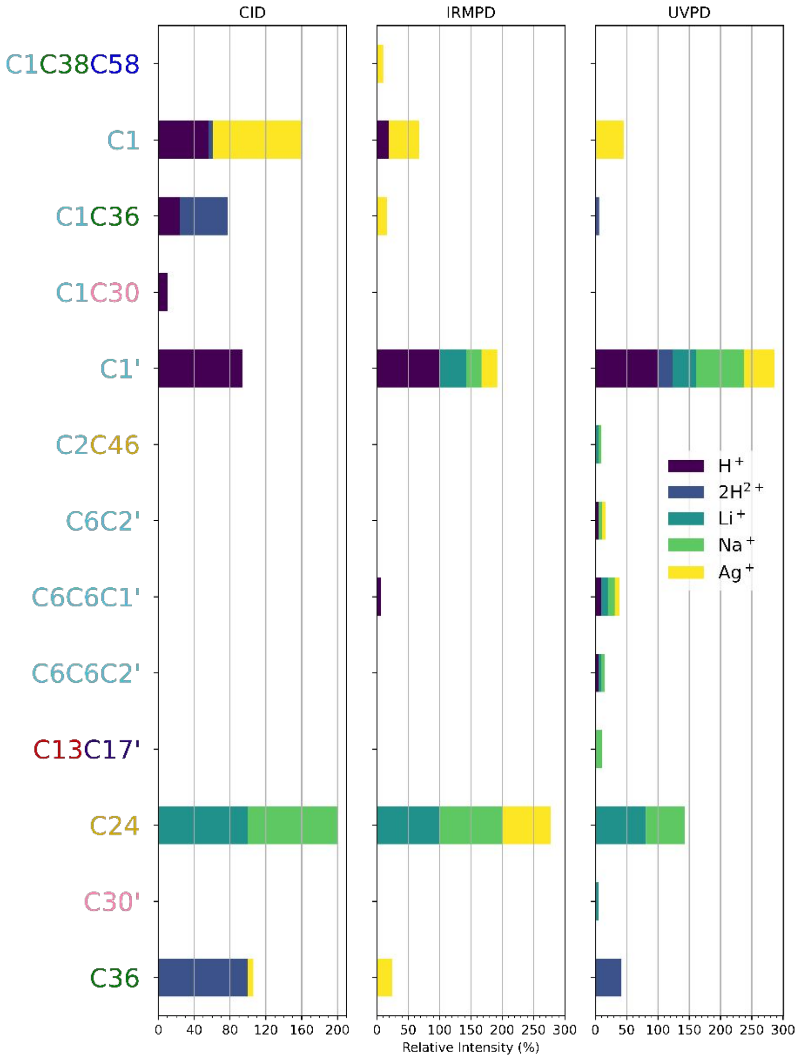

Supplementary Figure 39 Relative abundance of ions produced by fragmentation of VZ185 using CID-MS/MS, IRMPD-MS/MS, and UVPD-MS/MS for each cation, displaying fragments with relative intensities > 5%, colors for the cleavages are based on the moieties described in Scheme 1 and Table 1.

Supplementary Tables of dBET1

Table S1 showing peak list, signal-to-noise ratio (S/N), elemental composition, and assignment with mass errors (ppm) of the $[M+H]^+$ dBET1 compound by CID MS/MS, calibration points are marked by an asterisk (✱).

Measured *m/z*	S/N	Elemental Composition	Theoretical *m/z*	Assignment Error (ppm)	Assignment	
238.056138	17.5	$C_{14}H_{10}N_2S$	238.055921	-0.913	[C13C14C22C24-H]	
246.069727	145.9	$C_{12}H_{12}N_3OS$	246.069560	-0.679	C1C25	
274.045290	10.7	$C_{15}H_{13}ClNS$	274.045175	-0.418	C14C20	
287.066248	183.7	$C_{14}H_{11}N_2O_5$	287.066248	0.000	C8'	✱
289.056119	24.3	$C_{15}H_{14}ClN_2S$	289.056074	-0.157	C14C24	
314.051272	50.2	$C_{16}H_{13}ClN_3S$	314.051323	0.162	C20C24	
314.077086	7.2	$C_{15}H_{12}N_3O_5$	314.077147	0.195	[C6'-H_2O]	
315.061115	75.4	$C_{15}H_{11}N_2O_6$	315.061162	0.149	C7'	
327.035226	16.0	$C_{17}H_{12}ClN_2OS$	327.035338	0.342	C1C15	
327.071590	24.4	$C_{18}H_{16}ClN_2S$	327.071724	0.409	C11C40	
340.066906	97.1	$C_{18}H_{15}ClN_3S$	340.066973	0.196	[C2C12-H_2]	
341.062157	614.0	$C_{17}H_{14}ClN_4S$	341.062222	0.191	C3	
350.092830	13.9	$C_{19}H_{15}ClN_4O$	350.092890	0.172	C1C34	
355.077785	169.4	$C_{18}H_{16}ClN_4S$	355.077872	0.244	[C2-H_2]	
357.093433	40.9	$C_{18}H_{18}ClN_4S$	357.093522	0.250	C2	
359.090456	8.5	$C_{17}H_{16}ClN_4O_3$	359.090545	0.247	C10C15C18'	
366.046158	109.2	$C_{19}H_{13}ClN_3OS$	366.046237	0.216	[C1-NH_3]	
382.088724	289.5	$C_{19}H_{17}ClN_5S$	382.088771	0.124	C1C11	
383.072879	15582.6	$C_{19}H_{16}ClN_4OS$	383.072786	-0.243	C1	
386.134669	1410.2	$C_{19}H_{20}N_3O_6$	386.134662	-0.017	C4'	✱
393.297468	26.5	$C_{20}H_{37}N_6O_2$	393.297251	-0.551		
403.161202	971.9	$C_{19}H_{23}N_4O_6$	403.161211	0.023	C1'	✱
453.162273	31.4	$C_{23}H_{26}ClN_6S$	453.162270	-0.006	C7C11	✱
454.146392	17.4	$C_{23}H_{25}ClN_5OS$	454.146286	-0.233	C6	
471.173031	14.1	$C_{23}H_{28}ClN_6OS$	471.172835	-0.416	[C7+H_2]	
785.229657	2100.5	$C_{38}H_{38}ClN_8O_7S$	785.226721	-3.739	[M+H]	
			Abs mean error	0.250		
			Mean std dev	0.209		

Table S2 showing peak list, signal-to-noise ratio (S/N), elemental composition, and assignment with mass errors (ppm) of the $[M+H]^+$ dBET1 compound by IRMPD MS/MS, calibration points are marked by an asterisk (✱).

Measured *m/z*	S/N	Elemental Composition	Theoretical *m/z*	Assignment Error (ppm)	Assignment	
112.075688	11.6	$C_6H_{10}NO$	112.075690	0.013	[C9C4'-H]	
218.074624	63.4	$C_{11}H_{12}N_3S$	218.074645	0.096	C2C25	
220.090266	20.4	$C_{11}H_{14}N_3S$	220.090295	0.131	[C2C25+H_2]	
246.069531	2370.5	$C_{12}H_{12}N_3OS$	246.069560	0.119	C1C25	
274.045168	33.4	$C_{15}H_{13}ClNS$	274.045175	0.024	C14C20	
275.102593	35.9	$C_{14}H_{15}N_2O_4$	275.102633	0.145	C99C4'	
287.066246	545.2	$C_{14}H_{11}N_2O_5$	287.066248	0.007	C8'	✱
289.056082	20.1	$C_{15}H_{14}ClN_2S$	289.056074	-0.028	C14C24	
300.024396	16.7	$C_{16}H_{11}ClNOS$	300.024439	0.144	[C1C17-H_2]	
314.051320	104.4	$C_{16}H_{13}ClN_3S$	314.051323	0.009	C20C24	
314.077246	7.6	$C_{15}H_{12}N_3O_5$	314.077147	-0.316	[C6'-H_2O]	
315.061155	418.9	$C_{15}H_{11}N_2O_6$	315.061162	0.022	C7'	
327.035306	45.6	$C_{17}H_{12}ClN_2OS$	327.035338	0.097	C1C15	
327.071692	45.8	$C_{18}H_{16}ClN_2S$	327.071724	0.098	C11C40	
340.066979	293.4	$C_{18}H_{15}ClN_3S$	340.066973	-0.019	[C2C12-H_2]	
341.062228	2303.5	$C_{17}H_{14}ClN_4S$	341.062222	-0.019	C3	✱
342.046223	45.4	$C_{17}H_{13}ClN_3OS$	342.046237	0.042	C1C36	
343.059269	566.3	$C_{16}H_{12}ClN_4O_3$	343.059244	-0.073		
350.092836	24.3	$C_{19}H_{15}ClN_4O$	350.092890	0.154	C1C34	
355.077866	433.7	$C_{18}H_{16}ClN_4S$	355.077872	0.018	[C2-H_2]	
357.074905	119.5	$C_{17}H_{14}ClN_4O_3$	357.074894	-0.031		
357.093526	29.8	$C_{18}H_{18}ClN_4S$	357.093522	-0.011	C2	
359.090480	8.5	$C_{17}H_{16}ClN_4O_3$	359.090545	0.180	C10C15C18'	
366.046258	694.5	$C_{19}H_{13}ClN_3OS$	366.046237	-0.058	[C1-NH_3]	
368.061888	35.0	$C_{19}H_{15}ClN_3OS$	368.061887	-0.002	C1C12	
370.113885	36.8	$C_{20}H_{21}ClN_3S$	370.113923	0.102		
382.088781	403.9	$C_{19}H_{17}ClN_5S$	382.088771	-0.026	C1C11	
383.072899	27055.3	$C_{19}H_{16}ClN_4OS$	383.072786	-0.294	C1	
385.150621	80.2	$C_{19}H_{21}N_4O_5$	385.150646	0.064	[C1'-OH]	
386.134695	1896.5	$C_{19}H_{20}N_3O_6$	386.134662	-0.085	C4'	
403.161254	2216.9	$C_{19}H_{23}N_4O_6$	403.161211	-0.106	C1'	
436.135691	12.3	$C_{23}H_{23}ClN_5S$	436.135721	0.068		
437.119758	188.2	$C_{23}H_{22}ClN_4OS$	437.119737	-0.048	[C6C12-H_4]	
453.162261	73.8	$C_{23}H_{26}ClN_6S$	453.162270	0.019	C7C11	✱
454.146236	9.0	$C_{23}H_{25}ClN_5OS$	454.146286	0.109	C6	
455.159327	15.7	$C_{22}H_{24}ClN_6O_3$	455.159293	-0.075		
471.172973	9.0	$C_{23}H_{28}ClN_6OS$	471.172835	-0.294	[C7+H_2]	
475.146731	21.7	$C_{25}H_{24}ClN_6S$	475.146620	-0.234		
493.157161	10.0	$C_{25}H_{26}ClN_6OS$	493.157185	0.048		
494.141161	15.9	$C_{25}H_{25}ClN_5O_2S$	494.141200	0.079		
767.216163	76.9	$C_{38}H_{36}ClN_8O_6S$	767.216156	-0.009	[M-OH_2]	✱
768.200132	126.2	$C_{38}H_{35}ClN_7O_7S$	768.200172	0.052	[M-NH_3]	
785.227806	15545.6	$C_{38}H_{38}ClN_8O_7S$	785.226721	-1.382	[M+H]	
			Abs mean error	0.087		
			Mean std dev	0.082		

Table S3 showing peak list, signal-to-noise ratio (S/N), elemental composition, and assignment with mass errors (ppm) of the $[M+H]^+$ dBET1 compound by UVPD MS/MS, calibration points are marked by an asterisk (✱).

Measured *m/z*	S/N	Elemental Composition	Theoretical *m/z*	Assignment Error (ppm)	Assignment	
138.994606	47.5	C_7H_4ClO	138.994519	-0.625		
150.037261	47.3	C_8H_8NS	150.037197	-0.428	C16C20C23	
162.037239	35.3	C_9H_8NS	162.037197	-0.260	C3C17C23	
163.032483	42.2	$C_8H_7N_2S$	163.032446	-0.228	C15C21C24	
176.034219	130.2	$C_9H_6NO_3$	176.034220	0.006	C96C6'	✱
177.048101	146.5	$C_9H_9N_2S$	177.048096	-0.027	C12C24C25	
204.058960	230.9	$C_{10}H_{10}N_3S$	204.058995	0.173	C3C25	
205.076005	15.5	$C_{14}H_9N_2$	205.076025	0.100	C2C15C22C29	
214.068487	19.3	$C_{13}H_{12}NS$	214.068497	0.047	[C17C22C24+H_2]	
218.074598	779.3	$C_{11}H_{12}N_3S$	218.074645	0.218	C2C25	
220.090250	214.4	$C_{11}H_{14}N_3S$	220.090295	0.204	[C2C25+H_2]	
223.032393	21.0	$C_{13}H_7N_2S$	223.032446	0.239	C3C13C13C15C22	
233.018571	54.8	$C_{13}H_{10}ClS$	233.018626	0.235	C17C20	
237.048061	32.5	$C_{14}H_9N_2S$	237.048096	0.146	[C13C14C22C24-H]	
237.060618	18.2	$C_{15}H_{11}NS$	237.060672	0.227		
239.063684	97.5	$C_{14}H_{11}N_2S$	239.063746	0.259	C13C14C22C24	
245.085471	54.1	$C_{12}H_{13}N_4S$	245.085544	0.296	C2C21	
246.069499	1859.1	$C_{12}H_{12}N_3OS$	246.069560	0.249	C1C25	
248.010895	24.1	$C_{12}H_7ClNO_3$	248.010897	0.010		
248.029492	36.8	$C_{13}H_{11}ClNS$	248.029525	0.134	C17C24	
254.060476	18.8	$C_{15}H_{11}ClN_2$	254.060527	0.201	C3C29C37	
258.001232	33.8	$C_{13}H_7ClN_2S$	258.001299	0.259	C3C13C13C15	
272.029490	70.0	$C_{15}H_{11}ClNS$	272.029525	0.129		
272.072595	69.2	$C_{13}H_{12}N_4OS$	272.072634	0.144	C1C21	
273.024730	168.5	$C_{14}H_{10}ClN_2S$	273.024774	0.162	C13C20C33	
274.045141	261.2	$C_{15}H_{13}ClNS$	274.045175	0.123	C14C20	
275.021772	49.6	$C_{13}H_8ClN_2O_3$	275.021796	0.086		
275.102587	47.9	$C_{14}H_{15}N_2O_4$	275.102633	0.168	C99C4'	
276.042170	77.0	$C_{14}H_{11}ClNO_3$	276.042197	0.097		
279.082419	29.0	$C_{16}H_{13}N_3S$	279.082470	0.183	C20C22C24	
286.045065	30.6	$C_8H_9ClN_7O_3$	286.044991	-0.259		
286.082110	16.8	$C_{14}H_{12}N_3O_4$	286.082232	0.426	[C99C1'-H_4]	
287.040380	55.7	$C_{15}H_{12}ClN_2S$	287.040424	0.154	[C14C24-H_2]	
287.066205	981.0	$C_{14}H_{11}N_2O_5$	287.066248	0.149	C8'	
289.056021	470.2	$C_{15}H_{14}ClN_2S$	289.056074	0.183	C14C24	
299.040421	104.3	$C_{16}H_{12}ClN_2S$	299.040424	0.010	[C2C15-H]	✱
300.024404	74.7	$C_{16}H_{11}ClNOS$	300.024439	0.117	[C1C17-H_2]	
300.035528	22.8	$C_{15}H_{11}ClN_3S$	300.035673	0.484	C12C13C24	
300.060792	38.1	$C_{17}H_{15}ClNS$	300.060825	0.111		
301.037481	26.8	$C_{15}H_{10}ClN_2O_3$	301.037446	-0.117		
301.056059	47.2	$C_{16}H_{14}ClN_2S$	301.056074	0.050	C2C15	
302.021494	25.1	$C_{15}H_9ClNO_4$	302.021462	-0.105		
304.090353	15.1	$C_{18}H_{14}N_3S$	304.090295	-0.192		
305.098110	207.1	$C_{18}H_{15}N_3S$	305.098120	0.033	[C2C12-H_2]	
311.040433	18.6	$C_{17}H_{12}ClN_2S$	311.040424	-0.028	[C3C37-H_2]	
312.048248	39.6	$C_{17}H_{13}ClN_2S$	312.048249	0.003	[C3C37-H]	
313.055993	29.7	$C_{17}H_{14}ClN_2S$	313.056074	0.259	C3C37	
314.051316	435.0	$C_{16}H_{13}ClN_3S$	314.051323	0.023	C20C24	
314.077104	13.9	$C_{15}H_{12}N_3O_5$	314.077147	0.138	[C6'-H_2O]	
315.061152	228.3	$C_{15}H_{11}N_2O_6$	315.061162	0.033	C7'	
319.101135	20.6	$C_{18}H_{15}N_4S$	319.101194	0.186	[C2C22-H]	
320.108934	22.7	$C_{18}H_{16}N_4S$	320.109019	0.267	C2C22	

321.116867	22.9	$C_{18}H_{17}N_4S$	321.116844	-0.070		
324.035660	17.5	$C_{17}H_{11}ClN_3S$	324.035673	0.041	C1C11C36	
327.035365	218.2	$C_{17}H_{12}ClN_2OS$	327.035338	-0.083	C1C15	
327.046578	15.4	$C_{16}H_{12}ClN_4S$	327.046572	-0.017	C3C13	
327.071723	249.1	$C_{18}H_{16}ClN_2S$	327.071724	0.002	C11C40	
328.066949	28.8	$C_{17}H_{15}ClN_3S$	328.066973	0.075		
329.062227	90.0	$C_{16}H_{14}ClN_4S$	329.062222	-0.014	C24	*
329.087318	24.1	$C_{18}H_{18}ClN_2S$	329.087374	0.170	C17C40	
330.072151	16.3	$C_{15}H_{12}N_3O_6$	330.072062	-0.269	C6'	
331.059239	28.7	$C_{15}H_{12}ClN_4O_3$	331.059244	0.016		
338.051324	39.4	$C_{18}H_{13}ClN_3S$	338.051323	-0.002	[C2C12-H_4]	
339.059069	20.0	$C_{18}H_{14}ClN_3S$	339.059148	0.232	[C2C12-H_3]	
340.067002	406.7	$C_{18}H_{15}ClN_3S$	340.066973	-0.084	[C2C12-H_2]	
341.062217	3899.1	$C_{17}H_{14}ClN_4S$	341.062222	0.015	C3	
342.046271	177.1	$C_{17}H_{13}ClN_3OS$	342.046237	-0.098	C1C36	
343.077885	28.2	$C_{17}H_{16}ClN_4S$	343.077872	-0.037	[C3+H_2]	
348.103956	16.4	$C_{19}H_{16}N_4OS$	348.103934	-0.065	C1C22	
350.092936	16.1	$C_{19}H_{15}ClN_4O$	350.092890	-0.131	C1C34	
355.077904	631.9	$C_{18}H_{16}ClN_4S$	355.077872	-0.091	[C2-H_2]	
357.093607	115.5	$C_{18}H_{18}ClN_4S$	357.093522	-0.239	C2	
359.090719	35.1	$C_{17}H_{16}ClN_4O_3$	359.090545	-0.484	C10C15C18'	
365.062225	24.8	$C_{19}H_{14}ClN_4S$	365.062222	-0.009		
366.046337	95.3	$C_{19}H_{13}ClN_3OS$	366.046237	-0.274	[C1-NH_3]	
368.061979	16.9	$C_{19}H_{15}ClN_3OS$	368.061887	-0.250	C1C12	
370.113957	40.4	$C_{20}H_{21}ClN_3S$	370.113923	-0.092		
382.088871	183.9	$C_{19}H_{17}ClN_5S$	382.088771	-0.261	C1C11	
383.072701	16355.0	$C_{19}H_{16}ClN_4OS$	383.072786	0.222	C1	
385.150694	65.9	$C_{19}H_{21}N_4O_5$	385.150646	-0.126	[C1'-OH]	
386.134690	546.2	$C_{19}H_{20}N_3O_6$	386.134662	-0.071	C4'	
403.161178	1499.0	$C_{19}H_{23}N_4O_6$	403.161211	0.081	C1'	
437.119259	24.1	$C_{26}H_{19}N_3O_2S$	437.119249	-0.024		
453.162268	47.4	$C_{23}H_{26}ClN_6S$	453.162270	0.004	C7C11	*
471.172458	15.3	$C_{23}H_{28}ClN_6OS$	471.172835	0.801	[C7+H_2]	
785.227945	66210.0	$C_{38}H_{38}ClN_8O_7S$	785.226721	-1.559	[M+H]	
			Abs mean error	0.165		
			Mean std dev	0.141		

Table S4 showing peak list, signal-to-noise ratio (S/N), elemental composition, and assignment with mass errors (ppm) of the $[M+Li]^+$ dBET1 compound by CID MS/MS, calibration points are marked by an asterisk (✱).

Measured *m/z*	S/N	Elemental Composition	Theoretical *m/z*	Assignment Error (ppm)	Assignment	
293.074426	10.8	$C_{14}H_{10}N_2O_5Li$	293.074426	0.000	C8'	✱
321.069399	10.8	$C_{15}H_{10}N_2O_6Li$	321.069341	-0.180	[C7'-H]	
338.095857	343.9	$C_{15}H_{13}N_3O_6Li$	338.095890	0.096	[C6'+H]	
343.059227	38.9	$C_{16}H_{12}ClN_4O_3$	343.059244	0.050	[C10C13C15C21-H_6]	
344.062598	45.6	$C_{14}H_{10}N_5O_6$	344.062559	-0.114		
345.055053	39.8	$C_{17}H_9N_6OS$	345.055307	0.735		
348.078224	45.3	$C_{17}H_{14}ClN_4SLi$	348.078225	0.002	[C3+H]	
350.075399	6.7	$C_{16}H_{12}ClN_4O_3Li$	350.075248	-0.432	[C10C13C15C21-H_6]	
355.077851	119.9	$C_{18}H_{16}ClN_4S$	355.077872	0.060	[C2+H]	
361.086024	423.0	$C_{18}H_{15}ClN_4SLi$	361.086050	0.072	[C2-H_2]	
361.113547	31.9	$C_{19}H_{22}ClN_2OS$	361.113589	0.115	[C1C37+H_6]	
363.101675	369.4	$C_{18}H_{17}ClN_4SLi$	363.101700	0.068	C2	
364.111505	30.7	$C_{17}H_{15}N_3O_6Li$	364.111540	0.096	C40	
365.098728	101.8	$C_{17}H_{15}ClN_4O_3Li$	365.098723	-0.013	C10C15C18'	
367.121702	11.7	$C_{19}H_{21}ClN_2OSLi$	367.121767	0.176	C6C17C46	
378.127168	174.6	$C_{18}H_{17}N_3O_6Li$	378.127190	0.057	C41'	
379.096602	198.0	$C_{18}H_{17}ClN_4OSLi$	379.096615	0.035	[C1C13+H_2]	
381.112267	62.9	$C_{18}H_{19}ClN_4OSLi$	381.112265	-0.006	[C1C13+H_4]	
383.072759	15.6	$C_{19}H_{16}ClN_4OS$	383.072786	0.070	[C1+H]	
385.069831	79.3	$C_{18}H_{14}ClN_4O_4$	385.069809	-0.058		
386.134686	12.2	$C_{19}H_{20}N_3O_6$	386.134662	-0.063	C4'	
389.080963	31.0	$C_{19}H_{15}ClN_4OSLi$	389.080965	0.005	C1	
390.127182	152.3	$C_{19}H_{17}N_3O_6Li$	390.127190	0.020	[C4'-H_2]	
391.158863	12.0	$C_{19}H_{20}N_4O_5Li$	391.158825	-0.096	[C1'-OH]	
392.142839	235.6	$C_{19}H_{19}N_3O_6Li$	392.142840	0.002	[C4'-H]	✱
394.132660	365.4	$C_{20}H_{22}ClN_3OSLi$	394.132666	0.016	C7C17	
404.117011	132.6	$C_{21}H_{20}ClN_3OSLi$	404.117016	0.012	[C6C15-H_2]	
405.138091	13.3	$C_{19}H_{18}N_4O_6Li$	405.138089	-0.004	[C1'-H_3]	
406.107241	6.3	$C_{19}H_{18}ClN_5OSLi$	406.107514	0.673	C4	
406.132622	23.3	$C_{21}H_{22}ClN_3OSLi$	406.132666	0.108	C6C15	
409.169390	3123.1	$C_{19}H_{22}N_4O_6Li$	409.169389	-0.002	[C1'+H]	✱
419.115386	15.9	$C_{20}H_{19}ClN_5OSLi$	419.115339	-0.113	C41	
419.153747	46.5	$C_{20}H_{20}N_4O_6Li$	419.153739	-0.018	[C2'-OH]	
428.119375	35.6	$C_{22}H_{23}ClN_3O_2S$	428.119402	0.064	[C9C17-H_2]	
432.123156	35.2	$C_{21}H_{20}ClN_5OSLi$	432.123164	0.017	C40'	
435.148634	17428.0	$C_{20}H_{20}N_4O_7Li$	435.148654	0.047	C2'	
437.164320	2887.0	$C_{20}H_{22}N_4O_7Li$	437.164304	-0.036	[C2'+H_2]	
446.138836	103.9	$C_{22}H_{22}ClN_5OSLi$	446.138814	-0.050	C5	
453.138325	11.0	$C_{23}H_{24}ClN_5OS$	453.138461	0.300	C6	
459.170602	12.8	$C_{23}H_{25}ClN_6SLi$	459.170449	-0.334	C7C11	
477.181071	226.9	$C_{23}H_{27}ClN_6OSLi$	477.181013	-0.123	[C7+H_2]	
487.165418	676.8	$C_{24}H_{25}ClN_6OSLi$	487.165363	-0.113	[C8C11-H_2]	
489.181056	510.1	$C_{24}H_{27}ClN_6OSLi$	489.181013	-0.088	C8C11	
490.202181	80.8	$C_{22}H_{25}N_7O_6Li$	490.202086	-0.194	C18C20C83	
503.160339	27.8	$C_{24}H_{25}ClN_6O_2SLi$	503.160278	-0.121	C8	
503.186263	22.0	$C_{31}H_{28}LiO_4S$	503.186285	0.044		
505.175990	40.6	$C_{24}H_{27}ClN_6O_2SLi$	505.175928	-0.122	[C8+H]	
511.167810	176.6	$C_{25}H_{28}ClN_6O_2S$	511.167749	-0.120	[C9+H]	
514.202080	22.6	$C_{24}H_{25}N_7O_6Li$	514.202086	0.011	C11C50C51	
517.175995	76.6	$C_{25}H_{27}ClN_6O_2SLi$	517.175928	-0.130	[C9-H_2]	
524.186497	71.1	$C_{25}H_{23}N_7O_6Li$	524.186436	-0.117	[C11C50-H_4]	

531.161753	32.0	$C_{26}H_{25}ClLiN_4O_6$	531.161717	-0.068		
542.197069	652.4	$C_{25}H_{25}N_7O_7Li$	542.197001	-0.126	[C50-H_2]	
641.180967	612.8	$C_{32}H_{31}ClN_4O_6SLi$	641.180738	-0.358	[C17C83+H_2]	
654.231871	74.1	$C_{31}H_{33}N_7O_7SLi$	654.231672	-0.304	C25	
681.175869	336.2	$C_{34}H_{31}ClN_4O_7SLi$	681.175653	-0.318	[C17-H_3CN]	
708.186622	7764.4	$C_{35}H_{32}ClN_5O_7SLi$	708.186552	-0.099	[C17-H_2]	*
709.190046	2875.4	$C_{36}H_{27}ClN_8O_6Li$	709.189663	-0.540	[C29-O_2H][C29-H_4]	
750.208297	32.0	$C_{36}H_{34}ClLiN_7O_7S$	750.208350	0.070		
757.247043	20.7	$C_{38}H_{35}ClN_8O_7Li$	757.247178	0.179	[C34-H_2]	
758.255742	13.9	$C_{38}H_{36}ClN_8O_7Li$	758.255003	-0.974	[C34-H]	
763.228501	21.1	$C_{38}H_{37}ClN_6O_7SLi$	763.228751	0.327	C37	
773.224179	34.4	$C_{38}H_{35}ClN_8O_6SLi$	773.224335	0.201	[M-OH_2]	
774.208316	7.4	$C_{38}H_{34}ClN_7O_7SLi$	774.208350	0.044	[M-NH_3]	
791.234692	14657.9	$C_{38}H_{37}ClN_8O_7SLi$	791.234899	0.262	[M+Li]	
			Abs mean error	0.141		
			Mean std dev	0.180		

Table S5 showing peak list, signal-to-noise ratio (S/N), elemental composition, and assignment with mass errors (ppm) of the [M+Li]+ dBET1 compound by IRMPD MS/MS, calibration points are marked by an asterisk (✱).

Measured *m/z*	S/N	Elemental Composition	Theoretical *m/z*	Assignment Error (ppm)	Assignment	
101.034576	395.0	$C_3H_5N_2O_2$	101.034554	-0.215	[C80C95+H_3]	
105.099877	108.4	$C_5H_{10}N_2Li$	105.099853	-0.229	C7C11C2'	
106.083895	154.8	C_5H_9NOLi	106.083869	-0.244	C6C2'	
107.966831	317.3	C_2H_3ClNS	107.966924	0.864		
121.094792	20.5	$C_5H_{10}N_2OLi$	121.094768	-0.202	C7C2'	
123.110435	805.5	$C_5H_{12}N_2OLi$	123.110418	-0.142	[C7C2'+H_2]	
131.079146	32.5	$C_6H_8N_2OLi$	131.079118	-0.215	[C1C37C47'+H_2]	
133.094791	414.2	$C_6H_{10}N_2OLi$	133.094768	-0.172	C8C11C2'	
135.110429	3018.6	$C_6H_{12}N_2OLi$	135.110418	-0.081	C7C3'	✱
139.036583	51.8	$C_8H_4O_2Li$	139.036584	0.009	C98C7'	
150.037233	14.8	C_8H_8NS	150.037197	-0.237	[C3C17C21+H]	
155.081522	554.5	$C_7H_{11}N_2O_2$	155.081504	-0.115	C9C2'	
161.089699	115.1	$C_7H_{10}N_2O_2Li$	161.089682	-0.103	[C9C2'-H]	
163.105368	16.4	$C_7H_{12}N_2O_2Li$	163.105332	-0.219	[C9C2'+H]	
164.113175	10.7	$C_7H_{13}N_2O_2Li$	164.113157	-0.107	[C9C2'+H_2]	
165.073381	68.9	$C_7H_{10}LiO_4$	165.073364	-0.105		
167.031519	37.4	$C_9H_4O_3Li$	167.031499	-0.122	[C96C7'-H]	
170.042423	28.1	$C_8H_5NO_3Li$	170.042398	-0.146	C84C90	
171.085285	55.7	$C_7H_8LiN_4O$	171.085266	-0.112	C21C36C40C18'	
176.034242	163.1	$C_9H_6NO_3$	176.034220	-0.126	C90C97'	
181.047145	64.3	$C_{10}H_6O_3Li$	181.047149	0.020	[C95C7'-O]	
182.042425	31.2	$C_9H_5NO_3Li$	182.042398	-0.147	C96C6'	
183.083937	126.8	$C_7H_{12}LiO_5$	183.083928	-0.048		
184.058041	8.1	$C_9H_7NO_3Li$	184.058048	0.036	[C99C8'+H_2]	
186.132552	14.7	$C_8H_{13}LiN_5$	186.132550	-0.009	[C40C18'C20'+H_2]	
189.092114	37.4	$C_{10}H_{14}LiOS$	189.091991	-0.652		
192.063183	29.1	$C_{11}H_7NO_2Li$	192.063133	-0.261	[C92C10'-H_2]	
193.072021	98.2	$C_8H_9N_4O_2$	193.072002	-0.099		
198.132576	51.8	$C_9H_{13}LiN_5$	198.132550	-0.132		
204.124394	105.5	$C_{10}H_{14}N_5$	204.124372	-0.110	C6C11C8'C20'	
207.110450	231.4	$C_{12}H_{12}N_2OLi$	207.110418	-0.155	C22C26C40	
208.058080	40.8	$C_{11}H_7NO_3Li$	208.058048	-0.156	[C93C8'-H]	
210.132571	63.7	$C_{10}H_{13}N_5Li$	210.132550	-0.101	[C6C11C8'C20'-H]	
216.143139	51.8	$C_9H_{15}LiN_5O$	216.143115	-0.111		
218.074669	85.0	$C_{11}H_{12}N_3S$	218.074645	-0.111	[C2C25+H]	
220.058112	7.2	$C_{12}H_7NO_3Li$	220.058048	-0.292	C90C10'	
222.073741	23.0	$C_{12}H_9NO_3Li$	222.073698	-0.195	[C90C10'+H_2]	
225.094522	129.2	$C_9H_{14}LiO_6$	225.094493	-0.127		
232.105714	80.9	$C_{13}H_{11}LiN_3O$	232.105667	-0.202	C4C15C34C39	
237.084635	72.1	$C_{12}H_{10}N_2O_3Li$	237.084597	-0.161	[C79-OH_2]	
238.055973	34.5	$C_{14}H_{10}N_2S$	238.055921	-0.218	C13C14C22C24	
238.127499	95.2	$C_{11}H_{13}N_5OLi$	238.127465	-0.142	C5C45C48	
244.119311	87.4	$C_{12}H_{14}N_5O$	244.119287	-0.100	C5C21C45	
246.069573	198.6	$C_{12}H_{12}N_3OS$	246.069560	-0.054	C15C21C41	
247.068966	13.0	$C_{13}H_8N_2O_3Li$	247.068947	-0.077	[C10'-OH]	
249.084635	58.4	$C_{13}H_{10}N_2O_3Li$	249.084597	-0.151	[C99C41'-OH]	
250.068664	17.3	$C_{13}H_9NO_4Li$	250.068613	-0.204	C95C41'	
251.100294	12.0	$C_{13}H_{12}N_2O_3Li$	251.100247	-0.187	[C98C2'-H_2]	
253.079559	41.9	$C_{12}H_{10}N_2O_4Li$	253.079512	-0.185	C79	
259.068950	6.8	$C_{14}H_8N_2O_3Li$	259.068947	-0.011	[C95C4'-OH][C95C4'-H_4]	
262.129881	295.3	$C_{12}H_{16}N_5O_2$	262.129851	-0.114	C8C12C21C45	

265.079518	22.9	$C_{13}H_{10}N_2O_4Li$	265.079512	-0.021	[C99C41'-H_2]	
268.138057	68.2	$C_{12}H_{15}N_5O_2Li$	268.138030	-0.102	[C8C12C21C45-H]	
272.029617	11.1	$C_{15}H_{11}ClNS$	272.029525	-0.340	[C2C17-H_2]	
274.045235	208.7	$C_{15}H_{13}ClNS$	274.045175	-0.221	C2C17	
277.079550	41.0	$C_{14}H_{10}N_2O_4Li$	277.079512	-0.137	[C9'-OH]	
278.063585	7.2	$C_{14}H_9NO_5Li$	278.063527	-0.208	C85C8'	
278.087362	10.9	$C_{14}H_{11}N_2O_4Li$	278.087337	-0.091	[C99C4'-H_2]	
280.053367	5698.9	$C_{15}H_{12}ClNSLi$	280.053353	-0.049	C14C20	
280.066622	71.9	$C_{13}H_9N_2O_5Li$	280.066601	-0.077	[C9'+H]	
281.074452	1120.4	$C_{13}H_{10}N_2O_5Li$	281.074426	-0.093	[C9'+H_2]	
281.110869	41.1	$C_{14}H_{14}N_2O_4Li$	281.110812	-0.202	C99C4'	
286.045241	28.9	$C_{16}H_{13}ClNS$	286.045175	-0.231	C1C17C11	
287.066299	79.9	$C_{14}H_{11}N_2O_5$	287.066248	-0.179	[C8'+H]	
291.131592	6.6	$C_{16}H_{16}N_2O_3Li$	291.131547	-0.155	[C93C4'-OH]	
293.074391	19211.8	$C_{14}H_{10}N_2O_5Li$	293.074426	0.118	C8'	
299.040492	250.3	$C_{16}H_{12}ClN_2S$	299.040424	-0.229	[C2C15-H]	
300.024520	117.9	$C_{16}H_{11}ClNOS$	300.024439	-0.270	[C1C17-H]	
304.092176	168.9	$C_{17}H_{19}ClNS$	304.092125	-0.168	[C1C11C51+H_4]	
307.064298	41.6	$C_{16}H_{13}ClN_2SLi$	307.064252	-0.151	C2C15	
308.048333	28.2	$C_{16}H_{12}ClNOSLi$	308.048268	-0.212	C1C17	
310.072137	234.8	$C_{15}H_{13}LiNO_4S$	310.071984	-0.493		
318.142466	11.1	$C_{17}H_{17}N_3O_3Li$	318.142446	-0.062	[C82C4'-O_2]	
320.048321	44.6	$C_{17}H_{12}ClNOSLi$	320.048268	-0.165	[C1C44C18'-H_2]	
320.085414	52.6	$C_{15}H_{11}N_3O_5Li$	320.085325	-0.278	[C6'-H_2O]	
321.069378	117.3	$C_{15}H_{10}N_2O_6Li$	321.069341	-0.116	[C7'-H]	
323.059231	758.9	$C_{16}H_{13}ClN_2OSLi$	323.059167	-0.197	C4C17	
324.116708	21.5	$C_{15}H_{15}N_3O_5Li$	324.116625	-0.255	[C49C6'+H_3]	
333.079985	11.0	$C_{18}H_{15}ClN_2SLi$	333.079902	-0.249	C11C40	
334.137429	33.3	$C_{17}H_{17}N_3O_4Li$	334.137361	-0.204	[C94C3'-OH]	
338.095885	4798.9	$C_{15}H_{13}N_3O_6Li$	338.095890	0.014	[C6'+H]	*
341.062318	13.0	$C_{17}H_{14}ClN_4S$	341.062222	-0.283	[C3+H]	
342.070209	9.2	$C_{17}H_{15}ClN_4S$	342.070047	-0.472	[C3+H_2]	
343.059324	226.2	$C_{16}H_{12}ClN_4O_3$	343.059244	-0.232	[C10C13C15C21-H_6]	
348.054488	27.6	$C_{17}H_{12}ClN_3OSLi$	348.054416	-0.208	C1C36	
348.078317	41.1	$C_{17}H_{14}ClN_4SLi$	348.078225	-0.265	[C3+H]	
348.153070	7.9	$C_{18}H_{19}N_3O_4Li$	348.153011	-0.170	[C93C3'-OH]	
350.075433	7.6	$C_{16}H_{12}ClN_4O_3Li$	350.075248	-0.530	[C10C13C15C21-H_6]	
350.095952	34.3	$C_{16}H_{13}N_3O_6Li$	350.095890	-0.178	C5'	
354.093915	118.4	$C_{15}H_{14}ClLiN_5O_3$	354.093972	0.160		
355.077960	196.5	$C_{18}H_{16}ClN_4S$	355.077872	-0.248	[C2+H]	
357.045980	81.8	$C_{15}H_{11}ClLiN_2O_6$	357.046019	0.110		
357.118760	144.6	$C_{17}H_{19}ClLiN_2O_4$	357.118790	0.083		
361.086115	1536.7	$C_{18}H_{15}ClN_4SLi$	361.086050	-0.179	[C2-H_2]	
361.113644	167.2	$C_{19}H_{22}ClN_2OS$	361.113589	-0.151	[C1C37+H_6]	
363.101765	996.3	$C_{18}H_{17}ClN_4SLi$	363.101700	-0.178	C2	
365.098820	94.5	$C_{17}H_{15}ClN_4O_3Li$	365.098723	-0.266	C10C15C18'	
367.121864	139.3	$C_{19}H_{21}ClN_2OSLi$	367.121767	-0.263	C6C17C46	
371.098023	51.6	$C_{20}H_{20}ClN_2OS$	371.097939	-0.227	C5C17	
377.106230	430.4	$C_{20}H_{19}ClN_2OSLi$	377.106117	-0.301	[C5C17-H]	
378.127233	13.4	$C_{18}H_{17}N_3O_6Li$	378.127190	-0.114	C41'	
385.069898	72.4	$C_{18}H_{14}ClN_4O_4$	385.069809	-0.231		
386.134641	12.4	$C_{19}H_{20}N_3O_6$	386.134662	0.055	C4'	
389.081049	250.1	$C_{19}H_{15}ClN_4OSLi$	389.080965	-0.216	C1	
390.127295	114.6	$C_{19}H_{17}N_3O_6Li$	390.127190	-0.270	[C4'-H_2]	
391.158978	28.5	$C_{19}H_{20}N_4O_5Li$	391.158825	-0.392	[C1'-OH]	
392.142946	659.1	$C_{19}H_{19}N_3O_6Li$	392.142840	-0.270	[C4'-H]	
394.132724	1721.8	$C_{20}H_{22}ClN_3OSLi$	394.132666	-0.148	C7C17	

403.161291	54.5	$C_{19}H_{23}N_4O_6$	403.161211	-0.197	C1'	
404.117085	2275.0	$C_{21}H_{20}ClN_3OSLi$	404.117016	-0.171	[C6C15-H_2]	
406.132790	395.9	$C_{21}H_{22}ClN_3OSLi$	406.132666	-0.305	C6C15	
409.169398	4548.4	$C_{19}H_{22}N_4O_6Li$	409.169389	-0.022	[C1'+H]	*
419.153886	65.6	$C_{20}H_{20}N_4O_6Li$	419.153739	-0.350	[C2'-OH]	
428.119535	529.1	$C_{22}H_{23}ClN_3O_2S$	428.119402	-0.310	[C9C17-H_2]	
435.148576	40307.2	$C_{20}H_{20}N_4O_7Li$	435.148654	0.180	C2'	
437.164299	17141.1	$C_{20}H_{22}N_4O_7Li$	437.164304	0.012	[C2'+H_2]	
464.175345	133.0	$C_{21}H_{23}LiN_5O_7$	464.175203	-0.306		
475.146753	31.7	$C_{22}H_{21}ClLiN_6O_4$	475.146736	-0.035		
477.181205	49.8	$C_{23}H_{27}ClN_6OSLi$	477.181013	-0.402	[C7+H_2]	
487.165507	412.1	$C_{24}H_{25}ClN_6OSLi$	487.165363	-0.297	[C8C11-H_2]	
489.181155	376.0	$C_{24}H_{27}ClN_6OSLi$	489.181013	-0.290	C8C11	
490.202305	294.3	$C_{22}H_{25}N_7O_6Li$	490.202086	-0.446	C18C20C83	
503.160528	44.0	$C_{24}H_{25}ClN_6O_2SLi$	503.160278	-0.497	C8	
505.176095	24.5	$C_{24}H_{27}ClN_6O_2SLi$	505.175928	-0.331	[C8+H]	
511.167956	139.9	$C_{25}H_{28}ClN_6O_2S$	511.167749	-0.406	[C9+H]	
517.176129	129.4	$C_{25}H_{27}ClN_6O_2SLi$	517.175928	-0.389	[C9-H_2]	
519.191622	12.1	$C_{25}H_{29}ClN_6O_2SLi$	519.191578	-0.085	C9	
524.186625	55.4	$C_{25}H_{23}N_7O_6Li$	524.186436	-0.360	[C11C50-H_4]	
542.197202	167.6	$C_{25}H_{25}N_7O_7Li$	542.197001	-0.371	[C50-H_2]	
554.149016	20.0	$C_{29}H_{24}N_5O_5S$	554.149266	0.451		
569.097409	44.7	$C_{33}H_{19}ClLiO_7$	569.097385	-0.041		
572.987326	40.1	$C_{27}H_7ClLiN_4O_7S$	572.987852	0.918		
576.063293	37.1	$C_{36}H_{15}ClNO_5$	576.063327	0.059		
585.086675	35.7	$C_{32}H_{17}N_4O_6S$	585.086332	-0.587		
586.087660	106.1	$C_{35}H_{21}ClNO_4S$	586.087433	-0.387		
610.043738	22.3	$C_{34}H_{13}ClN_3O_7$	610.043654	-0.138		
641.181015	88.1	$C_{32}H_{31}ClN_4O_6SLi$	641.180738	-0.433	[C17C83+H_2]	
652.028682	28.1	$C_{33}H_7LiN_7O_7S$	652.028222	-0.706		
654.232039	28.1	$C_{31}H_{33}N_7O_7SLi$	654.231672	-0.561	C25	
656.189704	50.5	$C_{33}H_{27}LiN_7O_6S$	656.189808	0.158		
681.175898	3026.4	$C_{34}H_{31}ClN_4O_7SLi$	681.175653	-0.360	[C17-H_3CN]	
708.186614	25532.0	$C_{35}H_{32}ClN_5O_7SLi$	708.186552	-0.088	[C17-H_2]	*
709.189889	9727.9	$C_{36}H_{27}ClN_8O_6Li$	709.189663	-0.319	[C29-O_2H]	
721.032685	45.8	$C_{37}H_{14}ClN_6O_7S$	721.032772	0.121		
757.247555	14.7	$C_{38}H_{35}ClN_8O_7Li$	757.247178	-0.499	[C34-H_2]	
773.224866	16.0	$C_{38}H_{35}ClN_8O_6SLi$	773.224335	-0.687	[M-OH_2]	
774.208987	18.1	$C_{38}H_{34}ClN_7O_7SLi$	774.208350	-0.822	[M-NH_3]	
789.219870	19.8	$C_{38}H_{35}ClN_8O_7SLi$	789.219249	-0.787	[M-H_2]	
791.234842	28076.3	$C_{38}H_{37}ClN_8O_7SLi$	791.234899	0.072	[M+Li]	
			Abs mean error	0.202		
			Mean std dev	0.119		

Table S6 showing peak list, signal-to-noise ratio (S/N), elemental composition, and assignment with mass errors (ppm) of the $[M+Li]^+$ dBET1 compound by UVPD MS/MS, calibration points are marked by an asterisk (✱).

Measured *m/z*	S/N	Elemental Composition	Theoretical *m/z*	Assignment Error (ppm)	Assignment	
105.099863	7.9	$C_5H_{10}N_2Li$	105.099853	-0.097	C7C11C2'	
106.083873	33.6	C_5H_9NOLi	106.083869	-0.041	C6C2'	
118.047487	12.0	$C_5H_5NO_2Li$	118.047483	-0.036	C99'	
121.094774	8.6	$C_5H_{10}N_2OLi$	121.094768	-0.047	C7C2'	
123.110417	276.8	$C_5H_{12}N_2OLi$	123.110418	0.008	[C7C2'+H_2]	
133.094765	118.5	$C_6H_{10}N_2OLi$	133.094768	0.021	C8C11C2'	
135.110415	407.6	$C_6H_{12}N_2OLi$	135.110418	0.021	C7C3'	
137.126064	12.9	$C_6H_{14}N_2OLi$	137.126068	0.030	C7C3'/C9C1'	
155.081500	302.2	$C_7H_{11}N_2O_2$	155.081504	0.025	C9C2'	
161.089682	28.7	$C_7H_{10}N_2O_2Li$	161.089682	0.002	[C9C2'-H]	
163.105334	24.2	$C_7H_{12}N_2O_2Li$	163.105332	-0.011	[C9C2'+H]	
164.113153	33.8	$C_7H_{13}N_2O_2Li$	164.113157	0.022	[C9C2'+H_2]	
167.031498	35.2	$C_9H_4O_3Li$	167.031499	0.005	[C96C7'-H]	
169.034572	145.8	$C_8H_4NO_3Li$	169.034573	0.004	C99C9'	
170.042397	25.0	$C_8H_5NO_3Li$	170.042398	0.004	C84C90	
171.085266	32.0	$C_7H_8LiN_4O$	171.085266	0.002	C21C36C40C18'	
176.034221	14.1	$C_9H_6NO_3$	176.034220	-0.006	C90C97'	
182.042396	237.0	$C_9H_5NO_3Li$	182.042398	0.013	C96C6'	
185.041962	13.2	$C_{12}H_9S$	185.041948	-0.075	C17C20C32	
186.132553	43.6	$C_8H_{13}LiN_5$	186.132550	-0.018	[C40C18'C20'+H_2]	
192.063138	13.5	$C_{11}H_7NO_2Li$	192.063133	-0.025	[C92C10'-H_2]	
193.070937	7.7	$C_{11}H_8NO_2Li$	193.070958	0.109	[C92C10'-H]	
195.050223	231.3	$C_{10}H_6NO_3Li$	195.050223	0.001	C94C8'	
196.058045	71.4	$C_{10}H_7NO_3Li$	196.058048	0.017	C94C8'	
199.080175	26.7	$C_8H_8LiN_4O_2$	199.080180	0.025		
204.124364	39.9	$C_{10}H_{14}N_5$	204.124372	0.038	C6C11C8'C20'	
207.110446	9.2	$C_{12}H_{12}N_2OLi$	207.110418	-0.133	C22C26C40	
208.058051	71.5	$C_{11}H_7NO_3Li$	208.058048	-0.012	[C93C8'-H]	
209.065873	102.6	$C_{11}H_8NO_3Li$	209.065873	0.001	C93C8'	
210.037313	14.8	$C_{10}H_5NO_4Li$	210.037313	-0.001	C99C7'	
210.132557	28.4	$C_{10}H_{13}N_5Li$	210.132550	-0.032	[C6C11C8'C20'-H]	
218.074653	18.0	$C_{11}H_{12}N_3S$	218.074645	-0.038	[C2C25+H]	
220.090266	17.4	$C_{11}H_{14}N_3S$	220.090295	0.134	[C2C25+H_3]	
222.073697	113.4	$C_{12}H_9NO_3Li$	222.073698	0.003	[C90C10'+H_2]	
224.082823	267.3	$C_{11}H_{11}N_3SLi$	224.082823	0.000	C2C25	✱
225.090640	11.2	$C_{11}H_{12}N_3SLi$	225.090648	0.036	[C2C25+H]	
226.098465	26.9	$C_{11}H_{13}N_3SLi$	226.098473	0.037	[C2C25+H_2]	
233.018619	14.1	$C_{13}H_{10}ClS$	233.018626	0.032	[C47+H_2]	
235.034291	17.5	$C_{13}H_{12}ClS$	235.034276	-0.064	C47	
235.105321	20.8	$C_{13}H_{12}LiN_2O_2$	235.105332	0.046		
236.052878	18.3	$C_{12}H_7NO_4Li$	236.052963	0.361	C93C7'	
237.084589	13.2	$C_{12}H_{10}N_2O_3Li$	237.084597	0.035	[C79-OH_2]	
238.055909	16.5	$C_{14}H_{10}N_2S$	238.055921	0.052	C13C14C22C24	
238.127463	39.2	$C_{11}H_{13}N_5OLi$	238.127465	0.008	C5C45C48	
241.068262	33.1	$C_{11}H_4LiN_7$	241.068273	0.046		
244.119303	19.9	$C_{12}H_{14}N_5O$	244.119287	-0.066	C5C21C45	
246.069564	21.6	$C_{12}H_{12}N_3OS$	246.069560	-0.017	C15C21C41	
247.068980	7.6	$C_{13}H_8N_2O_3Li$	247.068947	-0.134	[C10'-OH]	
249.084577	48.2	$C_{13}H_{10}N_2O_3Li$	249.084597	0.079	[C99C41'-OH]	
250.068607	23.6	$C_{13}H_9NO_4Li$	250.068613	0.024	C95C41'	
251.093720	50.7	$C_{12}H_{12}N_4SLi$	251.093722	0.009	C2C21	
253.079538	19.1	$C_{12}H_{10}N_2O_4Li$	253.079512	-0.104	C79	

262.129847	76.5	$C_{12}H_{16}N_5O_2$	262.129851	0.016	C8C12C21C45	
267.095165	18.9	$C_{13}H_{12}N_2O_4Li$	267.095162	-0.013	[C99C41'+H]	
268.138027	12.2	$C_{12}H_{15}N_5O_2Li$	268.138030	0.012	[C8C12C21C45-H]	
272.029557	16.6	$C_4H_{10}N_5O_7S$	272.029545	-0.045		
274.045183	152.1	$C_{15}H_{13}ClNS$	274.045175	-0.029	C2C17	
277.079518	25.3	$C_{14}H_{10}N_2O_4Li$	277.079512	-0.020	[C9'-OH]	
278.087330	30.5	$C_{14}H_{11}N_2O_4Li$	278.087337	0.026	[C99C4'-H_2]	
279.088567	6.9	$C_{13}H_{12}N_4OSLi$	279.088637	0.252	[C1C21+H]	
280.053356	236.9	$C_{15}H_{12}ClNSLi$	280.053353	-0.011	C14C20	
280.066605	176.4	$C_{13}H_9N_2O_5Li$	280.066601	-0.015	[C9'+H]	
281.074427	476.5	$C_{13}H_{10}N_2O_5Li$	281.074426	-0.005	[C9'+H_2]	
281.110829	18.9	$C_{14}H_{14}N_2O_4Li$	281.110812	-0.060	C99C4'	
286.045191	10.5	$C_{16}H_{13}ClNS$	286.045175	-0.055	C1C17C11	
287.066269	35.7	$C_{14}H_{11}N_2O_5$	287.066248	-0.073	[C8'+H]	
293.061172	11.5	$C_{16}H_{13}ClNSLi$	293.061178	0.020	C1C17C11'	
293.074426	3013.0	$C_{14}H_{10}N_2O_5Li$	293.074426	0.002	C8'	*
294.106080	10.6	$C_{14}H_{13}N_3O_4Li$	294.106061	-0.065	[C99C1'-H_2]	
298.137403	20.7	$C_{14}H_{17}N_3O_4Li$	298.137361	-0.142	[C99C1'+H_2]	
299.040415	17.0	$C_{16}H_{12}ClN_2S$	299.040424	0.031	[C2C15-H]	
300.024469	17.4	$C_{16}H_{11}ClNOS$	300.024439	-0.101	[C1C17-H]	
304.092120	104.8	$C_{17}H_{19}ClNS$	304.092125	0.017		
306.044129	7.3	$C_{15}H_{10}ClN_3SLi$	306.043851	-0.909	C12C13C24	
307.064320	8.6	$C_{16}H_{13}ClN_2SLi$	307.064252	-0.221	C2C15	
320.048301	15.0	$C_{17}H_{12}ClNOSLi$	320.048268	-0.103	[C1C44C18'-H_2]	
320.059515	24.1	$C_{16}H_{12}ClN_3SLi$	320.059501	-0.043	C20C24	
320.085339	16.5	$C_{15}H_{11}N_3O_5Li$	320.085325	-0.044	[C6'-H_2O]	
321.069345	289.5	$C_{15}H_{10}N_2O_6Li$	321.069341	-0.013	C7'	
323.059154	50.8	$C_{16}H_{13}ClN_2OSLi$	323.059167	0.041	C4C17	
324.116627	126.6	$C_{15}H_{15}N_3O_5Li$	324.116625	-0.006	[C49C6'+H_3]	
333.079903	29.9	$C_{18}H_{15}ClN_2SLi$	333.079902	-0.004	C11C40	
335.070400	42.8	$C_{16}H_{13}ClN_4SLi$	335.070400	0.001	C24	
338.095893	951.4	$C_{15}H_{13}N_3O_6Li$	338.095890	-0.009	[C6'+H]	*
341.062229	14.6	$C_{17}H_{14}ClN_4S$	341.062222	-0.021	[C3+H]	
343.059278	35.2	$C_{16}H_{12}ClN_4O_3$	343.059244	-0.099	[C10C13C15C21-H_6]	
344.062605	33.5	$C_{18}H_5LiN_6O_2$	344.062853	0.720		
345.055069	25.2	$C_{21}H_{12}ClNO_2$	345.055108	0.114		
346.075215	15.8	$C_{18}H_{14}ClN_3SLi$	346.075151	-0.185	[C2C12-H_2]	
347.070423	24.5	$C_{17}H_{13}ClN_4SLi$	347.070400	-0.066	C3	
348.054466	8.2	$C_{17}H_{12}ClN_3OSLi$	348.054416	-0.144	C1C36	
348.078225	118.5	$C_{17}H_{14}ClN_4SLi$	348.078225	-0.001	[C3+H]	
348.153212	9.7	$C_{18}H_{19}N_3O_4Li$	348.153011	-0.579	[C93C3'-OH]	
349.086070	15.6	$C_{17}H_{15}ClN_4SLi$	349.086050	-0.058	[C3+H_2]	
350.075304	36.0	$C_{16}H_{12}ClN_4O_3Li$	350.075248	-0.159	[C10C13C15C21-H_6]	
350.095899	12.5	$C_{16}H_{13}N_3O_6Li$	350.095890	-0.026	C5'	
355.077861	70.9	$C_{18}H_{16}ClN_4S$	355.077872	0.032	[C2+H]	
361.086049	600.6	$C_{18}H_{15}ClN_4SLi$	361.086050	0.002	[C2-H_2]	*
362.093864	11.4	$C_{18}H_{16}ClN_4SLi$	362.093875	0.031	[C2+H]	
363.101706	272.2	$C_{18}H_{17}ClN_4SLi$	363.101700	-0.018	C2	
364.111505	49.9	$C_{17}H_{15}N_3O_6Li$	364.111540	0.097	C40	
364.147914	88.5	$C_{18}H_{19}N_3O_5Li$	364.147926	0.032	[C90C1'-H_2]	
365.098770	75.6	$C_{17}H_{15}ClN_4O_3Li$	365.098723	-0.128	C10C15C18'	
367.121761	17.6	$C_{19}H_{21}ClN_2OSLi$	367.121767	0.016	C6C17C46	
377.106123	21.3	$C_{20}H_{19}ClN_2OSLi$	377.106117	-0.016	[C5C17-H]	
378.127182	143.5	$C_{18}H_{17}N_3O_6Li$	378.127190	0.021	C41'	
385.069839	53.0	$C_{18}H_{14}ClN_4O_4$	385.069809	-0.079		
389.080984	94.9	$C_{19}H_{15}ClN_4OSLi$	389.080965	-0.050	C1	
390.127158	85.7	$C_{19}H_{17}N_3O_6Li$	390.127190	0.083	[C4'-H_2]	

391.158809	20.3	$C_{19}H_{20}N_4O_5Li$	391.158825	0.040	[C1'-OH]	
392.142837	230.9	$C_{19}H_{19}N_3O_6Li$	392.142840	0.007	C4'	*
394.132667	359.6	$C_{20}H_{22}ClN_3OSLi$	394.132666	-0.002	C7C17	
404.117019	117.9	$C_{21}H_{20}ClN_3OSLi$	404.117016	-0.007	[C6C15-H_2]	
405.138113	10.6	$C_{19}H_{18}N_4O_6Li$	405.138089	-0.059	[C1'-H_3]	
406.132628	29.6	$C_{21}H_{22}ClN_3OSLi$	406.132666	0.094	C6C15	
407.153767	52.0	$C_{19}H_{20}N_4O_6Li$	407.153739	-0.070	[C1'-H]	
409.169389	559.7	$C_{19}H_{22}N_4O_6Li$	409.169389	0.000	C1'	*
419.115299	50.8	$C_{20}H_{19}ClN_5OSLi$	419.115339	0.096	C41	
428.119342	20.5	$C_{22}H_{23}ClN_3O_2S$	428.119402	0.141	[C9C17-H_2]	
432.123138	85.7	$C_{21}H_{20}ClN_5OSLi$	432.123164	0.060	C40'	
435.148609	3316.2	$C_{20}H_{20}N_4O_7Li$	435.148654	0.103	C2'	
437.164282	185.6	$C_{20}H_{22}N_4O_7Li$	437.164304	0.051	[C2'+H_2]	
446.138768	135.2	$C_{22}H_{22}ClN_5OSLi$	446.138814	0.103	C5	
477.180887	30.8	$C_{23}H_{27}ClN_6OSLi$	477.181013	0.264	[C7+H_2]	
487.165261	66.8	$C_{24}H_{25}ClN_6OSLi$	487.165363	0.208	[C8C11-H_2]	
489.180919	35.4	$C_{24}H_{27}ClN_6OSLi$	489.181013	0.191	C8C11	
503.160350	13.3	$C_{24}H_{25}ClN_6O_2SLi$	503.160278	-0.143	C8	
517.175615	30.7	$C_{25}H_{27}ClN_6O_2SLi$	517.175928	0.606	[C9-H_2]	
680.202788	5.8	$C_{33}H_{32}ClN_7O_5SLi$	680.202871	0.122	[C99+H_2]	
708.186623	55.7	$C_{35}H_{32}ClN_5O_7SLi$	708.186552	-0.101	[C17-H_2]	*
709.189402	21.0	$C_{36}H_{27}ClN_8O_6Li$	709.189663	0.368	[C29-H_2O]	
720.233844	5.7	$C_{36}H_{36}ClN_7O_5SLi$	720.234171	0.453	C92	
756.265680	13.7	$C_{38}H_{37}N_8O_7SLi$	756.266047	0.485	C22	
791.233862	10412.8	$C_{38}H_{37}ClN_8O_7SLi$	791.234899	1.310	[M+Li]	
			Abs mean error	0.081		
			Mean std dev	0.131		

Table S7 showing peak list, signal-to-noise ratio (S/N), elemental composition, and assignment with mass errors (ppm) of the $[M+Na]^+$ dBET1 compound by CID MS/MS, calibration points are marked by an asterisk (✱).

Measured *m/z*	S/N	Elemental Composition	Theoretical *m/z*	Assignment Error (ppm)	Assignment	
287.066084	8.8	$C_{14}H_{11}N_2O_5$	287.066248	0.571	[C8'+H]	
309.047981	36.4	$C_{14}H_{10}N_2O_5Na$	309.048192	0.681	C8'	
337.043107	16.5	$C_{15}H_{10}N_2O_6Na$	337.043107	0.000	[C7'-H]	✱
341.062136	19.6	$C_{17}H_{14}ClN_4S$	341.062222	0.252	[C3+H]	
343.058988	10.6	$C_{16}H_{12}ClN_4O_3$	343.059244	0.746	[C10C13C15C21-H_6]	
343.100585	6.5	$C_{17}H_{21}ClN_2SNa$	343.100618	0.096	[C17C41+H_4]	
354.069610	19.4	$C_{15}H_{13}N_3O_6Na$	354.069656	0.131	[C6'+H]	
355.077833	21.9	$C_{18}H_{16}ClN_4S$	355.077872	0.110	[C2+H]	
362.080031	71.8	$C_{19}H_{13}ClN_5O$	362.080314	0.781	[C1C34-H_4]	
364.051848	29.8	$C_{17}H_{14}ClN_4SNa$	364.051991	0.393	[C3+H]	
366.048987	8.7	$C_{16}H_{12}ClN_4O_3Na$	366.049014	0.075	[C10C13C15C21-H_6]	
377.059739	330.6	$C_{18}H_{15}ClN_4SNa$	377.059816	0.205	[C2-H_2]	
379.075358	118.9	$C_{18}H_{17}ClN_4SNa$	379.075466	0.285	C2	
380.085290	23.9	$C_{17}H_{15}N_3O_6Na$	380.085306	0.043	C40	
381.072439	34.6	$C_{17}H_{15}ClN_4O_3Na$	381.072489	0.131	C10C15C18'	
383.072668	60.9	$C_{19}H_{16}ClN_4OS$	383.072786	0.309	[C1+H]	
385.069804	19.0	$C_{19}H_{13}ClN_5ONa$	385.070083	0.725	[C1C34-H_4]	
386.134864	7.0	$C_{19}H_{20}N_3O_6$	386.134662	-0.522	C4'	
394.100931	94.6	$C_{18}H_{17}N_3O_6Na$	394.100956	0.064	C41'	
405.054552	26.2	$C_{19}H_{15}ClN_4OSNa$	405.054731	0.443	C1	
406.100928	33.8	$C_{19}H_{17}N_3O_6Na$	406.100956	0.069	[C4'-H_2]	
408.116381	16.8	$C_{19}H_{19}N_3O_6Na$	408.116606	0.552	[C4'-H]	
410.106431	15.0	$C_{20}H_{22}ClN_3OSNa$	410.106432	0.001	C7C17	
421.111907	9.2	$C_{19}H_{18}N_4O_6Na$	421.111855	-0.123	[C1'-H_3]	
425.143155	1902.7	$C_{19}H_{22}N_4O_6Na$	425.143155	0.001	[C1'+H]	✱
448.096867	14.3	$C_{21}H_{20}ClN_5OSNa$	448.096930	0.140	C40'	
451.122499	8172.6	$C_{20}H_{20}N_4O_7Na$	451.122420	-0.175	C2'	
453.138094	755.1	$C_{20}H_{22}N_4O_7Na$	453.138070	-0.053	[C2'+H_2]	
462.112679	32.7	$C_{22}H_{22}ClN_5OSNa$	462.112580	-0.214	C5	
480.149169	7.7	$C_{24}H_{25}ClN_6OS$	480.149360	0.398	[C8C11-H_2]	
493.154780	80.7	$C_{23}H_{27}ClN_6OSNa$	493.154779	-0.001	[C7+H_2]	✱
503.139314	18.8	$C_{24}H_{25}ClN_6OSNa$	503.139129	-0.367	[C8C11-H_2]	
506.175872	38.1	$C_{22}H_{25}N_7O_6Na$	506.175852	-0.040	C18C20C83	
511.167766	32.3	$C_{25}H_{28}ClN_6O_2S$	511.167749	-0.033	[C9+H]	
530.176226	22.4	$C_{24}H_{25}N_7O_6Na$	530.175852	-0.705	C11C50C51	
533.149691	10.0	$C_{25}H_{27}ClN_6O_2SNa$	533.149694	0.006	[C9-H_2]	
540.160202	21.2	$C_{25}H_{23}N_7O_6Na$	540.160202	0.001	[C11C50-H_4]	
558.170879	467.6	$C_{33}H_{29}NO_4SNa$	558.170950	0.128	[C50+H_2]	
657.154507	513.3	$C_{32}H_{31}ClN_4O_6SNa$	657.154504	-0.004	[C17C83+H_2]	
670.205497	41.3	$C_{31}H_{33}N_7O_7SNa$	670.205438	-0.087	C25	
697.149295	157.8	$C_{34}H_{31}ClN_4O_7SNa$	697.149419	0.177	[C17-H_3CN]	
724.160318	3961.2	$C_{35}H_{32}ClN_5O_7SNa$	724.160318	0.000	[C17-H_2]	✱
725.163632	1308.1	$C_{36}H_{27}ClN_8O_6Na$	725.163429	-0.280	[C29-H_2O]	
773.220844	12.3	$C_{38}H_{35}ClN_8O_7Na$	773.220944	0.130	[C34-H_2]	
779.201923	21.7	$C_{38}H_{37}ClN_6O_7SNa$	779.202517	0.763	C37	
807.208539	12785.9	$C_{38}H_{37}ClN_8O_7SNa$	807.208665	0.156	[M+Na]	
			Abs mean error	0.245		
			Mean std dev	0.248		

Table S8 showing peak list, signal-to-noise ratio (S/N), elemental composition, and assignment with mass errors (ppm) of the $[M+Na]^+$ dBET1 compound by IRMPD MS/MS, calibration points are marked by an asterisk (✱).

Measured *m/z*	S/N	Elemental Composition	Theoretical *m/z*	Assignment Error (ppm)	Assignment	
151.083971	412.7	$C_6H_{12}N_2NaO$	151.084184	1.410	C7C3'	
176.034059	15.7	$C_9H_6NO_3$	176.034220	0.915	C90C97'	
204.124232	66.2	$C_{10}H_{14}N_5$	204.124372	0.688	C6C11C8'C20'	
218.074446	15.2	$C_{11}H_{12}N_3S$	218.074645	0.910	[C2C25+H]	
226.106205	31.3	$C_{10}H_{13}N_5Na$	226.106316	0.490	[C6C11C8'C20'-H]	
246.069436	46.5	$C_{12}H_{12}N_3OS$	246.069560	0.505	C15C21C41	
284.111605	9.3	$C_{12}H_{15}N_5O_2Na$	284.111795	0.668	[C8C12C21C45-H]	
287.066083	13.4	$C_{14}H_{11}N_2O_5$	287.066248	0.574	[C8'+H]	
297.048256	10.1	$C_{13}H_{10}N_2O_5Na$	297.048192	-0.216	[C9'+H_2]	
309.048149	24.5	$C_{14}H_{10}N_2O_5Na$	309.048192	0.139	C8'	
337.043107	27.9	$C_{15}H_{10}N_2O_6Na$	337.043107	0.001	[C7'-H]	✱
341.062158	42.3	$C_{17}H_{14}ClN_4S$	341.062222	0.187	[C3+H]	
343.059245	17.3	$C_{16}H_{12}ClN_4O_3$	343.059244	-0.003	[C10C13C15C21-H_6]	
354.069636	61.9	$C_{15}H_{13}N_3O_6Na$	354.069656	0.056	[C6'+H]	
355.077953	12.8	$C_{18}H_{16}ClN_4S$	355.077872	-0.228	[C2+H]	
361.113894	42.9	$C_{19}H_{22}ClN_2OS$	361.113589	-0.843	[C1C37+H_6]	
362.080054	38.2	$C_{19}H_{13}ClN_5O$	362.080314	0.718	[C1C34-H_4]	
377.059978	430.3	$C_{18}H_{15}ClN_4SNa$	377.059816	-0.430	[C2-H_2]	
379.075523	58.1	$C_{18}H_{17}ClN_4SNa$	379.075466	-0.150	C2	
381.072376	16.4	$C_{17}H_{15}ClN_4O_3Na$	381.072489	0.297	C10C15C18'	
383.072776	27.2	$C_{19}H_{16}ClN_4OS$	383.072786	0.026	[C1+H]	
385.069902	7.9	$C_{19}H_{13}ClN_5ONa$	385.070083	0.471	[C1C34-H_4]	
403.161159	21.3	$C_{19}H_{23}N_4O_6$	403.161211	0.129	C1'	
405.054804	16.1	$C_{19}H_{15}ClN_4OSNa$	405.054731	-0.180	C1	
406.101000	11.1	$C_{19}H_{17}N_3O_6Na$	406.100956	-0.108	[C4'-H_2]	
408.116641	16.6	$C_{19}H_{19}N_3O_6Na$	408.116606	-0.085	[C4'-H]	
410.106866	22.5	$C_{23}H_{16}N_5OS$	410.107008	0.346	[C6C22-H_6]	
421.112252	6.3	$C_{19}H_{18}N_4O_6Na$	421.111855	-0.943	[C1'-H_3]	
425.143091	1357.7	$C_{19}H_{22}N_4O_6Na$	425.143155	0.151	[C1'+H]	
451.122428	9688.6	$C_{20}H_{20}N_4O_7Na$	451.122420	-0.018	C2'	✱
453.138116	1840.5	$C_{20}H_{22}N_4O_7Na$	453.138070	-0.101	[C2'+H_2]	
480.149355	20.7	$C_{24}H_{25}ClN_6OS$	480.149360	0.011	[C8C11-H_2]	
493.154769	8.2	$C_{23}H_{27}ClN_6OSNa$	493.154779	0.020	[C7+H_2]	✱
506.175788	67.0	$C_{22}H_{25}N_7O_6Na$	506.175852	0.126	C18C20C83	
511.167634	7.3	$C_{25}H_{28}ClN_6O_2S$	511.167749	0.225	[C9+H]	
533.149413	7.8	$C_{25}H_{27}ClN_6O_2SNa$	533.149694	0.526	[C9-H_2]	
540.160457	8.0	$C_{25}H_{23}N_7O_6Na$	540.160202	-0.473	[C11C50-H_4]	
558.170501	42.8	$C_{33}H_{29}NO_4SNa$	558.170950	0.805	[C50+H_2]	
657.154157	52.5	$C_{32}H_{31}ClN_4O_6SNa$	657.154504	0.527	[C17C83+H_2]	
697.149278	242.6	$C_{34}H_{31}ClN_4O_7SNa$	697.149419	0.202	[C17-H_3CN]	
724.160321	2911.8	$C_{35}H_{32}ClN_5O_7SNa$	724.160318	-0.004	[C17-H_2]	✱
725.163695	944.5	$C_{36}H_{27}ClN_8O_6Na$	725.163429	-0.367	[C29-H_2O]	
773.221275	5.5	$C_{38}H_{35}ClN_8O_7Na$	773.220944	-0.428	[C34-H_2]	
807.208389	11443.3	$C_{38}H_{37}ClN_8O_7SNa$	807.208665	0.342	[M+Na]	
			Abs mean error	0.365		
			Mean std dev	0.328		

Table S9 showing peak list, signal-to-noise ratio (S/N), elemental composition, and assignment with mass errors (ppm) of the $[M+Na]^+$ dBET1 compound by UVPD MS/MS, calibration points are marked by an asterisk (✱).

Measured *m/z*	S/N	Elemental Composition	Theoretical *m/z*	Assignment Error (ppm)	Assignment	
122.057644	17.3	C_5H_9NONa	122.057635	-0.072	C6C2'	
149.068527	20.9	$C_6H_{10}N_2ONa$	149.068534	0.044	C8C11C2'	
151.084185	141.0	$C_6H_{12}N_2ONa$	151.084184	-0.005	C7C3'	✱
166.071270	20.9	$C_6H_{11}N_2O_2Na$	166.071273	0.015	[C8C2'+H]	
180.086921	40.0	$C_7H_{13}N_2O_2Na$	180.086923	0.011	[C9C2'+H$_2$]	✱
198.016154	20.3	$C_9H_5NO_3Na$	198.016164	0.053	C96C6'	
202.106315	36.4	$C_8H_{13}N_5Na$	202.106316	0.006		
204.124373	39.4	$C_{10}H_{14}N_5$	204.124372	-0.005	C6C11C8'C20'	
226.106299	38.8	$C_{10}H_{13}N_5Na$	226.106316	0.077	[C6C11C8'C20'-H]	
240.056580	97.0	$C_{11}H_{11}N_3SNa$	240.056589	0.039	C2C25	
262.129833	15.7	$C_{12}H_{16}N_5O_2$	262.129851	0.071	C8C12C21C45	
294.061089	37.2	$C_{14}H_{11}N_2O_4Na$	294.061103	0.048	[C99C4'-H$_2$]	
296.040367	56.0	$C_{13}H_9N_2O_5Na$	296.040367	-0.001	[C9'+H]	
297.048234	26.0	$C_{13}H_{10}N_2O_5Na$	297.048192	-0.141	[C9'+H$_2$]	
304.092139	45.8	$C_{17}H_{19}ClNS$	304.092125	-0.045	[C1C11C51+H$_4$]	
309.048192	257.3	$C_{14}H_{10}N_2O_5Na$	309.048192	0.000	C8'	
314.111124	29.5	$C_{14}H_{17}N_3O_4Na$	314.111127	0.009	[C99C1'+H$_2$]	
337.043110	120.4	$C_{15}H_{10}N_2O_6Na$	337.043107	-0.009	[C7'-H]	✱
340.090395	102.2	$C_{15}H_{15}N_3O_5Na$	340.090391	-0.012	[C49C6'+H$_3$]	
343.100609	38.6	$C_{17}H_{21}ClN_2SNa$	343.100618	0.026	[C17C41+H$_4$]	
351.044125	28.3	$C_{16}H_{13}ClN_4SNa$	351.044166	0.116	C24	
354.069668	143.2	$C_{15}H_{13}N_3O_6Na$	354.069656	-0.033	[C6'+H]	
355.077884	24.2	$C_{18}H_{16}ClN_4S$	355.077872	-0.034	[C2+H]	
361.113599	51.0	$C_{19}H_{22}ClN_2OS$	361.113589	-0.028	[C1C37+H$_6$]	
364.052000	52.9	$C_{17}H_{14}ClN_4SNa$	364.051991	-0.026	[C3+H]	
366.049130	14.6	$C_{16}H_{12}ClN_4O_3Na$	366.049014	-0.316	[C10C13C15C21-H$_6$]	
366.106027	14.2	$C_{17}H_{17}N_3O_5Na$	366.106041	0.039	[C78C40'+H]	
377.059826	441.4	$C_{18}H_{15}ClN_4SNa$	377.059816	-0.026	[C2-H$_2$]	
379.075474	75.7	$C_{18}H_{17}ClN_4SNa$	379.075466	-0.021	C2	
380.085310	67.8	$C_{17}H_{15}N_3O_6Na$	380.085306	-0.010	C40	
380.121709	90.2	$C_{18}H_{19}N_3O_5Na$	380.121691	-0.047	[C90C1'-H$_2$]	
381.072528	22.4	$C_{17}H_{15}ClN_4O_3Na$	381.072489	-0.101	C10C15C18'	
394.100955	166.5	$C_{18}H_{17}N_3O_6Na$	394.100956	0.002	C41'	
405.054761	61.0	$C_{19}H_{15}ClN_4OSNa$	405.054731	-0.073	C1	
406.100931	54.8	$C_{19}H_{17}N_3O_6Na$	406.100956	0.061	[C4'-H$_2$]	
407.132623	13.8	$C_{19}H_{20}N_4O_5Na$	407.132590	-0.082	[C1'-OH]	
408.116606	62.6	$C_{19}H_{19}N_3O_6Na$	408.116606	0.000	[C4'-H]	
410.106426	21.8	$C_{20}H_{22}ClN_3OSNa$	410.106432	0.016	C7C17	
421.112077	13.9	$C_{19}H_{18}N_4O_6Na$	421.111855	-0.527	[C1'-H$_3$]	
423.127479	80.4	$C_{19}H_{20}N_4O_6Na$	423.127505	0.061	[C1'-H]	
425.143156	1062.7	$C_{19}H_{22}N_4O_6Na$	425.143155	-0.004	[C1'+H]	✱
435.089010	27.5	$C_{20}H_{19}ClN_5OSNa$	435.089105	0.218	C41	
448.096943	101.1	$C_{21}H_{20}ClN_5OSNa$	448.096930	-0.029	C40'	
451.122425	1781.3	$C_{20}H_{20}N_4O_7Na$	451.122420	-0.012	C2'	
453.138081	106.6	$C_{20}H_{22}N_4O_7Na$	453.138070	-0.025	[C2'+H$_2$]	
462.112584	156.3	$C_{22}H_{22}ClN_5OSNa$	462.112580	-0.009	C5	✱
491.139284	12.8	$C_{23}H_{25}ClN_6OSNa$	491.139129	-0.316	C7	
493.154909	41.5	$C_{23}H_{27}ClN_6OSNa$	493.154779	-0.264	[C7+H$_2$]	
519.134152	23.3	$C_{24}H_{25}ClN_6O_2SNa$	519.134044	-0.207	C8	
657.154312	16.1	$C_{32}H_{31}ClN_4O_6SNa$	657.154504	0.293	[C17C83+H$_2$]	
724.160302	67.0	$C_{35}H_{32}ClN_5O_7SNa$	724.160318	0.022	[C17-H$_2$]	✱

725.163451	24.4	$C_{36}H_{27}ClN_8O_6Na$	725.163429	-0.030	[C29-O_2H][C29-H_4]
772.239547	22.7	$C_{38}H_{37}N_8O_7SNa$	772.239812	0.343	C22
806.200405	13.9	$C_{38}H_{36}ClN_8O_7SNa$	806.200840	0.540	[M-H]
807.207949	10035.1	$C_{38}H_{37}ClN_8O_7SNa$	807.208665	0.887	[M+Na]
			Abs mean error	0.078	
			Mean std dev	0.111	

Table S10 showing peak list, signal-to-noise ratio (S/N), elemental composition, and assignment with mass errors (ppm) of the $[M+Ag]^+$ dBET1 compound by CID MS/MS, calibration points are marked by an asterisk (✱).

Measured *m/z*	S/N	Elemental Composition	Theoretical *m/z*	Assignment Error (ppm)	Assignment	
341.062223	15.4	$C_{17}H_{14}ClN_4S$	341.062222	-0.001	[C3+H]	✱
349.085016	18.5	$C_{19}H_{14}ClN_4O$	349.085065	0.140	C1C34	
355.077841	674.2	$C_{18}H_{16}ClN_4S$	355.077872	0.086	[C2+H]	
358.082602	6.9	$C_{17}H_{15}ClN_4O_3$	358.082720	0.328	C10C15C18'	
379.942339	14.1	$C_{15}H_{12}ClNSAg$	379.942441	0.267	C14C20	
381.057124	236.5	$C_{19}H_{14}ClN_4OS$	381.057136	0.032	[C1-H]	
382.064913	18.4	$C_{19}H_{15}ClN_4OS$	382.064961	0.125	C1	
386.134648	179.0	$C_{19}H_{20}N_3O_6$	386.134662	0.037	C4'	
392.963522	23.9	$C_{14}H_{10}N_2O_5Ag$	392.963514	-0.021	C8'	
399.129896	219.1	$C_{19}H_{19}N_4O_6$	399.129911	0.038		
403.161209	308.6	$C_{19}H_{23}N_4O_6$	403.161211	0.006	C1'	✱
412.099531	9.5	$C_{20}H_{19}ClN_5OS$	412.099336	-0.472	C41	
422.948208	11.6	$C_{16}H_{13}ClN_2OSAg$	422.948255	0.111	C4C17	
437.984986	31.6	$C_{15}H_{13}N_3O_6Ag$	437.984978	-0.018	[C6'+H]	
446.959481	11.0	$C_{17}H_{13}ClN_4SAg$	446.959488	0.015	C3	
447.967335	146.0	$C_{17}H_{14}ClN_4SAg$	447.967313	-0.049	[C3+H]	
449.964303	42.3	$C_{16}H_{12}ClN_4O_3Ag$	449.964336	0.073	[C10C13C15C21-H_6]	
454.982215	9.2	$C_{19}H_{13}ClN_4OAg$	454.982332	0.257	[C1C34-H_2]	
460.975186	487.6	$C_{18}H_{15}ClN_4SAg$	460.975138	-0.103	[C2-H_2]	
462.990810	926.2	$C_{18}H_{17}ClN_4SAg$	462.990788	-0.046	C2	
464.987834	298.2	$C_{17}H_{15}ClN_4O_3Ag$	464.987811	-0.049	C10C15C18'	
478.985891	6.7	$C_{18}H_{17}ClN_4OSAg$	478.985703	-0.392	[C1C13+H_2]	
488.970084	58.0	$C_{19}H_{15}ClN_4OSAg$	488.970053	-0.064	C1	
490.016322	114.0	$C_{19}H_{17}N_3O_6Ag$	490.016278	-0.090	[C4'-H_2]	
492.031942	122.8	$C_{19}H_{19}AgN_3O_6$	492.031928	-0.029		
494.021681	6.0	$C_{20}H_{22}ClN_3OSAg$	494.021754	0.149	C7C17	
499.073071	42.2	$C_{21}H_{29}AgClN_2OS$	499.073455	0.769		
501.072741	20.1	$C_{26}H_{22}AgN_2O_2$	501.072671	-0.139		
504.006116	29.5	$C_{21}H_{20}ClN_3OSAg$	504.006104	-0.024	[C6C15-H_2]	
505.027236	93.0	$C_{19}H_{18}N_4O_6Ag$	505.027177	-0.118	[C1'-H_3]	
507.042839	419.6	$C_{19}H_{20}N_4O_6Ag$	507.042827	-0.024	[C1'-H]	
509.058488	1466.7	$C_{19}H_{22}AgN_4O_6$	509.058477	-0.021	C1'	✱
519.042771	31.8	$C_{20}H_{20}N_4O_6Ag$	519.042827	0.108	[C2'-OH]	
535.037733	327.4	$C_{20}H_{20}N_4O_7Ag$	535.037742	0.016	C2'	✱
537.053382	132.8	$C_{20}H_{22}N_4O_7Ag$	537.053392	0.020	[C2'+H_2]	
546.027874	15.3	$C_{22}H_{22}ClN_5OSAg$	546.027902	0.051	C5	
560.043360	5.9	$C_{23}H_{24}ClN_5OSAg$	560.043552	0.343	C6	
575.054468	13.7	$C_{23}H_{25}ClN_6OSAg$	575.054451	-0.030	C7	
577.070252	30.5	$C_{23}H_{27}ClN_6OSAg$	577.070101	-0.262	[C7+H_2]	
587.054583	59.0	$C_{24}H_{25}ClN_6OSAg$	587.054451	-0.225	[C8C11-H_2]	
590.091270	67.5	$C_{22}H_{25}N_7O_6Ag$	590.091175	-0.160	C18C20C83	
603.075565	17.7	$C_{31}H_{28}AgO_4S$	603.075374	-0.316		
624.075662	6.5	$C_{33}H_{27}AgNO_3S$	624.075708	0.074		
642.086256	48.6	$C_{33}H_{29}NO_4SAg$	642.086273	0.026	[C50+H_2]	
689.091181	21.5	$C_{37}H_{18}ClN_8O_3S$	689.090562	-0.898		
702.177710	6.7	$C_{35}H_{33}ClN_5O_7S$	702.178374	0.945	C17	
741.069827	149.7	$C_{32}H_{31}ClN_4O_6SAg$	741.069827	0.000	[C17C83+H_2]	✱
751.238996	9.3	$C_{38}H_{36}ClN_8O_7$	751.239000	0.006	[C34-H]	
754.120287	19.6	$C_{31}H_{33}N_7O_7SAg$	754.120761	0.628	C25	
781.064367	17.2	$C_{34}H_{31}ClN_4O_7SAg$	781.064741	0.478	[C17-H_3CN]	
783.210959	398.3	$C_{38}H_{36}ClN_8O_7S$	783.211071	0.142	[M-H]	
808.075386	760.0	$C_{35}H_{32}ClN_5O_7SAg$	808.075640	0.314	[C17-H_2]	

809.078772	272.9	$C_{36}H_{27}ClN_8O_6Ag$	809.078751	-0.025	[C29-H_2O]
835.086526	5.2	$C_{36}H_{33}ClN_6O_7SAg$	835.086539	0.016	C15
857.136097	27.1	$C_{38}H_{35}ClN_8O_7Ag$	857.136266	0.198	[C34-H_2]
863.129104	11.7	$C_{37}H_{37}ClN_8O_6SAg$	863.129073	-0.036	[M-CO_2]
873.113638	18.3	$C_{38}H_{35}ClN_8O_6SAg$	873.113423	-0.246	[M-OH_2]
874.097544	14.9	$C_{38}H_{34}ClN_7O_7SAg$	874.097438	-0.121	[M-NH_3]
889.108216	245.0	$C_{38}H_{35}ClN_8O_7SAg$	889.108337	0.137	[M-H_2]
891.123896	3684.3	$C_{38}H_{37}ClN_8O_7SAg$	891.123987	0.103	[M+Ag]
			Abs mean error	0.146	
			Mean std dev	0.188	

Table S11 showing peak list, signal-to-noise ratio (S/N), elemental composition, and assignment with mass errors (ppm) of the $[M+Ag]^+$ dBET1 compound by IRMPD MS/MS, calibration points are marked by an asterisk (✱).

Measured *m/z*	S/N	Elemental Composition	Theoretical *m/z*	Assignment Error (ppm)	Assignment	
106.904600	47.6	Ag	106.904543	-0.534		
125.070989	18.0	$C_6H_9N_2O$	125.070939	-0.402	C8C2'	
176.034219	320.9	$C_9H_6NO_3$	176.034220	0.004	C90C97'	✱
185.946746	32.7	C_5H_5NAg	185.946742	-0.023	C2C37C50	
188.957664	94.2	$C_4H_6N_2Ag$	188.957641	-0.120	[C3C37C47'+H_2]	
202.973291	252.1	$C_5H_8N_2Ag$	202.973291	-0.000	[C2C37C47'+H_2]	
230.968196	106.3	$C_6H_8N_2OAg$	230.968206	0.043	[C1C37C47'+H_2]	
232.983876	7.5	$C_6H_{10}N_2OAg$	232.983856	-0.086	C8C11C2'	
238.983198	21.6	$C_6H_{12}AgO_3$	238.983187	-0.046		
241.068294	41.9	$C_{15}H_{13}OS$	241.068163	-0.544		
274.045111	15.2	$C_{15}H_{13}ClNS$	274.045175	0.234	C2C17	
275.102615	25.8	$C_{14}H_{15}N_2O_4$	275.102633	0.067		
287.066242	1265.1	$C_{14}H_{11}N_2O_5$	287.066248	0.022	[C8'+H]	✱
300.024408	22.1	$C_{16}H_{11}ClNOS$	300.024439	0.103	[C1C17-H]	
315.061166	128.3	$C_{15}H_{11}N_2O_6$	315.061163	-0.010	C7'	✱
349.085078	12.9	$C_{19}H_{14}ClN_4O$	349.085065	-0.038	C1C34	
350.982731	18.1	$C_{12}H_{12}N_4SAg$	350.982811	0.229	C2C21	
355.077876	891.6	$C_{18}H_{16}ClN_4S$	355.077872	-0.011	[C2+H]	✱
356.085714	34.0	$C_{18}H_{17}ClN_4S$	356.085697	-0.048	C2	
379.942456	118.5	$C_{15}H_{12}ClNSAg$	379.942441	-0.038	C14C20	
380.963520	21.6	$C_{13}H_{10}N_2O_5Ag$	380.963514	-0.015	[C9'+H_2]	
381.057156	207.6	$C_{19}H_{14}ClN_4OS$	381.057136	-0.052	[C1-H]	
382.064939	17.9	$C_{19}H_{15}ClN_4OS$	382.064961	0.058	C1	
386.134686	344.1	$C_{19}H_{20}N_3O_6$	386.134662	-0.062	C4'	
392.963528	68.4	$C_{14}H_{10}N_2O_5Ag$	392.963514	-0.036	C8'	
399.129915	651.9	$C_{19}H_{19}N_4O_6$	399.129911	-0.011	[C1'-H_2]	
403.161212	1699.6	$C_{19}H_{23}N_4O_6$	403.161211	-0.001	C1'	✱
437.985038	32.5	$C_{15}H_{13}N_3O_6Ag$	437.984978	-0.138	[C6'+H]	
447.967263	8.6	$C_{17}H_{14}ClN_4SAg$	447.967313	0.112	[C3+H]	
454.982504	8.5	$C_{19}H_{13}ClN_4OAg$	454.982332	-0.378	[C1C34-H_2]	
460.005857	27.1	$C_{18}H_{15}N_3O_5Ag$	460.005714	-0.310	[C41'-H_2O]	
460.975157	1334.8	$C_{18}H_{15}ClN_4SAg$	460.975138	-0.040	[C2-H_2]	
462.990807	1051.1	$C_{18}H_{17}ClN_4SAg$	462.990788	-0.041	C2	
464.987910	341.1	$C_{17}H_{15}ClN_4O_3Ag$	464.987811	-0.214	C10C15C18'	
477.032282	12.8	$C_{18}H_{18}N_4O_5Ag$	477.032263	-0.039	C15C21C96C18'	
488.000617	11.5	$C_{19}H_{15}N_3O_6Ag$	488.000628	0.022	[C4'-H_4]	
488.970112	38.1	$C_{19}H_{15}ClN_4OSAg$	488.970053	-0.121	C1	
489.032275	33.6	$C_{19}H_{18}N_4O_5Ag$	489.032263	-0.024	[C1'-H_2O]	
490.016314	205.1	$C_{19}H_{17}N_3O_6Ag$	490.016278	-0.074	[C4'-H_2]	
492.031952	163.0	$C_{19}H_{19}AgN_3O_6$	492.031928	-0.049		
494.021717	23.0	$C_{20}H_{22}ClN_3OSAg$	494.021754	0.076	C7C17	
504.006127	116.6	$C_{21}H_{20}ClN_3OSAg$	504.006104	-0.045	[C6C15-H_2]	
505.027197	367.8	$C_{19}H_{18}N_4O_6Ag$	505.027177	-0.039	[C1'-H_3]	
507.042836	306.6	$C_{19}H_{20}N_4O_6Ag$	507.042827	-0.019	[C1'-H]	
509.058484	1481.2	$C_{19}H_{22}N_4O_6Ag$	509.058477	-0.014	[C1'+H]	✱
519.042930	36.0	$C_{20}H_{20}N_4O_6Ag$	519.042827	-0.198	[C2'-OH]	
535.037777	135.5	$C_{20}H_{20}N_4O_7Ag$	535.037742	-0.065	C2'	
537.053405	183.3	$C_{20}H_{22}N_4O_7Ag$	537.053392	-0.023	[C2'+H_2]	
575.054689	12.3	$C_{23}H_{25}ClN_6OSAg$	575.054451	-0.413	C7	
577.070269	21.9	$C_{23}H_{27}ClN_6OSAg$	577.070101	-0.292	[C7+H_2]	
587.054444	19.7	$C_{24}H_{25}ClN_6OSAg$	587.054451	0.012	[C8C11-H_2]	
590.091176	67.4	$C_{22}H_{25}N_7O_6Ag$	590.091175	-0.001	C18C20C83	✱
741.069811	49.7	$C_{32}H_{31}ClN_4O_6SAg$	741.069827	0.022	[C17C83+H_2]	✱

781.064364	7.1	$C_{34}H_{31}ClN_4O_7SAg$	781.064741	0.483	[C17-H_3CN]
783.211099	422.4	$C_{38}H_{36}ClN_8O_7S$	783.211071	-0.035	[M-H]
808.075634	714.5	$C_{35}H_{32}ClN_5O_7SAg$	808.075640	0.007	[C17-H_2]
809.078975	255.0	$C_{36}H_{27}ClN_8O_6Ag$	809.078751	-0.277	[C29-H_2O]
857.135642	10.9	$C_{38}H_{35}ClN_8O_7Ag$	857.136266	0.729	[C34-H_2]
874.097682	66.0	$C_{38}H_{34}ClN_7O_7SAg$	874.097438	-0.279	[M-NH_3]
889.108408	391.6	$C_{38}H_{35}ClN_8O_7SAg$	889.108337	-0.080	[M-H_2]
891.123637	5688.0	$C_{38}H_{37}ClN_8O_7SAg$	891.123987	0.392	[M+Ag]
			Abs mean error	0.113	
			Mean std dev	0.150	

Table S12 showing peak list, signal-to-noise ratio (S/N), elemental composition, and assignment with mass errors (ppm) of the $[M+Ag]^+$ dBET1 compound by UVPD MS/MS, calibration points are marked by an asterisk (✱).

Measured *m/z*	S/N	Elemental Composition	Theoretical *m/z*	Assignment Error (ppm)	Assignment	
106.904521	124.6	Ag	106.904543	0.207		
125.070944	8.4	$C_6H_9N_2O$	125.070939	-0.037	C8C2'	
147.931089	18.1	C_2H_3NAg	147.931092	0.021	C14C17'	
150.037201	23.9	C_8H_8NS	150.037197	-0.024	[C3C17C21+H]	
162.037208	10.2	C_9H_8NS	162.037197	-0.066	[C2C17C21-H]	
175.962397	20.6	C_4H_7NAg	175.962392	-0.029	[C6C1'-H_2]	
176.034219	60.4	$C_9H_6NO_3$	176.034220	0.007	C90C97'	✱
185.946752	41.2	C_5H_5NAg	185.946742	-0.055	C2C37C50	
188.957722	12.0	$C_4H_6N_2Ag$	188.957641	-0.429	[C3C37C47'+H_2]	
189.952897	240.4	$C_3H_5N_3Ag$	189.952890	-0.035	[C17'+H_2]	
195.004597	68.7	$C_4H_{12}N_2Ag$	195.004591	-0.032	[C7C1'+H_2]	
202.049880	27.6	$C_{11}H_8NO_3$	202.049870	-0.050	C93C8'	
202.973307	140.8	$C_5H_8N_2Ag$	202.973291	-0.077	[C2C37C47'+H_2]	
204.988958	22.5	$C_5H_{10}N_2Ag$	204.988941	-0.083	C7C11C2'	
214.948152	28.3	$C_4H_4N_4Ag$	214.948139	-0.061	C3C18'C20'	
217.066828	13.0	$C_{11}H_{11}N_3S$	217.066820	-0.037	C2C25	
218.074655	125.5	$C_{11}H_{12}N_3S$	218.074645	-0.047	[C2C25+H]	
220.090319	8.7	$C_{11}H_{14}N_3S$	220.090295	-0.111	[C2C25+H_3]	
220.983867	13.3	$C_5H_{10}N_2OAg$	220.983856	-0.049	C7C2'	
222.999583	14.3	$C_5H_{12}N_2OAg$	222.999506	-0.346	[C7C2'+H_2]	
223.032439	9.1	$C_{13}H_7N_2S$	223.032446	0.032	[C3C13C13C15C22+H]	
230.968223	41.4	$C_6H_8N_2OAg$	230.968206	-0.072	[C1C37C47'+H_2]	
232.983902	16.4	$C_6H_{10}N_2OAg$	232.983856	-0.199	C8C11C2'	
233.018667	23.5	$C_{13}H_{10}ClS$	233.018626	-0.176	[C47+H_2]	
234.999535	31.2	$C_6H_{12}N_2OAg$	234.999506	-0.123	C7C3'	
238.055947	79.1	$C_{14}H_{10}N_2S$	238.055921	-0.107	C13C14C22C24	

241.068435	17.6	$C_4H_{12}ClN_7O_3$	241.068466	0.127		
245.085565	29.7	$C_{12}H_{13}N_4S$	245.085544	-0.084	[C2C21+H]	
246.069579	53.5	$C_{12}H_{12}N_3OS$	246.069560	-0.076	C15C21C41	
247.021672	7.8	$C_{13}H_{10}ClNS$	247.021700	0.113	C17C24	
271.064804	7.7	$C_{13}H_{11}N_4OS$	271.064809	0.017	C1C21	
272.029541	50.0	$C_{15}H_{11}ClNS$	272.029525	-0.057	[C2C17-H_2]	
273.037326	9.0	$C_{15}H_{12}ClNS$	273.037350	0.088	C14C20	
274.045196	299.1	$C_{15}H_{13}ClNS$	274.045175	-0.076	C2C17	
281.931545	10.5	$C_9H_5NO_3Ag$	281.931486	-0.208	C96C6'	
286.045196	75.0	$C_{16}H_{13}ClNS$	286.045175	-0.073	C1C17C11	
287.066265	372.2	$C_{14}H_{11}N_2O_5$	287.066248	-0.060	[C8'+H]	
295.947094	14.3	$C_{10}H_7NO_3Ag$	295.947136	0.141	C94C8'	
300.024474	17.1	$C_{16}H_{11}ClNOS$	300.024439	-0.118	[C1C17-H]	
300.060871	39.5	$C_{17}H_{15}ClNS$	300.060825	-0.154		
306.093371	27.7	$C_{17}H_{14}N_4S$	306.093369	-0.006		
311.040536	14.3	$C_{17}H_{12}ClN_2S$	311.040424	-0.362	[C3C37-H]	
312.048269	13.2	$C_{17}H_{13}ClN_2S$	312.048249	-0.064	C3C37	
313.056070	45.8	$C_{17}H_{14}ClN_2S$	313.056074	0.011		
314.051330	38.1	$C_{16}H_{13}ClN_3S$	314.051323	-0.021		
315.061174	186.4	$C_{15}H_{11}N_2O_6$	315.061163	-0.037	C7'	*
319.101163	10.9	$C_{18}H_{15}N_4S$	319.101194	0.098	C2C22	
321.090114	18.4	$C_{18}H_{14}ClN_4$	321.090151	0.114		
323.105803	69.6	$C_{18}H_{16}ClN_4$	323.105801	-0.006		
323.971933	76.3	$C_{11}H_{11}N_3SAg$	323.971912	-0.065	C2C25	
324.979693	11.7	$C_{11}H_{12}N_3SAg$	324.979737	0.136	[C2C25+H]	
325.971565	74.5	$C_{13}H_7N_4Ag$	325.971614	0.151	C3C22C45	
325.987535	18.6	$C_{11}H_{13}N_3SAg$	325.987562	0.084	[C2C25+H_2]	
326.063881	14.0	$C_{18}H_{15}ClN_2S$	326.063899	0.055	C11C40	
327.071744	38.1	$C_{18}H_{16}ClN_2S$	327.071724	-0.062	C2C37	
329.062162	28.1	$C_{16}H_{14}ClN_4S$	329.062222	0.182	[C24+H]	

338.051482	8.8	$C_{18}H_{13}ClN_3S$	338.051323	-0.471	[C2C12-H_3]	
339.059185	10.3	$C_{18}H_{14}ClN_3S$	339.059148	-0.108	[C2C12-H_2]	
341.062216	211.9	$C_{17}H_{14}ClN_4S$	341.062222	0.018	[C3+H]	*
342.070029	68.2	$C_{17}H_{15}ClN_4S$	342.070047	0.054	[C3+H_2]	
343.059286	65.9	$C_{16}H_{12}ClN_4O_3$	343.059244	-0.123	[C10C13C15C21-H_6]	
349.085067	43.0	$C_{19}H_{14}ClN_4O$	349.085065	-0.005	C1C34	
350.982825	39.2	$C_{12}H_{12}N_4SAg$	350.982811	-0.039	C2C21	
353.062225	35.6	$C_{18}H_{14}ClN_4S$	353.062222	-0.009	[C2-H]	
354.069982	18.4	$C_{18}H_{15}ClN_4S$	354.070047	0.183	[C2-H_2]	
355.077863	1592.8	$C_{18}H_{16}ClN_4S$	355.077872	0.025	[C2+H]	*
356.085684	50.5	$C_{18}H_{17}ClN_4S$	356.085697	0.037	C2	
358.082728	10.5	$C_{17}H_{15}ClN_4O_3$	358.082720	-0.023	C10C15C18'	
359.850556	201.2	$C_7AgClN_5O_2S$	359.850666	0.306		
364.968617	9.6	$C_{13}H_{10}N_2O_4Ag$	364.968600	-0.046	[C99C41'-H_2]	
379.942411	185.4	$C_{15}H_{12}ClNSAg$	379.942441	0.079	C14C20	
379.955728	11.8	$C_{13}H_9N_2O_5Ag$	379.955689	-0.103	[C9'+H]	
380.963496	20.3	$C_{13}H_{10}N_2O_5Ag$	380.963514	0.049	[C9'+H_2]	
381.057105	217.5	$C_{19}H_{14}ClN_4OS$	381.057136	0.081	[C1-H]	
382.064963	47.2	$C_{19}H_{15}ClN_4OS$	382.064961	-0.007	C1	
383.072756	200.0	$C_{19}H_{16}ClN_4OS$	383.072786	0.079	[C1+H]	
384.119008	76.5	$C_{19}H_{18}N_3O_6$	384.119012	0.009	[C4'-H_2]	
386.134630	88.7	$C_{19}H_{20}N_3O_6$	386.134662	0.082	C4'	
392.963475	137.3	$C_{14}H_{10}N_2O_5Ag$	392.963514	0.099	C8'	
398.026383	9.5	$C_{14}H_{17}N_3O_4Ag$	398.026449	0.167	[C99C1'+H_2]	
399.129869	632.2	$C_{19}H_{19}N_4O_6$	399.129911	0.106	[C1'-H_2]	
400.099301	49.2	$C_{19}H_{19}ClN_5OS$	400.099336	0.088	[C4+H_2]	
403.161113	14.7	$C_{19}H_{23}N_4O_6$	403.161211	0.242	C1'	
412.099279	35.3	$C_{20}H_{19}ClN_5OS$	412.099336	0.140	C41	
420.958468	8.1	$C_{15}H_{10}N_2O_6Ag$	420.958429	-0.092	[C7'-H]	
427.015920	21.5	$C_{17}H_{21}ClN_2SAg$	427.015941	0.049	[C17C41+H_4]	

437.984907	46.0	$C_{15}H_{13}N_3O_6Ag$	437.984978	0.163	[C6'+H]
446.959354	17.2	$C_{17}H_{13}ClN_4SAg$	446.959488	0.301	C3
447.967210	101.3	$C_{17}H_{14}ClN_4SAg$	447.967313	0.229	[C3+H]
449.964363	26.6	$C_{16}H_{12}ClN_4O_3Ag$	449.964336	-0.060	[C10C13C15C21-H_6]
449.984786	10.7	$C_{16}H_{13}N_3O_6Ag$	449.984978	0.426	C5'
460.005614	24.2	$C_{18}H_{15}N_3O_5Ag$	460.005714	0.217	[C41'-H_2O]
460.975023	352.3	$C_{18}H_{15}ClN_4SAg$	460.975138	0.251	[C2-H_2]
462.990661	284.2	$C_{18}H_{17}ClN_4SAg$	462.990788	0.274	C2
464.036775	9.6	$C_{18}H_{19}N_3O_5Ag$	464.037014	0.514	[C90C1'-H_2]
464.987690	83.5	$C_{17}H_{15}ClN_4O_3Ag$	464.987811	0.261	C10C15C18'
477.032011	10.7	$C_{18}H_{18}N_4O_5Ag$	477.032263	0.529	C15C21C96C18'
478.016220	14.3	$C_{18}H_{17}N_3O_6Ag$	478.016278	0.120	C41'
488.000997	6.0	$C_{19}H_{15}N_3O_6Ag$	488.000628	-0.757	[C4'-H_4]
488.969875	55.6	$C_{19}H_{15}ClN_4OSAg$	488.970053	0.365	C1
489.032105	33.1	$C_{19}H_{18}N_4O_5Ag$	489.032263	0.323	[C1'-H_2O]
490.016126	144.4	$C_{19}H_{17}N_3O_6Ag$	490.016278	0.310	[C4'-H_2]
492.031765	147.7	$C_{19}H_{19}N_3O_6Ag$	492.031928	0.332	[C4'-H]
504.006008	29.9	$C_{21}H_{20}ClN_3OSAg$	504.006104	0.191	[C6C15-H_2]
505.026988	191.2	$C_{19}H_{18}N_4O_6Ag$	505.027177	0.374	[C1'-H_3]
507.042604	141.2	$C_{19}H_{20}N_4O_6Ag$	507.042827	0.439	[C1'-H]
509.058261	182.3	$C_{19}H_{22}N_4O_6Ag$	509.058477	0.424	[C1'+H]
511.167779	9.8	$C_{25}H_{28}ClN_6O_2S$	511.167749	-0.058	[C9+H]
519.042987	9.0	$C_{20}H_{20}N_4O_6Ag$	519.042827	-0.308	[C2'-OH]
532.011863	22.0	$C_{21}H_{20}ClN_5OSAg$	532.012252	0.730	C40'
535.037478	48.6	$C_{20}H_{20}N_4O_7Ag$	535.037742	0.493	C2'
537.052955	12.2	$C_{20}H_{22}N_4O_7Ag$	537.053392	0.813	[C2'+H_2]
546.027677	39.4	$C_{22}H_{22}ClN_5OSAg$	546.027902	0.412	C5
573.038503	8.3	$C_{23}H_{23}ClN_6OSAg$	573.038801	0.520	[C7-H_3]
575.053967	10.1	$C_{23}H_{25}ClN_6OSAg$	575.054451	0.841	C7
577.069784	68.9	$C_{23}H_{27}ClN_6OSAg$	577.070101	0.549	[C7+H_2]

603.048888	5.8	$C_{24}H_{25}ClN_6O_2SAg$	603.049366	0.793	C8	
808.074306	27.4	$C_{33}H_{30}ClN_8O_6SAg$	808.074298	-0.010	C43C87	*
809.078519	9.8	$C_{36}H_{27}ClN_8O_6Ag$	809.078751	0.286	[C29-H_2O]	
856.154415	11.0	$C_{38}H_{37}N_8O_7SAg$	856.155135	0.842	C22	
890.115525	18.4	$C_{38}H_{36}ClN_8O_7SAg$	890.116162	0.716	[M-H]	
891.122333	8048.4	$C_{38}H_{37}ClN_8O_7SAg$	891.123987	1.856	[M+Ag]	
			Abs mean error	0.188		
			Mean std dev	0.203		

Table S13 showing peak list, signal-to-noise ratio (S/N), elemental composition, and assignment with mass errors (ppm) of the [M-H]$^-$ dBET1 compound by CID MS/MS, calibration points are marked by an asterisk (✱).

Measured *m/z*	S/N	Elemental Composition	Theoretical *m/z*	Assignment Error (ppm)	Assignment	
128.035333	11.1	$C_5H_6NO_3$	128.035317	-0.121	[C88C96C6'+H_2]	
130.029864	8.8	C_8H_4NO	130.029837	-0.208	C1C18'C23	
147.008770	10.2	$C_8H_3O_3$	147.008768	-0.016	C95C9'	
148.040394	8.5	$C_8H_6NO_2$	148.040402	0.055	C6'C98	
160.040433	8.1	$C_9H_6NO_2$	160.040402	-0.191	C98C5'	
161.024421	63.3	$C_9H_5O_3$	161.024418	-0.017	C95C8'/C96C7'	
162.019657	22.4	$C_8H_4NO_3$	162.019667	0.064	C84C90	
174.019662	80.8	$C_9H_4NO_3$	174.019667	0.030	C96C6'	
176.035316	405.7	$C_9H_6NO_3$	176.035317	0.004	[C99C8'+H_2]	✱
186.056050	46.1	$C_{11}H_8NO_2$	186.056052	0.010	C92C10'	
188.035319	52.1	$C_{10}H_6NO_3$	188.035317	-0.012	C94C8'	
190.050976	15.4	$C_{10}H_8NO_3$	190.050967	-0.046	C96C5'	
200.071702	32.0	$C_{12}H_{10}NO_2$	200.071702	-0.002	C98C4'	
201.030551	20.4	$C_{10}H_5N_2O_3$	201.030566	0.073	C87C10'	
202.014582	69.5	$C_{10}H_4NO_4$	202.014581	-0.007	C99C7'	✱
202.050971	25.9	$C_{11}H_8NO_3$	202.050967	-0.022	C93C8'	
212.035313	27.1	$C_{12}H_6NO_3$	212.035317	0.020	C90C10'	
220.025118	10.1	$C_{10}H_6NO_5$	220.025146	0.125	C86C91C7'	
226.050993	16.4	$C_{13}H_8NO_3$	226.050967	-0.113	[C92C7'-OH]	
229.061859	8.0	$C_{12}H_9N_2O_3$	229.061866	0.031	[C79-OH_2]	
230.045877	884.1	$C_{12}H_8NO_4$	230.045881	0.019	C90C9'	
243.070994	56.5	$C_{12}H_{11}N_4S$	243.070991	-0.011	C2C21	
243.077512	114.5	$C_{13}H_{11}N_2O_3$	243.077516	0.018	[C98C2'-H_2]	
244.061538	76.8	$C_{13}H_{10}NO_4$	244.061531	-0.027	C90C8'/C92C7'	
259.072428	50.3	$C_{13}H_{11}N_2O_4$	259.072430	0.008	[C99C41'+H]	
269.056769	17.5	$C_{14}H_9N_2O_4$	269.056780	0.039	[C9'-OH]	
270.040818	15.0	$C_{14}H_8NO_5$	270.040796	-0.081	C85C8'	
273.051616	11063.9	$C_{13}H_9N_2O_5$	273.051695	0.290	[C9'+H_2]	
273.088091	76.1	$C_{14}H_{13}N_2O_4$	273.088081	-0.037	C99C4'	
285.051699	151.3	$C_{14}H_9N_2O_5$	285.051695	-0.013	C8'	
287.067317	2603.3	$C_{14}H_{11}N_2O_5$	287.067345	0.097	[C8'+H_2]	
299.067350	80.7	$C_{15}H_{11}N_2O_5$	299.067345	-0.018	[C7'-O]	
300.025542	303.4	$C_{16}H_{11}ClNOS$	300.025536	-0.020	C1C17	
300.135389	24.2	$C_{16}H_{18}N_3O_3$	300.135365	-0.081	[C26C96C20'-O]	
304.130275	23.1	$C_{15}H_{18}N_3O_4$	304.130280	0.016	[C99C3'-OH]	
313.046636	42.3	$C_{15}H_9N_2O_6$	313.046610	-0.082	C7'	
314.151052	23.2	$C_{17}H_{20}N_3O_3$	314.151015	-0.117	[C94C3'-O_2H]	
326.114633	109.1	$C_{17}H_{16}N_3O_4$	326.114630	-0.010	[C94C3'-OH]	
330.073167	22.7	$C_{15}H_{12}N_3O_6$	330.073159	-0.024	[C6'+H]	
330.109551	71.7	$C_{16}H_{16}N_3O_5$	330.109544	-0.022	C94C2'	
340.130266	77.3	$C_{18}H_{18}N_3O_4$	340.130280	0.040	[C93C3'-OH]	
341.063296	16.9	$C_{17}H_{14}ClN_4S$	341.063319	0.067	[C3+H_2]	
341.114213	7.7	$C_{18}H_{17}N_2O_5$	341.114295	0.239	C15C25C95C16'	
347.070520	12.4	$C_{19}H_{12}ClN_4O$	347.070512	-0.022	[C1C34-H_2]	
351.109871	21.2	$C_{18}H_{15}N_4O_4$	351.109879	0.024	[C26C94C20'-OH]	
353.063327	46.0	$C_{18}H_{14}ClN_4S$	353.063319	-0.024	[C2-H_2]	
354.120797	80.8	$C_{17}H_{16}N_5O_4$	354.120778	-0.053	C96C14'C20'	
355.078968	899.5	$C_{18}H_{16}ClN_4S$	355.078969	0.002	C2	✱
356.125208	13.0	$C_{18}H_{18}N_3O_5$	356.125194	-0.038	[C90C1'-H_2]	
357.076034	142.0	$C_{17}H_{14}ClN_4O_3$	357.075992	-0.118	C10C15C18'	
357.094683	20.5	$C_{18}H_{18}ClN_4S$	357.094619	-0.178	[C2+H_4]	
357.156806	9.9	$C_{18}H_{21}N_4O_4$	357.156829	0.065	[C89C1'-OH]	

358.140854	461.4	$C_{18}H_{20}N_3O_5$	358.140844	-0.029	C90C1'/C92C2'	
366.109622	7.4	$C_{19}H_{16}N_3O_5$	366.109544	-0.213	C23C26C95	
369.120444	66.3	$C_{18}H_{17}N_4O_5$	369.120443	-0.003	C15C21C96C18'	
381.058214	90.2	$C_{19}H_{14}ClN_4OS$	381.058234	0.052	C1	
382.104516	9.1	$C_{19}H_{16}N_3O_6$	382.104459	-0.149	[C4'-H_2]	
383.136056	45.8	$C_{19}H_{19}N_4O_5$	383.136093	0.098	[C1'-OH]	
384.120076	997.8	$C_{19}H_{18}N_3O_6$	384.120109	0.086	C4'	
395.073870	12.7	$C_{20}H_{16}ClN_4OS$	395.073884	0.034	C12C41	
395.183665	21.0	$C_{20}H_{23}N_6O_3$	395.183712	0.119	C10C23C34	
397.115613	7.4	$C_{19}H_{17}N_4O_6$	397.115358	-0.643	[C1'-H_3]	
399.131008	18.4	$C_{19}H_{19}N_4O_6$	399.131008	0.001	[C1'-H]	
401.146637	652.0	$C_{19}H_{21}N_4O_6$	401.146658	0.051	C1'	
421.163093	8.2	$C_{21}H_{21}N_6O_4$	421.162977	-0.276	C23C96C18'	
424.173899	40.4	$C_{20}H_{22}N_7O_4$	424.173876	-0.055	C99C18'C20'	
427.125884	1973.2	$C_{20}H_{19}N_4O_7$	427.125922	0.089	C2'	
439.173521	30.6	$C_{21}H_{23}N_6O_5$	439.173541	0.045	C95C18'	
452.131751	11.8	$C_{23}H_{23}ClN_5OS$	452.131733	-0.039	C6	
461.149821	10.5	$C_{24}H_{22}ClN_6O_2$	461.149825	0.008	C8C34	
469.158275	82.7	$C_{23}H_{26}ClN_6OS$	469.158282	0.015	[C7+H_2]	
481.158313	12.8	$C_{24}H_{26}ClN_6OS$	481.158282	-0.064	C8C11	
482.179346	123.4	$C_{22}H_{24}N_7O_6$	482.179355	0.019	C18C20C83	
491.168800	7.0	$C_{24}H_{23}N_6O_6$	491.168456	-0.701	C17C21C18'	
495.137551	228.6	$C_{24}H_{24}ClN_6O_2S$	495.137546	-0.009	C8	*
507.137536	37.1	$C_{25}H_{24}ClN_6O_2S$	507.137546	0.019	[C9-H_4]	
509.153186	104.7	$C_{25}H_{26}ClN_6O_2S$	509.153197	0.023	[C9-H_2]	*
511.132256	11.3	$C_{24}H_{24}ClN_6O_3S$	511.132461	0.401	[C10-Me]	
525.148136	65.6	$C_{25}H_{26}ClN_6O_3S$	525.148111	-0.047	C10	
545.197766	12.5	$C_{28}H_{29}N_6O_4S$	545.197648	-0.216	C39C96	
556.073937	66.7	$C_{29}H_{19}ClN_3O_5S$	556.073943	0.011	[C13C17C95-H_4]	
568.121531	6.8	$C_{30}H_{23}ClN_5O_3S$	568.121562	0.055		
572.105204	14.4	$C_{30}H_{23}ClN_3O_5S$	572.105243	0.068	[C17C95-H_4]	
577.140048	8.4	$C_{31}H_{22}ClN_6O_4$	577.139654	-0.682		
588.203667	9.8	$C_{29}H_{30}N_7O_5S$	588.203462	-0.348	C39C99	
590.152214	7.5	$C_{31}H_{29}ClN_3O_5S$	590.152193	-0.036		
596.103975	17.6	$C_{33}H_{18}N_5O_5S$	596.103413	-0.942	[C22C37C99-H_{12}]	
599.152546	11.5	$C_{32}H_{28}ClN_4O_4S$	599.152528	-0.030	[C22C27C96-H_2]	
601.179404	15.3	$C_{31}H_{30}ClN_6O_3S$	601.179411	0.011	C98	
603.203168	26.7	$C_{30}H_{31}N_6O_6S$	603.203127	-0.069	C21C37C82	
611.127400	106.5	$C_{31}H_{24}ClN_6O_4S$	611.127376	-0.040	[C96C13-H_2]	
613.168458	10.1	$C_{33}H_{30}ClN_4O_4S$	613.168178	-0.457	[C37C95-OH]	
614.174746	9.9	$C_{31}H_{29}ClN_7O_3S$	614.174660	-0.140	[C81C99-Me][C81C99-OH]	
621.165881	11.4	$C_{33}H_{26}ClN_6O_5$	621.165869	-0.019	[C95-H_4]	
627.158792	9.1	$C_{32}H_{28}ClN_6O_4S$	627.158676	-0.184	[C96-H_2]	
628.190280	55.3	$C_{32}H_{31}ClN_7O_3S$	628.190310	0.048	[C81C99-OH]	
636.195673	7.5	$C_{34}H_{31}ClN_7O_2S$	636.195396	-0.435		
637.106873	14.7	$C_{32}H_{22}ClN_6O_5S$	637.106640	-0.366	[C95-Me][C95-H_4]	
638.211102	23.9	$C_{34}H_{33}ClN_7O_2S$	638.211046	-0.088	[C94-O_4H]	
644.185193	11.8	$C_{32}H_{31}ClN_7O_4S$	644.185225	0.050	[C81C94-Me]	
646.208981	50.1	$C_{31}H_{32}N_7O_7S$	646.208941	-0.062	C25	
646.224174	20.2	$C_{35}H_{32}N_7O_4S$	646.224197	0.036	[C22C93-OH]	
650.219172	60.4	$C_{34}H_{32}N_7O_5S$	650.219112	-0.092	C22C94	
655.153500	972.9	$C_{33}H_{28}ClN_6O_5S$	655.153590	0.137	C95	
664.190400	50.1	$C_{35}H_{31}ClN_7O_3S$	664.190310	-0.135	[C93-O_2H]	
665.193656	17.9	$C_{33}H_{29}N_8O_6S$	665.193625	-0.047	C13C32C87	
672.180269	55.5	$C_{33}H_{31}ClN_7O_5S$	672.180140	-0.192	[C99+H_2]	
678.206169	16.8	$C_{36}H_{33}ClN_7O_3S$	678.205960	-0.309	[C92-O_2H]	

682.200798	1019.8	$C_{35}H_{33}ClN_7O_4S$	682.200875	0.113	[C93-OH]	
686.195739	435.4	$C_{34}H_{33}ClN_7O_5S$	686.195790	0.074	C94	
691.219083	16.1	$C_{36}H_{32}ClN_8O_5$	691.218967	-0.168	[C82-H_2]	
696.216486	217.5	$C_{36}H_{35}ClN_7O_4S$	696.216525	0.056	[C92-OH]	
698.213484	49.1	$C_{35}H_{33}ClN_7O_7$	698.213548	0.092	C34C36	
701.167282	9.0	$C_{36}H_{26}ClN_8O_6$	701.166932	-0.499	[C29-O_2H][C29-H_4]	
704.185303	37.5	$C_{37}H_{31}ClN_7O_4S$	704.185225	-0.110	[C13-O_3H]	
704.229722	92.4	$C_{37}H_{34}N_7O_6S$	704.229677	-0.064	C22C90	
707.196248	44.9	$C_{36}H_{32}ClN_8O_4S$	707.196124	-0.175	[C82-O_2H]	
709.211928	25.6	$C_{36}H_{34}ClN_8O_4S$	709.211774	-0.217	[C82-O_2]	
711.191162	20.2	$C_{35}H_{32}ClN_8O_5S$	711.191039	-0.173	[C87-O]	
712.211425	18.9	$C_{36}H_{35}ClN_7O_5S$	712.211440	0.021	C92	*
722.195755	179.9	$C_{37}H_{33}ClN_7O_5S$	722.195790	0.048	[C90-OH]	
725.206582	795.0	$C_{36}H_{34}ClN_8O_5S$	725.206689	0.148	C82C89	
739.222407	55.3	$C_{37}H_{36}ClN_8O_5S$	739.222339	-0.092	[C81-O]	
740.206015	4363.5	$C_{37}H_{35}ClN_7O_6S$	740.206354	0.458	C90	
743.206667	22.4	$C_{37}H_{36}ClN_6O_7S$	743.206020	-0.871	C14C31	
747.235477	41.0	$C_{38}H_{35}N_8O_7S$	747.235490	0.017	[C22-H_2]	
749.224432	34.5	$C_{38}H_{34}ClN_8O_7$	749.224447	0.019	[C34-H_2]	
755.217260	71.7	$C_{37}H_{36}ClN_8O_6S$	755.217253	-0.009	[M-CO_2]	*
765.201624	49.6	$C_{38}H_{34}ClN_8O_6S$	765.201603	-0.028	[M-OH_2]	*
783.210623	11713.0	$C_{38}H_{36}ClN_8O_7S$	783.212168	1.972	[M-H]	
			Abs mean error	0.112		
			Mean std dev	0.161		

Table S14 showing peak list, signal-to-noise ratio (S/N), elemental composition, and assignment with mass errors (ppm) of the [M-H]⁻ dBET1 compound by IRMPD MS/MS, calibration points are marked by an asterisk (✱).

Measured *m/z*	S/N	Elemental Composition	Theoretical *m/z*	Assignment Error (ppm)	Assignment	
100.004011	13.6	$C_3H_2NO_3$	100.004017	0.064		
107.036314	63.3	$C_4H_3N_4$	107.036320	0.056	C3C18'C20'	
123.067621	14.0	$C_5H_7N_4$	123.067620	-0.010	C2C18'C20'	
127.051297	23.4	$C_5H_7N_2O_2$	127.051301	0.029	[C95'+H_2]	
128.035313	244.0	$C_5H_6NO_3$	128.035317	0.028	[C88C96C6'+H_2]	
130.029842	11.8	C_8H_4NO	130.029837	-0.037	C1C18'C23	
148.040402	23.1	$C_8H_6NO_2$	148.040402	0.001	C6'C98	
153.030571	10.5	$C_6H_5N_2O_3$	153.030566	-0.033	C96'	
158.024764	11.8	$C_9H_4NO_2$	158.024752	-0.077	C94C10'	
160.040402	34.6	$C_9H_6NO_2$	160.040402	-0.001	C98C5'	
161.024406	15.1	$C_9H_5O_3$	161.024418	0.076	C95C8'/C96C7'	
162.019639	15.2	$C_8H_4NO_3$	162.019667	0.174	C84C90	
174.019671	75.4	$C_9H_4NO_3$	174.019667	-0.025	C96C6'	
174.056048	12.1	$C_{10}H_8NO_2$	174.056052	0.025	C98C40'	
176.035319	468.6	$C_9H_6NO_3$	176.035317	-0.009	[C99C8'+H_2]	✱
186.056052	246.6	$C_{11}H_8NO_2$	186.056052	-0.002	C92C10'	
188.035314	55.9	$C_{10}H_6NO_3$	188.035317	0.015	C94C8'	✱
190.050971	190.2	$C_{10}H_8NO_3$	190.050967	-0.022	C96C5'	
199.087693	14.9	$C_{12}H_{11}N_2O$	199.087687	-0.030	C22C26C40	
200.071704	31.1	$C_{12}H_{10}NO_2$	200.071702	-0.009	C98C4'	
202.014582	283.6	$C_{10}H_4NO_4$	202.014581	-0.005	C99C7'	✱
202.050971	118.0	$C_{11}H_8NO_3$	202.050967	-0.020	C93C8'	
212.035326	286.3	$C_{12}H_6NO_3$	212.035317	-0.042	C90C10'	
214.050972	51.8	$C_{12}H_8NO_3$	214.050967	-0.022	[C90C10'+H_2]	
216.030241	44.6	$C_{11}H_6NO_4$	216.030231	-0.045	C94C7'	
226.050982	80.0	$C_{13}H_8NO_3$	226.050967	-0.067	[C92C7'-OH]	
228.030262	12.1	$C_{12}H_6NO_4$	228.030231	-0.138	C93C7'	
228.066626	17.6	$C_{13}H_{10}NO_3$	228.066617	-0.038	C96C4'	
230.045882	1136.1	$C_{12}H_8NO_4$	230.045881	-0.005	C90C9'	
242.045872	34.8	$C_{13}H_8NO_4$	242.045881	0.036	C95C41'	
243.070991	95.8	$C_{12}H_{11}N_4S$	243.070991	0.000	C2C21	
243.077524	96.9	$C_{13}H_{11}N_2O_3$	243.077516	-0.035	[C98C2'-H_2]	
244.061540	439.4	$C_{13}H_{10}NO_4$	244.061531	-0.038	C90C8'/C92C7'	
251.046233	11.9	$C_{14}H_7N_2O_3$	251.046216	-0.069	[C95C4'-OH][C95C4'-H_4]	
253.134658	72.1	$C_{16}H_{17}N_2O$	253.134637	-0.084	C6C31C32C42	
256.145558	24.7	$C_{15}H_{18}N_3O$	256.145536	-0.087	C6C28C31C42	
257.059965	63.1	$C_{13}H_{10}ClN_4$	257.059948	-0.068		
258.088460	16.1	$C_{13}H_{12}N_3O_3$	258.088415	-0.173	C6C30C31C42	
270.040814	31.6	$C_{14}H_8NO_5$	270.040796	-0.065	C85C8'	
270.161184	60.7	$C_{16}H_{20}N_3O$	270.161186	0.007		
273.051646	7926.3	$C_{13}H_9N_2O_5$	273.051695	0.181	[C9'+H_2]	
273.088097	140.9	$C_{14}H_{13}N_2O_4$	273.088081	-0.060	C99C4'	
277.073099	39.9	$C_{15}H_9N_4O_2$	277.073099	-0.001		
280.141522	25.0	$C_{12}H_{18}N_5O_3$	280.141513	-0.031		
282.124798	84.6	$C_{16}H_{16}N_3O_2$	282.124800	0.006		
283.108816	108.6	$C_{16}H_{15}N_2O_3$	283.108816	0.000	[C93C4'-OH]	
287.067343	1120.3	$C_{14}H_{11}N_2O_5$	287.067345	0.007	[C8'+H_2]	✱
289.032041	50.3	$C_{13}H_{10}ClN_4S$	289.032019	-0.075	C3C27	
296.140459	59.8	$C_{17}H_{18}N_3O_2$	296.140450	-0.031	C8C31C32C42	
298.119740	10.1	$C_{16}H_{16}N_3O_3$	298.119715	-0.084	C10C26C22	
299.067347	660.4	$C_{15}H_{11}N_2O_5$	299.067345	-0.007	[C7'-O]	
300.025548	134.8	$C_{16}H_{11}ClNOS$	300.025536	-0.041	C1C17	

300.135378	51.8	$C_{16}H_{18}N_3O_3$	300.135365	-0.044	[C26C96C20'-O]	
304.130303	16.0	$C_{15}H_{18}N_3O_4$	304.130280	-0.075	[C99C3'-OH]	
310.119726	133.1	$C_{17}H_{16}N_3O_3$	310.119715	-0.036	[C82C4'-O_2]	
313.046686	14.5	$C_{15}H_9N_2O_6$	313.046610	-0.242	C7'	
314.151025	130.2	$C_{17}H_{20}N_3O_3$	314.151015	-0.031	[C94C3'-O_2H]	
321.091300	36.8	$C_7H_{13}N_8O_7$	321.091268	-0.099		
322.119727	33.2	$C_{18}H_{16}N_3O_3$	322.119715	-0.036	[C10C22C31C42-H_2]	
324.135397	20.5	$C_{18}H_{18}N_3O_3$	324.135365	-0.100	C10C22C31C42	
326.114626	295.0	$C_{17}H_{16}N_3O_4$	326.114630	0.013	[C94C3'-OH]	
330.109544	47.1	$C_{16}H_{16}N_3O_5$	330.109544	-0.000	C94C2'	
333.054784	10.8	$C_{18}H_{10}ClN_4O$	333.054862	0.234		
335.106897	10.6	$C_{19}H_{16}ClN_4$	335.106898	0.004		
336.098996	14.3	$C_{18}H_{14}N_3O_4$	336.098980	-0.048		
339.047660	40.1	$C_{17}H_{12}ClN_4S$	339.047669	0.027	C3	
340.130279	265.6	$C_{18}H_{18}N_3O_4$	340.130280	0.003	[C93C3'-OH]	
341.063324	22.9	$C_{17}H_{14}ClN_4S$	341.063319	-0.015	[C3+H_2]	
341.114395	11.9	$C_{18}H_{17}N_2O_5$	341.114295	-0.292	C15C25C95C16'	
342.145927	85.1	$C_{18}H_{20}N_3O_4$	342.145930	0.009	[C89C4'-O]	
347.070490	12.2	$C_{19}H_{12}ClN_4O$	347.070512	0.064	[C1C34-H_2]	
351.011275	10.9	$C_{17}H_8ClN_4OS$	351.011283	0.024	C1C13C13	
353.063336	73.0	$C_{18}H_{14}ClN_4S$	353.063319	-0.048	[C2-H_2]	
354.120794	141.6	$C_{17}H_{16}N_5O_4$	354.120778	-0.045	C96C14'C20'	
355.078967	1033.4	$C_{18}H_{16}ClN_4S$	355.078969	0.006	C2	*
356.125214	20.2	$C_{18}H_{18}N_3O_5$	356.125194	-0.056	[C90C1'-H_2]	
357.076030	195.9	$C_{17}H_{14}ClN_4O_3$	357.075992	-0.107	C10C15C18'	
357.094564	14.3	$C_{18}H_{18}ClN_4S$	357.094619	0.153	[C2+H_4]	
358.140862	266.4	$C_{18}H_{20}N_3O_5$	358.140844	-0.050	C90C1'/C92C2'	
366.034787	43.0	$C_{29}H_4N$	366.034923	0.372		
366.109523	12.1	$C_{19}H_{16}N_3O_5$	366.109544	0.057	C23C26C95	
367.078978	16.4	$C_{19}H_{16}ClN_4S$	367.078969	-0.025	[C1-OH]	
369.120425	11.7	$C_{18}H_{17}N_4O_5$	369.120443	0.049	C15C21C96C18'	
381.058240	134.8	$C_{19}H_{14}ClN_4OS$	381.058234	-0.015	C1	
383.136136	25.0	$C_{19}H_{19}N_4O_5$	383.136093	-0.112	[C1'-OH]	
384.120087	942.1	$C_{19}H_{18}N_3O_6$	384.120109	0.056	C4'	
395.073910	79.7	$C_{20}H_{16}ClN_4OS$	395.073884	-0.067	C12C41	
395.183705	23.6	$C_{20}H_{23}N_6O_3$	395.183712	0.018	C10C23C34	
401.146666	71.2	$C_{19}H_{21}N_4O_6$	401.146658	-0.021	C1'	*
412.123827	12.3	$C_{23}H_{18}N_5OS$	412.123755	-0.173		
421.163005	41.8	$C_{21}H_{21}N_6O_4$	421.162977	-0.066	C23C96C18'	
424.173885	30.6	$C_{20}H_{22}N_7O_4$	424.173876	-0.021	C99C18'C20'	
427.125934	507.1	$C_{20}H_{19}N_4O_7$	427.125922	-0.028	C2'	
460.173869	16.3	$C_{23}H_{22}N_7O_4$	460.173876	0.015		
466.053448	13.7	$C_{25}H_{13}ClN_5OS$	466.053482	0.074		
469.078287	105.5	$C_{27}H_{18}ClN_2O_2S$	469.078300	0.028		
469.158319	52.1	$C_{23}H_{26}ClN_6OS$	469.158282	-0.079	[C7+H_2]	
475.165449	25.9	$C_{25}H_{24}ClN_6O_2$	475.165475	0.054	[C9C34-H_2]	
482.179392	21.7	$C_{22}H_{24}N_7O_6$	482.179355	-0.078	C18C20C83	
487.160984	68.3	$C_{21}H_{24}ClN_8O_4$	487.161453	0.962		
490.143994	13.8	$C_{29}H_{21}ClN_5O$	490.144012	0.037		
491.168414	11.5	$C_{24}H_{23}N_6O_6$	491.168456	0.086	C17C21C18'	
495.137607	30.9	$C_{24}H_{24}ClN_6O_2S$	495.137546	-0.122	C8	
499.119402	15.8	$C_{25}H_{19}N_6O_4S$	499.119398	-0.007		
509.153205	118.4	$C_{25}H_{26}ClN_6O_2S$	509.153197	-0.015	[C9-H_2]	*
512.140307	76.5	$C_{35}H_{18}N_3O_2$	512.140450	0.279		
516.123333	19.0	$C_{30}H_{19}ClN_5O_2$	516.123276	-0.110		
524.131750	41.6	$C_{29}H_{23}ClN_5OS$	524.131733	-0.032		
525.135150	12.5	$C_{27}H_{21}N_6O_4S$	525.135048	-0.194	C13C13C30C96	
525.148129	19.5	$C_{25}H_{26}ClN_6O_3S$	525.148111	-0.034	C10	
534.133698	12.8	$C_{30}H_{21}ClN_5O_3$	534.133841	0.267		

547.109981	17.5	$C_{29}H_{24}ClN_2O_5S$	547.109994	0.024		
550.110966	45.5	$C_{30}H_{21}ClN_5O_2S$	550.110997	0.056		
556.073894	473.8	$C_{29}H_{19}ClN_3O_5S$	556.073943	0.088	[C13C17C95-H_4]	
566.106155	10.2	$C_{30}H_{21}ClN_5O_3S$	566.105912	-0.428		
568.121531	31.4	$C_{30}H_{23}ClN_5O_3S$	568.121562	0.054		
572.105221	17.3	$C_{30}H_{23}ClN_3O_5S$	572.105243	0.039	[C17C95-H_4]	
577.139547	16.6	$C_{31}H_{22}ClN_6O_4$	577.139654	0.186		
594.100791	45.9	$C_{31}H_{21}ClN_5O_4S$	594.100827	0.061		
599.152497	13.8	$C_{32}H_{28}ClN_4O_4S$	599.152528	0.051	[C22C27C96-H_2]	
603.118980	11.2	$C_{32}H_{20}ClN_6O_5$	603.118919	-0.101		
611.127355	159.0	$C_{31}H_{24}ClN_6O_4S$	611.127376	0.035	[C96C13-H_2]	
613.168011	17.2	$C_{33}H_{30}ClN_4O_4S$	613.168178	0.273	[C37C95-OH]	
637.106458	17.1	$C_{32}H_{22}ClN_6O_5S$	637.106640	0.286	[C95-Me][C95-H_4]	
638.211116	46.7	$C_{34}H_{33}ClN_7O_2S$	638.211046	-0.110	[C94-O_4H]	
640.208399	11.7	$C_{33}H_{31}ClN_7O_5$	640.208068	-0.516	C34C99	
646.224271	24.3	$C_{35}H_{32}N_7O_4S$	646.224197	-0.115	[C22C93-OH]	
650.219181	23.6	$C_{34}H_{32}N_7O_5S$	650.219112	-0.106	C22C94	
654.169510	11.8	$C_{33}H_{29}ClN_7O_4S$	654.169575	0.099		
655.153573	169.9	$C_{33}H_{28}ClN_6O_5S$	655.153590	0.027	C95	*
664.190289	55.8	$C_{35}H_{31}ClN_7O_3S$	664.190310	0.031	[C93-O_2H]	
665.193588	19.0	$C_{33}H_{29}N_8O_6S$	665.193625	0.056	C13C32C87	
672.179822	12.5	$C_{33}H_{31}ClN_7O_5S$	672.180140	0.473	[C99+H_2]	
682.200851	189.0	$C_{35}H_{33}ClN_7O_4S$	682.200875	0.035	[C93-OH]	
686.195829	48.6	$C_{34}H_{33}ClN_7O_5S$	686.195790	-0.057	C94	
696.216523	59.5	$C_{36}H_{35}ClN_7O_4S$	696.216525	0.003	[C92-OH]	
698.213653	14.6	$C_{35}H_{33}ClN_7O_7$	698.213548	-0.150	C34C36	
704.185035	11.1	$C_{37}H_{31}ClN_7O_4S$	704.185225	0.270	[C13-O_3H]	
704.229644	22.9	$C_{37}H_{34}N_7O_6S$	704.229677	0.047	C22C90	
707.195981	10.4	$C_{36}H_{32}ClN_8O_4S$	707.196124	0.202	[C82-O_2H]	
722.195824	87.3	$C_{37}H_{33}ClN_7O_5S$	722.195790	-0.047	[C90-OH]	
725.206629	102.5	$C_{36}H_{34}ClN_8O_5S$	725.206689	0.082	C89C89	
739.221940	15.3	$C_{37}H_{36}ClN_8O_5S$	739.222339	0.540	[C81-O]	
740.206219	932.9	$C_{37}H_{35}ClN_7O_6S$	740.206354	0.183	C90	
747.235564	10.5	$C_{38}H_{35}N_8O_7S$	747.235490	-0.099	[C22-H_2]	
765.201540	37.0	$C_{38}H_{34}ClN_8O_6S$	765.201603	0.082	[M-OH_2]	
783.211141	14142.8	$C_{38}H_{36}ClN_8O_7S$	783.212168	1.312	[M-H]	
			Abs mean error	0.076		
			Mean std dev	0.096		

Table S15 showing peak list, signal-to-noise ratio (S/N), elemental composition, and assignment with mass errors (ppm) of the [M-H]- dBET1 compound by UVPD MS/MS, calibration points are marked by an asterisk (✱).

Measured *m/z*	S/N	Elemental Composition	Theoretical *m/z*	Assignment Error (ppm)	Assignment	
128.035314	58.3	$C_5H_6NO_3$	128.035317	0.020	[C88C96C6'+H_2]	
160.040398	9.4	$C_9H_6NO_2$	160.040402	0.026	C98C5'	
161.011837	27.5	$C_8H_3NO_3$	161.011842	0.032	C99C9'	
162.019666	68.1	$C_8H_4NO_3$	162.019667	0.006	C84C90	
174.019665	39.6	$C_9H_4NO_3$	174.019667	0.010	C96C6'	✱
176.035316	382.5	$C_9H_6NO_3$	176.035317	0.006	[C99C8'+H_2]	
185.048239	13.9	$C_{11}H_7NO_2$	185.048227	-0.067	[C92C10'-H]	
186.056044	53.2	$C_{11}H_8NO_2$	186.056052	0.043	C92C10'	
188.035316	117.7	$C_{10}H_6NO_3$	188.035317	0.004	C94C8'	
190.050966	15.0	$C_{10}H_8NO_3$	190.050967	0.007	C96C5'	
200.071701	22.2	$C_{12}H_{10}NO_2$	200.071702	0.005	C98C4'	
201.030561	20.2	$C_{10}H_5N_2O_3$	201.030566	0.026	C87C10'	
201.043140	7.9	$C_{11}H_7NO_3$	201.043142	0.008	C93C8'	
202.014586	66.2	$C_{10}H_4NO_4$	202.014581	-0.027	C99C7'	✱
212.035314	26.0	$C_{12}H_6NO_3$	212.035317	0.016	C90C10'	
216.030226	9.6	$C_{11}H_6NO_4$	216.030231	0.025	C94C7'	
216.060070	10.8	$C_{11}H_{10}N_3S$	216.060092	0.100	C2C25	
226.050972	18.3	$C_{13}H_8NO_3$	226.050967	-0.023	[C92C7'-OH]	
228.030224	16.6	$C_{12}H_6NO_4$	228.030231	0.031	C93C7'	
229.061903	7.7	$C_{12}H_9N_2O_3$	229.061866	-0.161	[C79-OH_2]	
230.045881	617.0	$C_{12}H_8NO_4$	230.045881	-0.001	C90C9'	
243.070987	92.1	$C_{12}H_{11}N_4S$	243.070991	0.016	C2C21	
243.077547	15.4	$C_{13}H_{11}N_2O_3$	243.077516	-0.126	[C98C2'-H_2]	
244.061536	25.1	$C_{13}H_{10}NO_4$	244.061531	-0.020	C90C8'/C92C7'	
258.088492	8.9	$C_{13}H_{12}N_3O_3$	258.088415	-0.297	C6C30C31C42	
259.072441	9.4	$C_{13}H_{11}N_2O_4$	259.072430	-0.042	[C99C41'+H]	
270.040810	12.1	$C_{14}H_8NO_5$	270.040796	-0.050	C85C8'	
272.030597	7.7	$C_{15}H_{11}ClNS$	272.030622	0.093	C14C20	
272.104060	12.2	$C_{14}H_{14}N_3O_3$	272.104065	0.019	[C99C4'-O_2]	
273.051688	2692.2	$C_{13}H_9N_2O_5$	273.051695	0.025	[C9'+H_2]	✱
273.088090	38.9	$C_{14}H_{13}N_2O_4$	273.088081	-0.032	C99C4'	
283.108803	18.2	$C_{16}H_{15}N_2O_3$	283.108816	0.046	[C93C4'-OH]	
286.059529	11.1	$C_{14}H_{10}N_2O_5$	286.059520	-0.031	[C8'+H]	
287.067343	692.9	$C_{14}H_{11}N_2O_5$	287.067345	0.006	[C8'+H_2]	✱
298.119721	8.7	$C_{16}H_{16}N_3O_3$	298.119715	-0.020	C10C26C22	
299.067331	58.0	$C_{15}H_{11}N_2O_5$	299.067345	0.048	[C7'-O]	
300.025527	60.6	$C_{16}H_{11}ClNOS$	300.025536	0.030	C1C17	
300.135360	56.2	$C_{16}H_{18}N_3O_3$	300.135365	0.017	[C26C96C20'-O]	
313.046561	13.8	$C_{15}H_9N_2O_6$	313.046610	0.157	C7'	
321.091250	28.1	$C_{18}H_{14}ClN_4$	321.091248	-0.006		
326.114592	28.4	$C_{17}H_{16}N_3O_4$	326.114630	0.116	[C94C3'-OH]	
339.047650	17.1	$C_{17}H_{12}ClN_4S$	339.047669	0.055	C3	
340.055495	21.4	$C_{17}H_{13}ClN_4S$	340.055494	-0.003	[C3+H]	
340.130285	19.6	$C_{18}H_{18}N_3O_4$	340.130280	-0.016	[C93C3'-OH]	
341.063367	12.0	$C_{17}H_{14}ClN_4S$	341.063319	-0.142	[C3+H_2]	
347.070539	18.6	$C_{19}H_{12}ClN_4O$	347.070512	-0.077	[C1C34-H_2]	
353.063339	21.5	$C_{18}H_{14}ClN_4S$	353.063319	-0.056	[C2-H_2]	
354.120826	11.9	$C_{17}H_{16}N_5O_4$	354.120778	-0.137	C96C14'C20'	
355.078973	152.4	$C_{18}H_{16}ClN_4S$	355.078969	-0.010	C2	
357.076012	47.7	$C_{17}H_{14}ClN_4O_3$	357.075992	-0.056	C10C15C18'	
358.140864	91.7	$C_{18}H_{20}N_3O_5$	358.140844	-0.055	C90C1'/C92C2'	
381.058189	19.3	$C_{19}H_{14}ClN_4OS$	381.058234	0.118	C1	
383.136158	6.4	$C_{19}H_{19}N_4O_5$	383.136093	-0.169	[C1'-OH]	

384.120112	264.5	$C_{19}H_{18}N_3O_6$	384.120109	-0.007	C4'	∗
401.146641	82.8	$C_{19}H_{21}N_4O_6$	401.146658	0.042	C1'	
427.125925	357.7	$C_{20}H_{19}N_4O_7$	427.125922	-0.007	C2'	∗
469.158293	13.8	$C_{23}H_{26}ClN_6OS$	469.158282	-0.024	[C7+H_2]	
482.179313	21.7	$C_{22}H_{24}N_7O_6$	482.179355	0.088	C18C20C83	
495.137687	22.1	$C_{24}H_{24}ClN_6O_2S$	495.137546	-0.285	C8	
509.153268	17.6	$C_{25}H_{26}ClN_6O_2S$	509.153197	-0.139	[C9-H_2]	
556.074004	37.2	$C_{29}H_{19}ClN_3O_5S$	556.073943	-0.110	[C13C17C95-H_4]	
611.127397	28.4	$C_{31}H_{24}ClN_6O_4S$	611.127376	-0.034	[C96C13-H_2]	
650.219465	8.4	$C_{34}H_{32}N_7O_5S$	650.219112	-0.543	C22C94	
655.153620	98.9	$C_{33}H_{28}ClN_6O_5S$	655.153590	-0.045	C95	∗
672.180078	7.3	$C_{33}H_{31}ClN_7O_5S$	672.180140	0.093	[C99+H_2]	
682.200907	158.0	$C_{35}H_{33}ClN_7O_4S$	682.200875	-0.048	[C93-OH]	
686.195961	39.8	$C_{34}H_{33}ClN_7O_5S$	686.195790	-0.249	C94	
696.216784	16.4	$C_{36}H_{35}ClN_7O_4S$	696.216525	-0.372	[C92-OH]	
704.229878	11.8	$C_{37}H_{34}N_7O_6S$	704.229677	-0.285	C22C90	
722.195840	26.4	$C_{37}H_{33}ClN_7O_5S$	722.195790	-0.069	[C90-OH]	
725.206657	72.9	$C_{36}H_{34}ClN_8O_5S$	725.206689	0.045	C89C89	∗
740.206421	191.8	$C_{37}H_{35}ClN_7O_6S$	740.206354	-0.090	C90	
748.243878	12.2	$C_{38}H_{36}N_8O_7S$	748.243315	-0.753	C22	
765.202051	6.2	$C_{38}H_{34}ClN_8O_6S$	765.201603	-0.585	[M-OH_2]	
783.212089	7889.1	$C_{38}H_{36}ClN_8O_7S$	783.212168	0.101	[M-H]	
			Abs mean error	0.084		
			Mean std dev	0.124		

Supplementary tables for 2DMS of dBET1

Table S16 showing peak list, signal-to-noise ratio (S/N), and possible elemental composition with mass errors (ppm) of the hydrolysed dBET1 compound by 2DMS in tandem with UVPD, calibrated by the precursor at 785.226723 *m/z*.

Measured *m/z*	S/N	Elemental Composition	Theoretical *m/z*	Assignment Error (ppm)
285.275741	37.7	$C_{14}H_{33}N_6$	285.276122	1.334
287.218351	84.1	$C_{13}H_{29}N_4Na_2$	287.218212	-0.484
289.150654	72.2	$C_{11}H_{21}N_4O_5$	289.150646	-0.026
293.205156	89.8	$C_{17}H_{29}N_2S$	293.204597	-1.908
296.217846	732.4	$C_{12}H_{30}N_3O_5$	296.217997	0.508
301.136643	1425.5	$C_{14}H_{27}ClNaOS$	301.136335	-1.022
301.173140	762.8	$C_{12}H_{26}N_2NaO_5$	301.173393	0.841
302.139883	252.7	$C_{18}H_{16}N_5$	302.140022	0.460
303.034803	412.7	$C_{11}H_4N_8NaO_2$	303.034942	0.459
309.199552	784.1	$C_{17}H_{29}N_2OS$	309.199511	-0.133
313.269407	162.5	$C_{17}H_{42}ClS$	313.269027	-1.213
327.003373	1265.2	$C_{10}H_{14}ClNa_2O_5S$	327.004037	2.032
329.000351	1121.3	$C_{13}H_6ClN_6OS$	329.000684	1.013
337.099056	801.0	$C_{21}H_{18}ClO_2$	337.098984	-0.214
341.190059	283.2	$C_{25}H_{25}O$	341.189992	-0.198
348.984640	252.0	$C_{13}H_3ClN_6NaO_3$	348.984736	0.275
350.981614	203.7	$C_{18}ClN_6O$	350.981663	0.139
353.260642	408.6	$C_{22}H_{38}ClO$	353.260570	-0.203
355.064306	605.7	$C_{17}H_{17}ClN_2NaOS$	355.064233	-0.207
361.321142	1028.3	$C_{23}H_{41}N_2O$	361.321340	0.547
365.129121	465.8	$C_{15}H_{26}ClN_2O_4S$	365.129633	1.402
369.115945	399.9	$C_{19}H_{23}ClN_2NaS$	369.116268	0.875
371.095108	537.4	$C_{23}H_{16}ClN_2O$	371.094567	-1.459
371.309531	956.4	$C_{21}H_{43}N_2OS$	371.309062	-1.262
372.094645	144.1	$C_{11}H_{19}N_5NaO_6S$	372.094825	0.483
373.091730	80.0	$C_{12}H_{17}N_6O_6S$	373.092480	2.009
376.291439	87.1	$C_{17}H_{38}N_5O_4$	376.291831	1.041
379.240964	148.9	$C_{22}H_{37}Na_2S$	379.240587	-0.993
381.291218	269.1	$C_{24}H_{42}ClO$	381.291870	1.710
382.294573	63.2	$C_{21}H_{37}N_5Na$	382.294117	-1.192
387.173270	298.1	$C_{21}H_{27}N_2O_3S$	387.173690	1.085
388.176638	88.1	$C_{12}H_{35}ClNO_8S$	388.176642	0.012
389.105383	126.7	$C_{20}H_{21}O_6S$	389.105336	-0.122
391.277688	295.2	$C_{23}H_{39}N_2OS$	391.277761	0.186
394.294151	311.2	$C_{22}H_{37}N_5Na$	394.294117	-0.087
395.360633	86.5	$C_{20}H_{43}N_8$	395.360520	-0.286
401.075863	556.9	$C_{17}H_{14}ClN_6O_4$	401.075957	0.234
402.079335	126.7	$C_{23}H_{16}NO_4S$	402.079456	0.301
403.072867	196.9	$C_{16}H_{20}ClN_2O_6S$	403.072512	-0.880
403.225786	534.3	$C_{22}H_{32}ClN_4O$	403.225916	0.322
404.199400	203.2	$C_{16}H_{31}ClN_7OS$	404.199384	-0.041
406.286144	62.6	$C_{17}H_{45}ClN_3O_3S$	406.286468	0.797
408.301429	2517.3	$C_{26}H_{38}N_3O$	408.300939	-1.201
409.154391	691.8	$C_{26}H_{21}N_2O_3$	409.154669	0.680
410.157946	207.6	$C_{10}H_{29}ClN_7O_6S$	410.158307	0.879
415.029503	120.1	$C_{21}H_9N_2Na_2O_5$	415.030136	1.525
417.129589	134.6	$C_{13}H_{21}N_8O_6S$	417.129928	0.813
419.232628	471.2	$C_{18}H_{35}N_4O_5S$	419.232268	-0.858
425.128409	132.4	$C_{19}H_{23}ClN_6NaS$	425.128564	0.363

425.207363	597.2	$C_{17}H_{29}N_8O_3S$	425.207784	0.990
429.081399	1231.0	$C_{25}H_{18}ClN_2OS$	429.082288	2.073
431.077920	209.8	$C_{17}H_{22}ClN_2Na_2O_4S$	431.077871	-0.114
445.112520	4677.0	$C_{18}H_{18}ClN_8O_4$	445.113405	1.988
459.139675	1063.1	$C_{25}H_{28}ClO_4S$	459.139135	-1.176
462.138804	1739.1	$C_{17}H_{25}ClN_5O_8$	462.138617	-0.405
467.094235	756.6	$C_{23}H_{20}ClN_4O_3S$	467.093916	-0.684
471.164704	174.6	$C_{22}H_{26}ClN_6Na_2O$	471.164652	-0.111
478.312931	196.7	$C_{31}H_{44}NOS$	478.313813	1.843
483.067969	47.1	$C_{26}H_{16}ClN_4O_2S$	483.067701	-0.556
489.047721	51.5	$C_{18}H_{20}ClN_2Na_2O_7S$	489.046965	-1.546
503.099790	447.8	$C_{19}H_{24}ClN_4O_8S$	503.099789	-0.003
519.130845	1655.7	$C_{24}H_{24}N_4NaO_6S$	519.130876	0.060
525.172269	196.4	$C_{25}H_{27}N_4Na_2O_6$	525.172050	-0.417
529.170217	239.9	$C_{23}H_{26}ClN_8O_5$	529.170920	1.329
536.157209	660.1	$C_{27}H_{24}ClN_7NaO_2$	536.157221	0.022
538.154307	175.2	$C_{29}H_{24}N_5O_4S$	538.154352	0.084
541.112993	243.4	$C_{28}H_{24}KNO_8$	541.113350	0.660
579.098043	137.0	$C_{29}H_{24}ClN_2O_7S$	579.098726	1.179
581.235223	227.1	$C_{35}H_{34}N_4NaOS$	581.234554	-1.150
593.150364	328.9	$C_{31}H_{30}ClN_2O_6S$	593.150762	0.672
610.177218	262.0	$C_{31}H_{33}ClN_3O_6S$	610.177311	0.153
611.176112	69.1	$C_{35}H_{32}ClN_2O_4S$	611.176583	0.771
612.172653	63.5	$C_{34}H_{31}ClN_3O_4S$	612.171832	-1.341
615.132281	71.6	$C_{33}H_{20}N_8NaO_2S$	615.132214	-0.110
616.131850	29.0	$C_{27}H_{24}ClN_7NaO_7$	616.131795	-0.089
621.303503	45.2	$C_{35}H_{46}ClN_4O_2S$	621.302452	-1.691
624.229096	18.4	$C_{37}H_{35}N_3NaO_3S$	624.229134	0.061
637.299184	5615.3	$C_{32}H_{41}N_6O_8$	637.298039	-1.797
654.325994	2022.4	$C_{41}H_{44}N_5OS$	654.326109	0.176
659.281695	1758.0	$C_{37}H_{44}ClN_4O_3S$	659.281717	0.034
675.173588	345.8	$C_{28}H_{32}ClN_8O_8S$	675.174685	1.625
693.184713	7572.3	$C_{34}H_{31}ClN_6NaO_7$	693.183496	-1.756
711.196128	186.1	$C_{36}H_{32}ClN_6O_8$	711.196466	0.476
721.254262	144.3	$C_{37}H_{37}N_8O_6S$	721.255129	1.201
785.226723	11097.1	$C_{38}H_{38}ClN_8O_7S$	785.226721	-0.003
803.238110	483.0	$C_{38}H_{40}ClN_8O_8S$	803.237286	-1.025
			Abs mean error	0.786
			Mean std dev	0.627

Supplementary Tables of VZ185

Table S17 showing peak list, signal-to-noise ratio (S/N), elemental composition, and assignment with mass errors (ppm) of the $[M+H]^+$ VZ185 compound by CID MS/MS, calibration points are marked by an asterisk (✱).

Measured *m/z*	S/N	Elemental Composition	Theoretical *m/z*	Assignment Error (ppm)	Assignment	
249.102259	42.1	$C_{16}H_{13}N_2O$	249.102239	-0.081	C6C6C1'	
264.089330	20.4	$C_{16}H_{12}N_2O_2$	264.089329	-0.004	C6C2'	✱
267.112844	26.7	$C_{16}H_{15}N_2O_2$	267.112804	-0.150	[C6C2'+H_2]	
309.123367	5245.9	$C_{18}H_{17}N_2O_3$	309.123369	0.007	C1'	✱
313.118289	18.1	$C_{17}H_{17}N_2O_4$	313.118283	-0.020	[C53C46'-H]	
332.179064	8.6	$C_{18}H_{26}N_3OS$	332.179110	0.138	[C1C29C44+H_2]	
344.179045	9.8	$C_{19}H_{26}N_3OS$	344.179110	0.188	C16C31	
356.179107	559.9	$C_{20}H_{26}N_3OS$	356.179110	0.008	[C1C30-H_2]	
358.194756	220.8	$C_{20}H_{28}N_3OS$	358.194760	0.011	C1C30	✱
370.194664	15.2	$C_{21}H_{28}N_3OS$	370.194760	0.260	[C2C30-H_2]	
400.168944	126.2	$C_{21}H_{26}N_3O_3S$	400.168939	-0.013	C36C20	✱
451.216252	33.6	$C_{25}H_{31}N_4O_2S$	451.216224	-0.062	C6C7C42C43	
463.270402	93.5	$C_{27}H_{35}N_4O_3$	463.270367	-0.075	C24	
468.242798	166.6	$C_{25}H_{34}N_5O_2S$	468.242773	-0.053	C1C36C35	
469.226809	30.5	$C_{25}H_{33}N_4O_3S$	469.226789	-0.043	C15C36	
486.253351	1206.1	$C_{25}H_{36}N_5O_3S$	486.253338	-0.026	C1C36	✱
488.249199	52.9	$C_{25}H_{35}FN_5O_2S$	488.249001	-0.406		
488.268983	86.5	$C_{25}H_{38}N_5O_3S$	488.268988	0.010	[C1C36+H_2]	
495.265961	23.0	$C_{22}H_{36}FN_8O_2S$	495.266048	0.177		
500.269408	6.0	$C_{26}H_{38}N_5O_3S$	500.268988	-0.840	C36C2	
645.130306	5.8	$C_{40}H_{17}N_6O_4$	645.130579	0.423		
654.310823	70.4	$C_{37}H_{44}N_5O_4S$	654.310853	0.045	C29	✱
666.310982	35.1	$C_{38}H_{44}N_5O_4S$	666.310853	-0.193	[C6C6C8'-H_4]	
684.346288	50.9	$C_{40}H_{50}N_3O_5S$	684.346569	0.410		
685.354187	2521.0	$C_{35}H_{50}FN_6O_5S$	685.354195	0.012	C1	✱
699.369931	109.9	$C_{36}H_{52}FN_6O_5S$	699.369845	-0.123	[C2-H_2]	
796.385106	84.8	$C_{43}H_{54}N_7O_6S$	796.385080	-0.033	[C36+H]	✱
981.470580	18.3	$C_{52}H_{66}FN_8O_8S$	981.470287	-0.299	[M-CH_3]	
995.486136	3580.8	$C_{53}H_{68}FN_8O_8S$	995.485937	-0.200	[M+H]	
			Abs mean error	0.112		
			Mean std dev	0.176		

Table S18 showing peak list, signal-to-noise ratio (S/N), elemental composition, and assignment with mass errors (ppm) of the $[M+H]^+$ VZ185 compound by IRMPD MS/MS, calibration points are marked by an asterisk (✱).

Measured *m/z*	S/N	Elemental Composition	Theoretical *m/z*	Assignment Error (ppm)	Assignment	
112.112077	60.1	$C_7H_{14}N$	112.112076	-0.004	C17C24	✱
129.138621	46.6	$C_7H_{17}N_2$	129.138625	0.030	C2C22/C1C23	
151.122964	9.1	$C_9H_{15}N_2$	151.122975	0.070	[C1C24-H_4]	
153.138614	624.2	$C_9H_{17}N_2$	153.138625	0.069	[C1C24-H_2]	
155.154265	92.6	$C_9H_{19}N_2$	155.154275	0.062	C1C24	
161.070911	16.5	$C_9H_9N_2O$	161.070939	0.173	[C8+H_2]	
163.086577	20.1	$C_9H_{11}N_2O$	163.086589	0.077	[C8+H_4]	
180.080796	9.1	$C_{13}H_{10}N$	180.080776	-0.111	C6C11C13C4	
194.096417	31.6	$C_{14}H_{12}N$	194.096426	0.046	C6C6C17'	
200.108123	43.3	$C_{10}H_{15}FNO_2$	200.108133	0.050	C36'	✱
204.080788	7.2	$C_{15}H_{10}N$	204.080776	-0.061	C6C6C11C2'	
206.083842	28.7	$C_9H_9FN_5$	206.083650	-0.931		
208.075679	59.5	$C_{14}H_{10}NO$	208.075690	0.051	C6C6C1'C14	
210.091326	24.0	$C_{14}H_{12}NO$	210.091340	0.065	[C6C6C1'C14+H_2]	
211.086554	7.2	$C_{13}H_{11}N_2O$	211.086589	0.165	C6C4	
220.075690	40.5	$C_{15}H_{10}NO$	220.075690	0.002	C6C6C1'C12	
221.107390	7.6	$C_{15}H_{13}N_2$	221.107325	-0.293	[C6C6C11C2'+H_2]	
222.091354	46.3	$C_{15}H_{12}NO$	222.091340	-0.061	[C6C6C1'C12+H_2]	
224.106996	49.5	$C_{15}H_{14}NO$	224.106990	-0.027	[C6C6C1'C12+H_4]	
231.091678	7.4	$C_{16}H_{11}N_2$	231.091675	-0.011	C6C6C11C1'	
233.070963	21.1	$C_{15}H_9N_2O$	233.070939	-0.103	[C6C6C2'-H_2]	
234.078764	508.1	$C_{15}H_{10}N_2O$	234.078764	0.002	C6C6C2'	✱
235.086586	31.6	$C_{15}H_{11}N_2O$	235.086589	0.011	[C6C6C2'+H]	
236.070604	247.3	$C_{15}H_{10}NO_2$	236.070605	0.003	C6C12C2'	
236.094423	15.4	$C_{15}H_{12}N_2O$	236.094414	-0.039	C6C6C10C1'	
237.102231	12.2	$C_{15}H_{13}N_2O$	237.102239	0.033	[C6C6C10C1'+H]	
238.086248	81.8	$C_{15}H_{12}NO_2$	238.086255	0.029	C6C12C2'	
239.094099	41.0	$C_{10}H_{12}FN_4O_2$	239.093880	-0.918		
240.101907	11.1	$C_{15}H_{14}NO_2$	240.101905	-0.008	[C6C12C2'+H_2]	
247.086602	100.4	$C_{16}H_{11}N_2O$	247.086589	-0.053	[C6C6C1'-H_2]	
248.070604	29.9	$C_{16}H_{10}NO_2$	248.070605	0.003	[C6C1'-OH]	
249.065869	19.3	$C_{15}H_9N_2O_2$	249.065854	-0.061	[C6C7C2'-H]	
249.102240	1071.7	$C_{16}H_{13}N_2O$	249.102239	-0.003	C6C6C1'	✱
250.086236	21.8	$C_{16}H_{12}NO_2$	250.086255	0.076	[C6C12C1'-H_2]	
251.081581	9.1	$C_{15}H_{11}N_2O_2$	251.081504	-0.305	[C6C7C2'+H]	
251.117888	33.1	$C_{16}H_{15}N_2O$	251.117890	0.009	[C6C6C1'+H_2]	
252.101909	189.6	$C_{16}H_{14}NO_2$	252.101905	-0.015	C6C12C1'	
254.117578	20.2	$C_{16}H_{16}NO_2$	254.117555	-0.092	C11C12C2'	
261.102251	43.4	$C_{17}H_{13}N_2O$	261.102239	-0.044	C6C6C13C16'	
262.110101	19.5	$C_{12}H_{13}FN_5O$	262.109865	-0.899		
263.081511	246.9	$C_{16}H_{11}N_2O_2$	263.081504	-0.025	C6C1'	
264.089335	395.3	$C_{16}H_{12}N_2O_2$	264.089329	-0.022	C6C2'	✱
266.081175	63.5	$C_{16}H_{12}NO_3$	266.081170	-0.020	C13C42C2'	
267.089020	39.6	$C_{11}H_{12}FN_4O_3$	267.088795	-0.842		
267.112887	10.9	$C_{16}H_{15}N_2O_2$	267.112804	-0.310	[C6C2'+H_2]	
268.096854	34.4	$C_{16}H_{14}NO_3$	268.096820	-0.128	C2'C13	
277.097165	116.7	$C_{17}H_{13}N_2O_2$	277.097154	-0.040	[C7C1'-H_2]	
278.081172	20.3	$C_{17}H_{12}NO_3$	278.081170	-0.007	C9C1'	
278.104997	290.2	$C_{17}H_{14}N_2O_2$	278.104979	-0.065	C6C1'	
279.076436	70.5	$C_{16}H_{11}N_2O_3$	279.076419	-0.061	C2'C7C11	
279.112814	144.3	$C_{17}H_{15}N_2O_2$	279.112804	-0.035	[C6C1'+H]	
282.112476	150.0	$C_{17}H_{16}NO_3$	282.112470	-0.020	C13C2'	✱
287.121223	37.8	$C_{16}H_{19}N_2OS$	287.121261	0.133	C31C20'	

293.092071	37.0	$C_{17}H_{13}N_2O_3$	293.092069	-0.009	[C6C1'-H_2]	
294.099901	179.9	$C_{17}H_{14}N_2O_3$	294.099894	-0.024	[C2'-H]	
309.123358	14806.3	$C_{18}H_{17}N_2O_3$	309.123369	0.037	C1'	
313.155819	65.0	$C_{15}H_{22}FN_2O_4$	313.155812	-0.021	C31'	
315.152563	12.9	$C_{18}H_{23}N_2OS$	315.152561	-0.006	C17C30	∗
332.179043	7.3	$C_{18}H_{26}N_3OS$	332.179110	0.200	[C1C29C44+H_2]	
356.179134	368.6	$C_{20}H_{26}N_3OS$	356.179110	-0.068	[C1C30-H_2]	
358.194778	195.1	$C_{20}H_{28}N_3OS$	358.194760	-0.050	C1C30	∗
370.194782	13.0	$C_{21}H_{28}N_3OS$	370.194760	-0.061	[C2C30-H_2]	
400.168964	56.4	$C_{21}H_{26}N_3O_3S$	400.168939	-0.062	C36C20	
451.216386	36.6	$C_{25}H_{31}N_4O_2S$	451.216224	-0.359	C6C7C42C43	
463.270374	47.8	$C_{27}H_{35}N_4O_3$	463.270367	-0.016	C24	∗
468.242737	88.2	$C_{25}H_{34}N_5O_2S$	468.242773	0.077	C1C36C35	
469.226960	18.1	$C_{25}H_{33}N_4O_3S$	469.226789	-0.365	C15C36	
486.253408	320.1	$C_{25}H_{36}N_5O_3S$	486.253338	-0.144	C1C36	
654.310853	45.3	$C_{37}H_{44}N_5O_4S$	654.310853	-0.000	C29	∗
666.310724	22.2	$C_{38}H_{44}N_5O_4S$	666.310853	0.193	[C6C6C8'-H_4]	
685.354301	2400.9	$C_{35}H_{50}FN_6O_5S$	685.354195	-0.155	C1	
687.349918	88.5	$C_{37}H_{47}N_6O_7$	687.350074	0.227	C7C7C51C68	
699.369821	94.5	$C_{36}H_{52}FN_6O_5S$	699.369845	0.034	[C2-H_2]	∗
796.385024	56.9	$C_{43}H_{54}N_7O_6S$	796.385080	0.071	[C36+H]	∗
981.469723	6.3	$C_{52}H_{66}FN_8O_8S$	981.470287	0.574	[M-CH_3]	
995.486168	8472.1	$C_{53}H_{68}FN_8O_8S$	995.485937	-0.232	[M+H]	
			Abs mean error	0.084		
			Mean std dev	0.105		

Table S19 showing peak list, signal-to-noise ratio (S/N), elemental composition, and assignment with mass errors (ppm) of the [M+H]+ VZ185 compound by UVPD MS/MS, calibration points are marked by an asterisk (✱).

Measured *m/z*	S/N	Elemental Composition	Theoretical *m/z*	Assignment Error (ppm)	Assignment	
99.091675	62.7	$C_5H_{11}N_2$	99.091675	-0.002	C2C20/C1C21	✱
110.096433	13.6	$C_7H_{12}N$	110.096426	-0.064	[C17C24-H_2]	
111.104269	13.1	$C_7H_{13}N$	111.104251	-0.162	[C17C24-H]	
112.112083	86.9	$C_7H_{14}N$	112.112076	-0.062	C17C24	
114.127734	47.6	$C_7H_{16}N$	114.127726	-0.071	[C17C24+H_2]	
124.112094	9.9	$C_8H_{14}N$	124.112076	-0.143	[C16C24-H_2]	
125.119893	19.2	$C_8H_{15}N$	125.119901	0.062	[C16C24-H]	
126.127730	29.5	$C_8H_{16}N$	126.127726	-0.032	C16C24	
128.143379	13.5	$C_8H_{18}N$	128.143376	-0.021	[C16C24+H_2]	
129.138633	16.9	$C_7H_{17}N_2$	129.138625	-0.064	C2C22/C1C23	
134.096432	13.1	$C_9H_{12}N$	134.096426	-0.046	[C15C24-H_5]	
137.107326	10.3	$C_8H_{13}N_2$	137.107325	-0.008	[C1C46-H_4]	
139.054240	19.3	$C_{11}H_7$	139.054227	-0.095		
140.049456	11.4	$C_{10}H_6N$	140.049476	0.140		
149.107279	9.2	$C_9H_{13}N_2$	149.107325	0.306	[C1C24-H_6]	
151.054223	46.2	$C_{12}H_7$	151.054227	0.024		
151.122971	51.5	$C_9H_{15}N_2$	151.122975	0.028	[C1C24-H_4]	
152.062038	38.9	$C_{12}H_8$	152.062052	0.092		
152.130794	12.7	$C_9H_{16}N_2$	152.130800	0.041	C1C24	
153.057281	8.5	$C_{11}H_7N$	153.057301	0.131	C5C11C13C42	
153.069856	17.4	$C_{12}H_9$	153.069877	0.138		
153.138618	235.5	$C_9H_{17}N_2$	153.138625	0.048	[C1C24-H_2]	
155.154266	252.6	$C_9H_{19}N_2$	155.154275	0.061	C1C24	
161.070915	11.5	$C_9H_9N_2O$	161.070939	0.150	[C8+H_2]	
163.086578	11.8	$C_9H_{11}N_2O$	163.086589	0.066	[C8+H_4]	
164.049463	26.6	$C_{12}H_6N$	164.049476	0.082	[C5C11C13-H_4]	
165.057285	15.7	$C_{12}H_7N$	165.057301	0.099	[C5C11C13-H_3]	
165.069854	15.9	$C_{13}H_9$	165.069877	0.141		
166.065120	60.4	$C_{12}H_8N$	166.065126	0.034	[C5C11C13-H_2]	
167.072947	44.1	$C_{12}H_9N$	167.072951	0.026	[C5C11C13-H]	
168.080773	14.4	$C_{12}H_{10}N$	168.080776	0.016	C5C11C13	
169.076010	20.5	$C_{11}H_9N_2$	169.076025	0.086	C11C50	
172.113203	67.0	$C_9H_{15}FNO$	172.113219	0.094	C37'	
176.049473	10.8	$C_{13}H_6N$	176.049476	0.018		
177.057302	15.2	$C_{13}H_7N$	177.057301	-0.005	[C6C11C13C4-H_3]	
178.065119	57.8	$C_{13}H_8N$	178.065126	0.041	[C6C11C13C4-H_2]	
179.072942	50.7	$C_{13}H_9N$	179.072951	0.048	[C6C11C13C4-H]	
180.080766	101.5	$C_{13}H_{10}N$	180.080776	0.054	C6C11C13C4	
181.088597	18.3	$C_{13}H_{11}N$	181.088601	0.020		
182.059999	16.3	$C_{12}H_8NO$	182.060040	0.225	[C5-OH]	
183.055266	10.1	$C_{11}H_7N_2O$	183.055289	0.128	[C50-H_2]	
185.070919	11.2	$C_{11}H_9N_2O$	185.070939	0.106	C50	
187.050198	9.9	$C_{10}H_7N_2O_2$	187.050204	0.033		
190.065109	10.9	$C_{14}H_8N$	190.065126	0.088		
191.060364	47.2	$C_{13}H_7N_2$	191.060375	0.060	[C6C11C4-H_2]	
192.068197	69.2	$C_{13}H_8N_2$	192.068200	0.016	C6C6C11C42C3'	
193.076016	35.7	$C_{13}H_9N_2$	193.076025	0.047		
194.096410	52.4	$C_{14}H_{12}N$	194.096426	0.081	C6C6C17'	
195.067852	35.6	$C_{13}H_9NO$	195.067865	0.066		
196.075687	27.2	$C_{13}H_{10}NO$	196.075690	0.017		
197.070920	39.2	$C_{12}H_9N_2O$	197.070939	0.094	C5	

198.078765	20.4	$C_{12}H_{10}N_2O$	198.078764	-0.004		
200.108125	32.2	$C_{10}H_{15}FNO_2$	200.108133	0.042	C36'	
203.060365	22.2	$C_{14}H_7N_2$	203.060375	0.050		
204.047743	32.5	$C_{11}H_{10}NOS$	204.047762	0.094	C30C24'	
204.080770	16.4	$C_{15}H_{10}N$	204.080776	0.029	C6C6C11C2'	
205.076017	258.8	$C_{14}H_9N_2$	205.076025	0.039	C6C6C10C2'	*
206.060055	19.5	$C_{14}H_8NO$	206.060040	-0.070	C6C6C12C2'	
206.063402	50.9	$C_{11}H_{12}NOS$	206.063412	0.050	C30C24'	
206.083839	184.0	$C_{14}H_{10}N_2$	206.083850	0.053		
207.091658	69.7	$C_{14}H_{11}N_2$	207.091675	0.083	C6C6C11C3'	
208.075682	59.5	$C_{14}H_{10}NO$	208.075690	0.039	C6C6C1'C14	
208.099489	26.3	$C_{14}H_{12}N_2$	208.099500	0.052	[C6C6C11C3'+H]	
209.083475	19.1	$C_{14}H_{11}NO$	209.083515	0.189		
210.091327	41.9	$C_{14}H_{12}NO$	210.091340	0.064	[C6C6C1'C14+H_2]	
211.086574	12.2	$C_{13}H_{11}N_2O$	211.086589	0.073	C6C4	
216.047749	9.9	$C_{12}H_{10}NOS$	216.047762	0.062	C29C23'	
217.076046	12.3	$C_{15}H_9N_2$	217.076025	-0.095		
218.063425	20.9	$C_{12}H_{12}NOS$	218.063412	-0.060		
218.083808	19.4	$C_{15}H_{10}N_2$	218.083850	0.192		
219.055314	9.6	$C_{14}H_7N_2O$	219.055289	-0.114	[C6C6C3'-H_4]	
219.091659	44.2	$C_{15}H_{11}N_2$	219.091675	0.072	C6C6C11C2'	
220.063105	43.3	$C_{14}H_8N_2O$	220.063114	0.040	C6C6C42C2'	
220.075700	45.2	$C_{15}H_{10}NO$	220.075690	-0.045	C6C6C1'C12	
220.099490	26.7	$C_{15}H_{12}N_2$	220.099500	0.046		
221.070934	449.0	$C_{14}H_9N_2O$	221.070939	0.024		
221.107324	16.6	$C_{15}H_{13}N_2$	221.107325	0.004	[C6C6C11C2'+H_2]	
222.078756	25.2	$C_{14}H_{10}N_2O$	222.078764	0.036		
222.091336	59.2	$C_{15}H_{12}NO$	222.091340	0.019	[C6C6C1'C12+H_2]	
223.062802	17.3	$C_{14}H_9NO_2$	223.062780	-0.097	[C6C6C3'-H_2]	
223.086593	32.4	$C_{14}H_{11}N_2O$	223.086589	-0.016	C6C6C3'	
224.070575	8.2	$C_{14}H_{10}NO_2$	224.070605	0.135	C6C6C13C3'	
224.106986	28.3	$C_{15}H_{14}NO$	224.106990	0.018	[C6C6C1'C12+H_4]	
228.047696	7.1	$C_{13}H_{10}NOS$	228.047762	0.292	[C30C23' -H_2]	
230.063412	17.7	$C_{13}H_{12}NOS$	230.063412	-0.002	C30C23'	
231.091674	9.3	$C_{16}H_{11}N_2$	231.091675	0.006	C6C6C11C1'	
233.070940	230.4	$C_{15}H_9N_2O$	233.070939	-0.003	[C6C6C2'-H_2]	
234.078761	1194.5	$C_{15}H_{10}N_2O$	234.078764	0.014	C6C6C2'	*
235.086589	737.7	$C_{15}H_{11}N_2O$	235.086589	0.000	[C6C6C2'+H]	
236.070601	201.5	$C_{15}H_{10}NO_2$	236.070605	0.018	C6C12C2'	
236.094412	156.5	$C_{15}H_{12}N_2O$	236.094414	0.009	C6C6C10C1'	
237.102235	39.0	$C_{15}H_{13}N_2O$	237.102239	0.017	[C6C6C10C1'+H]	
238.086246	57.5	$C_{15}H_{12}NO_2$	238.086255	0.038	C6C12C2'	
240.101901	13.6	$C_{15}H_{14}NO_2$	240.101905	0.017	[C6C12C2'+H_2]	
244.079100	12.5	$C_{14}H_{14}NOS$	244.079062	-0.154	[C30C22'-H_2]	
247.086590	158.3	$C_{16}H_{11}N_2O$	247.086589	-0.006	[C6C6C1'-H_2]	
248.070603	39.0	$C_{16}H_{10}NO_2$	248.070605	0.008	[C6C1'-OH]	
248.094419	114.7	$C_{16}H_{12}N_2O$	248.094414	-0.020	[C6C6C1'-H]	
249.065860	76.9	$C_{15}H_9N_2O_2$	249.065854	-0.024	[C6C7C2'-H]	
249.102238	2127.3	$C_{16}H_{13}N_2O$	249.102239	0.006	C6C6C1'	
250.073684	21.3	$C_{15}H_{10}N_2O_2$	250.073679	-0.019	C6C7C2'	
250.086255	10.7	$C_{16}H_{12}NO_2$	250.086255	0.002	[C6C12C1'-H_2]	
251.081512	35.6	$C_{15}H_{11}N_2O_2$	251.081504	-0.033	[C6C7C2'+H]	
251.117882	60.2	$C_{16}H_{15}N_2O$	251.117890	0.033	[C6C6C1'+H_2]	
252.065538	8.4	$C_{15}H_{10}NO_3$	252.065520	-0.070	C6C6C13C2'	
252.101907	238.5	$C_{16}H_{14}NO_2$	252.101905	-0.006	C6C12C1'	
253.105262	40.6	$C_6H_{14}FN_6O_4$	253.105508	0.970		
254.117591	10.2	$C_{16}H_{16}NO_2$	254.117555	-0.142	C11C12C2'	
258.094729	25.1	$C_{15}H_{16}NOS$	258.094712	-0.064	C30C21'	
261.102236	41.5	$C_{17}H_{13}N_2O$	261.102239	0.013	C6C6C13C16'	
262.073675	24.2	$C_{16}H_{10}N_2O_2$	262.073679	0.015	[C6C7C1']	

262.110086	21.4	$C_{17}H_{14}N_2O$	262.110064	-0.083		
263.081502	265.9	$C_{16}H_{11}N_2O_2$	263.081504	0.007	C6C1'	
264.089331	1324.2	$C_{16}H_{12}N_2O_2$	264.089329	-0.006	C6C2'	*
266.081174	87.4	$C_{16}H_{12}NO_3$	266.081170	-0.015	C13C42C2'	
268.096821	45.5	$C_{16}H_{14}NO_3$	268.096820	-0.005	C2'C13	
272.110349	11.6	$C_{16}H_{18}NOS$	272.110362	0.047	[C30C20'-H_2]	
274.126003	38.2	$C_{16}H_{20}NOS$	274.126012	0.033	C30C20'	
277.097156	174.7	$C_{17}H_{13}N_2O_2$	277.097154	-0.006	[C7C1'-H_2]	
278.081187	36.8	$C_{17}H_{12}NO_3$	278.081170	-0.063	C9C1'	
278.104986	304.7	$C_{17}H_{14}N_2O_2$	278.104979	-0.026	C6C1'	*
279.076438	59.2	$C_{16}H_{11}N_2O_3$	279.076419	-0.067	C2'C7C11	
279.112809	410.2	$C_{17}H_{15}N_2O_2$	279.112804	-0.017	[C6C1'+H]	
281.128465	19.7	$C_{17}H_{17}N_2O_2$	281.128454	-0.038	C10C1'	
282.112477	255.7	$C_{17}H_{16}NO_3$	282.112470	-0.026	C13C2'	
285.105607	8.1	$C_{16}H_{17}N_2OS$	285.105611	0.013	[C31C20'-H_2]	
287.121254	44.7	$C_{16}H_{19}N_2OS$	287.121261	0.025	C31C20'	
289.136927	89.7	$C_{16}H_{21}N_2OS$	289.136911	-0.056	[C31C20' +H_2]	
290.140268	16.8	$C_{17}H_{16}N_5$	290.140022	-0.849		
293.092079	45.5	$C_{17}H_{13}N_2O_3$	293.092069	-0.032	[C6C1'-H_2]	
294.099903	277.0	$C_{17}H_{14}N_2O_3$	294.099894	-0.031	[C2'-H]	
295.107726	32.6	$C_{17}H_{15}N_2O_3$	295.107719	-0.023	C2'	
301.136918	143.0	$C_{17}H_{21}N_2OS$	301.136911	-0.025	C33C32C20'	*
307.107747	48.9	$C_{18}H_{15}N_2O_3$	307.107719	-0.090	[C1'-H_2]	
309.123359	22230.3	$C_{18}H_{17}N_2O_3$	309.123369	0.032	C1'	
313.136995	15.4	$C_{18}H_{21}N_2OS$	313.136911	-0.268	[C17C30-H_2]	
313.155834	73.3	$C_{15}H_{22}FN_2O_4$	313.155812	-0.071	C31'	
315.152573	78.5	$C_{18}H_{23}N_2OS$	315.152561	-0.036	C17C30	*
317.168250	7.4	$C_{18}H_{25}N_2OS$	317.168211	-0.125	[C17C30+H_2]	
320.139276	12.6	$C_{19}H_{18}N_3O_2$	320.139353	0.239	C11C16'	
322.118653	12.2	$C_{18}H_{16}N_3O_3$	322.118618	-0.108	[C15'-H_2]	
323.139061	7.4	$C_{19}H_{19}N_2O_3$	323.139019	-0.129	C13C16'	
324.134276	20.5	$C_{18}H_{18}N_3O_3$	324.134268	-0.025	C15'	
327.152581	57.6	$C_{19}H_{23}N_2OS$	327.152561	-0.060	C16C30	
332.179166	18.2	$C_{18}H_{26}N_3OS$	332.179110	-0.168	[C1C29C44+H_2]	
333.114260	8.8	$C_{16}H_{19}N_3O_3S$	333.114164	-0.287	C25C36	
334.155015	36.2	$C_{20}H_{20}N_3O_2$	334.155003	-0.037	C6C18'	
337.142120	15.6	$C_{19}H_{19}N_3O_3$	337.142093	-0.080	C16'	
338.149905	17.2	$C_{19}H_{20}N_3O_3$	338.149918	0.038	[C16'+H]	
344.179132	11.7	$C_{19}H_{26}N_3OS$	344.179110	-0.063	C16C31	
348.170661	26.5	$C_{21}H_{22}N_3O_2$	348.170653	-0.024	C6C19'	
351.157884	6.5	$C_{20}H_{21}N_3O_3$	351.157743	-0.401	C17'	
352.165625	21.6	$C_{20}H_{22}N_3O_3$	352.165568	-0.163	[C17'+H]	
356.179113	191.4	$C_{20}H_{26}N_3OS$	356.179110	-0.009	[C1C30-H_2]	
358.194770	112.1	$C_{20}H_{28}N_3OS$	358.194760	-0.027	C1C30	*
360.174008	11.1	$C_{19}H_{26}N_3O_2S$	360.174025	0.046		
366.181134	10.9	$C_{21}H_{24}N_3O_3$	366.181218	0.229	C18'	
370.194747	10.0	$C_{21}H_{28}N_3OS$	370.194760	0.036	[C2C30-H_2]	
374.189654	6.9	$C_{20}H_{28}N_3O_2S$	374.189675	0.056	[C16C33+H]	
393.192039	8.7	$C_{25}H_{28}FNS$	393.192101	0.159		
400.168939	103.5	$C_{21}H_{26}N_3O_3S$	400.168939	0.001	C36C20	*
402.184509	6.9	$C_{21}H_{28}N_3O_3S$	402.184589	0.199	[C36C20+H_2]	
413.200594	41.4	$C_{22}H_{29}N_4O_2S$	413.200574	-0.047	C2C33	
431.211102	48.0	$C_{22}H_{31}N_4O_3S$	431.211139	0.086	C36C18	
445.226817	66.8	$C_{23}H_{33}N_4O_3S$	445.226789	-0.063	C36C17	
451.216183	26.9	$C_{25}H_{31}N_4O_2S$	451.216224	0.090	C6C7C42C43	
457.226622	9.2	$C_{20}H_{38}FO_8S$	457.226594	-0.062		
463.270345	21.2	$C_{27}H_{35}N_4O_3$	463.270367	0.048	C24	
468.242768	46.4	$C_{25}H_{34}N_5O_2S$	468.242773	0.012	C1C36C35	
471.242629	9.3	$C_{25}H_{35}N_4O_3S$	471.242439	-0.403	[C39C22'-O]	
473.221697	14.0	$C_{24}H_{33}N_4O_4S$	473.221703	0.012	C39C23'	

485.245743	6.1	$C_{28}H_{30}FN_6O$	485.245964	0.456		
486.253330	42.7	$C_{25}H_{36}N_5O_3S$	486.253338	0.016	C1C36	∗
526.248522	9.4	$C_{27}H_{36}N_5O_4S$	526.248252	-0.514		
542.266735	8.0	$C_{33}H_{32}N_7O$	542.266285	-0.830		
630.311823	23.1	$C_{32}H_{45}FN_5O_5S$	630.311995	0.273	[C18+H]	
644.327587	70.7	$C_{33}H_{47}FN_5O_5S$	644.327645	0.090	C17	∗
652.248613	6.7	$C_{34}H_{39}FN_3O_7S$	652.248726	0.173		
656.327548	9.8	$C_{34}H_{47}FN_5O_5S$	656.327645	0.148		
658.343227	13.5	$C_{34}H_{49}FN_5O_5S$	658.343295	0.103	C16	
671.338370	7.7	$C_{34}H_{48}FN_6O_5S$	671.338544	0.260	[C1-Me]	
684.345810	6.8	$C_{46}H_{44}N_4O_2$	684.345878	0.099		
685.354081	141.8	$C_{35}H_{50}FN_6O_5S$	685.354195	0.166	C1	
699.369720	10.3	$C_{36}H_{52}FN_6O_5S$	699.369845	0.179	[C2-H_2]	
709.316433	10.6	$C_{39}H_{45}N_6O_5S$	709.316666	0.328	C33	
776.358399	6.8	$C_{50}H_{50}NO_7$	776.358179	-0.284		
782.442986	6.0	$C_{52}H_{56}N_5O_2$	782.442852	-0.171		
794.369286	67.6	$C_{43}H_{52}N_7O_6S$	794.369430	0.181	C36	
795.377398	41.8	$C_{43}H_{53}N_7O_6S$	795.377255	-0.180	[C36+H]	
796.385840	6.7	$C_{43}H_{54}N_7O_6S$	796.385080	-0.954	[C36+H_2]	
823.372185	13.3	$C_{44}H_{53}N_7O_7S$	823.372170	-0.018	C37	∗
851.390524	15.5	$C_{45}H_{55}N_8O_7S$	851.390894	0.434	C38C39	
852.393999	5.5	$C_{49}H_{53}FN_8O_3S$	852.393988	-0.013	[C6C6-Me_2][C6C6-O_3]	
864.447438	22.9	$C_{48}H_{62}N_7O_6S$	864.447680	0.279	[C40C69+H]	
994.479062	10.0	$C_{53}H_{67}FN_8O_8S$	994.478112	-0.955	[M-H]	
995.485196	13580.2	$C_{53}H_{68}FN_8O_8S$	995.485937	0.744	[M+H]	
			Abs mean error	0.082		
			Mean std dev	0.111		

Table S20 showing peak list, signal-to-noise ratio (S/N), elemental composition, and assignment with mass errors (ppm) of the $[M+Li]^+$ VZ185 compound by CID MS/MS, calibration points are marked by an asterisk (✱).

Measured *m/z*	S/N	Elemental Composition	Theoretical *m/z*	Assignment Error (ppm)	Assignment	
309.123141	108.5	$C_{18}H_{17}N_2O_3$	309.123369	0.739	[C1'+H]	
313.118045	15.4	$C_{17}H_{17}N_2O_4$	313.118283	0.760	C53C46'	
336.190270	93.5	$C_{15}H_{24}FN_3O_4Li$	336.190539	0.800	C30'	
463.270142	102783.9	$C_{27}H_{35}N_4O_3$	463.270367	0.485	C24	
469.278214	31.8	$C_{27}H_{34}N_4O_3Li$	469.278546	0.706	[C24-H]	
539.230592	1356.9	$C_{26}H_{33}FN_4O_5SLi$	539.231024	0.801	[C24'+H]	
685.353784	62.0	$C_{35}H_{50}FN_6O_5S$	685.354195	0.599	C1	
689.345055	108.3	$C_{38}H_{46}N_6O_4SLi$	689.345580	0.761	[C31+H]	
698.356754	22.2	$C_{45}H_{45}LiN_3O_4$	698.356462	-0.419		
699.329451	13.2	$C_{39}H_{44}N_6O_4SLi$	699.329930	0.686	[C33-OH]	
802.392776	267.0	$C_{43}H_{53}N_7O_6SLi$	802.393258	0.601	[C36+H_2]	
1001.494115	47587.5	$C_{53}H_{67}FN_8O_8SLi$	1001.494115	0.000	[M+Li]	✱
			Abs mean error	0.694		
			Mean std dev	0.103		

Table S21 showing peak list, signal-to-noise ratio (S/N), elemental composition, and assignment with mass errors (ppm) of the [M+Li]+ VZ185 compound by IRMPD MS/MS, calibration points are marked by an asterisk (✱).

Measured *m/z*	S/N	Elemental Composition	Theoretical *m/z*	Assignment Error (ppm)	Assignment	
112.112076	41.7	$C_7H_{14}N$	112.112076	0.000	C17C24	✱
153.138612	153.1	$C_9H_{17}N_2$	153.138625	0.083	C2C46	
194.096421	31.9	$C_{14}H_{12}N$	194.096426	0.023	[C6C6C9C56C19'-H2]	
197.070908	26.8	$C_{12}H_9N_2O$	197.070939	0.155		
200.108112	26.5	$C_{10}H_{15}FNO_2$	200.108133	0.105	[C36'+H]	
206.083838	22.8	$C_{14}H_{10}N_2$	206.083850	0.060	C6C6C11C3'	
206.096422	22.5	$C_{15}H_{12}N$	206.096426	0.018		
208.075668	70.8	$C_{14}H_{10}NO$	208.075690	0.105		
210.091327	51.3	$C_{14}H_{12}NO$	210.091340	0.062		
220.099490	21.1	$C_{15}H_{12}N_2$	220.099500	0.045	[C6C6C11C2'+H2]	
233.070934	11.7	$C_{15}H_9N_2O$	233.070939	0.022	C6C6C2'	
234.078750	798.1	$C_{15}H_{10}N_2O$	234.078764	0.062	[C6C6C2'+H]	
235.062766	15.1	$C_{15}H_9NO_2$	235.062780	0.062	C6C12C2'	
235.086578	66.1	$C_{15}H_{11}N_2O$	235.086589	0.047	C6C6C10C1'	
236.070594	969.4	$C_{15}H_{10}NO_2$	236.070605	0.048	C6C7C12C1'	
236.094410	28.1	$C_{15}H_{12}N_2O$	236.094414	0.018	[C6C6C10C1'+H]	
238.086244	294.2	$C_{15}H_{12}NO_2$	238.086255	0.044	[C6C7C12C1'+H2]	
239.094061	47.6	$C_{15}H_{13}NO_2$	239.094080	0.079	[C6C12C2'+H2]	
246.078780	11.3	$C_{16}H_{10}N_2O$	246.078764	-0.067	[C6C6C1'-H2]	
247.086585	245.2	$C_{16}H_{11}N_2O$	247.086589	0.016	[C6C6C1'-H]	
248.070602	179.5	$C_{16}H_{10}NO_2$	248.070605	0.014	[C6C13C2'-H2]	
248.094405	109.8	$C_{16}H_{12}N_2O$	248.094414	0.035	C6C6C1'	
249.065843	56.1	$C_{15}H_9N_2O_2$	249.065854	0.043	C6C7C2'	
249.102230	4394.1	$C_{16}H_{13}N_2O$	249.102239	0.034	[C6C6C1'+H]	
250.086253	149.5	$C_{16}H_{12}NO_2$	250.086255	0.010	C6C2'	
252.101900	1051.4	$C_{16}H_{14}NO_2$	252.101905	0.019	[C6C13C2'+H2]	
252.102492	149.2	$C_{14}H_{15}NOSLi$	252.102890	1.580	C30C22'	
260.094429	12.5	$C_{17}H_{12}N_2O$	260.094414	-0.056	C6C6C13C16'	
261.065932	9.6	$C_{16}H_9N_2O_2$	261.065854	-0.298	[C6C7C1']	
261.102237	193.3	$C_{17}H_{13}N_2O$	261.102239	0.008	[C6C6C13C16'+H]	
262.073680	15.5	$C_{16}H_{10}N_2O_2$	262.073679	-0.005	C6C1'	
263.081500	985.3	$C_{16}H_{11}N_2O_2$	263.081504	0.014	C6C2'	
264.089326	1747.2	$C_{11}H_{11}FN_5O_2$	264.089129	-0.747		✱
266.081164	389.8	$C_{16}H_{12}NO_3$	266.081170	0.024	C6C13C2'	
266.104981	104.7	$C_{16}H_{14}N_2O_2$	266.104979	-0.008	[C6C2'+H2]	
267.088989	150.8	$C_{16}H_{13}NO_3$	267.088995	0.023	C2'C13	
268.096821	197.8	$C_{16}H_{14}NO_3$	268.096820	-0.002	C12C2'	
276.089319	14.0	$C_{17}H_{12}N_2O_2$	276.089329	0.037	[C7C1'-H2]	
277.097151	657.6	$C_{17}H_{13}N_2O_2$	277.097154	0.009	C6C1'	
278.104980	2067.7	$C_{17}H_{14}N_2O_2$	278.104979	-0.005	[C6C1'+H]	
279.112802	611.1	$C_{17}H_{15}N_2O_2$	279.112804	0.006	C11C2'	
282.112474	1313.8	$C_{17}H_{16}NO_3$	282.112470	-0.015	C12C1'	
292.120648	31.9	$C_{13}H_{15}FN_5O_2$	292.120429	-0.748		
293.092071	205.5	$C_{17}H_{13}N_2O_3$	293.092069	-0.008	[C2'-H]	
293.092861	29.8	$C_6H_{18}FN_4O_6S$	293.092560	-1.028		
294.099902	1427.8	$C_{17}H_{14}N_2O_3$	294.099894	-0.027	C2'	
297.123383	42.6	$C_{17}H_{17}N_2O_3$	297.123369	-0.048	[C2'+H3]	
308.115581	19.6	$C_{18}H_{16}N_2O_3$	308.115544	-0.121	C1'	
309.123373	37858.4	$C_{18}H_{17}N_2O_3$	309.123369	-0.014	[C1'+H]	✱
313.118308	36.7	$C_{17}H_{17}N_2O_4$	313.118283	-0.081	C53C46'	
323.126457	37.3	$C_{18}H_{17}N_3O_3$	323.126443	-0.045	C15'	

336.190568	282.7	$C_{15}H_{24}FN_3O_4Li$	336.190539	-0.088	C30'	
340.147848	12.8	$C_{19}H_{22}N_3OS$	340.147810	-0.111	[C2C29-H_2]	
356.179149	42.4	$C_{17}H_{23}N_3O_5Li$	356.179226	0.217	C39C52	
358.174936	34.1	$C_{17}H_{22}FN_3O_4Li$	358.174889	-0.130	[C26-H_4]	
359.178328	38.6	$C_{12}H_{23}N_8O_5$	359.178592	0.736		
361.193955	19.8	$C_{12}H_{25}N_8O_5$	361.194242	0.795		
368.179145	69.8	$C_{21}H_{26}N_3OS$	368.179110	-0.095	[C15C33-H_2O]	
370.174922	8.5	$C_{18}H_{22}FN_3O_4Li$	370.174889	-0.088	[C57C43'-H_2]	
380.196888	47.7	$C_{22}H_{26}N_3O_3$	380.196868	-0.053	C13C21	
400.169011	8.4	$C_{21}H_{26}N_3O_3S$	400.168939	-0.180	[C25C40C57+H_2]	
463.270456	80063.7	$C_{27}H_{35}N_4O_3$	463.270367	-0.193	C24	
464.241301	8.1	$C_{26}H_{32}N_4O_4$	464.241807	1.090	[C42C25'-H_2]	
468.242767	13.9	$C_{25}H_{34}N_5O_2S$	468.242773	0.013	C19C32C35C39C66	
539.231127	547.6	$C_{26}H_{33}FN_4O_5SLi$	539.231024	-0.191	[C24'+H]	
684.346502	24.1	$C_{35}H_{49}FN_6O_5S$	684.346370	-0.193	[C1-H]	
685.349788	41.0	$C_{41}H_{45}N_6O_4$	685.349680	-0.158		
689.357292	18.6	$C_{41}H_{47}FLiN_2O_6$	689.357270	-0.032		
802.393253	21.1	$C_{43}H_{53}N_7O_6SLi$	802.393258	0.006	[C36+H_2]	*
1001.494536	10807.3	$C_{53}H_{67}FN_8O_8SLi$	1001.494115	-0.420	[M+Li]	
			Abs mean error	0.104		
			Mean std dev	0.247		

Table S22 showing peak list, signal-to-noise ratio (S/N), elemental composition, and assignment with mass errors (ppm) of the $[M+Li]^+$ VZ185 compound by UVPD MS/MS, calibration points are marked by an asterisk (✱).

Measured *m/z*	S/N	Elemental Composition	Theoretical *m/z*	Assignment Error (ppm)	Assignment	
102.052569	52.9	C_5H_5NOLi	102.052569	0.000	C8C56	
110.096427	72.7	$C_7H_{12}N$	110.096426	-0.006	[C17C24-H_2]	
111.104249	24.0	$C_7H_{13}N$	111.104251	0.021	[C17C24-H]	
112.112075	328.0	$C_7H_{14}N$	112.112076	0.005	C17C24	✱
117.063468	17.2	$C_5H_6N_2OLi$	117.063468	-0.004	C35C36C30'	
120.063132	54.6	$C_5H_7NO_2Li$	120.063133	0.009	C37C33'	
123.066620	33.4	C_5H_7FNOLi	123.066622	0.012	C38C37'	
124.074440	18.5	C_5H_8FNOLi	124.074447	0.057	[C38C37'+H]	
124.112079	22.1	$C_8H_{14}N$	124.112076	-0.027	[C16C24-H]	
125.107325	20.5	$C_7H_{13}N_2$	125.107325	-0.002	[C1C23+H]	
126.127730	29.5	$C_8H_{16}N$	126.127726	-0.031	C15C46	
135.074032	162.1	$C_5H_8N_2O_2Li$	135.074032	0.000	[C36C30'-H_2]	
137.089683	478.9	$C_5H_{10}N_2O_2Li$	137.089682	-0.008	C36C30'	✱
149.089683	83.1	$C_6H_{10}N_2O_2Li$	149.089682	-0.007	C36C29'	
151.054234	25.9	$C_{12}H_7$	151.054227	-0.043		
151.122981	13.3	$C_9H_{15}N_2$	151.122975	-0.042	C1C24	
152.062060	27.0	$C_{12}H_8$	152.062052	-0.054		
153.138625	683.2	$C_9H_{17}N_2$	153.138625	-0.001	C2C46	
153.138833	52.5	$C_9H_{17}N_2$	153.138625	-1.355	C2C46	
155.154276	152.7	$C_9H_{19}N_2$	155.154275	-0.007	[C1C24+H]	
158.115156	12.4	$C_9H_{13}LiNO$	158.115169	0.082		
164.049480	16.2	$C_{12}H_6N$	164.049476	-0.026	[C5C11C13-H_3]	
164.105746	17.9	$C_8H_{12}FNOLi$	164.105747	0.004	C57C37'	
165.057260	8.3	$C_{12}H_7N$	165.057301	0.251	[C5C11C13-H_2]	
166.065135	22.7	$C_{12}H_8N$	166.065126	-0.055	[C5C11C13-H]	
167.072957	22.8	$C_{12}H_9N$	167.072951	-0.037	C5C11C13	
167.154278	13.6	$C_{10}H_{19}N_2$	167.154275	-0.017	[C2C24-H]	
168.064270	18.5	$C_6H_8FLiNO_3$	168.064276	0.036		
169.076031	10.0	$C_{11}H_9N_2$	169.076025	-0.033		
172.113235	14.2	$C_9H_{15}FNO$	172.113219	-0.095		
177.057294	8.3	$C_{13}H_7N$	177.057301	0.040	[C6C11C13C4-H_2]	
178.065146	28.3	$C_{13}H_8N$	178.065126	-0.113	[C6C11C13C4-H]	
178.121402	76.6	$C_9H_{14}FNOLi$	178.121397	-0.029	C37'	
179.072955	33.9	$C_{13}H_9N$	179.072951	-0.023	C6C11C13C4	
180.080778	54.3	$C_{13}H_{10}N$	180.080776	-0.012	C6C6C11C12C3'	
188.052837	13.1	$C_{11}H_{10}NS$	188.052847	0.055	[C25C30+H]	
191.060404	24.0	$C_{13}H_7N_2$	191.060375	-0.152	C6C6C11C42C3'	
192.068204	28.6	$C_{13}H_8N_2$	192.068200	-0.022	[C4C6C11-H]	
193.076051	19.2	$C_{13}H_9N_2$	193.076025	-0.134	C4C6C11	
193.088617	7.4	$C_{14}H_{11}N$	193.088601	-0.082	C6C6C17'	
194.096432	36.4	$C_{14}H_{12}N$	194.096426	-0.031	[C6C6C9C56C19'-H_2]	
195.067869	13.3	$C_{13}H_9NO$	195.067865	-0.019	C4C6C13	
196.075675	16.0	$C_{13}H_{10}NO$	196.075690	0.076	[C4C6C13+H]	
197.070936	24.3	$C_{12}H_9N_2O$	197.070939	0.013	C5	
203.060401	10.9	$C_{14}H_7N_2$	203.060375	-0.130		
204.080755	13.3	$C_{15}H_{10}N$	204.080776	0.104		
205.076035	131.4	$C_{14}H_9N_2$	205.076025	-0.047	[C6C6C11C3'-H]	
206.083860	74.4	$C_{14}H_{10}N_2$	206.083850	-0.048	C6C6C11C3'	
206.116315	32.9	$C_{10}H_{14}FNO_2Li$	206.116312	-0.015	C36'	
207.067885	13.3	$C_{14}H_9NO$	207.067865	-0.098	C6C6C1'C14	
207.091691	28.6	$C_{14}H_{11}N_2$	207.091675	-0.076	[C6C6C11C3'+H]	
208.075687	38.0	$C_{14}H_{10}NO$	208.075690	0.016	C6C6C13C3'	

209.083522	8.3	$C_{14}H_{11}NO$	209.083515	-0.032	[C6C6C1'C14+H_2]	
210.055938	82.0	$C_{11}H_9NOSLi$	210.055940	0.007	C30C24'	
210.091358	30.6	$C_{14}H_{12}NO$	210.091340	-0.085	[C6C6C13C3'+H_2]	
216.084160	10.6	$C_{13}H_{14}NS$	216.084147	-0.059		
218.083843	8.6	$C_{15}H_{10}N_2$	218.083850	0.032	C6C6C11C2'	
218.103745	16.5	$C_{10}H_{12}FN_2O_2Li$	218.103736	-0.042	[C48-H_2]	
219.091690	19.9	$C_{15}H_{11}N_2$	219.091675	-0.070		
220.063110	19.6	$C_{14}H_8N_2O$	220.063114	0.016	[C6C6C3'-H_2]	
220.075706	25.4	$C_{15}H_{10}NO$	220.075690	-0.074	C6C6C1'C12	
220.099479	20.5	$C_{15}H_{12}N_2$	220.099500	0.095	[C6C6C11C2'+H_2]	
221.070946	162.1	$C_{14}H_9N_2O$	221.070939	-0.030		
222.078741	11.8	$C_{14}H_{10}N_2O$	222.078764	0.103	C6C6C3'	
222.091341	41.7	$C_{15}H_{12}NO$	222.091340	-0.004	[C6C6C1'C12+H_2]	
223.086596	14.6	$C_{14}H_{11}N_2O$	223.086589	-0.033	[C6C6C3'+H]	
224.059065	10.9	$C_{10}H_9FN_2O_3$	224.059172	0.480	[C40C33'-H_4]	
224.070628	11.8	$C_{14}H_{10}NO_2$	224.070605	-0.101	[C6C6C13C3'+H]	
224.106991	19.1	$C_{15}H_{14}NO$	224.106990	-0.004		
225.066838	28.3	$C_{11}H_{10}N_2OSLi$	225.066839	0.003	C31C24'	
233.070954	87.2	$C_{15}H_9N_2O$	233.070939	-0.065	C6C6C2'	
234.078767	709.0	$C_{15}H_{10}N_2O$	234.078764	-0.015	[C6C6C2'+H]	*
235.086592	258.3	$C_{15}H_{11}N_2O$	235.086589	-0.014	C6C6C10C1'	
236.070607	120.8	$C_{15}H_{10}NO_2$	236.070605	-0.009	C6C7C12C1'	
236.094430	49.6	$C_{15}H_{12}N_2O$	236.094414	-0.066	[C6C6C10C1'+H]	
236.114302	83.7	$C_{10}H_{14}FN_2O_3Li$	236.114300	-0.009	[C40C33'+H]	
238.086269	44.8	$C_{15}H_{12}NO_2$	238.086255	-0.060	[C6C7C12C1'+H_2]	
247.086590	94.7	$C_{16}H_{11}N_2O$	247.086589	-0.004	[C6C6C1'-H]	
248.070616	22.8	$C_{16}H_{10}NO_2$	248.070605	-0.046	[C6C13C2'-H_2]	
248.094418	69.4	$C_{16}H_{12}N_2O$	248.094414	-0.016	C6C6C1'	
249.065884	32.4	$C_{15}H_9N_2O_2$	249.065854	-0.118	C6C7C2'	
249.102240	1536.5	$C_{16}H_{13}N_2O$	249.102239	-0.003	[C6C6C1'+H]	
250.073661	15.9	$C_{15}H_{10}N_2O_2$	250.073679	0.073	[C6C7C2'+H]	
252.101903	149.9	$C_{16}H_{14}NO_2$	252.101905	0.007	[C6C13C2'+H_2]	
261.102236	25.1	$C_{17}H_{13}N_2O$	261.102239	0.012	[C6C6C13C16'+H]	
262.073715	8.5	$C_{16}H_{10}N_2O_2$	262.073679	-0.138	C6C1'	
263.081502	123.4	$C_{16}H_{11}N_2O_2$	263.081504	0.006	C6C2'	
266.081169	47.9	$C_{16}H_{12}NO_3$	266.081170	0.003	C6C13C2'	
266.104962	15.6	$C_{16}H_{14}N_2O_2$	266.104979	0.064	[C6C2'+H_2]	
268.096837	31.6	$C_{16}H_{14}NO_3$	268.096820	-0.063	C12C2'	
270.076067	32.3	$C_{15}H_{12}NO_4$	270.076084	0.065		
277.097152	82.8	$C_{17}H_{13}N_2O_2$	277.097154	0.006	C6C1'	
277.153436	50.4	$C_{13}H_{19}FN_2O_3Li$	277.153426	-0.036	C57C33'	
278.104979	158.9	$C_{17}H_{14}N_2O_2$	278.104979	0.001	[C6C1'+H]	
279.112803	198.3	$C_{17}H_{15}N_2O_2$	279.112804	0.004	C11C2'	
282.112461	138.6	$C_{17}H_{16}NO_3$	282.112470	0.033	C12C1'	
291.169059	32.7	$C_{14}H_{21}FN_2O_3Li$	291.169076	0.058	C33'	
293.092098	21.8	$C_{17}H_{13}N_2O_3$	293.092069	-0.100	[C2'-H]	
294.099894	134.7	$C_{17}H_{14}N_2O_3$	294.099894	0.000	C2'	
301.115908	26.2	$C_{17}H_{14}N_2O_3Li$	301.115897	-0.037	C2'	
309.123347	4797.5	$C_{18}H_{17}N_2O_3$	309.123369	0.071	[C1'+H]	*
313.118270	89.0	$C_{17}H_{17}N_2O_4$	313.118283	0.041	C53C46'	
318.179912	20.9	$C_{15}H_{22}FN_3O_3Li$	318.179975	0.197	C35C30'	
319.163996	100.8	$C_{15}H_{21}FN_2O_4Li$	319.163990	-0.019	C31'	
320.103948	22.2	$C_{16}H_{15}LiN_3O_2S$	320.103953	0.016		
321.203421	15.6	$C_{15}H_{25}FLiN_3O_3$	321.203450	0.090		
322.119609	33.9	$C_{16}H_{17}N_3O_2SLi$	322.119603	-0.018	C25C36	
323.139002	116.4	$C_{19}H_{19}N_2O_3$	323.139019	0.054	[C36C53C21'-H_4]	
334.119534	9.5	$C_{17}H_{17}N_3O_2SLi$	334.119603	0.207	C35C36C24'	
336.190517	616.5	$C_{15}H_{24}FN_3O_4Li$	336.190539	0.065	C30'	
338.114474	123.9	$C_{16}H_{17}N_3O_3SLi$	338.114517	0.127	C36C24'	
339.122316	104.6	$C_{16}H_{18}N_3O_3SLi$	339.122343	0.080	C25C36	

340.130146	12.3	$C_{16}H_{19}N_3O_3SLi$	340.130168	0.064	[C37C62+H_2]	
344.158074	33.1	$C_{19}H_{19}N_3O_3Li$	344.158096	0.064	[C16'+H]	
350.150914	71.6	$C_{18}H_{21}N_3O_2SLi$	350.150903	-0.031	C36C58C20'	
352.130132	17.0	$C_{17}H_{19}N_3O_3SLi$	352.130168	0.103	C36C46'	
356.179101	11.2	$C_{17}H_{23}N_3O_5Li$	356.179226	0.350	C39C52	
357.187011	8.7	$C_{20}H_{27}N_3OS$	357.186935	-0.212	C1C30	
394.177108	19.4	$C_{20}H_{25}N_3O_3SLi$	394.177118	0.026	C36C21'	
408.192705	16.4	$C_{21}H_{27}N_3O_3SLi$	408.192768	0.155	[C36C20+H_2]	
421.187995	29.5	$C_{21}H_{26}N_4O_3SLi$	421.188017	0.052	C19C36	
463.270213	9403.6	$C_{27}H_{35}N_4O_3$	463.270367	0.333	C24	
469.278401	16.6	$C_{27}H_{34}N_4O_3Li$	469.278546	0.309	[C24-H]	
482.160369	23.4	$C_{22}H_{24}FN_4O_5SLi$	482.160599	0.476	[C38C24'+H]	
492.261472	74.0	$C_{25}H_{35}N_5O_3SLi$	492.261516	0.090	C1C36	
521.220276	46.6	$C_{26}H_{31}FN_4O_4SLi$	521.220460	0.354	C25	
537.214552	7.5	$C_{26}H_{31}FN_4O_5SLi$	537.215374	1.530	[C24'-H]	
538.223112	85.1	$C_{26}H_{32}FN_4O_5SLi$	538.223199	0.161	C24'	
539.230854	114.2	$C_{26}H_{33}FN_4O_5SLi$	539.231024	0.316	[C24'+H]	
551.230939	71.5	$C_{27}H_{33}FN_4O_5SLi$	551.231024	0.154	C46'	
578.241310	13.4	$C_{28}H_{34}FN_5O_5SLi$	578.241923	1.060	C18C38	
607.292916	15.3	$C_{34}H_{40}N_4O_4SLi$	607.292482	-0.715	C29C45	
620.288698	68.2	$C_{31}H_{40}FN_5O_5SLi$	620.288874	0.284	[C19-H_2]	
634.304578	64.3	$C_{32}H_{42}FN_5O_5SLi$	634.304524	-0.085	[C18-H_2]	
636.320495	25.1	$C_{32}H_{44}FN_5O_5SLi$	636.320174	-0.505	[C18+H]	
648.319683	83.0	$C_{33}H_{44}FN_5O_5SLi$	648.320174	0.757	[C17-H_2]	
666.311782	7.2	$C_{35}H_{45}FN_5O_5S$	666.311995	0.320	[C15-H_4]	
677.345603	8.4	$C_{34}H_{47}FN_6O_5SLi$	677.346723	1.654	[C1-Me]	
689.345364	5.7	$C_{38}H_{46}N_6O_4SLi$	689.345580	0.313	[C31+H]	
691.362408	36.6	$C_{35}H_{49}FN_6O_5SLi$	691.362373	-0.050	[C1-H]	*
715.323585	5.9	$C_{39}H_{44}N_6O_5SLi$	715.324845	1.762	C33	
800.377179	41.0	$C_{43}H_{51}N_7O_6SLi$	800.377608	0.536	C36	
801.386319	12.8	$C_{43}H_{52}N_7O_6SLi$	801.385433	-1.106	[C36+H]	
857.398363	15.6	$C_{45}H_{54}N_8O_7SLi$	857.399072	0.827	C38C39	
858.402787	10.7	$C_{49}H_{52}FN_8O_3SLi$	858.402167	-0.723	[C6C6-Me_2][C6C6-O_3]	
870.456571	12.8	$C_{48}H_{61}N_7O_6SLi$	870.455859	-0.818	[C40C69+H]	
1001.492722	8197.0	$C_{53}H_{67}FN_8O_8SLi$	1001.494115	1.391	[M+Li]	
			Abs mean error	0.171		
			Mean std dev	0.327		

Table S23 showing peak list, signal-to-noise ratio (S/N), elemental composition, and assignment with mass errors (ppm) of the $[M+Na]^+$ VZ185 compound by CID MS/MS, calibration points are marked by an asterisk (✱).

Measured *m/z*	S/N	Elemental Composition	Theoretical *m/z*	Assignment Error (ppm)	Assignment	
309.123370	120.9	$C_{18}H_{17}N_2O_3$	309.123369	-0.004	[C1'+H]	✱
327.133935	29.2	$C_{18}H_{19}N_2O_4$	327.133934	-0.002	[C37C30'-H_4]	
335.139021	499.7	$C_{20}H_{19}N_2O_3$	335.139019	-0.007	[C13C17'-H_2]	
352.164309	161.9	$C_{15}H_{24}FN_3O_4Na$	352.164305	-0.011	C30'	
353.266218	22.5	$C_{19}H_{38}NaO_4$	353.266230	0.035		
356.179051	12.3	$C_{20}H_{26}N_3OS$	356.179110	0.166	[C15C31-H_2]	
463.270447	83278.8	$C_{27}H_{35}N_4O_3$	463.270367	-0.173	C24	
485.252190	11.3	$C_{27}H_{34}N_4O_3Na$	485.252312	0.251	[C24-H]	
486.253269	16.9	$C_{25}H_{36}N_5O_3S$	486.253338	0.142	[C1C36+H]	
509.242798	66.3	$C_{25}H_{36}N_5O_3SNa$	509.243107	0.607	[C1C36+H]	
555.204778	1740.9	$C_{26}H_{33}FN_4O_5SNa$	555.204790	0.022	[C24'+H]	✱
639.262621	8.9	$C_{31}H_{41}FN_4O_6SNa$	639.262305	-0.495	C20'	
664.294142	11.8	$C_{33}H_{44}FN_5O_5SNa$	664.293940	-0.304	[C17-H_2]	
685.354036	36.6	$C_{35}H_{50}FN_6O_5S$	685.354195	0.232	C1	
685.435644	346.9	$C_{38}H_{61}N_4O_5S$	685.435719	0.109		
705.319390	94.9	$C_{38}H_{46}N_6O_4SNa$	705.319346	-0.063	[C31+H]	
707.336308	19.4	$C_{35}H_{49}FN_6O_5SNa$	707.336139	-0.239	[C1-H]	
715.303608	12.2	$C_{39}H_{44}N_6O_4SNa$	715.303696	0.123	[C33-OH]	
818.367043	636.8	$C_{43}H_{53}N_7O_6SNa$	818.367024	-0.024	[C36+H_2]	✱
1017.468152	44054.4	$C_{53}H_{67}FN_8O_8SNa$	1017.467881	-0.266	[M+Na]	
			Abs mean error	0.168		
			Mean std dev	0.176		

Table S24 showing peak list, signal-to-noise ratio (S/N), elemental composition, and assignment with mass errors (ppm) of the [M+Na]+ VZ185 compound by IRMPD MS/MS, calibration points are marked by an asterisk (✱).

Measured *m/z*	S/N	Elemental Composition	Theoretical *m/z*	Assignment Error (ppm)	Assignment	
112.112156	19.2	$C_7H_{14}N$	112.112076	-0.714	C17C24	
153.138675	59.9	$C_9H_{17}N_2$	153.138625	-0.328	C2C46	
155.154318	26.5	$C_9H_{19}N_2$	155.154275	-0.275	[C1C24+H]	
161.070990	9.7	$C_9H_9N_2O$	161.070939	-0.319	[C8+H_2]	
175.075375	22.0	$C_{11}H_{11}O_2$	175.075356	-0.110	C64C1'	
194.096441	10.6	$C_{14}H_{12}N$	194.096426	-0.079	[C6C6C9C56C19'-H_2]	
197.070962	12.9	$C_{12}H_9N_2O$	197.070939	-0.115	C5	
200.108145	8.6	$C_{10}H_{15}FNO_2$	200.108133	-0.059	[C36'+H]	
206.083838	11.5	$C_{14}H_{10}N_2$	206.083850	0.056	C6C6C11C3'	
208.075693	28.1	$C_{14}H_{10}NO$	208.075690	-0.015	C6C6C13C3'	
210.091362	32.9	$C_{14}H_{12}NO$	210.091340	-0.105	[C6C6C13C3'+H_2]	
220.075689	37.7	$C_{15}H_{10}NO$	220.075690	0.004	C6C6C1'C12	
220.099560	9.4	$C_{15}H_{12}N_2$	220.099500	-0.272	[C6C6C11C2'+H_2]	
222.091352	48.6	$C_{15}H_{12}NO$	222.091340	-0.052	[C6C6C1'C12+H_2]	
224.106979	24.5	$C_{15}H_{14}NO$	224.106991	0.055	[C6C6C12C1'+H_4]	
234.078766	355.6	$C_{15}H_{10}N_2O$	234.078764	-0.007	[C6C6C2'+H]	✱
235.062831	9.9	$C_{15}H_9NO_2$	235.062780	-0.219	[C6C12C2'-H_2]	
235.086587	23.4	$C_{15}H_{11}N_2O$	235.086589	0.010	C6C6C10C1'	
236.070606	353.8	$C_{15}H_{10}NO_2$	236.070605	-0.005	C6C7C12C1'	
236.094380	13.0	$C_{15}H_{12}N_2O$	236.094414	0.143	[C6C6C10C1'+H]	
237.102239	19.6	$C_{15}H_{13}N_2O$	237.102239	0.002	[C6C6C2'+H_4]	
238.086255	101.6	$C_{15}H_{12}NO_2$	238.086255	-0.001	[C6C7C12C1'+H_2]	
239.094088	20.1	$C_{15}H_{13}NO_2$	239.094080	-0.036	[C6C12C2'+H_2]	
247.086584	103.6	$C_{16}H_{11}N_2O$	247.086589	0.019	[C6C6C1'-H]	
248.070612	66.9	$C_{16}H_{10}NO_2$	248.070605	-0.028	[C6C13C2'-H_2]	
248.094417	42.4	$C_{16}H_{12}N_2O$	248.094414	-0.010	C6C6C1'	
249.065843	19.3	$C_{15}H_9N_2O_2$	249.065854	0.045	C6C7C2'	
249.102237	1687.5	$C_{16}H_{13}N_2O$	249.102239	0.009	[C6C6C1'+H]	
250.086253	52.6	$C_{16}H_{12}NO_2$	250.086255	0.006	C6C13C2'	
252.101906	399.4	$C_{16}H_{14}NO_2$	252.101905	-0.005	[C6C13C2'+H_2]	
260.094486	7.3	$C_{17}H_{12}N_2O$	260.094414	-0.276	C6C6C13C16'	
261.102241	80.8	$C_{17}H_{13}N_2O$	261.102239	-0.007	[C6C6C13C16'+H]	
263.081501	368.7	$C_{16}H_{11}N_2O_2$	263.081504	0.013	[C6C2'-H]	
264.084864	65.8	$C_6H_{11}FN_7O_4$	264.085106	0.916		
264.089327	659.0	$C_{16}H_{12}N_2O_2$	264.089329	0.008	C6C2'	
266.081167	143.2	$C_{16}H_{12}NO_3$	266.081170	0.010	[C12C2'-H_2]	
266.104972	35.8	$C_{16}H_{14}N_2O_2$	266.104979	0.027	[C6C2'+H_2]	
267.088989	59.3	$C_{16}H_{13}NO_3$	267.088995	0.024	C2'C13	
268.096829	65.2	$C_{16}H_{14}NO_3$	268.096820	-0.035	C12C2'	
274.126077	7.0	$C_{16}H_{20}NOS$	274.126012	-0.236	[C30C20'+H]	
275.117884	18.4	$C_{18}H_{15}N_2O$	275.117890	0.021		
276.089324	8.1	$C_{17}H_{12}N_2O_2$	276.089329	0.019	[C6C1'-H_2]	
276.101890	16.1	$C_{18}H_{14}NO_2$	276.101905	0.054		
277.097146	235.5	$C_{17}H_{13}N_2O_2$	277.097154	0.028	C6C1'	
278.081169	58.6	$C_{17}H_{12}NO_3$	278.081170	0.003		
278.104974	693.6	$C_{17}H_{14}N_2O_2$	278.104979	0.017	[C6C1'+H]	✱
279.076414	125.7	$C_{16}H_{11}N_2O_3$	279.076419	0.018		
279.089002	41.2	$C_{12}H_{12}FN_4O_3$	279.088795	-0.743		
279.112794	317.1	$C_{17}H_{15}N_2O_2$	279.112804	0.037	C11C2'	
282.112467	439.9	$C_{17}H_{16}NO_3$	282.112470	0.011	C12C1'	
291.112798	16.1	$C_{18}H_{15}N_2O_2$	291.112804	0.021	C6C1'	
292.084245	9.6	$C_{17}H_{12}N_2O_3$	292.084244	-0.003	[C7C1'-H_2]	

292.096824	15.2	$C_{18}H_{14}NO_3$	292.096820	-0.013		
292.120608	26.1	$C_{13}H_{15}FN_5O_2$	292.120429	-0.612		
293.092064	105.0	$C_{17}H_{13}N_2O_3$	293.092069	0.017	[C2'-H_2]	
293.128445	91.0	$C_{18}H_{17}N_2O_2$	293.128454	0.032	C11C1'	
294.099884	472.8	$C_{17}H_{14}N_2O_3$	294.099894	0.035	[C2'-H]	
294.112468	226.7	$C_{18}H_{16}NO_3$	294.112470	0.006	[C13C1'-H]	
297.123410	8.7	$C_{17}H_{17}N_2O_3$	297.123369	-0.138	[C2'+H_2]	
302.104954	18.5	$C_{14}H_{13}FN_5O_2$	302.104779	-0.579		
303.112803	36.9	$C_{19}H_{15}N_2O_2$	303.112804	0.002		
304.120627	40.4	$C_{14}H_{15}FN_5O_2$	304.120429	-0.650		
306.112460	31.0	$C_{19}H_{16}NO_3$	306.112470	0.034		
307.107715	41.6	$C_{18}H_{15}N_2O_3$	307.107719	0.014	[C1'-H_2]	
307.144092	34.0	$C_{19}H_{19}N_2O_2$	307.144104	0.041		
308.128117	89.5	$C_{19}H_{18}NO_3$	308.128120	0.008		
309.123354	13207.8	$C_{18}H_{17}N_2O_3$	309.123369	0.050	[C1'+H]	
317.128458	13.1	$C_{20}H_{17}N_2O_2$	317.128454	-0.013		
319.107700	26.2	$C_{19}H_{15}N_2O_3$	319.107719	0.059	[C13C16'-H_4]	
320.115540	35.2	$C_{14}H_{15}FN_5O_3$	320.115344	-0.612		
323.126411	10.3	$C_{18}H_{17}N_3O_3$	323.126443	0.101	C15'	
323.139003	10.3	$C_{19}H_{19}N_2O_3$	323.139019	0.049	[C36C53C21'-H_4]	
327.133931	32.5	$C_{18}H_{19}N_2O_4$	327.133934	0.010	[C37C30'-H_4]	
335.139017	628.0	$C_{20}H_{19}N_2O_3$	335.139019	0.007	[C13C17'-H_2]	
352.164293	138.2	$C_{15}H_{24}FN_3O_4Na$	352.164305	0.033	C30'	
353.266223	77.1	$C_{19}H_{38}NaO_4$	353.266230	0.020		
354.163470	29.2	$C_{20}H_{24}N_3OS$	354.163460	-0.029	[C1C30-H]	
356.179109	85.4	$C_{20}H_{26}N_3OS$	356.179110	0.003	[C15C31-H_2]	
357.186927	18.2	$C_{20}H_{27}N_3OS$	357.186935	0.022	C1C30	
358.194746	26.1	$C_{20}H_{28}N_3OS$	358.194760	0.040	C15C31	
360.323646	12.9	$C_{22}H_{43}NNaO$	360.323686	0.112		
368.179198	8.1	$C_{21}H_{26}N_3OS$	368.179110	-0.238	[C15C33-H_2O]	
370.194741	26.0	$C_{21}H_{28}N_3OS$	370.194760	0.051	C2C30	
380.196764	14.4	$C_{22}H_{26}N_3O_3$	380.196868	0.273	C13C21	
381.297598	28.9	$C_{21}H_{42}NaO_4$	381.297531	-0.175		
382.194739	35.3	$C_{22}H_{28}N_3OS$	382.194760	0.054	C3C30	
400.168979	8.8	$C_{21}H_{26}N_3O_3S$	400.168939	-0.099	[C25C40C57+H_2]	
463.270376	49796.0	$C_{27}H_{35}N_4O_3$	463.270367	-0.020	C24	*
468.242654	10.8	$C_{25}H_{34}N_5O_2S$	468.242773	0.253	C19C32C35C39C66	
486.253403	11.8	$C_{25}H_{36}N_5O_3S$	486.253338	-0.134	[C1C36+H]	
509.242936	41.1	$C_{25}H_{36}N_5O_3SNa$	509.243107	0.335	[C1C36+H]	
555.204792	419.0	$C_{26}H_{33}FN_4O_5SNa$	555.204790	-0.003	[C24'+H]	*
666.310647	5.7	$C_{38}H_{44}N_5O_4S$	666.310853	0.309	C30	
684.346381	9.6	$C_{35}H_{49}FN_6O_5S$	684.346370	-0.016	[C1-H]	
685.354211	32.6	$C_{35}H_{50}FN_6O_5S$	685.354195	-0.024	C1	
685.435664	30.7	$C_{38}H_{61}N_4O_5S$	685.435719	0.080		
818.367009	59.7	$C_{43}H_{53}N_7O_6SNa$	818.367024	0.018	[C36+H_2]	*
1017.468123	21274.6	$C_{53}H_{67}FN_8O_8SNa$	1017.467881	-0.238	[M+Na]	
			Abs mean error	0.082		
			Mean std dev	0.120		

Table S25 showing peak list, signal-to-noise ratio (S/N), elemental composition, and assignment with mass errors (ppm) of the $[M+Na]^+$ VZ185 compound by UVPD MS/MS, calibration points are marked by an asterisk (✱).

Measured *m/z*	S/N	Elemental Composition	Theoretical *m/z*	Assignment Error (ppm)	Assignment
99.091639	38.1	$C_5H_{11}N_2$	99.091675	0.368	[C2C20+H]
110.096395	41.7	$C_7H_{12}N$	110.096426	0.278	[C17C24-H_2]
111.104222	19.3	$C_7H_{13}N$	111.104251	0.264	[C17C24-H]
112.112045	194.6	$C_7H_{14}N$	112.112076	0.277	C17C24
114.127695	27.0	$C_7H_{16}N$	114.127726	0.273	[C17C24+H_3]
118.026299	23.9	C_5H_5NONa	118.026335	0.307	C8C56
123.091646	8.2	$C_7H_{11}N_2$	123.091675	0.233	[C1C23-H]
124.112050	14.5	$C_8H_{14}N$	124.112076	0.209	[C16C24-H]
125.107292	12.8	$C_7H_{13}N_2$	125.107325	0.266	[C1C23+H]
125.119867	16.1	$C_8H_{15}N$	125.119901	0.276	C16C24
126.127703	28.4	$C_8H_{16}N$	126.127726	0.181	C15C46
128.143353	10.7	$C_8H_{18}N$	128.143376	0.178	
133.028375	8.7	$C_8H_5O_2$	133.028406	0.236	
134.096402	11.3	$C_9H_{12}N$	134.096426	0.176	[C15C24-H_4]
137.107279	7.8	$C_8H_{13}N_2$	137.107325	0.338	[C1C46-H_3]
138.032550	11.0	C_5H_6FNONa	138.032563	0.093	C38C37'
139.040364	46.4	C_5H_7FNONa	139.040388	0.173	[C38C37'+H]
151.047768	15.7	$C_5H_8N_2O_2Na$	151.047798	0.201	C36C30'
151.122953	44.5	$C_9H_{15}N_2$	151.122975	0.146	[C1C24-H_3]
152.062027	25.1	$C_{12}H_8$	152.062052	0.163	
152.130790	13.6	$C_9H_{16}N_2$	152.130800	0.063	[C1C24-H_2]
153.063428	45.2	$C_5H_{10}N_2O_2Na$	153.063448	0.133	[C36C30'+H_2]
153.138603	512.9	$C_9H_{17}N_2$	153.138625	0.145	C2C46
155.154253	331.4	$C_9H_{19}N_2$	155.154275	0.141	[C1C24+H]
161.070924	15.9	$C_9H_9N_2O$	161.070939	0.096	[C8+H_2]
164.049481	8.1	$C_{12}H_6N$	164.049476	-0.033	[C5C11C13-H_3]
165.063428	61.6	$C_6H_{10}N_2O_2Na$	165.063448	0.122	C36C29'
166.065101	21.4	$C_{12}H_8N$	166.065126	0.152	[C5C11C13-H]
167.072932	20.9	$C_{12}H_9N$	167.072951	0.115	C5C11C13
167.154260	13.1	$C_{10}H_{19}N_2$	167.154275	0.090	[C2C24-H]
169.076011	12.4	$C_{11}H_9N_2$	169.076025	0.082	[C11C50+H]
172.113198	43.8	$C_9H_{15}FNO$	172.113219	0.121	C37'
175.075349	23.6	$C_{11}H_{11}O_2$	175.075356	0.042	C64C1'
178.065123	23.4	$C_{13}H_8N$	178.065126	0.019	[C6C11C13C4-H]
179.072931	28.5	$C_{13}H_9N$	179.072951	0.111	C6C11C13C4
180.079492	21.8	$C_8H_{12}FNONa$	180.079513	0.118	C57C37'
180.080756	52.0	$C_{13}H_{10}N$	180.080776	0.112	C6C6C11C12C3'
182.060053	7.4	$C_{12}H_8NO$	182.060040	-0.073	C5C13
191.060366	20.0	$C_{13}H_7N_2$	191.060375	0.047	C6C6C11C42C3'
192.068179	28.0	$C_{13}H_8N_2$	192.068200	0.110	[C4C6C11-H]
193.076039	9.5	$C_{13}H_9N_2$	193.076025	-0.071	C4C6C11
193.088595	8.4	$C_{14}H_{11}N$	193.088601	0.028	C6C6C17'
194.095171	15.4	$C_9H_{14}FNONa$	194.095163	-0.041	[C37'-H]
194.096424	24.3	$C_{14}H_{12}N$	194.096426	0.010	[C6C6C9C56C19'-H_2]
195.067865	17.6	$C_{13}H_9NO$	195.067865	0.002	C4C6C13
195.102971	32.8	$C_9H_{15}FNONa$	195.102988	0.087	C37'
196.075681	12.9	$C_{13}H_{10}NO$	196.075690	0.047	[C4C6C13+H]
197.070927	20.5	$C_{12}H_9N_2O$	197.070939	0.059	C5
198.078733	10.4	$C_{12}H_{10}N_2O$	198.078764	0.155	[C5+H]
200.108117	15.5	$C_{10}H_{15}FNO_2$	200.108133	0.078	[C36'+H]
204.047747	19.5	$C_{11}H_{10}NOS$	204.047762	0.075	[C30C24'-H]
204.080764	13.7	$C_{15}H_{10}N$	204.080776	0.059	

205.076019	97.0	$C_{14}H_9N_2$	205.076025	0.029	[C6C6C11C3'-H]	
206.063402	23.1	$C_{11}H_{12}NOS$	206.063412	0.050	[C30C24'+H_3]	
206.083843	68.8	$C_{14}H_{10}N_2$	206.083850	0.032	C6C6C11C3'	
207.067849	8.2	$C_{14}H_9NO$	207.067865	0.078	C6C6C1'C14	
207.091667	24.4	$C_{14}H_{11}N_2$	207.091675	0.041	[C6C6C11C3'+H]	
208.075677	34.4	$C_{14}H_{10}NO$	208.075690	0.061	C6C6C13C3'	
209.083507	8.8	$C_{14}H_{11}NO$	209.083515	0.037	[C6C6C1'C14+H_2]	
210.091348	28.2	$C_{14}H_{12}NO$	210.091340	-0.037	[C6C6C13C3'+H_2]	
218.063385	12.4	$C_{12}H_{12}NOS$	218.063412	0.122	C30C46'	
218.083854	8.6	$C_{15}H_{10}N_2$	218.083850	-0.020	C6C6C11C2'	
219.091694	16.3	$C_{15}H_{11}N_2$	219.091675	-0.088		
220.063082	13.1	$C_{14}H_8N_2O$	220.063114	0.145	[C6C6C3'-H_2]	
220.075686	21.2	$C_{15}H_{10}NO$	220.075690	0.019	C6C6C1'C12	
220.099511	11.9	$C_{15}H_{12}N_2$	220.099500	-0.051	[C6C6C11C2'+H_2]	
221.070941	155.8	$C_{14}H_9N_2O$	221.070939	-0.007	[C6C6C3'-H]	
222.078737	8.6	$C_{14}H_{10}N_2O$	222.078764	0.122	C6C6C3'	
222.090092	10.0	$C_{10}H_{14}FNO_2Na$	222.090078	-0.065	C36'	
222.091346	31.9	$C_{15}H_{12}NO$	222.091340	-0.025	[C6C6C1'C12+H_2]	
223.062756	7.3	$C_{14}H_9NO_2$	223.062780	0.108	C6C7C13C3'	
223.086580	16.6	$C_{14}H_{11}N_2O$	223.086589	0.040	[C6C6C3'+H]	
224.070571	8.8	$C_{14}H_{10}NO_2$	224.070605	0.154	[C6C7C13C3'+H]	
224.106999	17.0	$C_{15}H_{14}NO$	224.106991	-0.036	[C6C6C12C1'+H_4]	
230.063418	9.0	$C_{13}H_{12}NOS$	230.063412	-0.027	C30C23'	
233.070940	79.9	$C_{15}H_9N_2O$	233.070939	-0.002	C6C6C2'	
234.078764	524.0	$C_{15}H_{10}N_2O$	234.078764	0.001	[C6C6C2'+H]	*
235.086591	248.4	$C_{15}H_{11}N_2O$	235.086589	-0.007	C6C6C10C1'	
236.070608	101.1	$C_{15}H_{10}NO_2$	236.070605	-0.013	C6C7C12C1'	
236.094420	50.2	$C_{15}H_{12}N_2O$	236.094414	-0.027	[C6C6C10C1'+H]	
237.102232	17.0	$C_{15}H_{13}N_2O$	237.102239	0.028	[C6C6C2'+H_4]	
238.086257	30.9	$C_{15}H_{12}NO_2$	238.086255	-0.008	[C6C7C12C1'+H_2]	
244.079118	8.1	$C_{14}H_{14}NOS$	244.079062	-0.230	[C30C22'-H]	
247.086604	76.5	$C_{16}H_{11}N_2O$	247.086589	-0.062	[C6C6C1'-H]	
248.070638	22.7	$C_{16}H_{10}NO_2$	248.070605	-0.134	[C6C13C2'-H_2]	
248.094423	56.4	$C_{16}H_{12}N_2O$	248.094414	-0.036	C6C6C1'	
249.065882	28.4	$C_{15}H_9N_2O_2$	249.065854	-0.114	C6C7C2'	
249.102245	1090.2	$C_{16}H_{13}N_2O$	249.102239	-0.022	[C6C6C1'+H]	
250.073654	14.6	$C_{15}H_{10}N_2O_2$	250.073679	0.102	[C6C7C2'+H]	
252.088073	53.2	$C_{10}H_{14}FN_2O_3Na$	252.088066	-0.026	[C40C33'+H]	
252.101915	110.9	$C_{16}H_{14}NO_2$	252.101905	-0.041	[C6C13C2'+H_2]	
261.102212	26.0	$C_{17}H_{13}N_2O$	261.102239	0.103	[C6C6C13C16'+H]	
262.073720	8.2	$C_{16}H_{10}N_2O_2$	262.073679	-0.158	[C6C2'-H_2]	
263.081517	109.5	$C_{16}H_{11}N_2O_2$	263.081504	-0.049	[C6C2'-H]	
264.089340	558.4	$C_{16}H_{12}N_2O_2$	264.089329	-0.042	C6C2'	
266.081174	43.7	$C_{16}H_{12}NO_3$	266.081170	-0.015	[C12C2'-H_2]	
266.105000	13.3	$C_{16}H_{14}N_2O_2$	266.104979	-0.078	[C6C2'+H_2]	
268.096824	25.9	$C_{16}H_{14}NO_3$	268.096820	-0.015	C12C2'	
270.043378	14.3	$C_{12}H_{11}N_2O_2SNa$	270.043345	-0.121	[C33C24'+H]	
272.110341	9.4	$C_{16}H_{18}NOS$	272.110362	0.079	[C30C20'-H]	
274.125994	10.6	$C_{16}H_{20}NOS$	274.126012	0.065	[C30C20'+H]	
277.097172	81.8	$C_{17}H_{13}N_2O_2$	277.097154	-0.064	C6C1'	
278.104991	135.6	$C_{17}H_{14}N_2O_2$	278.104979	-0.044	[C6C1'+H]	
279.112813	249.2	$C_{17}H_{15}N_2O_2$	279.112804	-0.032	C11C2'	
282.112483	115.8	$C_{17}H_{16}NO_3$	282.112470	-0.045	C12C1'	
284.091736	21.9	$C_{17}H_{13}N_2ONa$	284.092009	0.961	[C6C6C13C16'+H]	
287.121262	13.5	$C_{16}H_{19}N_2OS$	287.121261	-0.003	C19C20'	
289.136905	12.5	$C_{16}H_{21}N_2OS$	289.136911	0.022	[C31C20'+H]	
293.092103	31.5	$C_{17}H_{13}N_2O_3$	293.092069	-0.116	[C2'-H_2]	
293.127216	29.6	$C_{13}H_{19}FN_2O_3Na$	293.127191	-0.084	C57C33'	
293.128391	10.4	$C_{18}H_{17}N_2O_2$	293.128454	0.215	C11C1'	
294.099887	106.9	$C_{17}H_{14}N_2O_3$	294.099894	0.023	[C2'-H]	

294.112486	51.0	$C_{18}H_{16}NO_3$	294.112470	-0.054	[C13C1'-H]	
295.107751	31.4	$C_{17}H_{15}N_2O_3$	295.107719	-0.109	C2'	
301.136897	35.5	$C_{17}H_{21}N_2OS$	301.136911	0.045	C18C30	
303.112834	12.4	$C_{19}H_{15}N_2O_2$	303.112804	-0.098	[C6C13C17'-H_3]	
303.152671	8.3	$C_{17}H_{23}N_2OS$	303.152561	-0.363	[C18C30+H_2]	
304.120636	34.2	$C_{19}H_{16}N_2O_2$	304.120629	-0.025	[C6C13C17'-H_2]	
307.107748	36.8	$C_{18}H_{15}N_2O_3$	307.107719	-0.094	[C1'-H_2]	
307.142900	36.1	$C_{14}H_{21}FN_2O_3Na$	307.142842	-0.188	C33'	
308.128129	27.1	$C_{19}H_{18}NO_3$	308.128120	-0.031		
308.150683	14.2	$C_{14}H_{22}FN_2O_3Na$	308.150667	-0.052	[C33'+H]	
309.123371	6977.9	$C_{18}H_{17}N_2O_3$	309.123369	-0.006	[C1'+H]	*
313.155774	18.3	$C_{15}H_{22}FN_2O_4$	313.155812	0.120	C31'	
315.152574	42.1	$C_{18}H_{23}N_2OS$	315.152561	-0.042	[C17C30+H]	
319.107724	10.4	$C_{19}H_{15}N_2O_3$	319.107719	-0.016	[C13C16'-H_4]	
320.115525	23.9	$C_{19}H_{16}N_2O_3$	320.115544	0.059	[C12C17'-H_4]	
323.139028	134.6	$C_{19}H_{19}N_2O_3$	323.139019	-0.029	[C36C53C21'-H_4]	
323.157761	407.2	$C_{14}H_{24}N_2NaO_5$	323.157743	-0.057		
325.118286	58.7	$C_{18}H_{17}N_2O_4$	325.118283	-0.008	[C37C51C20'-H_6]	
327.133958	49.3	$C_{18}H_{19}N_2O_4$	327.133934	-0.073	[C37C30'-H_4]	
327.152525	11.1	$C_{19}H_{23}N_2OS$	327.152561	0.111	[C16C30-H_2]	
329.168300	12.2	$C_{19}H_{25}N_2OS$	329.168211	-0.271	C16C30	
334.155135	11.4	$C_{20}H_{20}N_3O_2$	334.155003	-0.395	C7C18'	
335.139029	967.6	$C_{20}H_{19}N_2O_3$	335.139019	-0.030	[C13C17'-H_2]	
337.141760	7.2	$C_{19}H_{19}N_3O_3$	337.142093	0.987	C16'	
338.093402	16.3	$C_{16}H_{17}N_3O_2SNa$	338.093369	-0.097	C25C36	
350.150004	8.8	$C_{20}H_{20}N_3O_3$	350.149918	-0.245	C17'	
351.152668	23.9	$C_{15}H_{24}N_2NaO_6$	351.152657	-0.030		
352.164335	266.3	$C_{15}H_{24}FN_3O_4Na$	352.164305	-0.087	C30'	
354.088351	78.8	$C_{16}H_{17}N_3O_3SNa$	354.088283	-0.192	C36C24'	
354.163393	23.6	$C_{20}H_{24}N_3OS$	354.163460	0.189	[C1C30-H]	
355.096109	43.6	$C_{16}H_{18}N_3O_3SNa$	355.096108	-0.003	[C36C24'+H]	
356.103902	10.8	$C_{16}H_{19}N_3O_3SNa$	356.103933	0.087	[C37C62+H_2]	
356.179107	62.3	$C_{20}H_{26}N_3OS$	356.179110	0.009	[C15C31-H_2]	
357.186894	15.9	$C_{20}H_{27}N_3OS$	357.186935	0.115	C1C30	
358.194832	22.7	$C_{20}H_{28}N_3OS$	358.194760	-0.202	C15C31	
360.131861	26.7	$C_{19}H_{19}N_3O_3Na$	360.131862	0.004	C16'	
366.124730	39.1	$C_{18}H_{21}N_3O_2SNa$	366.124669	-0.167	C36C58C20'	
368.103934	15.1	$C_{17}H_{19}N_3O_3SNa$	368.103933	-0.002	C36C46'	
370.194747	21.4	$C_{21}H_{28}N_3OS$	370.194760	0.035	C2C30	
372.210420	17.4	$C_{21}H_{30}N_3OS$	372.210410	-0.026		
377.059723	14.9	$C_7H_{14}N_8NaO_7S$	377.059837	0.303		
400.168905	13.4	$C_{21}H_{26}N_3O_3S$	400.168939	0.084	[C25C40C57+H_2]	
410.150940	8.5	$C_{20}H_{25}N_3O_3SNa$	410.150884	-0.137	C36C21'	
424.166527	7.1	$C_{21}H_{27}N_3O_3SNa$	424.166534	0.016	[C36C20+H_2]	
437.161828	13.0	$C_{21}H_{26}N_4O_3SNa$	437.161783	-0.102	C19C36	
445.226920	11.1	$C_{23}H_{33}N_4O_3S$	445.226789	-0.293	[C17C36+H_2]	
449.104665	8.5	$C_{26}H_{14}FN_4O_3$	449.104445	-0.489		
457.424007	7.4	$C_{27}H_{54}N_4Na$	457.424068	0.134		
463.270358	5088.0	$C_{27}H_{35}N_4O_3$	463.270367	0.020	C24	*
485.252651	6.1	$C_{27}H_{34}N_4O_3Na$	485.252312	-0.698	[C24-H]	
491.302101	9.7	$C_{18}H_{44}FN_6O_6S$	491.302159	0.117		
492.123105	10.9	$C_{29}H_{16}FN_5NaO$	492.123109	0.008		
498.133884	16.0	$C_{22}H_{24}FN_4O_5SNa$	498.134365	0.965	[C38C24'+H]	
508.235141	21.6	$C_{25}H_{35}N_5O_3SNa$	508.235282	0.277	C1C36	
537.193648	16.0	$C_{31}H_{26}FN_4O_4$	537.193260	-0.722		
553.189581	13.5	$C_{26}H_{31}FN_4O_5SNa$	553.189140	-0.796	[C24'-H]	
554.196893	44.0	$C_{26}H_{32}FN_4O_5SNa$	554.196965	0.129	C24'	
555.204798	82.6	$C_{26}H_{33}FN_4O_5SNa$	555.204790	-0.015	[C24'+H]	*
567.204831	66.1	$C_{27}H_{33}FN_4O_5SNa$	567.204790	-0.072	C46'	
594.154237	9.9	$C_{25}H_{26}FN_7NaO_6S$	594.154152	-0.143		

623.267407	17.5	$C_{31}H_{41}FN_4O_5SNa$	623.267391	-0.026	[C20'-H]
636.262800	59.7	$C_{31}H_{40}FN_5O_5SNa$	636.262640	-0.252	[C19-H_2]
644.327952	8.9	$C_{33}H_{47}FN_5O_5S$	644.327645	-0.476	[C17+H]
650.278545	22.7	$C_{32}H_{42}FN_5O_5SNa$	650.278290	-0.392	[C18-H_2]
652.293846	24.6	$C_{32}H_{44}FN_5O_5SNa$	652.293940	0.143	[C18+H]
664.293852	121.6	$C_{33}H_{44}FN_5O_5SNa$	664.293940	0.133	[C17-H_2]
666.309257	6.3	$C_{33}H_{46}FN_5O_5SNa$	666.309590	0.500	C17
668.272297	101.8	$C_{37}H_{37}N_6O_5Na$	668.271764	-0.797	[C7C7C33-H_2]
679.243914	98.8	$C_{35}H_{38}N_5O_6SNa$	679.243501	-0.608	[C37C8'-H_6]
683.338368	10.6	$C_{35}H_{48}FN_6O_5S$	683.338544	0.257	[C1-H_2]
684.347111	9.8	$C_{43}H_{45}FN_4O_3$	684.347021	-0.132	
685.354249	40.6	$C_{35}H_{50}FN_6O_5S$	685.354195	-0.079	C1
699.369771	24.3	$C_{36}H_{52}FN_6O_5S$	699.369845	0.106	[C2+H]
702.356073	7.6	$C_{38}H_{50}N_6O_5S$	702.355791	-0.402	[C6C33+H_8]
707.336099	35.5	$C_{35}H_{49}FN_6O_5SNa$	707.336139	0.057	[C1-H]
731.297504	9.2	$C_{44}H_{39}N_6O_5$	731.297645	0.192	
810.364190	30.4	$C_{43}H_{52}N_7O_7S$	810.364345	0.191	[C37-]
816.350717	30.1	$C_{43}H_{51}N_7O_6SNa$	816.351374	0.805	C36
873.373162	12.0	$C_{45}H_{54}N_8O_7SNa$	873.372838	-0.371	C38C39
886.429905	7.1	$C_{48}H_{61}N_7O_6SNa$	886.429625	-0.316	[C40C69+H]
993.470387	21.6	$C_{53}H_{66}FN_8O_8S$	993.470287	-0.101	[M-H]
1017.467218	5825.0	$C_{53}H_{67}FN_8O_8SNa$	1017.467881	0.652	[M+Na]
			Abs mean error	0.145	
			Mean std dev	0.183	

Table S26 showing peak list, signal-to-noise ratio (S/N), elemental composition, and assignment with mass errors (ppm) of the $[M+Ag]^+$ VZ185 compound by CID MS/MS, calibration points are marked by an asterisk (✱).

Measured *m/z*	S/N	Elemental Composition	Theoretical *m/z*	Assignment Error (ppm)	Assignment	
309.123370	74.4	$C_{18}H_{17}N_2O_3$	309.123369	-0.003	[C1'+H]	∗
343.171414	19.6	$C_{19}H_{25}N_3OS$	343.171285	-0.375	C16C31	
354.163439	77.0	$C_{20}H_{24}N_3OS$	354.163460	0.060	[C1C30-H]	
355.171258	32.9	$C_{20}H_{25}N_3OS$	355.171285	0.075	[C1C30-H_2]	
356.179098	11.9	$C_{20}H_{26}N_3OS$	356.179110	0.033	[C15C31-H_2]	
358.194896	10.1	$C_{20}H_{28}N_3OS$	358.194760	-0.379	C15C31	
374.149908	9.8	$C_{22}H_{20}N_3O_3$	374.149918	0.027	[C19'-H_4]	
391.176497	8.9	$C_{22}H_{23}N_4O_3$	391.176467	-0.077	[C20-H_2]	
392.184274	23.0	$C_{22}H_{24}N_4O_3$	392.184292	0.047	[C20-H]	
393.192086	27.3	$C_{22}H_{25}N_4O_3$	393.192117	0.079	C20	
395.122281	9.7	$C_{14}H_{16}FN_8O_5$	395.122220	-0.156		
396.123483	12.9	$C_{14}H_{23}FN_3O_7S$	396.123526	0.108		
397.186551	11.8	$C_{13}H_{29}N_6O_6S$	397.186380	-0.430		
400.168937	33.7	$C_{21}H_{26}N_3O_3S$	400.168939	0.004	[C25C40C57+H_2]	∗
415.186292	37.1	$C_{22}H_{27}N_2O_6$	415.186363	0.170		
441.195438	8.0	$C_{23}H_{29}N_4O_3S$	441.195488	0.113	C35C39C46'	
461.254689	210.2	$C_{27}H_{33}N_4O_3$	461.254717	0.061	[C24-H_2]	
463.270366	1840.3	$C_{27}H_{35}N_4O_3$	463.270367	0.003	C24	∗
467.211050	7.1	$C_{25}H_{31}N_4O_3S$	467.211139	0.190	[C15C36-H_4]	
470.222079	22.8	$C_{24}H_{32}N_5O_3S$	470.222038	-0.087	C19C32C39C57	
484.237676	85.3	$C_{28}H_{29}FN_6O$	484.238139	0.957	[C1C38C58-H_3O]	
485.245504	254.6	$C_{25}H_{35}N_5O_3S$	485.245513	0.018	C1C36	∗
498.253348	36.7	$C_{26}H_{36}N_5O_3S$	498.253338	-0.020	C19C39C41C57	
522.061143	22.6	$C_{21}H_{25}N_3O_4SAg$	522.061121	-0.042	C37C21'	
524.076762	51.2	$C_{21}H_{27}N_3O_4SAg$	524.076771	0.016	[C37C21'+H_2]	
569.167521	28.3	$C_{27}H_{34}N_4O_3Ag$	569.167634	0.198	[C24-H]	
591.142802	128.5	$C_{28}H_{29}FN_6OAg$	591.143231	0.725	[C1C38C58-H_3O]	∗
618.311652	8.3	$C_{31}H_{45}FN_5O_5S$	618.311995	0.555	[C19+H_2]	
639.120094	19.3	$C_{26}H_{33}FN_4O_5SAg$	639.120113	0.030	[C24'+H]	
664.295198	72.7	$C_{38}H_{42}N_5O_4S$	664.295203	0.007	C6C6C45C48'	
666.310773	64.6	$C_{38}H_{44}N_5O_4S$	666.310853	0.120	C30	
669.322807	42.0	$C_{34}H_{46}FN_6O_5S$	669.322894	0.130	[C1C57-H]	
681.322110	19.7	$C_{38}H_{45}N_6O_4S$	681.321752	-0.525	C31	
682.330709	157.8	$C_{35}H_{47}FN_6O_5S$	682.330719	0.015	[C1-H_3]	
683.338532	815.2	$C_{35}H_{48}FN_6O_5S$	683.338544	0.017	[C1-H_2]	
684.346642	48892.7	$C_{35}H_{49}FN_6O_5S$	684.346370	-0.397	[C1-H]	
686.342111	1428.7	$C_{37}H_{46}N_6O_7$	686.342249	0.200	C7C7C51C68	
697.354142	695.5	$C_{36}H_{50}FN_6O_5S$	697.354195	0.076	[C2-H]	
702.405803	26.4	$C_{37}H_{57}FN_5O_5S$	702.405896	0.133	[C3C34+H_4]	
723.177604	168.8	$C_{31}H_{41}FN_4O_6SAg$	723.177627	0.032	C20'	
771.350529	22.2	$C_{41}H_{48}FN_6O_8$	771.351217	0.892	C38C42C45C51	
777.385320	15.3	$C_{41}H_{66}AgO_7$	777.385397	0.099		
787.218895	9.6	$C_{38}H_{44}N_6O_4SAg$	787.219018	0.156	[C6C6C39C8'-H_6]	
789.234598	146.3	$C_{38}H_{46}N_6O_4SAg$	789.234668	0.088	[C31+H]	
790.243633	999.3	$C_{35}H_{48}FN_6O_5SAg$	790.243636	0.004	[C1-H_2]	∗
791.246993	357.8	$C_{41}H_{44}N_6O_4Ag$	791.246947	-0.058	[C60-OH]	
793.246674	357.4	$C_{37}H_{46}N_6O_7Ag$	793.247341	0.841	C7C7C51C68	
794.369425	965.2	$C_{43}H_{52}N_7O_6S$	794.369430	0.007	[C36+H]	
798.438318	55.9	$C_{41}H_{61}FN_7O_6S$	798.438258	-0.076		
799.219107	46.2	$C_{39}H_{44}N_6O_4SAg$	799.219018	-0.112	[C33-OH]	
900.266649	98.5	$C_{43}H_{51}N_7O_6SAg$	900.266697	0.054	C36	
902.282334	2418.0	$C_{43}H_{53}N_7O_6SAg$	902.282347	0.015	[C36+H_2]	∗

916.261549	76.5	$C_{43}H_{51}N_7O_7SAg$	916.261611	0.068	[C37-Me]
979.454588	21.5	$C_{52}H_{64}FN_8O_8S$	979.454637	0.050	C57
991.454659	660.7	$C_{53}H_{64}FN_8O_8S$	991.454637	-0.022	[M-H_3]
993.470737	28680.0	$C_{53}H_{66}FN_8O_8S$	993.470287	-0.453	[M-H]
1075.367681	23.7	$C_{51}H_{65}FN_8O_8SAg$	1075.367554	-0.118	C61
1083.372898	65.3	$C_{53}H_{65}FN_8O_7SAg$	1083.372639	-0.239	[M-H_2O]
1101.383622	31698.4	$C_{53}H_{67}FN_8O_8SAg$	1101.383204	-0.380	[M+Ag]
			Abs mean error	0.169	
			Mean std dev	0.244	

Table S27 showing peak list, signal-to-noise ratio (S/N), elemental composition, and assignment with mass errors (ppm) of the $[M+Ag]^+$ VZ185 compound by IRMPD MS/MS, calibration points are marked by an asterisk (✱).

Measured *m/z*	S/N	Elemental Composition	Theoretical *m/z*	Assignment Error (ppm)	Assignment	
151.122990	21.5	$C_9H_{15}N_2$	151.122975	-0.097	[C1C24-H_3]	
152.130818	37.1	$C_9H_{16}N_2$	152.130800	-0.119	[C1C24-H_2]	
153.138634	76.8	$C_9H_{17}N_2$	153.138625	-0.057	C2C46	
155.154291	27.8	$C_9H_{19}N_2$	155.154275	-0.100	[C1C24+H]	
163.122977	7.8	$C_{10}H_{15}N_2$	163.122975	-0.012	[C2C24-H_5]	
165.138641	22.1	$C_{10}H_{17}N_2$	165.138625	-0.099	[C2C24-H_3]	
167.154276	53.8	$C_{10}H_{19}N_2$	167.154275	-0.003	[C2C24-H]	✱
200.108136	39.0	$C_{10}H_{15}FNO_2$	200.108133	-0.013	[C36'+H]	
204.047775	9.1	$C_{11}H_{10}NOS$	204.047762	-0.063	[C30C24'-H]	
206.063414	22.2	$C_{11}H_{12}NOS$	206.063412	-0.008	[C30C24'+H_3]	
219.058660	7.7	$C_{11}H_{11}N_2OS$	219.058661	0.005	[C31C24'+H]	
222.091325	7.4	$C_{15}H_{12}NO$	222.091340	0.068	[C6C6C1'C12+H_2]	
234.078760	39.7	$C_{15}H_{10}N_2O$	234.078764	0.015	[C6C6C2'+H]	
236.070595	71.5	$C_{15}H_{10}NO_2$	236.070605	0.040	C6C7C12C1'	
238.086250	17.6	$C_{15}H_{12}NO_2$	238.086255	0.022	[C6C7C12C1'+H_2]	
247.086606	7.0	$C_{16}H_{11}N_2O$	247.086589	-0.068	[C6C6C1'-H]	
248.070599	12.6	$C_{16}H_{10}NO_2$	248.070605	0.024	[C6C13C2'-H_2]	
249.102226	338.9	$C_{16}H_{13}N_2O$	249.102239	0.051	[C6C6C1'+H]	
250.086296	8.7	$C_{16}H_{12}NO_2$	250.086255	-0.165	C6C13C2'	
252.101910	86.2	$C_{16}H_{14}NO_2$	252.101905	-0.018	[C6C13C2'+H_2]	
259.035903	15.9	$C_9H_{16}N_2Ag$	259.035892	-0.042	[C1C24-H_2]	
260.110355	15.6	$C_{15}H_{18}NOS$	260.110362	0.025	[C30C21'+H]	
261.035523	18.6	$C_8H_9N_2O_8$	261.035342	-0.692		
261.102249	9.2	$C_{17}H_{13}N_2O$	261.102239	-0.040	[C6C6C13C16'+H]	
263.081508	63.2	$C_{16}H_{11}N_2O_2$	263.081504	-0.016	[C6C2'-H]	
264.089318	131.1	$C_{16}H_{12}N_2O_2$	264.089329	0.041	C6C2'	
266.081164	38.3	$C_{16}H_{12}NO_3$	266.081170	0.024	[C12C2'-H_2]	
267.088998	7.3	$C_{16}H_{13}NO_3$	267.088995	-0.011	C2'C13	
268.096805	20.7	$C_{16}H_{14}NO_3$	268.096820	0.056	C12C2'	
270.094685	7.5	$C_{16}H_{16}NOS$	270.094712	0.098	[C30C20'-H_3]	
272.110340	10.3	$C_{16}H_{18}NOS$	272.110362	0.082	[C30C20'-H]	
274.125996	44.9	$C_{16}H_{20}NOS$	274.126012	0.059	[C30C20'+H]	
277.097136	51.0	$C_{17}H_{13}N_2O_2$	277.097154	0.065	C6C1'	
278.104969	176.0	$C_{17}H_{14}N_2O_2$	278.104979	0.037	[C6C1'+H]	
279.112791	52.1	$C_{17}H_{15}N_2O_2$	279.112804	0.046	C11C2'	
282.112456	123.8	$C_{17}H_{16}NO_3$	282.112470	0.050	C12C1'	
287.121247	383.6	$C_{16}H_{19}N_2OS$	287.121261	0.048	C19C20'	
289.136908	57.0	$C_{16}H_{21}N_2OS$	289.136911	0.012	[C31C20'+H]	
293.092070	15.8	$C_{17}H_{13}N_2O_3$	293.092069	-0.002	[C2'-H_2]	
294.099876	131.2	$C_{17}H_{14}N_2O_3$	294.099894	0.061	[C2'-H]	
295.107684	13.2	$C_{17}H_{15}N_2O_3$	295.107719	0.118	C2'	
297.123350	35.4	$C_{17}H_{17}N_2O_3$	297.123369	0.064	[C2'+H_2]	
301.136898	27.3	$C_{17}H_{21}N_2OS$	301.136911	0.043	C18C30	
309.123356	5703.7	$C_{18}H_{17}N_2O_3$	309.123369	0.042	[C1'+H]	✱
313.155803	55.8	$C_{15}H_{22}FN_2O_4$	313.155812	0.028	C31'	
314.144719	10.4	$C_{18}H_{22}N_2OS$	314.144736	0.054	C17C30	
315.152572	43.2	$C_{18}H_{23}N_2OS$	315.152561	-0.034	[C17C30+H]	
327.152580	11.5	$C_{19}H_{23}N_2OS$	327.152561	-0.057	[C16C30-H_2]	
328.147839	6.9	$C_{18}H_{22}N_3OS$	328.147810	-0.088	[C17C31-H_2]	
332.179123	27.8	$C_{18}H_{26}N_3OS$	332.179110	-0.040	C1C26'	
335.138958	7.2	$C_{20}H_{19}N_2O_3$	335.139019	0.182	[C13C17'-H_2]	
339.152491	16.6	$C_{20}H_{23}N_2OS$	339.152561	0.206	[C15C30-H_4]	
340.147802	189.6	$C_{19}H_{22}N_3OS$	340.147810	0.024	[C1C29-H_2]	

342.173565	93.2	$C_{17}H_{28}NO_4S$	342.173356	-0.610		
351.152578	19.7	$C_{21}H_{23}N_2OS$	351.152561	-0.048		
353.168188	38.7	$C_{21}H_{25}N_2OS$	353.168211	0.064		
354.163459	684.0	$C_{20}H_{24}N_3OS$	354.163460	0.002	[C1C30-H]	
355.171283	363.2	$C_{20}H_{25}N_3OS$	355.171285	0.005	[C1C30-H_2]	
356.179111	457.8	$C_{20}H_{26}N_3OS$	356.179110	-0.003	[C15C31-H_2]	
357.186942	59.8	$C_{20}H_{27}N_3OS$	357.186935	-0.020	C1C30	
358.194749	147.7	$C_{20}H_{28}N_3OS$	358.194760	0.032	C15C31	
368.179112	513.8	$C_{21}H_{26}N_3OS$	368.179110	-0.007	[C15C33-H_2O]	
370.194759	214.2	$C_{21}H_{28}N_3OS$	370.194760	0.002	C2C30	
372.197820	146.0	$C_{20}H_{28}N_4OS$	372.197834	0.037	C1C31	
374.149926	23.5	$C_{22}H_{20}N_3O_3$	374.149918	-0.022	[C19'-H_4]	
382.158366	15.6	$C_{21}H_{24}N_3O_2S$	382.158375	0.024	[C15C33-H_4]	
382.182195	11.1	$C_{21}H_{26}N_4OS$	382.182184	-0.028	C1C33C32	
385.205624	22.1	$C_{21}H_{29}N_4OS$	385.205659	0.092	C2C31	
391.176411	22.8	$C_{22}H_{23}N_4O_3$	391.176467	0.144	[C20-H_2]	
392.184238	20.9	$C_{22}H_{24}N_4O_3$	392.184292	0.138	[C20-H]	
393.192117	57.2	$C_{22}H_{25}N_4O_3$	393.192117	-0.001	C20	
395.034349	7.0	$C_{16}H_{20}N_2OSAg$	395.034177	-0.436	C31C20'	
395.207786	9.2	$C_{22}H_{27}N_4O_3$	395.207767	-0.049	[C20+H_2]	
399.184918	62.2	$C_{21}H_{27}N_4O_2S$	399.184924	0.015	C1C33	
400.168946	531.7	$C_{21}H_{26}N_3O_3S$	400.168939	-0.018	[C25C40C57+H_2]	*
406.158327	15.6	$C_{23}H_{24}N_3O_2S$	406.158375	0.118	[C32C40C41C46'-H_2]	
409.013374	14.7	$C_{16}H_{18}AgN_2O_2S$	409.013442	0.167	C33C21'	
411.013088	12.3	$C_{13}H_{13}AgFN_6O_2$	411.012945	-0.349		
411.184856	10.1	$C_{22}H_{27}N_4O_2S$	411.184924	0.166		
415.186381	50.1	$C_{22}H_{27}N_2O_6$	415.186363	-0.044		
419.211198	56.4	$C_{21}H_{31}N_4O_3S$	419.211139	-0.140	C39C62	
425.200611	29.3	$C_{23}H_{29}N_4O_2S$	425.200574	-0.086	[C17C32C36-H_2]	
429.195438	13.8	$C_{22}H_{29}N_4O_3S$	429.195488	0.116	[C25C39+H]	
437.200672	13.3	$C_{24}H_{29}N_4O_2S$	437.200574	-0.224	C32C35C41C63C24'	
440.019212	14.8	$C_{16}H_{19}N_3O_3SAg$	440.019256	0.099	[C37C62+H_2]	
441.195506	67.7	$C_{23}H_{29}N_4O_3S$	441.195488	-0.040	C35C39C46'	
449.200604	43.2	$C_{25}H_{29}N_4O_2S$	449.200574	-0.068	C25C32C41C57C65	
450.203935	11.7	$C_{26}H_{24}N_7O$	450.203685	-0.556		
451.216223	58.0	$C_{25}H_{31}N_4O_2S$	451.216224	0.002		
452.019232	9.8	$C_{17}H_{19}N_3O_3SAg$	452.019256	0.052	C36C46'	
455.211157	97.9	$C_{24}H_{31}N_4O_3S$	455.211139	-0.038	C35C39C23'	
459.239098	22.7	$C_{27}H_{31}N_4O_3$	459.239067	-0.067	[C24-H_4]	
461.254733	1386.4	$C_{27}H_{33}N_4O_3$	461.254717	-0.036	[C24-H_2]	
463.084238	24.6	$C_{20}H_{26}N_3OSAg$	463.084202	-0.078	[C15C31-H_2]	
463.270378	15838.7	$C_{27}H_{35}N_4O_3$	463.270367	-0.025	C24	*
465.219295	93.7	$C_{25}H_{31}N_5O_2S$	465.219298	0.006	[C1C35C36-H_2]	
467.211144	36.3	$C_{25}H_{31}N_4O_3S$	467.211139	-0.011	[C15C36-H_4]	
467.230407	18.3	$C_{26}H_{27}N_8O$	467.230234	-0.370		
467.234923	17.8	$C_{25}H_{33}N_5O_2S$	467.234948	0.054	C1C35C36	
467.265323	20.0	$C_{26}H_{35}N_4O_4$	467.265282	-0.087		
468.242773	118.7	$C_{25}H_{34}N_5O_2S$	468.242773	-0.000	C19C32C35C39C66	
470.222058	310.4	$C_{24}H_{32}N_5O_3S$	470.222038	-0.042	C19C32C39C57	
471.202736	42.6	$C_{27}H_{27}N_4O_4$	471.202682	-0.115		
476.079469	105.1	$C_{15}H_{24}AgFN_7OS$	476.079251	-0.457		
477.249663	20.3	$C_{27}H_{33}N_4O_4$	477.249632	-0.065	C35C65C67C23'	
478.095113	196.9	$C_{15}H_{26}AgFN_7OS$	478.094901	-0.442		
480.242790	189.7	$C_{26}H_{34}N_5O_2S$	480.242773	-0.036	C19C35C39C57	

483.229885	41.2	$C_{25}H_{33}N_5O_3S$	483.229863	-0.046	C19C39C41C57C57	
484.237722	189.4	$C_{28}H_{29}FN_6O$	484.238139	0.862	[C1C38C58-H_3O]	
485.245526	3305.9	$C_{25}H_{35}N_5O_3S$	485.245513	-0.026	C1C36	*
498.253362	245.3	$C_{26}H_{36}N_5O_3S$	498.253338	-0.049	C19C39C41C57	
505.081994	13.3	$C_{21}H_{26}N_4O_2SAg$	505.082190	0.389	[C1C33-H]	
508.081619	7.7	$C_{21}H_{27}N_3O_3SAg$	508.081856	0.467	[C36C20+H_2]	
522.060925	9.0	$C_{21}H_{25}N_3O_4SAg$	522.061121	0.375	C37C21'	
524.076796	34.1	$C_{21}H_{27}N_3O_4SAg$	524.076771	-0.048	[C37C21'+H_2]	
569.167661	45.6	$C_{27}H_{34}N_4O_3Ag$	569.167634	-0.047	[C24-H]	
589.127204	39.7	$C_{20}H_{31}AgFN_8O_3S$	589.126929	-0.467		
591.142798	1970.4	$C_{28}H_{29}FN_6OAg$	591.143231	0.732	[C1C38C58-H_3O]	*
618.312085	38.5	$C_{31}H_{45}FN_5O_5S$	618.311995	-0.146	[C19+H_2]	
634.284598	11.6	$C_{37}H_{40}N_5O_3S$	634.284638	0.064	C6C30	
639.120327	16.0	$C_{26}H_{33}FN_4O_5SAg$	639.120113	-0.335	[C24'+H]	
641.119757	16.5	$C_{29}H_{21}N_8O_8S$	641.119757	-0.001		
662.279627	14.2	$C_{38}H_{40}N_5O_4S$	662.279552	-0.114	[C30-H_4]	
664.295235	131.7	$C_{38}H_{42}N_5O_4S$	664.295203	-0.049	C6C6C45C48'	
666.310865	241.4	$C_{38}H_{44}N_5O_4S$	666.310853	-0.018	C30	
669.322936	57.9	$C_{34}H_{46}FN_6O_5S$	669.322894	-0.063	[C1C57-H]	
680.314040	10.6	$C_{38}H_{44}N_6O_4S$	680.313927	-0.166	[C6C6C39C8'-H_6]	
681.322288	26.1	$C_{38}H_{45}N_6O_4S$	681.321752	-0.786	C31	
682.330676	64.2	$C_{35}H_{47}FN_6O_5S$	682.330719	0.063	[C1-H_3]	
683.338563	245.0	$C_{35}H_{48}FN_6O_5S$	683.338544	-0.027	[C1-H_2]	
684.346377	8954.8	$C_{35}H_{49}FN_6O_5S$	684.346370	-0.010	[C1-H]	*
686.342112	358.1	$C_{37}H_{46}N_6O_7$	686.342249	0.199	C7C7C51C68	
697.354205	505.9	$C_{36}H_{50}FN_6O_5S$	697.354195	-0.014	[C2-H]	
702.405860	15.9	$C_{37}H_{57}FN_5O_5S$	702.405896	0.051	[C3C34+H_4]	
708.285295	14.9	$C_{39}H_{42}N_5O_6S$	708.285032	-0.372		
723.177522	60.8	$C_{31}H_{41}FN_4O_6SAg$	723.177627	0.146	C20'	
759.332282	20.7	$C_{43}H_{47}N_6O_5S$	759.332316	0.045	C6C13C35C37	
772.233114	40.9	$C_{40}H_{47}AgN_3O_4S$	772.233271	0.203		
776.358820	112.9	$C_{43}H_{50}N_7O_5S$	776.358865	0.057	C35C36	
787.218867	76.8	$C_{38}H_{44}N_6O_4SAg$	787.219018	0.192	[C6C6C39C8'-H_6]	
789.234641	368.6	$C_{38}H_{46}N_6O_4SAg$	789.234668	0.034	[C31+H]	
790.243698	530.6	$C_{35}H_{48}FN_6O_5SAg$	790.243636	-0.078	[C1-H_2]	
791.246868	189.3	$C_{41}H_{44}N_6O_4Ag$	791.246947	0.100	[C60-OH]	
793.246751	183.5	$C_{37}H_{46}N_6O_7Ag$	793.247341	0.743	C7C7C51C68	
793.361774	46.0	$C_{43}H_{51}N_7O_6S$	793.361605	-0.213	C36	
794.369422	1521.8	$C_{43}H_{52}N_7O_6S$	794.369430	0.010	[C36+H]	*
799.218971	44.9	$C_{39}H_{44}N_6O_4SAg$	799.219018	0.059	[C33-OH]	
900.266668	214.7	$C_{43}H_{51}N_7O_6SAg$	900.266697	0.032	C36	
902.282273	3697.0	$C_{43}H_{53}N_7O_6SAg$	902.282347	0.082	[C36+H_2]	*
916.261273	25.2	$C_{43}H_{51}N_7O_7SAg$	916.261611	0.369	[C37-Me]	
979.454582	6.7	$C_{52}H_{64}FN_8O_8S$	979.454637	0.056	C57	
991.454396	88.8	$C_{53}H_{64}FN_8O_8S$	991.454637	0.243	[M-H_3]	
993.470210	2840.9	$C_{53}H_{66}FN_8O_8S$	993.470287	0.078	[M-H]	
1083.372634	63.8	$C_{53}H_{65}FN_8O_7SAg$	1083.372639	0.005	[M-H_2O]	
1101.383186	13619.0	$C_{53}H_{67}FN_8O_8SAg$	1101.383204	0.017	[M+Ag]	
			Abs mean error	0.097		
			Mean std dev	0.149		

Table S28 showing peak list, signal-to-noise ratio (S/N), elemental composition, and assignment with mass errors (ppm) of the $[M+Ag]^+$ VZ185 compound by UVPD MS/MS, calibration points are marked by an asterisk (✱).

Measured *m/z*	S/N	Elemental Composition	Theoretical *m/z*	Assignment Error (ppm)	Assignment
99.091672	27.2	$C_5H_{11}N_2$	99.091675	0.027	[C2C20+H]
100.112076	10.4	$C_6H_{14}N$	100.112076	0.001	[C18C21+H_2]
104.050618	41.2	C_4H_7FNO	104.050618	0.003	[C40'+H_2]
106.065110	11.4	C_7H_8N	106.065126	0.149	[C17C24-H_6]
108.080774	28.1	$C_7H_{10}N$	108.080776	0.019	[C17C24-H_4]
109.076021	20.9	$C_6H_9N_2$	109.076025	0.036	[C2C21-H_2]
109.088599	14.2	$C_7H_{11}N$	109.088601	0.020	[C17C24-H_3]
110.096427	116.2	$C_7H_{12}N$	110.096426	-0.007	[C17C24-H_2]
111.104249	124.4	$C_7H_{13}N$	111.104251	0.019	[C17C24-H]
112.112075	329.5	$C_7H_{14}N$	112.112076	0.009	C17C24
113.119909	7.7	$C_7H_{15}N$	113.119901	-0.067	[C17C24+H_2]
114.127724	191.5	$C_7H_{16}N$	114.127726	0.015	[C17C24+H_3]
116.050620	37.4	C_5H_7FNO	116.050618	-0.019	[C38C37'+H]
120.080772	14.1	$C_8H_{10}N$	120.080776	0.029	
121.076027	13.7	$C_7H_9N_2$	121.076025	-0.015	[C1C23-H_3]
122.096421	39.8	$C_8H_{12}N$	122.096426	0.044	
123.091671	52.8	$C_7H_{11}N_2$	123.091675	0.030	[C1C23-H]
123.104249	8.4	$C_8H_{13}N$	123.104251	0.019	[C16C24-H_2]
124.099494	18.2	$C_7H_{12}N_2$	124.099500	0.048	C1C23
124.112075	68.6	$C_8H_{14}N$	124.112076	0.006	[C16C24-H]
125.107322	39.5	$C_7H_{13}N_2$	125.107325	0.020	[C1C23+H]
125.119897	235.5	$C_8H_{15}N$	125.119901	0.033	C16C24
126.127722	110.5	$C_8H_{16}N$	126.127726	0.029	C15C46
128.143371	52.8	$C_8H_{18}N$	128.143376	0.042	
131.081503	31.6	$C_5H_{11}N_2O_2$	131.081504	0.005	[C36C30'+H_3]
134.096425	69.2	$C_9H_{12}N$	134.096426	0.005	[C15C24-H_4]
135.091672	21.3	$C_8H_{11}N_2$	135.091675	0.025	[C1C46-H_5]
135.104250	12.4	$C_9H_{13}N$	135.104251	0.008	[C15C24-H_3]
136.112073	53.8	$C_9H_{14}N$	136.112076	0.020	[C15C24-H_2]
137.107322	87.1	$C_8H_{13}N_2$	137.107325	0.026	[C1C46-H_3]
138.115150	15.4	$C_8H_{14}N_2$	138.115150	0.003	[C1C46-H_2]
139.054221	18.4	$C_{11}H_7$	139.054227	0.044	
139.122972	48.1	$C_8H_{15}N_2$	139.122975	0.023	[C2C23-H_2]
139.135551	19.6	$C_9H_{17}N$	139.135551	0.000	[C15C24+H]
140.143367	17.3	$C_9H_{18}N$	140.143376	0.068	
141.138621	14.1	$C_8H_{17}N_2$	141.138625	0.026	C2C23
142.159018	12.4	$C_9H_{20}N$	142.159026	0.053	
143.081501	15.0	$C_6H_{11}N_2O_2$	143.081504	0.018	[C36C29'+H]
148.099500	9.6	$C_9H_{12}N_2$	148.099500	-0.001	[C1C24-H_6]
148.112076	33.1	$C_{10}H_{14}N$	148.112076	-0.003	
149.107322	86.6	$C_9H_{13}N_2$	149.107325	0.019	[C1C24-H_5]
150.115143	35.4	$C_9H_{14}N_2$	150.115150	0.047	[C1C24-H_4]
151.122972	330.9	$C_9H_{15}N_2$	151.122975	0.022	[C1C24-H_3]
152.130794	155.6	$C_9H_{16}N_2$	152.130800	0.037	[C1C24-H_2]
153.138620	606.1	$C_9H_{17}N_2$	153.138625	0.033	C2C46
154.146452	8.7	$C_9H_{18}N_2$	154.146450	-0.013	C1C24
155.154271	113.5	$C_9H_{19}N_2$	155.154275	0.023	[C1C24+H]
163.122973	50.7	$C_{10}H_{15}N_2$	163.122975	0.014	[C2C24-H_5]
164.049458	14.9	$C_{12}H_6N$	164.049476	0.108	[C5C11C13-H_3]
165.057301	14.5	$C_{12}H_7N$	165.057301	-0.001	[C5C11C13-H_2]
165.138616	80.3	$C_{10}H_{17}N_2$	165.138625	0.055	[C2C24-H_3]
166.065123	41.0	$C_{12}H_8N$	166.065126	0.017	[C5C11C13-H]
167.072940	36.9	$C_{12}H_9N$	167.072951	0.066	C5C11C13

167.154275	60.2	$C_{10}H_{19}N_2$	167.154275	-0.002	[C2C24-H]	*
169.076011	16.6	$C_{11}H_9N_2$	169.076025	0.080	[C11C50+H]	
172.113214	270.9	$C_9H_{15}FNO$	172.113219	0.031	C37'	
177.057320	12.4	$C_{13}H_7N$	177.057301	-0.106	[C6C11C13C4-H_2]	
178.065122	49.0	$C_{13}H_8N$	178.065126	0.023	[C6C11C13C4-H]	
179.072948	39.5	$C_{13}H_9N$	179.072951	0.015	C6C11C13C4	
180.080775	74.4	$C_{13}H_{10}N$	180.080776	0.006	C6C6C11C12C3'	
182.060077	14.3	$C_{12}H_8NO$	182.060040	-0.203	C5C13	
183.055286	8.2	$C_{11}H_7N_2O$	183.055289	0.018	[C50-H]	
185.070926	12.2	$C_{11}H_9N_2O$	185.070939	0.070		
187.050203	7.8	$C_{10}H_7N_2O_2$	187.050204	0.005	C33C44C67C24'	
188.052842	16.2	$C_{11}H_{10}NS$	188.052847	0.027	[C25C30+H]	
189.060683	9.7	$C_{11}H_{11}NS$	189.060672	-0.059	[C25C30+H_2]	
190.065094	10.5	$C_{14}H_8N$	190.065126	0.169		
191.060365	31.6	$C_{13}H_7N_2$	191.060375	0.050	C6C6C11C42C3'	
191.072935	22.7	$C_{14}H_9N$	191.072951	0.086		
192.047759	44.7	$C_{10}H_{10}NOS$	192.047762	0.015	[C29C24'+H_2]	
192.068198	41.4	$C_{13}H_8N_2$	192.068200	0.012	[C4C6C11-H]	
192.080764	30.2	$C_{14}H_{10}N$	192.080776	0.063		
193.076013	25.4	$C_{13}H_9N_2$	193.076025	0.064	C4C6C11	
194.096419	43.7	$C_{14}H_{12}N$	194.096426	0.033	[C6C6C9C56C19'-H_2]	
195.067857	25.9	$C_{13}H_9NO$	195.067865	0.043	C4C6C13	
196.075700	24.3	$C_{13}H_{10}NO$	196.075690	-0.052	[C4C6C13+H]	
197.070940	29.3	$C_{12}H_9N_2O$	197.070939	-0.006	C5	
198.078777	12.7	$C_{12}H_{10}N_2O$	198.078764	-0.067	[C5+H]	
200.108125	170.6	$C_{10}H_{15}FNO_2$	200.108133	0.040	[C36'+H]	
203.060346	11.6	$C_{14}H_7N_2$	203.060375	0.144		
204.047758	147.0	$C_{11}H_{10}NOS$	204.047762	0.018	[C30C24'-H]	
205.055580	49.8	$C_{11}H_{11}NOS$	205.055587	0.033	[C30C24'+H_2]	
205.076023	162.4	$C_{14}H_9N_2$	205.076025	0.008	[C6C6C11C3'-H]	
206.063404	162.5	$C_{11}H_{12}NOS$	206.063412	0.040	[C30C24'+H_3]	
206.083846	97.2	$C_{14}H_{10}N_2$	206.083850	0.019	C6C6C11C3'	
207.067886	12.8	$C_{14}H_9NO$	207.067865	-0.100	C6C6C1'C14	
207.091666	37.6	$C_{14}H_{11}N_2$	207.091675	0.043	[C6C6C11C3'+H]	
208.075683	46.5	$C_{14}H_{10}NO$	208.075690	0.031	C6C6C13C3'	
209.083554	9.7	$C_{14}H_{11}NO$	209.083515	-0.186	[C6C6C1'C14+H_2]	
210.091334	34.1	$C_{14}H_{12}NO$	210.091340	0.030	[C6C6C13C3'+H_2]	
215.063733	19.6	$C_{12}H_{11}N_2S$	215.063746	0.062	[C25C32C33+H]	
216.047762	34.5	$C_{12}H_{10}NOS$	216.047762	-0.002	C30C46'	
218.083870	15.9	$C_{15}H_{10}N_2$	218.083850	-0.094	C6C6C11C2'	
219.055251	7.9	$C_{14}H_7N_2O$	219.055289	0.174	C6C6C42C2'	
219.058666	43.4	$C_{11}H_{11}N_2OS$	219.058661	-0.023	[C31C24'+H]	
219.091668	27.0	$C_{15}H_{11}N_2$	219.091675	0.031		
220.063103	24.7	$C_{14}H_8N_2O$	220.063114	0.050	[C6C6C3'-H_2]	
220.075703	34.4	$C_{15}H_{10}NO$	220.075690	-0.060	C6C6C1'C12	
220.099482	21.1	$C_{15}H_{12}N_2$	220.099500	0.084	[C6C6C11C2'+H_2]	
221.070942	205.2	$C_{14}H_9N_2O$	221.070939	-0.013	[C6C6C3'-H]	
222.078724	17.5	$C_{14}H_{10}N_2O$	222.078764	0.180	C6C6C3'	
222.091334	55.5	$C_{15}H_{12}NO$	222.091340	0.026	[C6C6C1'C12+H_2]	
223.062791	8.6	$C_{14}H_9NO_2$	223.062780	-0.047	C6C7C13C3'	
223.086613	25.3	$C_{14}H_{11}N_2O$	223.086589	-0.107	[C6C6C3'+H]	
224.070642	9.6	$C_{14}H_{10}NO_2$	224.070605	-0.164	[C6C7C13C3'+H]	
224.106974	27.9	$C_{15}H_{14}NO$	224.106990	0.073		
228.047784	36.1	$C_{13}H_{10}NOS$	228.047762	-0.097	[C30C23'-H_3]	
230.063408	67.7	$C_{13}H_{12}NOS$	230.063412	0.017	C30C23'	
232.024984	9.8	$C_8H_{15}NAg$	232.024993	0.038	C16C24	
232.079028	17.1	$C_{13}H_{14}NOS$	232.079062	0.148	[C30C23'+H_2]	
233.070945	115.4	$C_{15}H_9N_2O$	233.070939	-0.027	C6C6C2'	
234.078766	867.6	$C_{15}H_{10}N_2O$	234.078764	-0.008	[C6C6C2'+H]	

235.086595	318.7	$C_{15}H_{11}N_2O$	235.086589	-0.028	C6C6C10C1'	
236.070595	152.2	$C_{15}H_{10}NO_2$	236.070605	0.042	C6C7C12C1'	
236.094414	69.0	$C_{15}H_{12}N_2O$	236.094414	0.001	[C6C6C10C1'+H]	
238.086250	51.7	$C_{15}H_{12}NO_2$	238.086255	0.021	[C6C7C12C1'+H_2]	
244.079082	45.1	$C_{14}H_{14}NOS$	244.079062	-0.080	[C30C22'-H]	
246.094743	13.5	$C_{14}H_{16}NOS$	246.094712	-0.128	[C30C22'+H]	
247.086590	110.0	$C_{16}H_{11}N_2O$	247.086589	-0.003	[C6C6C1'-H]	
248.070593	30.6	$C_{16}H_{10}NO_2$	248.070605	0.048	[C6C13C2'-H_2]	
248.094417	89.8	$C_{16}H_{12}N_2O$	248.094414	-0.014	C6C6C1'	
249.065863	39.5	$C_{15}H_9N_2O_2$	249.065854	-0.036	C6C7C2'	
249.102237	1618.7	$C_{16}H_{13}N_2O$	249.102239	0.010	[C6C6C1'+H]	*
250.073657	19.7	$C_{15}H_{10}N_2O_2$	250.073679	0.089	[C6C7C2'+H]	
250.086316	14.3	$C_{16}H_{12}NO_2$	250.086255	-0.242	C6C13C2'	
250.110068	8.4	$C_{16}H_{14}N_2O$	250.110065	-0.010	[C6C6C1'+H_2]	
252.101911	174.8	$C_{16}H_{14}NO_2$	252.101905	-0.022	[C6C13C2'+H_2]	
258.094701	48.3	$C_{15}H_{16}NOS$	258.094712	0.043	C30C21'	
260.110364	45.4	$C_{15}H_{18}NOS$	260.110362	-0.009	[C30C21'+H]	
261.102241	36.7	$C_{17}H_{13}N_2O$	261.102239	-0.007	[C6C6C13C16'+H]	
262.073644	12.7	$C_{16}H_{10}N_2O_2$	262.073679	0.132	[C6C2'-H_2]	
263.081511	165.3	$C_{16}H_{11}N_2O_2$	263.081504	-0.026	[C6C2'-H]	
264.089334	967.5	$C_{16}H_{12}N_2O_2$	264.089329	-0.020	C6C2'	
266.081173	82.5	$C_{16}H_{12}NO_3$	266.081170	-0.010	[C12C2'-H_2]	
266.104935	20.1	$C_{16}H_{14}N_2O_2$	266.104979	0.165	[C6C2'+H_2]	
268.096819	40.8	$C_{16}H_{14}NO_3$	268.096820	0.004	C12C2'	
270.094671	19.6	$C_{16}H_{16}NOS$	270.094712	0.151	[C30C20'-H_3]	
272.110381	48.7	$C_{16}H_{18}NOS$	272.110362	-0.071	[C30C20'-H]	
274.126006	88.9	$C_{16}H_{20}NOS$	274.126012	0.023	[C30C20'+H]	
275.081525	14.2	$C_{17}H_{11}N_2O_2$	275.081504	-0.077	[C6C1'-H_3]	
277.097162	116.9	$C_{17}H_{13}N_2O_2$	277.097154	-0.030	C6C1'	
278.104981	262.3	$C_{17}H_{14}N_2O_2$	278.104979	-0.007	[C6C1'+H]	
279.112809	335.7	$C_{17}H_{15}N_2O_2$	279.112804	-0.016	C11C2'	
280.120615	19.6	$C_{17}H_{16}N_2O_2$	280.120629	0.048	C10C1'	
282.112486	191.1	$C_{17}H_{16}NO_3$	282.112470	-0.057	C12C1'	
284.091778	17.7	$C_{16}H_{14}NO_4$	284.091734	-0.155		
285.105639	26.1	$C_{16}H_{17}N_2OS$	285.105611	-0.098	[C19C30-H_2]	
285.160829	10.2	$C_{14}H_{22}FN_2O_3$	285.160897	0.239	[C33'+H]	
286.113516	14.6	$C_{16}H_{18}N_2OS$	286.113436	-0.280	[C31C20'-H_2]	
287.121269	271.9	$C_{16}H_{19}N_2OS$	287.121261	-0.028	C19C20'	
289.136915	116.8	$C_{16}H_{21}N_2OS$	289.136911	-0.014	[C31C20'+H]	
292.084239	9.7	$C_{17}H_{12}N_2O_3$	292.084244	0.016	[C7C1'-H_2]	
293.092051	34.8	$C_{17}H_{13}N_2O_3$	293.092069	0.060	[C2'-H_2]	
294.099895	204.5	$C_{17}H_{14}N_2O_3$	294.099894	-0.003	[C2'-H]	*
295.107744	42.8	$C_{17}H_{15}N_2O_3$	295.107719	-0.084	C2'	
297.123382	47.3	$C_{17}H_{17}N_2O_3$	297.123369	-0.045	[C2'+H_2]	
299.121261	59.1	$C_{17}H_{19}N_2OS$	299.121261	0.000	[C18C30-H_2]	
300.129181	7.0	$C_{17}H_{20}N_2OS$	300.129086	-0.316	C33C32C20'	
301.136919	150.1	$C_{17}H_{21}N_2OS$	301.136911	-0.028	C18C30	
303.152583	16.4	$C_{17}H_{23}N_2OS$	303.152561	-0.072	[C18C30+H_2]	
306.099909	28.3	$C_{18}H_{14}N_2O_3$	306.099894	-0.049	[C1'-H_3]	
306.123762	17.0	$C_{18}H_{16}N_3O_2$	306.123703	-0.191	C6C16'	
307.107722	162.2	$C_{18}H_{15}N_2O_3$	307.107719	-0.009	[C1'-H_2]	
308.115543	27.5	$C_{18}H_{16}N_2O_3$	308.115544	0.003	C1'	
309.123357	8815.8	$C_{18}H_{17}N_2O_3$	309.123369	0.040	[C1'+H]	
312.148055	18.1	$C_{15}H_{21}FN_2O_4$	312.147987	-0.219	[C31'-H]	
313.155819	288.3	$C_{15}H_{22}FN_2O_4$	313.155812	-0.023	C31'	
314.144770	27.7	$C_{18}H_{22}N_2OS$	314.144736	-0.110	C17C30	
315.152561	135.4	$C_{18}H_{23}N_2OS$	315.152561	-0.001	[C17C30+H]	
317.168176	10.7	$C_{18}H_{25}N_2OS$	317.168211	0.111	[C17C30+H_3]	
318.123672	18.9	$C_{19}H_{16}N_3O_2$	318.123703	0.096	[C6C17'-H_2]	
319.107755	14.4	$C_{19}H_{15}N_2O_3$	319.107719	-0.113	[C13C16'-H_4]	

319.131583	7.3	$C_{19}H_{17}N_3O_2$	319.131528	-0.171	C11C16'
320.139424	21.1	$C_{19}H_{18}N_3O_2$	320.139353	-0.221	C6C17'
322.118618	68.7	$C_{18}H_{16}N_3O_3$	322.118618	-0.001	[C15'-H]
323.139069	38.0	$C_{19}H_{19}N_2O_3$	323.139019	-0.155	[C36C53C21'-H_4]
324.134272	119.2	$C_{18}H_{18}N_3O_3$	324.134268	-0.012	
327.152596	40.7	$C_{19}H_{23}N_2OS$	327.152561	-0.107	[C16C30-H_2]
328.147741	22.4	$C_{18}H_{22}N_3OS$	328.147810	0.210	[C17C31-H_2]
329.168163	14.1	$C_{19}H_{25}N_2OS$	329.168211	0.145	C16C30
332.179142	15.4	$C_{18}H_{26}N_3OS$	332.179110	-0.096	C1C26'
333.114168	24.9	$C_{16}H_{19}N_3O_3S$	333.114164	-0.012	[C37C62+H_2]
334.155039	64.3	$C_{20}H_{20}N_3O_2$	334.155003	-0.108	C7C18'
335.139131	15.4	$C_{20}H_{19}N_2O_3$	335.139019	-0.335	[C13C17'-H_2]
336.134263	24.7	$C_{19}H_{18}N_3O_3$	336.134268	0.014	[C16'-H]
337.142109	97.4	$C_{19}H_{19}N_3O_3$	337.142093	-0.047	C16'
338.149942	68.4	$C_{19}H_{20}N_3O_3$	338.149918	-0.069	[C16'+H]
339.152492	21.1	$C_{20}H_{23}N_2OS$	339.152561	0.204	[C15C30-H_4]
340.147830	624.9	$C_{19}H_{22}N_3OS$	340.147810	-0.059	[C1C29-H_2]
350.149928	143.8	$C_{20}H_{20}N_3O_3$	350.149918	-0.030	C17'
351.157702	15.1	$C_{20}H_{21}N_3O_3$	351.157743	0.116	[C17'+H]
352.165560	77.8	$C_{20}H_{22}N_3O_3$	352.165568	0.023	[C17'+H_2]
353.173378	41.2	$C_{20}H_{23}N_3O_3$	353.173393	0.042	[C17'+H_3]
354.163466	601.9	$C_{20}H_{24}N_3OS$	354.163460	-0.017	[C1C30-H]
355.171303	183.6	$C_{20}H_{25}N_3OS$	355.171285	-0.049	[C1C30-H_2]
356.179130	572.8	$C_{20}H_{26}N_3OS$	356.179110	-0.055	[C15C31-H_2]
357.186959	49.2	$C_{20}H_{27}N_3OS$	357.186935	-0.066	C1C30
358.194737	25.8	$C_{20}H_{28}N_3OS$	358.194760	0.063	C15C31
365.160842	27.0	$C_{20}H_{21}N_4O_3$	365.160817	-0.067	C70C20'
366.181266	28.1	$C_{21}H_{24}N_3O_3$	366.181218	-0.132	[C18'+H]
367.132191	24.5	$C_{17}H_{23}N_2O_5S$	367.132219	0.076	
368.179141	187.0	$C_{21}H_{26}N_3OS$	368.179110	-0.084	[C15C33-H_2O]
370.194770	59.4	$C_{21}H_{28}N_3OS$	370.194760	-0.027	C2C30
372.173965	6.8	$C_{20}H_{26}N_3O_2S$	372.174025	0.160	C16C33
372.197863	120.5	$C_{20}H_{28}N_4OS$	372.197834	-0.077	C1C31
374.149932	34.6	$C_{22}H_{20}N_3O_3$	374.149918	-0.038	[C19'-H_4]
379.176501	44.9	$C_{21}H_{23}N_4O_3$	379.176467	-0.089	[C70C20+H_2]
382.158554	6.6	$C_{21}H_{24}N_3O_2S$	382.158375	-0.469	[C15C33-H_4]
382.182218	9.4	$C_{21}H_{26}N_4OS$	382.182184	-0.090	C1C33C32
385.205464	6.6	$C_{21}H_{29}N_4OS$	385.205659	0.507	C2C31
391.176587	25.8	$C_{22}H_{23}N_4O_3$	391.176467	-0.306	[C20-H_2]
392.184288	48.9	$C_{22}H_{24}N_4O_3$	392.184292	0.010	[C20-H]
393.192102	102.8	$C_{22}H_{25}N_4O_3$	393.192117	0.037	C20
395.207833	67.8	$C_{22}H_{27}N_4O_3$	395.207767	-0.167	[C20+H_2]
398.177071	19.3	$C_{21}H_{26}N_4O_2S$	398.177099	0.071	[C1C33-H]
400.168970	203.2	$C_{21}H_{26}N_3O_3S$	400.168939	-0.077	[C25C40C57+H_2]
400.192615	10.6	$C_{21}H_{28}N_4O_2S$	400.192749	0.334	C32C39C41C24'
405.192379	7.5	$C_{23}H_{25}N_4O_3$	405.192117	-0.645	[C21-H_2]
406.158313	6.5	$C_{23}H_{24}N_3O_2S$	406.158375	0.154	[C32C40C41C46'-H_2]
407.207749	134.0	$C_{23}H_{27}N_4O_3$	407.207767	0.045	C21
412.168885	15.9	$C_{22}H_{26}N_3O_3S$	412.168939	0.131	
413.200580	31.3	$C_{22}H_{29}N_4O_2S$	413.200574	-0.015	
419.211134	25.4	$C_{21}H_{31}N_4O_3S$	419.211139	0.012	C39C62
421.223477	22.0	$C_{24}H_{29}N_4O_3$	421.223417	-0.142	C22
423.184896	7.7	$C_{23}H_{27}N_4O_2S$	423.184924	0.066	[C17C32C36-H_4]
425.200602	14.2	$C_{23}H_{29}N_4O_2S$	425.200574	-0.065	[C17C32C36-H_2]
426.184624	11.4	$C_{23}H_{28}N_3O_3S$	426.184589	-0.082	[C17C48'-H_2]
426.208406	13.8	$C_{23}H_{30}N_4O_2S$	426.208399	-0.015	[C17C32C36-H]
427.216362	15.0	$C_{23}H_{31}N_4O_2S$	427.216224	-0.322	C17C32C36
429.195397	10.4	$C_{22}H_{29}N_4O_3S$	429.195488	0.211	[C25C39+H]
431.211213	37.8	$C_{22}H_{31}N_4O_3S$	431.211139	-0.172	[C18C36+H_2]

436.073399	6.4	$C_{19}H_{25}N_2OSAg$	436.073303	-0.219	C16C30	
441.195453	24.9	$C_{23}H_{29}N_4O_3S$	441.195488	0.080	C35C39C46'	
445.226817	133.6	$C_{23}H_{33}N_4O_3S$	445.226789	-0.062	[C17C36+H_2]	
449.200515	11.0	$C_{25}H_{29}N_4O_2S$	449.200574	0.131	C25C32C41C57C65	
455.211034	9.0	$C_{24}H_{31}N_4O_3S$	455.211139	0.230	C35C39C23'	
459.238959	6.5	$C_{27}H_{31}N_4O_3$	459.239067	0.235	[C24-H_4]	
461.254730	192.3	$C_{27}H_{33}N_4O_3$	461.254717	-0.028	[C24-H_2]	
463.270368	600.0	$C_{27}H_{35}N_4O_3$	463.270367	-0.002	C24	*
465.219487	22.8	$C_{25}H_{31}N_5O_2S$	465.219298	-0.405	[C1C35C36-H_2]	
467.211018	14.7	$C_{25}H_{31}N_4O_3S$	467.211139	0.258	[C15C36-H_4]	
468.242807	37.0	$C_{25}H_{34}N_5O_2S$	468.242773	-0.072	C19C32C35C39C66	
470.222082	141.4	$C_{24}H_{32}N_5O_3S$	470.222038	-0.093	C19C32C39C57	
480.242828	32.5	$C_{26}H_{34}N_5O_2S$	480.242773	-0.116	C19C35C39C57	
483.230147	20.6	$C_{25}H_{33}N_5O_3S$	483.229863	-0.589	C19C39C41C57C57	
484.237678	84.8	$C_{28}H_{29}FN_6O$	484.238139	0.952	[C1C38C58-H_3O]	
485.245539	389.1	$C_{25}H_{35}N_5O_3S$	485.245513	-0.053	C1C36	*
498.253344	58.6	$C_{26}H_{36}N_5O_3S$	498.253338	-0.012	C19C39C41C57	
513.240343	29.6	$C_{26}H_{35}N_5O_4S$	513.240427	0.163	C19C39C57	
591.142744	183.5	$C_{28}H_{29}FN_6OAg$	591.143231	0.823	[C1C38C58-H_3O]	*
618.312282	10.5	$C_{31}H_{45}FN_5O_5S$	618.311995	-0.463	[C19+H_2]	
630.311907	26.6	$C_{32}H_{45}FN_5O_5S$	630.311995	0.139		
642.311897	16.9	$C_{33}H_{45}FN_5O_5S$	642.311995	0.153	[C17-H]	
643.319795	198.4	$C_{33}H_{46}FN_5O_5S$	643.319820	0.038	C17	
644.327678	289.3	$C_{33}H_{47}FN_5O_5S$	644.327645	-0.051	[C17+H]	
656.327851	12.2	$C_{34}H_{47}FN_5O_5S$	656.327645	-0.314	[C16-H]	
658.343221	136.8	$C_{34}H_{49}FN_5O_5S$	658.343296	0.113	[C16+H]	
662.279637	7.1	$C_{38}H_{40}N_5O_4S$	662.279552	-0.128	[C30-H_4]	
664.295240	33.2	$C_{38}H_{42}N_5O_4S$	664.295203	-0.055	C6C6C45C48'	
665.303290	26.3	$C_{38}H_{43}N_5O_4S$	665.303028	-0.394	[C6C6C8'-H_4]	
666.310840	168.0	$C_{38}H_{44}N_5O_4S$	666.310853	0.020	C30	
669.322907	74.2	$C_{34}H_{46}FN_6O_5S$	669.322894	-0.019	[C1C57-H]	
670.343371	14.4	$C_{35}H_{49}FN_5O_5S$	670.343296	-0.111	C15	
681.321963	17.9	$C_{38}H_{45}N_6O_4S$	681.321752	-0.310	C31	
682.330750	52.6	$C_{35}H_{47}FN_6O_5S$	682.330719	-0.046	[C1-H_3]	
683.338633	100.8	$C_{35}H_{48}FN_6O_5S$	683.338544	-0.130	[C1-H_2]	
684.346165	6631.9	$C_{35}H_{49}FN_6O_5S$	684.346370	0.299	[C1-H]	
686.341951	264.2	$C_{37}H_{46}N_6O_7$	686.342249	0.435	C7C7C51C68	
697.354133	66.4	$C_{36}H_{50}FN_6O_5S$	697.354195	0.088	[C2-H]	
708.285256	15.8	$C_{39}H_{42}N_5O_6S$	708.285032	-0.316		
709.316838	22.4	$C_{39}H_{45}N_6O_5S$	709.316666	-0.243	C33	
723.177331	7.7	$C_{31}H_{41}FN_4O_6SAg$	723.177627	0.410	C20'	
725.176939	10.3	$C_{48}H_{27}N_3O_3S$	725.176764	-0.242		
759.333060	5.5	$C_{43}H_{47}N_6O_5S$	759.332316	-0.979	C6C13C35C37	
776.358609	18.2	$C_{43}H_{50}N_7O_5S$	776.358865	0.330	C35C36	
787.218833	5.7	$C_{38}H_{44}N_6O_4SAg$	787.219018	0.234	[C6C6C39C8'-H_6]	
789.235123	16.6	$C_{38}H_{46}N_6O_4SAg$	789.234668	-0.576	[C31+H]	
790.243630	21.2	$C_{35}H_{48}FN_6O_5SAg$	790.243636	0.007	[C1-H_2]	*
791.252010	6.3	$C_{35}H_{49}FN_6O_5SAg$	791.251461	-0.694	[C1-H]	
794.369514	103.6	$C_{43}H_{52}N_7O_6S$	794.369430	-0.106	[C36+H]	
822.363992	31.9	$C_{44}H_{52}N_7O_7S$	822.364345	0.430	C37	
900.266258	40.8	$C_{43}H_{51}N_7O_6SAg$	900.266697	0.488	C36	
901.275192	21.3	$C_{43}H_{52}N_7O_6SAg$	901.274522	-0.744	[C36+H]	
902.282360	95.8	$C_{43}H_{53}N_7O_6SAg$	902.282347	-0.014	[C36+H_2]	*
908.392220	12.7	$C_{52}H_{56}N_6O_7S$	908.392571	0.386		

952.443422	15.6	$C_{51}H_{63}FN_7O_8S$	952.443738	0.331	C28
957.288122	40.0	$C_{45}H_{54}N_8O_7SAg$	957.288160	0.040	C38C39
958.291838	18.2	$C_{49}H_{52}FN_8O_3SAg$	958.291255	-0.609	[C6C6-Me_2][C6C6-O_3]
965.439584	20.1	$C_{51}H_{62}FN_8O_8S$	965.438987	-0.618	C57C57
970.346125	9.4	$C_{50}H_{63}AgN_4O_7S$	970.346290	0.170	
979.454747	61.0	$C_{52}H_{64}FN_8O_8S$	979.454637	-0.112	C57
991.453775	45.5	$C_{53}H_{64}FN_8O_8S$	991.454637	0.870	[M-H_3]
993.470124	145.8	$C_{53}H_{66}FN_8O_8S$	993.470287	0.164	[M-H]
994.478580	181.0	$C_{53}H_{67}FN_8O_8S$	994.478112	-0.470	[M+H]
1075.367778	56.1	$C_{51}H_{65}FN_8O_8SAg$	1075.367554	-0.208	C61
1101.381374	10923.3	$C_{53}H_{67}FN_8O_8SAg$	1101.383204	1.662	[M+Ag]
			Abs mean error	0.112	
			Mean std dev	0.160	

Table S29 showing peak list, signal-to-noise ratio (S/N), elemental composition, and assignment with mass errors (ppm) of the $[M-H]^-$ VZ185 compound by CID MS/MS, calibration points are marked by an asterisk (✱).

**Measured *m/z***	**S/N**	**Elemental Composition**	**Theoretical *m/z***	**Assignment Error (ppm)**	**Assignment**	
200.108160	19.1	$C_{10}H_{15}FNO_2$	200.108133	-0.133	C36'	
244.637899	25.0	$C_{25}H_{39}N_5O_3S$	244.638132	0.952	[C1C36+H_2][C1C36+]	
309.123369	1774.0	$C_{18}H_{17}N_2O_3$	309.123369	0.001	C1'	✱
313.155821	70.5	$C_{15}H_{22}FN_2O_4$	313.155812	-0.027	[C31'-H]	
333.659062	307.8	$C_{38}H_{45}N_5O_4S$	333.659065	0.010	[C6C6C8'-H_4]	
334.121902	7.9	$C_{16}H_{20}N_3O_3S$	334.121989	0.259	[C37C62+H_2]	
358.194791	54.5	$C_{20}H_{28}N_3OS$	358.194760	-0.085	C1C30	
394.261440	46.2	$C_{20}H_{33}FN_5O_2$	394.261280	-0.406		
397.688429	19.8	$C_{43}H_{53}N_7O_6S$	397.688353	-0.191	[C36]2+]	
398.696182	48610.6	$C_{43}H_{55}N_7O_6S$	398.696178	-0.011	[C36+H_2][C36+]	
442.237624	68.9	$C_{29}H_{32}NO_3$	442.237670	0.104		
457.282318	16.4	$C_{22}H_{38}FN_4O_5$	457.282075	-0.531	[C16C71C38+H_6]	
460.248181	34.2	$C_{29}H_{34}NO_4$	460.248235	0.117		
463.270459	16.5	$C_{27}H_{35}N_4O_3$	463.270367	-0.199	[C24-H]	
486.253343	18.4	$C_{25}H_{36}N_5O_3S$	486.253338	-0.010	C1C36	✱
488.269007	24830.0	$C_{25}H_{38}N_5O_3S$	488.268988	-0.038	[C1C36+H_2]	
498.246630	27029.1	$C_{53}H_{69}FN_8O_8S$	498.246607	-0.046	[M+H_2][M+]	
654.310777	9.1	$C_{37}H_{44}N_5O_4S$	654.310853	0.116	C29	
666.310832	28.7	$C_{38}H_{44}N_5O_4S$	666.310853	0.031	[C6C6C8'-H_4]	✱
683.337379	475.4	$C_{38}H_{47}N_6O_4S$	683.337402	0.034	[C31+H]	
687.369812	2354.0	$C_{35}H_{52}FN_6O_5S$	687.369845	0.047	[C1+H_2]	
796.385098	3227.0	$C_{43}H_{54}N_7O_6S$	796.385080	-0.022	[C36+H_2]	✱
498.246630	27029.1	$C_{53}H_{69}FN_8O_8S$	498.246607	-0.046	[M+H_2][M+]	
			Abs mean error	0.150		
			Mean std dev	0.239		

Table S30 showing peak list, signal-to-noise ratio (S/N), elemental composition, and assignment with mass errors (ppm) of the [M-H]$^-$ VZ185 compound by UVPD MS/MS, calibration points are marked by an asterisk (✱).

Measured *m/z*	S/N	Elemental Composition	Theoretical *m/z*	Assignment Error (ppm)	Assignment	
112.112076	7.3	$C_7H_{14}N$	112.112076	-0.004	[C17C24-H]	✱
153.138627	53.0	$C_9H_{17}N_2$	153.138625	-0.014	[C1C24-H_2]	
155.154273	430.0	$C_9H_{19}N_2$	155.154275	0.015	C1C24	✱
172.113214	715.0	$C_9H_{15}FNO$	172.113219	0.028	[C37'-H]	
200.108134	757.0	$C_{10}H_{15}FNO_2$	200.108133	-0.007	C36'	✱
204.047783	14.5	$C_{11}H_{10}NOS$	204.047762	-0.102	C30C24'	
206.063405	46.5	$C_{11}H_{12}NOS$	206.063412	0.033	[C30C24'+H_2]	
218.063447	14.6	$C_{12}H_{12}NOS$	218.063412	-0.161	C30C46'	
219.058649	10.4	$C_{11}H_{11}N_2OS$	219.058661	0.056	C31C24'	
221.070944	12.5	$C_{14}H_9N_2O$	221.070939	-0.024	[C6C6C3'-H_2]	
223.086567	9.1	$C_{14}H_{11}N_2O$	223.086589	0.099	C6C6C3'	
230.063376	9.5	$C_{13}H_{12}NOS$	230.063412	0.156	[C30C23'-H_2]	
230.106122	8.9	$C_{10}H_{15}FN_2O_3$	230.106122	0.000	[C40C33'+H]	
234.078771	89.7	$C_{15}H_{10}N_2O$	234.078764	-0.028	C6C6C2'	
235.086599	25.3	$C_{15}H_{11}N_2O$	235.086589	-0.041	[C6C6C2'+H]	
236.070635	21.6	$C_{15}H_{10}NO_2$	236.070605	-0.127	[C6C12C2'-H_2]	
237.102331	9.9	$C_{15}H_{13}N_2O$	237.102239	-0.387	[C6C6C10C1'+H]	
248.061394	8.1	$C_{12}H_{12}N_2O_2S$	248.061400	0.025	[C33C24'+H]	
248.094427	9.2	$C_{16}H_{12}N_2O$	248.094414	-0.051	[C6C6C1'-H]	
249.102254	285.4	$C_{16}H_{13}N_2O$	249.102239	-0.059	C6C6C1'	
249.124558	359.5	$C_{10}H_{18}FN_2O_4$	249.124512	-0.186		
250.110070	9.7	$C_{16}H_{14}N_2O$	250.110064	-0.024	[C6C6C1'+H]	
251.081578	7.2	$C_{15}H_{11}N_2O_2$	251.081504	-0.296	[C6C7C2'+H]	
251.117908	14.0	$C_{16}H_{15}N_2O$	251.117890	-0.073	[C6C6C1'+H_2]	
252.101902	26.2	$C_{16}H_{14}NO_2$	252.101905	0.013	C6C12C1'	
258.094753	9.8	$C_{15}H_{16}NOS$	258.094712	-0.158	[C30C21'-H_2]	
261.102289	7.6	$C_{17}H_{13}N_2O$	261.102239	-0.190	C6C6C13C16'	
263.081525	24.7	$C_{16}H_{11}N_2O_2$	263.081504	-0.082	[C6C2'-H_2]	
264.089350	149.8	$C_{16}H_{12}N_2O_2$	264.089329	-0.079	[C6C2'-H]	
265.097139	8.9	$C_{16}H_{13}N_2O_2$	265.097154	0.056	C6C2'	
266.081198	11.7	$C_{16}H_{12}NO_3$	266.081170	-0.106	C13C42C2'	
274.126040	76.7	$C_{16}H_{20}NOS$	274.126012	-0.101	C30C20'	
276.141719	9.3	$C_{16}H_{22}NOS$	276.141662	-0.208	[C30C20'+H_2]	
277.097191	14.4	$C_{17}H_{13}N_2O_2$	277.097154	-0.135	[C6C1'-H_2]	
278.104994	44.1	$C_{17}H_{14}N_2O_2$	278.104979	-0.054	C6C1'	
279.076524	7.6	$C_{16}H_{11}N_2O_3$	279.076419	-0.375	C2'C7C11	
279.112826	111.7	$C_{17}H_{15}N_2O_2$	279.112804	-0.080	[C6C1'+H]	
280.120648	73.0	$C_{17}H_{16}N_2O_2$	280.120629	-0.067	C11C2'	
281.128469	55.2	$C_{17}H_{17}N_2O_2$	281.128454	-0.053	C10C1'	
282.112492	39.4	$C_{17}H_{16}NO_3$	282.112470	-0.077	C13C2'	
287.121268	24.6	$C_{16}H_{19}N_2OS$	287.121261	-0.023	[C31C20'-H_2]	✱
289.136925	50.3	$C_{16}H_{21}N_2OS$	289.136911	-0.049	C31C20'	
294.099933	40.4	$C_{17}H_{14}N_2O_3$	294.099894	-0.131	[C2'-H_2]	
295.107743	102.3	$C_{17}H_{15}N_2O_3$	295.107719	-0.082	[C2'-H]	
301.136934	92.1	$C_{17}H_{21}N_2OS$	301.136911	-0.075	C33C32C20'	
303.152603	11.8	$C_{17}H_{23}N_2OS$	303.152561	-0.138	C32C33C20'	
307.107730	19.7	$C_{18}H_{15}N_2O_3$	307.107719	-0.035	[C1'-H_3]	
309.123395	8064.9	$C_{18}H_{17}N_2O_3$	309.123369	-0.083	C1'	
310.131212	521.1	$C_{18}H_{18}N_2O_3$	310.131194	-0.058	[C1'+H]	
313.136964	6.9	$C_{18}H_{21}N_2OS$	313.136911	-0.169	[C17C30-H_2]	
313.155842	152.4	$C_{15}H_{22}FN_2O_4$	313.155812	-0.096	[C31'-H]	
315.152558	39.8	$C_{18}H_{23}N_2OS$	315.152561	0.009	C17C30	✱
317.168230	23.7	$C_{18}H_{25}N_2OS$	317.168211	-0.060	[C17C30+H_2]	

320.139382	34.7	$C_{19}H_{18}N_3O_2$	320.139353	-0.091	C11C16'	
322.118653	15.5	$C_{18}H_{16}N_3O_3$	322.118618	-0.108	[C15'-H_2]	
323.139038	10.0	$C_{19}H_{19}N_2O_3$	323.139019	-0.060	C13C16'	
324.134327	15.9	$C_{18}H_{18}N_3O_3$	324.134268	-0.181	C15'	
327.152551	33.7	$C_{19}H_{23}N_2OS$	327.152561	0.031	[C16C30-H_3]	
329.168243	27.7	$C_{19}H_{25}N_2OS$	329.168211	-0.096	C16C30	
332.179115	7.1	$C_{18}H_{26}N_3OS$	332.179110	-0.016	[C1C29C44+H_2]	
333.114214	25.6	$C_{16}H_{19}N_3O_3S$	333.114164	-0.151	[C36C24'+H]	
333.659104	121.8	$C_{38}H_{45}N_5O_4S$	333.659065	-0.117	[C6C6C8'-H_4]	
334.155024	8.8	$C_{20}H_{20}N_3O_2$	334.155003	-0.063	C6C18'	
334.160773	53.4	$C_{47}H_{44}N_2S$	334.160712	-0.182		
337.142095	25.0	$C_{19}H_{19}N_3O_3$	337.142093	-0.007	[C16'-H]	*
338.149966	49.2	$C_{19}H_{20}N_3O_3$	338.149918	-0.143	C16'	
341.168266	21.9	$C_{36}H_{47}FN_4O_8$	341.168348	0.242		
342.163382	7.0	$C_{19}H_{24}N_3OS$	342.163460	0.228	C2C29C44	
344.179116	8.1	$C_{19}H_{26}N_3OS$	344.179110	-0.018	C16C31	
346.194791	26.3	$C_{19}H_{28}N_3OS$	346.194760	-0.089	[C31C16-H_2]	
350.149940	18.3	$C_{20}H_{20}N_3O_3$	350.149918	-0.061	[C17'-H_2]	
351.157815	26.9	$C_{20}H_{21}N_3O_3$	351.157743	-0.204	C17'	
352.165586	43.4	$C_{20}H_{22}N_3O_3$	352.165568	-0.051	[C17'+H]	
356.179135	67.0	$C_{20}H_{26}N_3OS$	356.179110	-0.069	[C1C30-H_2]	
357.186978	10.8	$C_{20}H_{27}N_3OS$	357.186935	-0.120	[C15C31-H_2]	
358.194800	653.9	$C_{20}H_{28}N_3OS$	358.194760	-0.112	C1C30	
366.181314	15.2	$C_{21}H_{24}N_3O_3$	366.181218	-0.262	C18'	
370.194864	7.0	$C_{21}H_{28}N_3OS$	370.194760	-0.281	[C2C30-H_2]	
374.189669	13.2	$C_{20}H_{28}N_3O_2S$	374.189675	0.017	[C16C33+H]	
375.221407	24.9	$C_{36}H_{59}FN_8O_8$	375.221446	0.103		
388.683138	43.4	$C_{43}H_{51}N_7O_5S$	388.683071	-0.172		
397.688408	101.1	$C_{43}H_{53}N_7O_6S$	397.688353	-0.139	[C36]2+]	
398.696226	13515.4	$C_{43}H_{55}N_7O_6S$	398.696178	-0.119	[C36+H_2][C36+]	
400.169008	54.1	$C_{21}H_{26}N_3O_3S$	400.168939	-0.173	C36C20	
401.200604	9.6	$C_{21}H_{29}N_4O_2S$	401.200574	-0.076	C32C39C41C24'	
402.184705	17.7	$C_{21}H_{28}N_3O_3S$	402.184589	-0.289	[C36C20+H_2]	
407.207863	14.1	$C_{23}H_{27}N_4O_3$	407.207767	-0.235	[C21-H]	
413.200606	24.5	$C_{22}H_{29}N_4O_2S$	413.200574	-0.077	C2C33	
426.199104	37.7	$C_{45}H_{56}N_8O_7S$	426.199085	-0.045	[C38C39]2+]	
426.700807	21.3	$C_{49}H_{54}FN_8O_3S$	426.700632	-0.410	[C6C6-Me_2][C6C6-O_3]	
431.211249	33.9	$C_{22}H_{31}N_4O_3S$	431.211139	-0.255	C36C18	
432.727415	23.6	$C_{48}H_{63}N_7O_6S$	432.727478	0.145	[C40C69+H][C40C69+]	
433.229259	12.7	$C_{20}H_{30}FN_8S$	433.229269	0.022		
440.267026	40.9	$C_{26}H_{36}N_2O_4$	440.266959	-0.152	C40C53C20'	
442.262375	17.4	$C_{23}H_{40}NO_5S$	442.262171	-0.462		
445.226846	42.4	$C_{23}H_{33}N_4O_3S$	445.226789	-0.128	C36C17	
463.270462	35.4	$C_{27}H_{35}N_4O_3$	463.270367	-0.205	[C24-H]	
468.242773	23.6	$C_{25}H_{34}N_5O_2S$	468.242773	0.001	C1C35C36	*
471.242532	27.9	$C_{25}H_{35}N_4O_3S$	471.242439	-0.197	[C39C22'-O]	
486.253370	24.0	$C_{25}H_{36}N_5O_3S$	486.253338	-0.067	C1C36	
488.269047	2047.7	$C_{25}H_{38}N_5O_3S$	488.268988	-0.121	[C1C36+H_2]	
490.734850	19.0	$C_{52}H_{66}FN_8O_8S$	490.734869	0.038	[C57]2+]	
497.742762	866.6	$C_{53}H_{68}FN_8O_8S$	497.742694	-0.137	[M-H]	
498.246631	30328.6	$C_{53}H_{69}FN_8O_8S$	498.246607	-0.048	[M+H_2][M+]	
630.311986	24.7	$C_{32}H_{45}FN_5O_5S$	630.311995	0.014	[C18+H]	
642.312316	7.0	$C_{33}H_{45}FN_5O_5S$	642.311995	-0.500	[C17-H_2]	
643.320250	6.0	$C_{33}H_{46}FN_5O_5S$	643.319820	-0.668	[C17-H]	
644.327627	42.8	$C_{33}H_{47}FN_5O_5S$	644.327645	0.027	C17	*
658.343361	7.5	$C_{34}H_{49}FN_5O_5S$	658.343295	-0.100	C16	
683.337378	70.3	$C_{38}H_{47}N_6O_4S$	683.337402	0.035	[C31+H]	
685.354197	24.4	$C_{35}H_{50}FN_6O_5S$	685.354195	-0.003	[C1-H]	*
686.362214	17.5	$C_{35}H_{51}FN_6O_5S$	686.362020	-0.283	C1	
687.369801	488.1	$C_{35}H_{52}FN_6O_5S$	687.369845	0.064	[C1+H_2]	

689.365647	24.0	$C_{40}H_{52}FN_3O_4S$	689.365708	0.089	
796.384899	719.0	$C_{43}H_{54}N_7O_6S$	796.385080	0.227	[C36+H_2]
995.485943	6.6	$C_{53}H_{68}FN_8O_8S$	995.485937	-0.006	[M+H]
498.246631	30328.6	$C_{53}H_{69}FN_8O_8S$	498.246607	-0.048	[M+H_2][M+]
			Abs mean error	0.114	
			Mean std dev	0.110	
